# Single cell RNA sequencing uncovers cellular developmental sequences and novel potential intercellular communications in embryonic kidney

**DOI:** 10.1038/s41598-020-80154-y

**Published:** 2021-01-08

**Authors:** Isao Matsui, Ayumi Matsumoto, Kazunori Inoue, Yusuke Katsuma, Seiichi Yasuda, Karin Shimada, Yusuke Sakaguchi, Masayuki Mizui, Jun-ya Kaimori, Yoshitsugu Takabatake, Yoshitaka Isaka

**Affiliations:** 1 Department of Nephrology, Osaka University Graduate School of Medicine, 2-2 Yamada-oka, Suita, Osaka 565-0871 Japan; 2 Department of Inter-Organ Communication Research in Kidney Disease, Osaka University Graduate School of Medicine, 2-2 Yamada-oka, Suita, Osaka 565-0871 Japan

**Keywords:** Developmental biology, Nephrology

## Abstract

Kidney development requires the coordinated growth and differentiation of multiple cells. Despite recent single cell profiles in nephrogenesis research, tools for data analysis are rapidly developing, and offer an opportunity to gain additional insight into kidney development. In this study, single-cell RNA sequencing data obtained from embryonic mouse kidney were re-analyzed. Manifold learning based on partition-based graph-abstraction coordinated cells, reflecting their expected lineage relationships. Consequently, the coordination in combination with ForceAtlas2 enabled the inference of parietal epithelial cells of Bowman’s capsule and the inference of cells involved in the developmental process from the S-shaped body to each nephron segment. RNA velocity suggested developmental sequences of proximal tubules and podocytes. In combination with a Markov chain algorithm, RNA velocity suggested the self-renewal processes of nephron progenitors. NicheNet analyses suggested that not only cells belonging to ureteric bud and stroma, but also endothelial cells, macrophages, and pericytes may contribute to the differentiation of cells from nephron progenitors. Organ culture of embryonic mouse kidney demonstrated that nerve growth factor, one of the nephrogenesis-related factors inferred by NicheNet, contributed to mitochondrial biogenesis in developing distal tubules. These approaches suggested previously unrecognized aspects of the underlying mechanisms for kidney development.

## Introduction

The complex process of kidney development requires the coordinated growth and differentiation of multiple cells. Single cell RNA sequencing (scRNA-seq) is a rapidly developing technology that enables the systematic analysis of complex *in vivo* phenotypes at a cellular level^1^. Therefore, single cell analysis has recently been gaining considerable attention in nephrogenesis research. Tools for data analysis are rapidly developing, and offer an opportunity to gain additional insights into kidney development.

In the analyses of scRNA-seq data, cells are divided into clusters. The use of clustering of cells assumes that scRNA-seq data is composed of biologically distinct groups^1^. In contrast to clustering, trajectory analysis assumes that data lie on a connected manifold, and enables a better interpretation of continuous processes such as development. As summarized by Saelens et al., currently available trajectory inference tools can be categorized based on inferable trajectory types: linear, bifurcation, multifurcation, tree, cycle, connected, or disconnected^2^. For the comprehensive analysis of kidney development, the ability of an inference tool to evaluate disconnected trajectories is especially important, because cells of various origins play essential roles in the kidney development in a cooperative manner^3–5^. After assessing more than 70 tools, Saelens et al*.* concluded that partition-based graph abstraction (PAGA), which provides interpretable graph-like maps of data manifolds, was the best approach with which to analyze disconnected trajectories^2^.

In this study, we analyzed several scRNA-seq datasets obtained from the National Center for Biotechnology Information (NCBI) Sequence Read Archive (SRA). The trajectories of mouse embryonic kidney, mouse adult kidney, and human kidney organoid have been calculated using PAGA^6^. In combination with PAGA-initialized ForceAtlas2, RNA velocity analyses of mouse and human embryonic kidneys have been used, because PAGA and RNA velocity are two complementary approaches to study cell differentiation^7^. NicheNet has been applied to compute intercellular communication in nephrogenesis of mice^8^. These approaches suggested novel aspects of kidney development.

## Results

### Mapping of developing mouse kidney scRNA-seq data using PAGA and PAGA-initialized ForceAtlas2 in combination with RNA velocity

**Figure 1:**
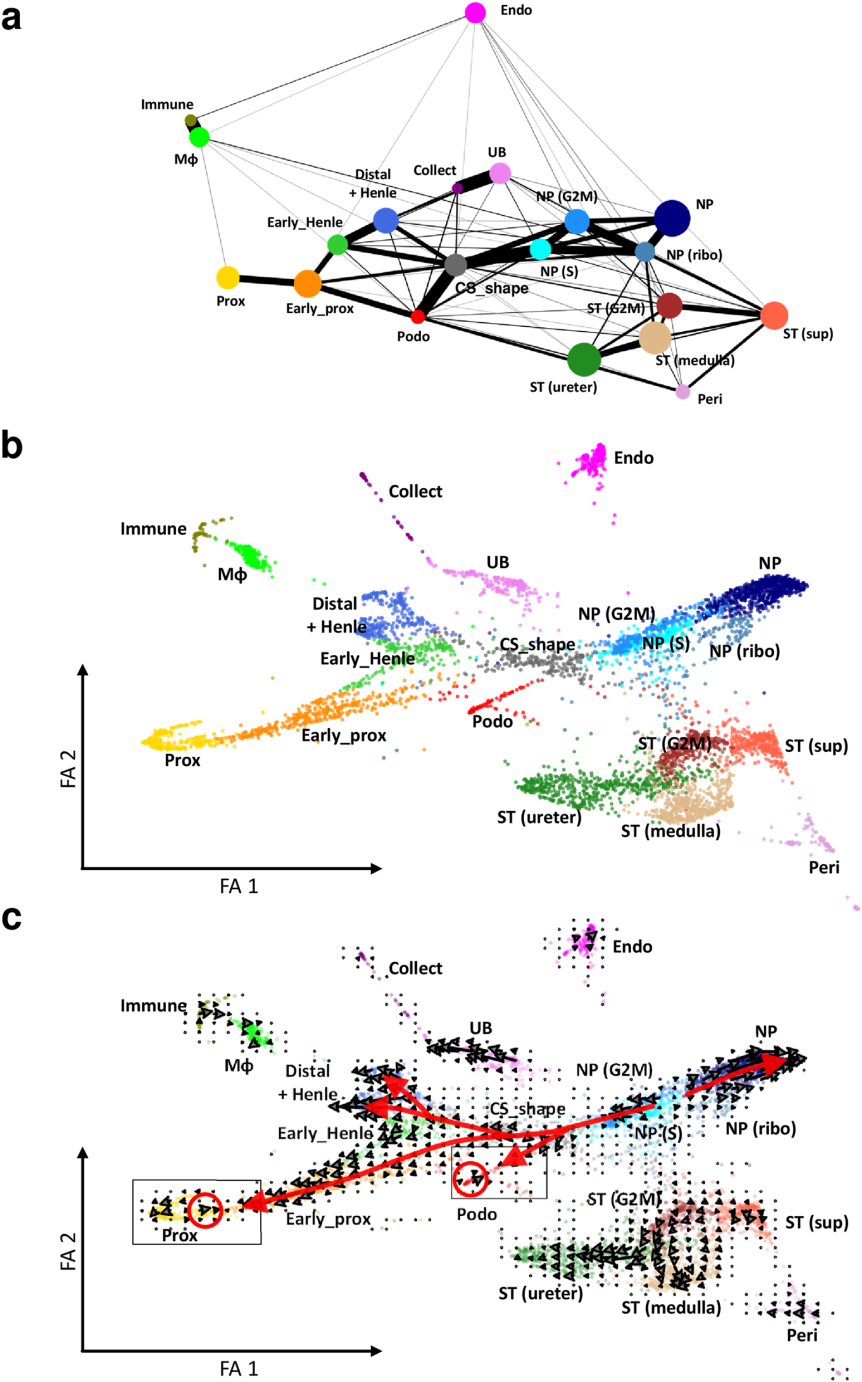
ForceAtlas2 initialized by partition-based graph abstraction (PAGA) coordinated cells in a way that reflects expected lineage relationships. (**a**) Coarse-grained abstraction of single cell RNA-sequencing (scRNA-seq) data obtained from embryonic mouse kidney at E18.5 is shown. The Louvain method was applied to calculate clusters. The size of the node reflects the number of cells belonging to the corresponding cluster, while the width of the edge shows the strength of the connectivity. (**b**) Cells were mapped by PAGA-initialized ForceAtlas2. (**c**) Grid-embedded RNA velocities are overlaid onto the map. Red arrows indicate the manually abstracted flow of the RNA velocities. Cells in black squares were reanalyzed, and the results presented in Fig. 3. RNA velocities in red circles are in the opposite direction compared to the majority of RNA velocities within the corresponding and the adjacent clusters. Data were analyzed using Scanpy version 1.4.4.post1 (https://scanpy.readthedocs.io/en/stable/) and scVelo version 0.1.25 (https://scvelo.readthedocs.io/). Abbreviations: NP, nephron progenitor; NP (S), S-phase nephron progenitor; NP (G2M), G2M-phase nephron progenitor; NP (ribo), ribosome-enriched nephron progenitor; CS_shape, comma and S shaped bodies; Podo, podocyte; Early_prox, early phase proximal tubule; Prox, proximal tubule; Early_Henle, early phase Henle; Distal + Henle, distal tubule and the loop of Henle; UB, ureteric bud; Collect, collecting duct; ST (G2M), G2M-phase stroma; ST (sup), superficial stroma; ST (medulla), medullary stroma; ST (ureter), ureter-associated stroma; Peri, pericyte; Endo, endothelium; Mφ, macrophage; Immune, immune cell; FA, ForceAtlas2.

Single cell RNA-seq data of embryonic mouse kidney from day 18.5 were pre-processed as described in the Methods section and Supplementary Fig. S1. Cells were divided into clusters using the Louvain method (Fig. 1a and Supplementary Table S1-1). Because clustering *per se* provides no information about the connectivity among the clusters, we calculated the trajectories among the annotated clusters using PAGA (Fig. 1a)^1^. Without any information about the origins of the constituent cells in the developing kidney, PAGA generated a graph-like map that reflects the developmental origin of each cluster (Fig. 1a). In Fig. 1a, the size of a node reflects the number of cells belonging to the corresponding cluster, while the width of an edge shows the strength of the trajectory. Among the constituent cells of the developing kidney, endothelia and immune-related cells were inferred to be the cell types most different from the rest of the cell types (Fig. 1a). The trajectories between the ureteric bud-derived cells and the nephron progenitor derived cells were weaker than those within each of these two groups of cells (Fig. 1a). The trajectories within the stromal cells were weaker than those within the ureteric bud-derived cells, and those within the nephron progenitor derived cells (Fig. 1a). The weaker trajectories within the stromal cells at embryonic day 18.5 might reflect the stromal heterogeneity, which was recently reported by England et al.^9^ We further analyzed scRNA-seq data obtained from mouse adult kidney, mouse embryonic kidney at day 14.5, and human kidney organoid, using PAGA (Supplementary Fig. S2)^10–12^. Reflecting the differentiation of the kidney constituent cells, the trajectories among proximal tubules, loop of Henle, and distal tubules in the adult mouse kidney were weak (Fig. 1a, Supplementary Fig. S2a, and Supplementary Table S1-2). Both in the analysis of embryonic mouse kidney at day 14.5 and in human kidney organoids, endothelial cells were inferred to be different from the rest of the cell types (Supplementary Fig. S2b, c, Supplementary Tables 1-3, and 1-4). The trajectories within the stromal cells at embryonic day 14.5 were stronger than those at embryonic day 18.5, suggesting that the stromal heterogeneity reported by England et al. might be achieved between embryonic days 14.5 and 18.5^9^.

PAGA-initialized ForceAtlas2 mapped cells in an easily interpretable manner (Fig. 1b). The cluster annotation shown in Fig. 1 was performed based on the expression patterns of the marker genes shown in Supplementary Table S1-1 and Supplementary Figs. S3, and S4. The expression patterns of all 3,000 highly variable genes, which were extracted during the pre-processing of the scRNA-seq data (Supplementary Fig. S1b), are summarized in Supplementary Fig. S5 in alphanumeric order. Although PAGA provides information about the strength of trajectories, PAGA alone cannot identify the direction of the trajectories. Therefore, we calculated the RNA velocity, and projected the velocity onto the PAGA-initialized ForceAtlas2 map (Fig. 1c)^13^.

### PAGA-initialized ForceAtlas2 generated an interpretable lineage-preserving map of mouse embryonic kidney

PAGA-initialized ForceAtlas2 interpretably mapped the origins of two types of tubular cells (Fig. 1b). All tubular cells originating from nephron progenitors were coordinated separately from tubular cells originating from ureteric buds, while the distance between the distal tubule and the ureteric buds, two cell types connected to each other during nephrogenesis, was calculated to be the shortest between the nephron progenitor-derived and the ureteric bud-derived tubular cells (Fig. 1b). Cells were mapped in a way that reflects the expected lineage relationships. Wnt family member 11 (*Wnt11*), a molecule upregulated in response to Ret proto-oncogene (*Ret*) activation at the tip of the ureteric bud, was distributed within the ureteric bud (UB) cluster and was away from the collecting duct (Collect) cluster (Fig. 1b and Supplementary Fig. S3-3a)^14^. Wnt9b, a signaling molecule required to induce renal vesicles at close proximity to the stalk of the ureteric bud, was expressed in the UB cluster cells next to the Collect cluster (Fig. 1b and Supplementary Fig. S3-3a). As Thiagarajan et al. reported, ureteric bud-derived tubular cells express distinct markers in different anatomical locations^15^. Solute carrier organic anion transporter family member 4A1 (*Slco4c1)*, a marker of the ureteric bud tip, and uroplakin 1B (*Upk1b)*, a marker of the collecting duct root, were expressed in the UB cluster and at the outer edge of the Collect cluster, respectively (Fig. 1b and Supplementary Fig. S3-3). The expression patterns of uroplakin 3A (*Upk3a)* and protein phosphatase 1 regulatory subunit 3C (*Ppp1r3C)* also reflected the anatomical topology reported by Thiagarajan et al. (Supplementary Fig. S3-3b)^15^.

**Figure 2:**
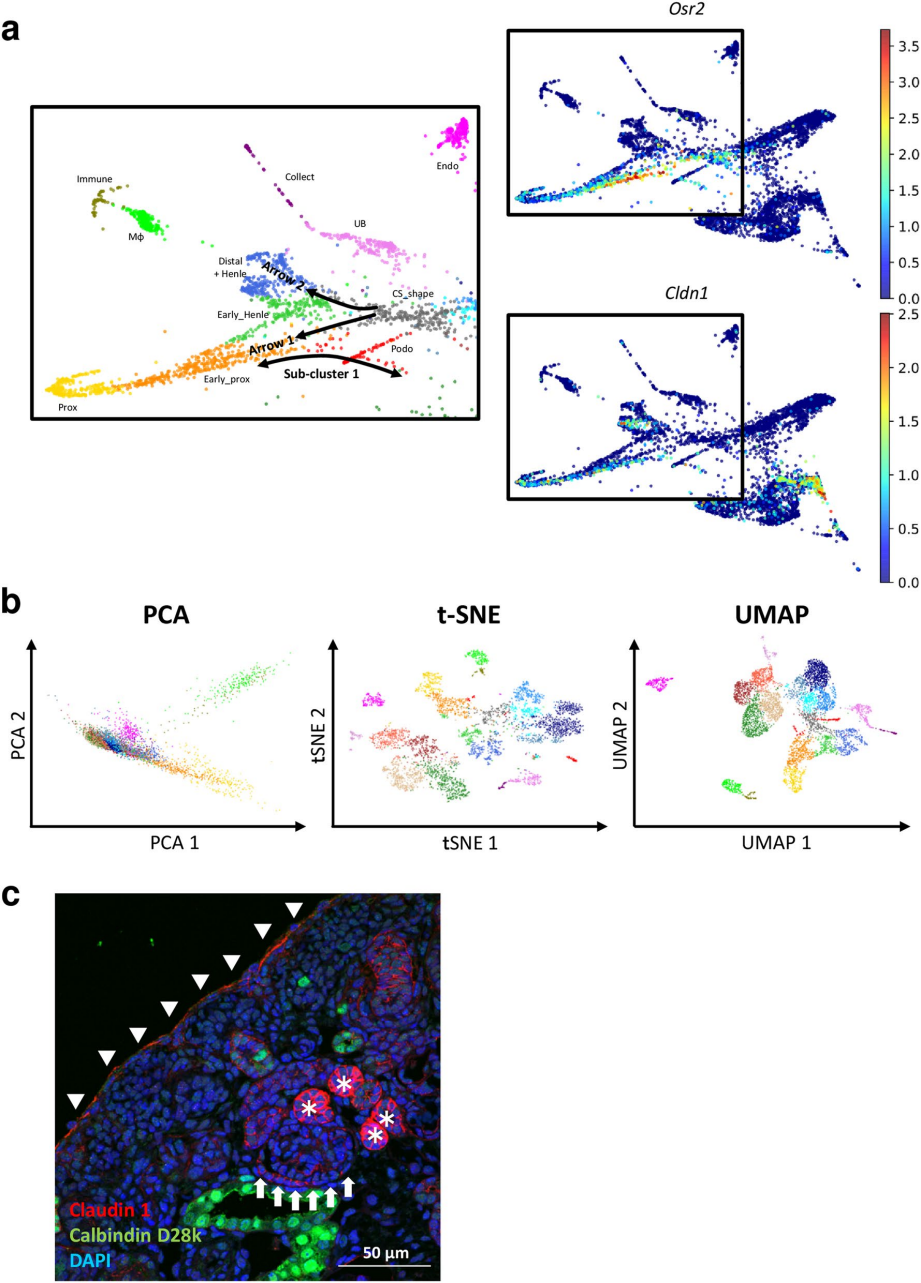
PAGA-initialized ForceAtlas2 enabled the identification of a sub-cluster that represented the Bowman’s capsule. (**a**) The left portion of Fig. 1b is shown with the addition of several annotating arrows. Expression levels of odd-skipped related 2 (*Osr2)* and claudin-1 (*Cldn1)* are also presented. Three black squares indicate the same portion in the PAGA-initialized ForceAtlas2 map. (**b**) Cells were visualized with principal component analysis (PCA), t-distributed stochastic neighbor embedding (t-SNE), and uniform manifold approximation and projection (UMAP). Dot colors indicate clusters shown in Fig 1. (**c**) Mouse embryonic kidney section at day 18.5, immunohistochemically stained with claudin 1 in red, calbindin D28k in green, and 4’,6-diamidino-2-phenylindole (DAPI) in blue (scale bar: 50 µm). Data were analyzed using Scanpy version 1.4.4.post1 (https://scanpy.readthedocs.io/en/stable/). Arrows indicate the parietal epithelial cells of the Bowman’s capsule. Arrowheads and asterisks indicate the surface of the kidney and the distal tubules, respectively.

Two aligned cell lines are depicted in the PAGA-initialized ForceAtlas2 map (Fig. 2a; arrows 1 and 2). Lindström et al. reported that a *Wnt4*-positive domain in the developing S-shaped body provides a register for the positioning of proximal cell fates^16^. In accordance with the report, *Wnt4*-positive cells were enriched in arrow 1 (Fig. 2a and Supplementary Fig. S3-4c). Arrow 1 also involves cells positive for delta-like protein 1 (*Dll1)*, whose hypomorphic allele results in reduction of proximal tubule formation (Fig. 2a and Supplementary S3-4**c**)^17^. Arrow 2 was secreted frizzled related protein 2 (*Sfrp2)-*positive (Fig. 2a and Supplementary Fig. S3-4c). Because the expression of *Sfrp2*, an antagonist of Wnt signaling, is enriched at the distal tubular side of the comma- and S-shaped bodies, arrow 2 also reflects the expected lineage relationships^18^. Arrow 2 was also enriched in cells positive for fibroblast growth factor 8 (*Fgf8)*, whose hypomorphs result in severely truncated tubular segments (Fig. 2a and Supplementary Fig. S3-4c)^19^.

Although the Louvain algorithm separated cells into 20, apparently meaningful clusters (Fig. 1), PAGA-initialized ForceAtlas2 suggested that a sub-cluster exists across the early phase proximal tubule (Early_prox) and the podocyte (Podo) clusters (sub-cluster 1 in Fig. 2a). It was difficult to identify sub-cluster 1 using principal component analysis (PCA), t-distributed stochastic neighbor embedding (t-SNE), or uniform manifold approximation and projection (UMAP) (Fig. 2b). Because there was no gene specifically expressed in sub-cluster 1, we could not fully characterize this sub-cluster (Supplementary Fig. S5). However, the expression pattern of odd-skipped related 2 (*Osr2)* suggested that sub-cluster 1 might represent the Bowman’s capsule; Lan et al. have reported that the *Osr2*^*IresCre*^ mouse expresses Cre recombinase in the developing proximal tubules and in the outer edge of developing glomeruli (Fig. 2a)^20^. Among 3,000 highly variable genes, claudin-1 (*Cldn1)*, a key component of tight junctions, was expressed along with sub-cluster 1, although it was not specific to this sub-cluster (Fig. 2a). Immunofluorescent staining for claudin 1 demonstrated that claudin 1 was distributed in the Bowman’s capsule, proximal tubules, distal tubules, and superficial stromal cells, in accordance with the expression pattern of *Cldn1* in the scRNA-seq analysis (Fig. 2c).

### RNA velocity analyses identified the self-renewal process of nephron progenitors

**Figure 3:**
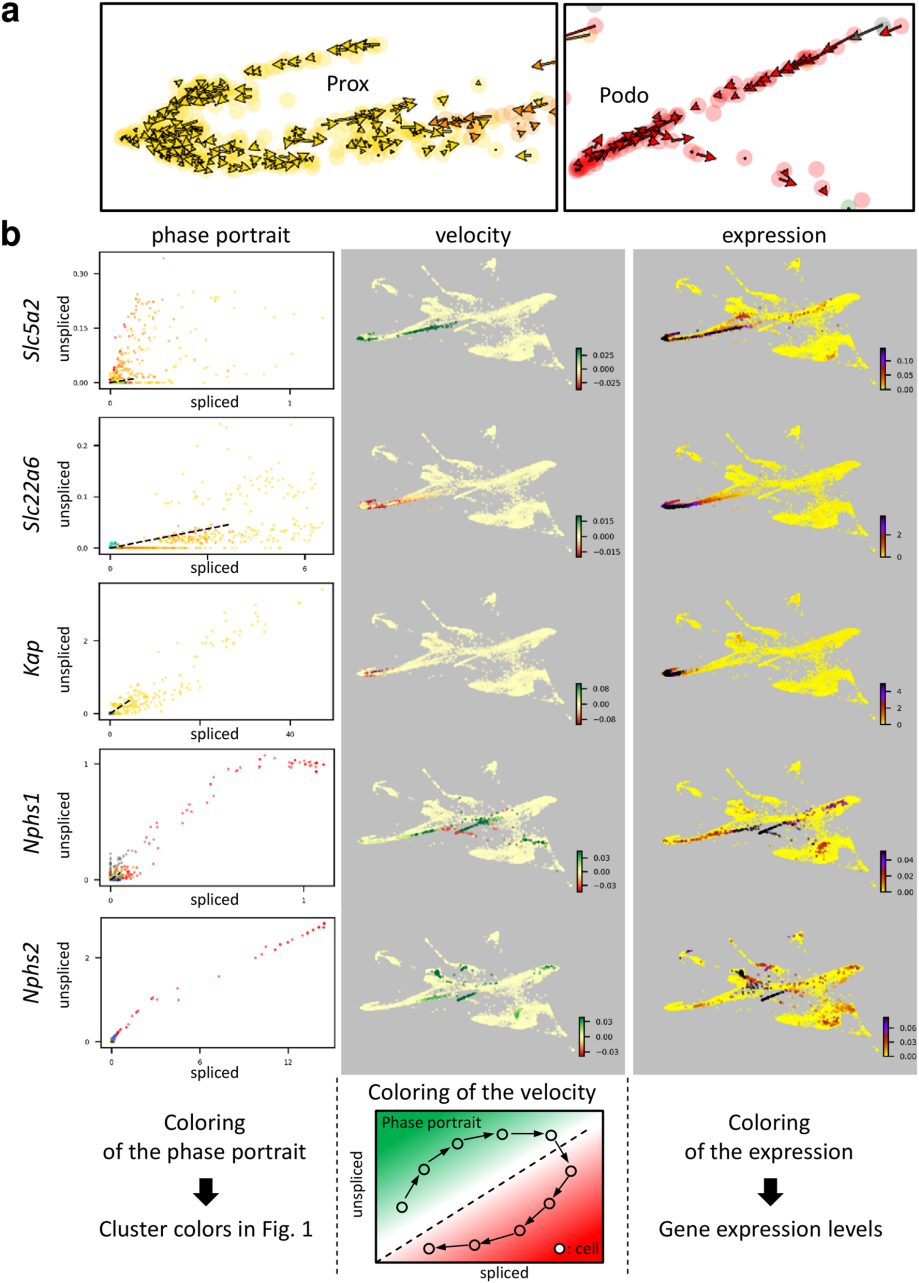
RNA velocities in the proximal tubule (Prox) and podocyte (Podo) clusters. (**a**) RNA velocities of the cells belonging to the squared regions in Fig. 1c are shown at single-cell resolution. (**b**) The phase portrait, RNA velocity, and expression levels of marker genes are summarized. Dot colors in the phase portraits correspond to cluster colors in Fig. 1. Dotted lines in the phase portraits indicate the steady-state ratio. Colors of the velocity plot indicate where the cells were allocated in the corresponding phase portraits. The dynamic ranges of RNA expression shown here are different from those in Supplementary Fig. S3. Dynamic ranges here are adjusted to show RNA expression, including low levels. Data were analyzed using Scanpy version 1.4.4.post1 (https://scanpy.readthedocs.io/en/stable/) and scVelo version 0.1.25 (https://scvelo.readthedocs.io/).

Although the majority of the RNA velocities shown in Fig. 1c were directed in an easily interpretable manner, several arrows in the proximal tubule (Prox) and the Podo clusters were in the opposite direction to the majority of RNA velocities in that cluster (Fig. 3a and red circles in Fig. 1c). The velocities in the nephron progenitor (NP) cluster were calculated to be in the opposite direction compared to those in the comma and S shaped bodies (CS_shape) and G2M-phase nephron progenitor (NP (G2M)) clusters (Fig. 1c).

To clarify why several arrows in the Prox cluster were in the opposite direction from the majority, levels of proximal tubular segment specific marker genes were visualized (Fig. 3b and Supplementary Fig. S6). The expression pattern of sodium/glucose cotransporter 2 (*Slc5a2)* a marker of the S1 segment, solute carrier family 22 member 6 (*Slc22a6*), a marker of the S2 segment, and kidney androgen-regulated protein (*Kap),* a marker of the S3 segment, were separately mapped by PAGA-initialized ForceAtlas2 (Supplementary Fig. S6). A color map presentation of the RNA velocity indicated that the tip of the Prox cluster and the parietal epithelial cells of the Bowman’s capsule had positive velocities for *Slc5a2*, while cells belonging to the S2 and S3 segments had negative velocities for *Slc22a6* and *Kap*, respectively (Fig. 3b). Because an opposite velocity in the Prox cluster was observed in the S2/S3 segment cells, the negative velocities of *Slc22a6* and *Kap* explained why the arrows in the root of the Prox cluster were mapped in the opposite direction (Figs. 1c, 3 and Supplementary Fig. S6). These findings suggested that differentiation of the S2 and S3 segments occurred before the differentiation of the S1 segment.

The RNA velocities of nephrin (*Nphs1)* and podocin (*Nphs2*) genes were also analyzed. In contrast to the *Nphs2* velocity, the *Nphs1* velocity at the tip of the Podo cluster was negative (Fig. 3b), indicating that *Nphs1* velocity explains, at least in part, why RNA velocities at the Podo cluster tip were in opposite directions (Figs. 1c, 3). We further analyzed scRNA-seq data obtained from human embryonic kidney at embryonic week 18 (Supplementary Fig. S7). This scRNA-seq data contain a large population of podocytes (Supplementary Fig. S7a). Analyses of RNA velocity demonstrated that both mouse and human embryonic kidney cells had similar directionality in RNA velocity (Fig. 1c and Supplementary Fig. S7a). The velocity of the nephrin, but not of the podocin gene, at the tip of the podocyte cluster was negative, not only in the embryonic mouse kidney, but also in the embryonic human kidney (Fig. 3b and Supplementary Fig. S7b). These findings suggested that the nephrin and podocin genes were not induced simultaneously in the podocyte developmental process. Putaala et al. have reported that *Nphs1*-Cre mice express Cre recombinase in podocytes at E13 or later, while Brunskill et al. demonstrated that *Nphs2*-Cre mice express Cre recombinase at E14.5 or later^21,22^. To obtain further insight into the developmental process of podocytes, we analyzed the RNA velocity of the highly expressed genes at the tip of podocyte cluster in human embryonic kidney data (cluster 0 in Supplementary Table 2 and Supplementary Fig. S7a). Although *MYL9, SPOCK2, PTH1R, MME*, and *AIF1* were similarly expressed in cluster 0 (expression column in Supplementary Fig. S8), the induction of these genes was not simultaneous in the development of podocytes (velocity column in Supplementary Fig. S8), indicating that the complex maturation of podocytes cannot be fully understood solely from conventional analyses of mRNA expression levels.

**Figure 4:**
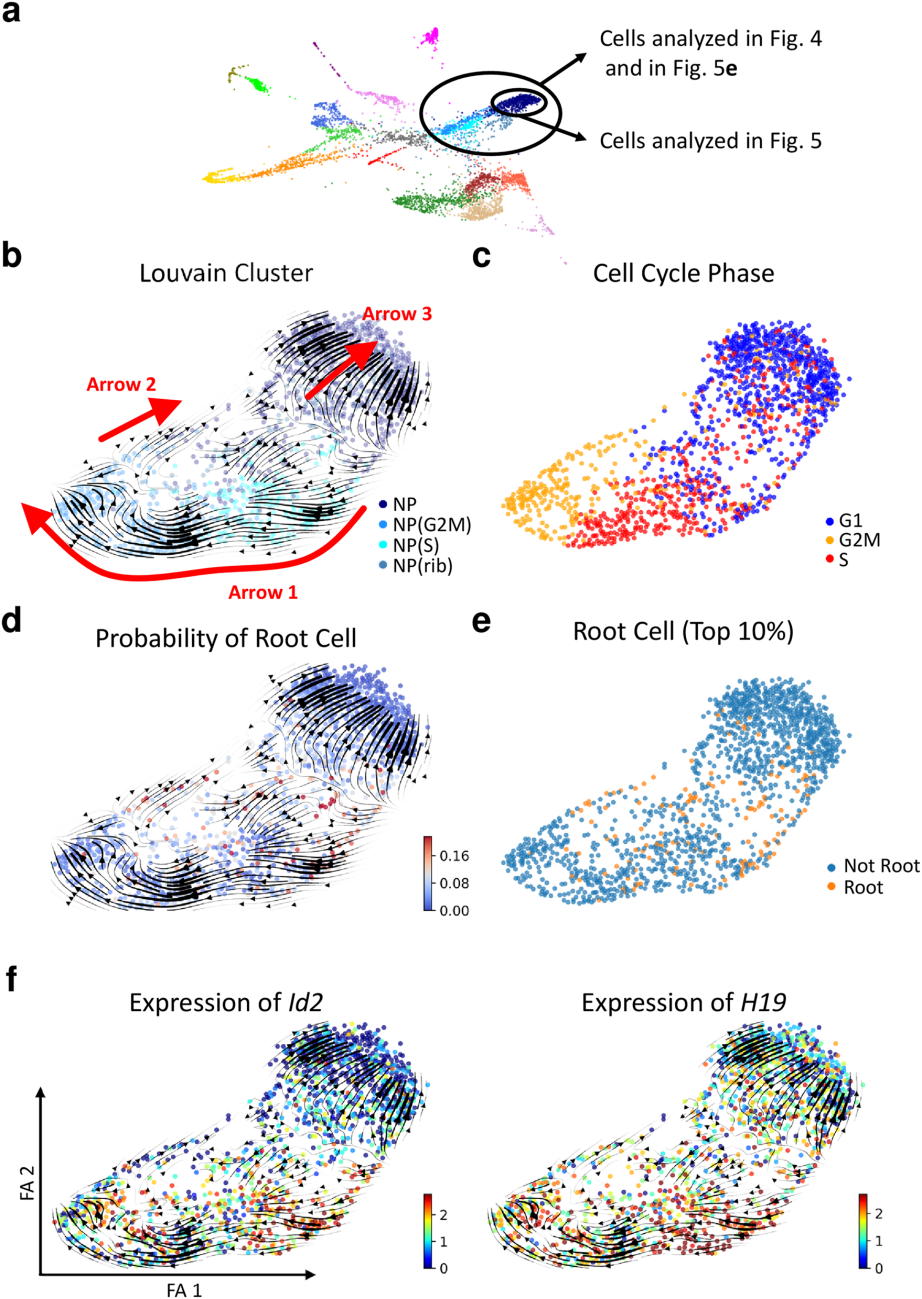
Identification of cells with high root probability in the nephron progenitors. (**a**) A subset consisting of the nephron progenitor (NP), S-phase NP (NP(S)), G2M-phase NP (NP(G2M)), and ribosome-enriched NP (NP(ribo)) clusters was analyzed. (**b**) The subset was mapped by PAGA-initialized ForceAtlas2. Stream-embedded RNA velocities were overlaid. Three major RNA velocity streams (arrows 1, 2, and 3) are observed. (**c**) The cell cycle phase is visualized. (**d**) The probability of RNA velocity root is calculated using CellRank. Red dots indicate cells with higher root probability. (**e**) Cells with top 10% root probability are visualized. (**f**) mRNA expression levels of *Id2* and *H19* are shown. Data were analyzed using Scanpy version 1.4.4.post1 (https://scanpy.readthedocs.io/en/stable/), scVelo version 0.1.25 (https://scvelo.readthedocs.io/), and CellRank version 1.1.dev264+g61bbda4 (https://cellrank.readthedocs.io/en/stable/#).

**Figure 5:**
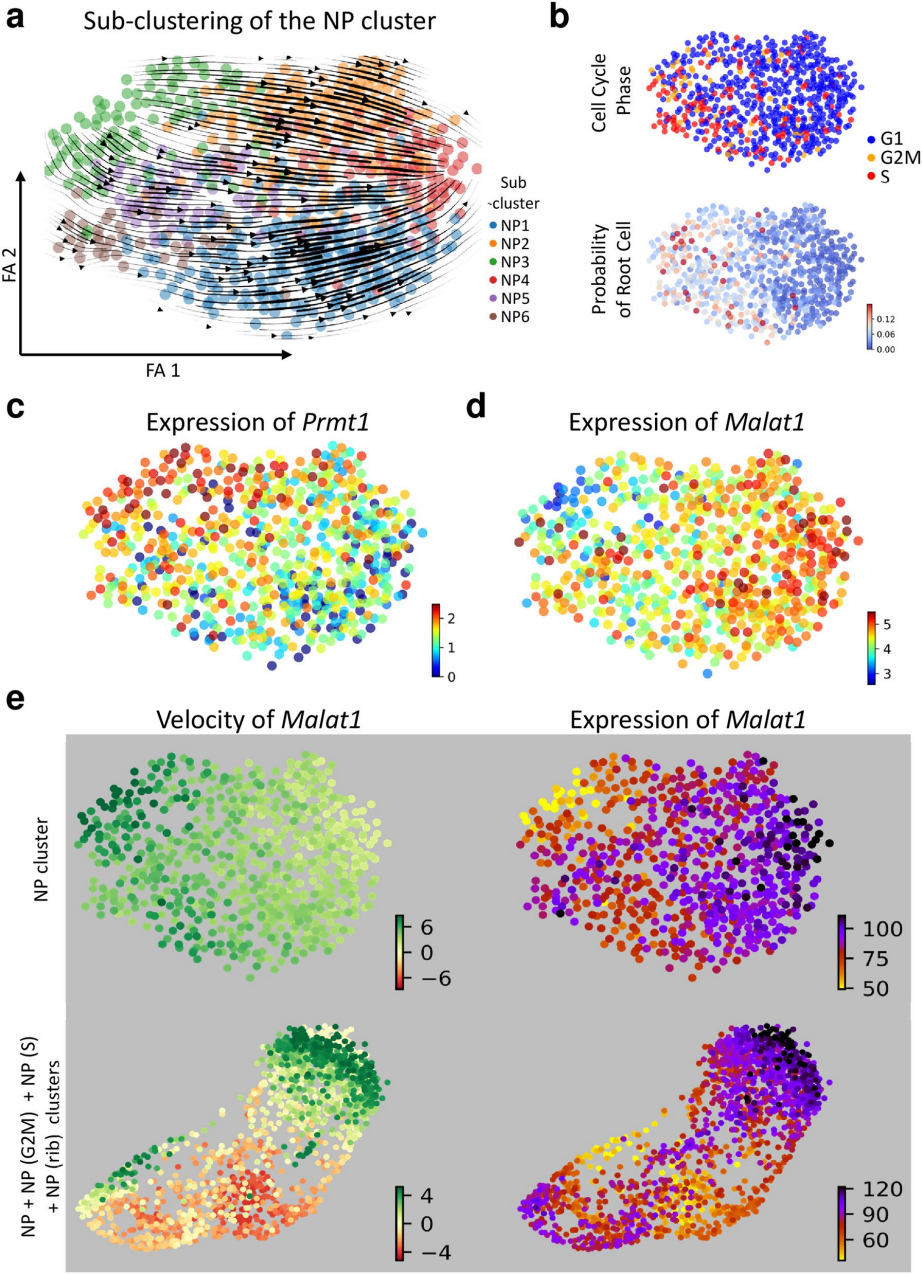
Analyses of cells belonging to the NP cluster. (**a**) The NP cluster cells have been extracted and then divided into six sub-clusters using the Louvain algorithm. Stream-embedded RNA velocities are overlaid. (**b**) The cell cycle phase and the probability of root cell in the NP cluster are visualized. (**c**) Expression levels of protein arginine methyltransferase 1 (*Prmt1)* and metastasis associated lung adenocarcinoma transcript 1 (*Malat1)* are visualized. (**e**) RNA velocity and mRNA expression levels of *Malat1* in the NP cluster and the NP + NP(G2M) + NP (S) + NP (rib) clusters are shown. Data were analyzed using Scanpy version 1.4.4.post1 (https://scanpy.readthedocs.io/en/stable/), scVelo version 0.1.25 (https://scvelo.readthedocs.io/), and CellRank version 1.1.dev264+g61bbda4 (https://cellrank.readthedocs.io/en/stable/#).

To understand why the velocity of the NP cluster was in the opposite direction compared to those of the CS_shape, S-phase NP (NP (S)), and NP(G2M) clusters (Fig. 1c), a cell subset composed of the NP, NP (S), NP (G2M), and ribosome-enriched NP (NP (ribo)) clusters was extracted, and mapped onto the PAGA-initialized ForceAtlas2 map, which was overlaid with the stream-embedded RNA velocity (Fig. 4a,b). There were three major RNA velocity streams in the subset (arrows 1, 2, and 3 in Fig. 4b). Because we annotated several nephron progenitor-related clusters based on cell cycle phase, the cell cycle phase is shown in Fig. 4c. Using the RNA velocity in combination with a Markov chain, the root of RNA velocity in the subset analyzed in Fig. 4 was inferred. Cells with high root-probability were distributed at the gap between the NP (S)/NP (G2M) clusters and the NP/NP (ribo) clusters (Fig. 4d). We selected the cells with the top 10% probability of being at the root of RNA velocity, and investigated the differentially expressed features in the root cells (Fig. 4e and Supplementary Table 3-1). The root cells expressed inhibitor of DNA binding 2 (*Id2),* a gene that has been shown to enhance the proliferation of stem cells (Fig. 4f and Supplementary Table 3-1)^23^. The root cells were also enriched in long non-coding RNA *H19*, which has been demonstrated to induce differentiation of stem cells (Fig. 4f and Supplementary Table 3-1)^24^. Therefore, the root of RNA velocity inferred by the Markov chain consisted of nephron progenitor cells that initiated proliferation and/or differentiation, but not cells in quiescent status. Arrow 1 in Fig. 4b represented cellular proliferation and differentiation from nephron progenitors to comma shaped bodies. In contrast to the arrow 1, which was easily interpretable, RNA velocity in the opposite direction was also observed at the gap between the NP (S)/NP (G2M) clusters and the NP/NP (ribo) clusters (arrow 2 in Fig. 4b). The direction of RNA velocities in the NP cluster was also opposite to arrow 1 in Fig. 4b (abstracted as arrow 3 in Fig. 4b). Fig. 4 indicated that the RNA velocities of the NP cluster stemmed from the gap, where cells that initiated proliferation exist, and we therefore hypothesized that the RNA velocity in the NP cluster represented the self-renewal of nephron progenitors. We also hypothesized that arrow 2 in Fig. 4b played some roles in the self-renewal process of nephron progenitors. To test this hypothesis, cells of the NP cluster were extracted and separated into six sub-clusters (Fig. 5a). RNA velocity in the NP cluster stemmed from sub-cluster NP3 and ended in NP4 (Fig. 5a). The NP cluster contained cells in G2M or S phase, and cells with high probability of being at the root of RNA velocity, and these cells were distributed mainly in the left hand portion of the plot, indicating that the cells belonging to the NP cluster became quiescent along the RNA velocity (Fig. 5b). Because the RNA velocity in the NP cluster stemmed from sub-cluster NP3 and ended in NP4, differentially expressed features between the NP3 and NP4 sub-clusters were examined (Supplementary Table S3-2). Protein arginine methyltransferase 1 *(Prmt1)* and metastasis associated lung adenocarcinoma transcript 1 (*Malat1)* were enriched in the NP3 and the NP4 sub-cluster, respectively (Supplementary Table S3-2, Fig. 5c,d). Because *Prmt1* is an inhibitor of stem cell self-renewal, while the long non-coding RNA *Malat1* is an enhancer of cellular stemness phenotypes, the RNA velocity in the NP cluster might represent the self-renewal of nephron progenitors^25–28^. The RNA velocity of *Malat1* was high in the NP3 sub-cluster, where mRNA expression levels of *Malat1* was low, and the RNA velocity of *Malat1* was neutral in the NP4 sub-cluster, where mRNA expression levels of *Malat1* was the highest (upper panel of Fig. 5e). The RNA velocity of *Malat1* was also high in the cells that showed the opposite RNA velocity at the gap between the NP (S)/NP (G2M) clusters and the NP/NP (ribo) clusters (arrow 2 in Figs. 4b,5e).

### NicheNet revealed previously unrecognized intercellular communication in nephrogenesis

**Figure 6:**
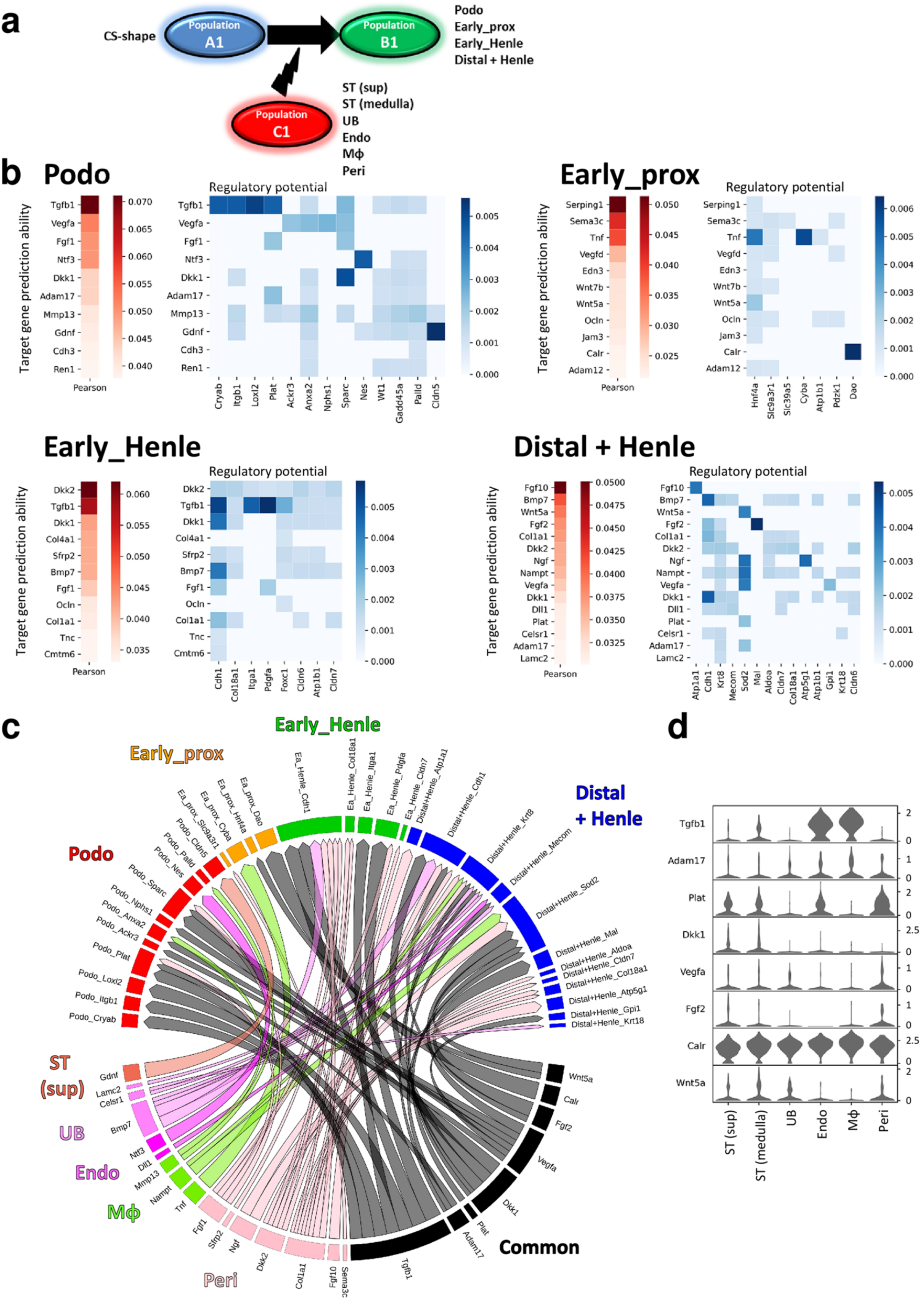
Intercellular communication in the development of each nephron segment from the comma-, and S-shaped bodies. (**a**) Intercellular communication in nephrogenesis was inferred using NicheNet. NicheNet infers intercellular communication based on ligand expression in sender cells (Population_C1), receptor expression in receiver cells (Population_A1), and intracellular signaling in the receiver cells (changes from the Population_A1 to B1). The signals from the superficial stroma (ST (sup)), medullary ST (ST (medulla)), ureteric bud (UB), endothelial (Endo), macrophage (Mφ), and pericyte (Peri) clusters that may regulate differentiation of the podocyte (Podo), early phase proximal tubule (Early_prox), early phase Henle (Early_Henle), and distal tubules and the loop of Henle (Distal + Henle) clusters from comma and S shaped bodies (CS_shape) cluster were analyzed. (**b**) Red heatmaps indicate the Pearson correlation, which represents the ability of each ligand expressed in Population_C1 to predict the changes from Population_A1 to B1. The blue heatmap denotes the regulatory potential between ligands expressed in Population_C1 and target genes expressed in Population_B1. (**c**) Results shown in (**b**) are summarized in a circos plot, in which the ligands expressed in Population_C1 were allocated to ST (sup)-, ST (medulla)-, UB-, Endo-, Mφ-, Peri-enriched, or common ligands based on the mean expression level of each ligand in each cluster. For example, bone morphogenetic protein 7 (*Bmp7)* was allocated to be an UB-enriched ligand because the mean expression level of *Bmp7* in the UB cluster was more than 1.5 times the mean *Bmp7* levels in the remaining clusters in Population_C1. The width of links represents the strength of the ligand-target regulatory potential shown in (**b**). (**d**) Stacked violin plot showing the origin of the common ligands in (**c**). All data were analyzed using R version 3.6.3 (http://www.R-project.org/) and NicheNet version 0.1.0 (https://github.com/saeyslab/nichenetr).

**Figure 7:**
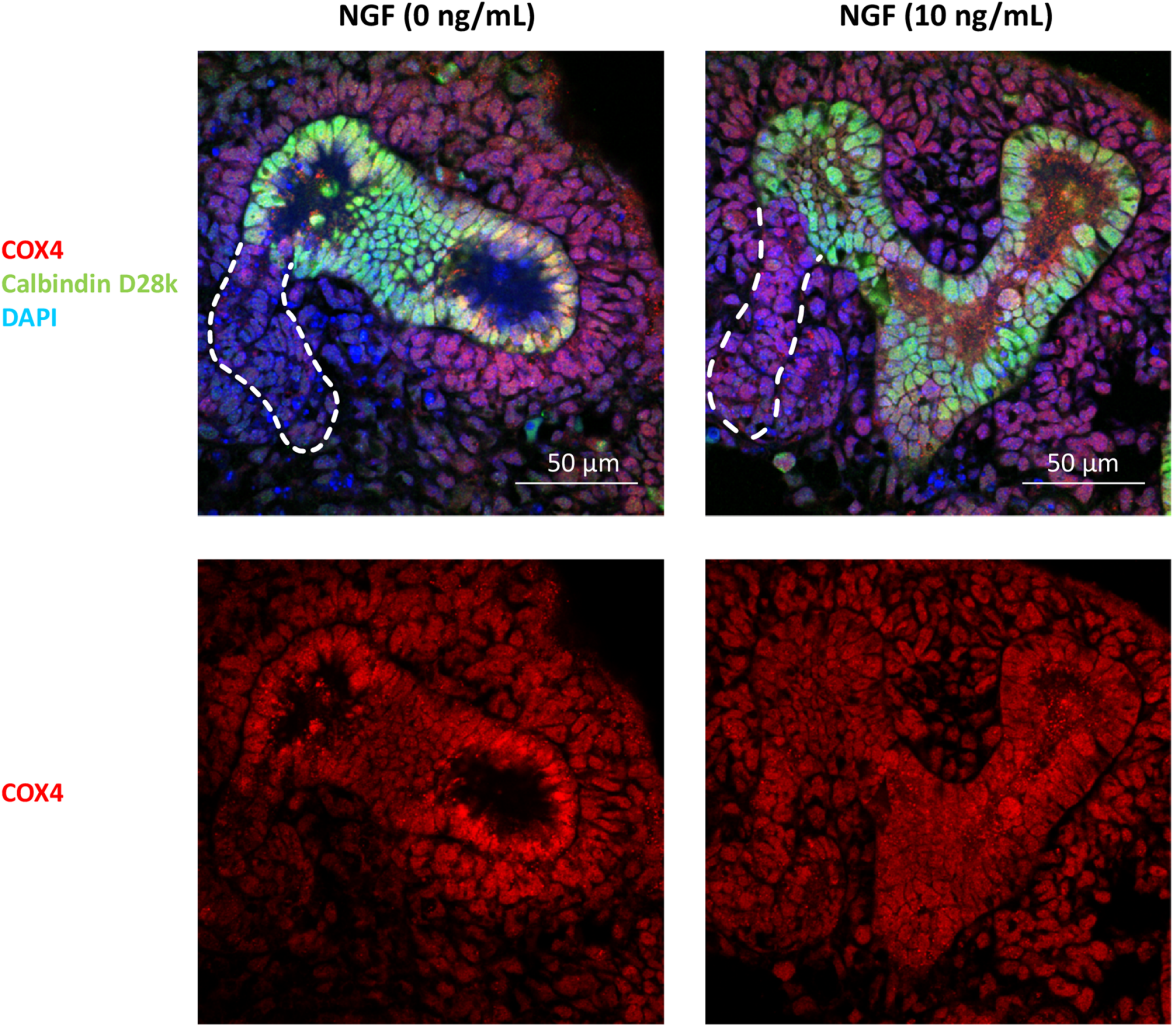
Nerve growth factor (NGF) increases mitochondrial biogenesis in embryonic distal tubules. Embryonic mouse kidneys cultured in the presence or absence of NGF (10 ng/mL) for 24 hours were analyzed. Sections were immunohistochemically stained with cytochrome c oxidase polypeptide IV (COX4), a marker of mitochondria, in red, calbindin D28k in green, and DAPI in blue (scale bar: 50 µm). Dotted white lines indicate distal tubules.

Intercellular communication in nephrogenesis was inferred using NicheNet, a recently developed method that models cell-cell communication by linking ligands to target genes^8^. In contrast to previously developed computational methods that infer intercellular communication based on ligand expression in sender cells (Population_C1 in Fig. 6a) and receptor expression in receiver cells (Population_A1 in Fig. 6a), NicheNet goes beyond ligand-receptor interactions and incorporates intracellular signaling in receiver cells as well (changes from Population_A1 to B1 in Fig. 6a)^8^. Because functional understanding of intercellular communication requires a knowledge of the way in which inferred ligand-receptor interactions result in changes in the downstream target genes in receiver cells, the ability of NicheNet to incorporate intracellular signaling of receiver cells is critically important for intercellular communication analysis^8^. As the RNA velocity demonstrated that the Podo, Early_prox, early phase loop of Henle (Early_Henle), and distal tubule and loop of Henle (Distal + Henle) clusters (hereafter, “Population_B1”) were differentiated from the CS_cluster (Population_A1), signals from the surrounding cells—the superficial stroma (ST (sup)), medullary ST (ST (medulla)), UB, endothelium (Endo), macrophage (Mφ), and pericyte (Peri) clusters (Population_C1)—that regulated differentiation of Population_B1 from Population_A1 were inferred (Fig. 6). Changes from Population_A1 to B1 are summarized in Supplementary Table S4. NicheNet inferred the Population_C1 ligands that best predicted the changes (red heat map in Fig. 6b). The Pearson correlation shown in Fig. 6b reflects the ability of each ligand to cause the changes shown in Supplementary Table S4^8^. NicheNet’s ligand-target matrices denoted the regulatory potential between ligands expressed in Population_C1, and target genes expressed in Population_B1 (blue heat map in Fig. 6b). To visualize communication among multiple cell types, the ligand-target regulatory potential shown in Fig. 6b has been summarized in a circos plot, in which the ligands expressed in Population_C1 were allocated to ST (sup)-, ST (medulla)-, UB-, Endo-, Mφ-, Peri-enriched, or common ligands (Fig. 6c). Ligands not enriched in a specific cluster were allocated to common ligands. The origins of the common ligands are shown in Fig. 6d. The circos plot showed that signals from stromal cells and ureteric buds, and also from endothelial cells, macrophages, and pericytes have the potential to contribute to the differentiation of each nephron segment (Fig. 6c). In accordance with a previous report from Magella et al., *Gdnf* was expressed not only in nephron progenitors but also in superficial stromal cells (Supplementary Fig. S3-6**e**)^11^. Stromal *Gdnf* was inferred to regulate *Cldn5* expression in podocytes (Fig. 6c). Because (1) the mitochondrion was one of the important therapeutic targets of kidney disease, (2) several target genes for the Distal + Henle cluster were mitochondrial genes, and (3) little was known about the nephrogenesis-related roles of *Ngf*, one of the ligands that was inferred to regulate the differentiation of the Distal + Henle cluster, the effects of NGF were analyzed. Organ culture of mouse embryonic kidneys revealed that NGF increased mitochondrial biogenesis in the distal tubules, while not affecting the gross morphology of the developing kidney (Fig. 7).

**Figure 8:**
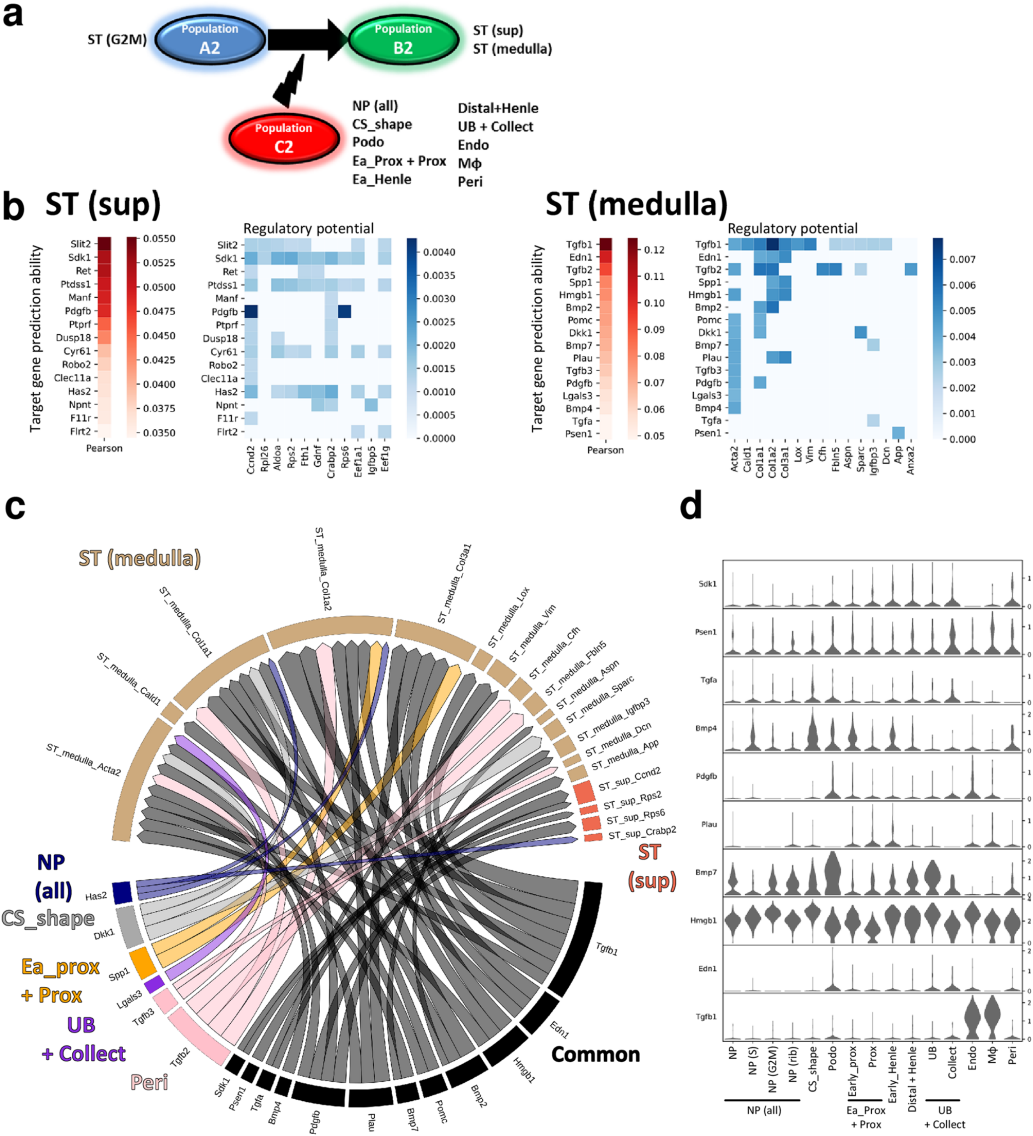
Intercellular communication in the development of stromal cells. (**a**) Intercellular communication in the development of stromal cells was inferred using NicheNet. The signals from the nephron progenitor (NP), S-phase NP (NP (S)), G2M-phase NP (NP (G2M)), ribosome-enriched NP (NP (ribo)), comma and S shaped bodies (CS_shape), podocyte (Podo), early phase proximal tubule (Early_prox), proximal tubule (Prox), early phase Henle (Early_Henle), distal tubules and the loop of Henle (Distal + Henle), ureteric bud (UB), collecting duct (Collect), endothelial (Endo), macrophage (Mφ), and pericyte (Peri) clusters that induced the superficial stroma (ST (sup)) and medullary ST (ST (medulla)) clusters from the G2M-phase ST (ST (G2M)) cluster were analyzed. Because many clusters were included in Population_C2, the NP, NP (ribo), NP (S), and NP (G2M) clusters were processed as one group, which was named NP (all). In a similar manner, the Early_prox and the Prox clusters were processed as one group, which was named Early_prox + Prox. The UB cluster and the Collect clusters were also processed as one group, which was named UB + Collect. (**b**) Red heatmaps indicate the Pearson correlation. The blue heatmap denotes the regulatory potential between ligands expressed in Population_C2 and target genes expressed in Population_B2. (**c**) Results shown in (**b**) are summarized in a circos plot. (**d**) Stacked violin plot showing the origin of the common ligands in Fig. 8c. All data were analyzed using R version 3.6.3 (http://www.R-project.org/) and NicheNet version 0.1.0 (https://github.com/saeyslab/nichenetr).

Finally, signals from the clusters in Population_C2 that induced the ST (sup) and ST (medulla) clusters (Population_B2) from the G2M-phase ST (ST (G2M)) cluster (Population_A2) were analyzed (Fig. 8a). Changes from Population_A2 to B2 were defined in a similar manner to those in Fig. 6 (Supplementary Table S5). NicheNet inferred the ligands that best predicted the changes from Population_A2 to B2 (Fig. 8b). Because platelet derived growth factor subunit B (*Pdgfb)*, a key inducer of mesangial cells in combination with platelet derived growth factor receptor beta *(Pdgfrb)*, was inferred as a ligand for gene induction in both ST (sup) and ST (medulla) clusters, *Pdgfrb* was visualized (Supplementary Fig. S9a). *Pdgfrb*-expressing cells were mainly mapped in the ST (medulla) cluster (Supplementary Fig. S9a)^29^. A circos plot indicated that various cell types have the potential to contribute to the differentiation of stromal cells (Fig. 8c). Violin plots of the common ligands revealed that *Pdgfb* was relatively enriched in the Podo and the Endo clusters, in accordance with the anatomical topology of mesangial cells (Fig. 8d). As many of the ligands for ST (medulla) were predicted to induce collagen type 1 alpha 1 (*Col1a1)* and collagen type 1 alpha 2 (*Col1a2)*, the expression of these genes was also visualized (Supplementary Fig. S9b). Immunofluorescent staining demonstrated that collagen I surrounded tubules and vessels, indicating that medullary stromal cells adjacent to tubules and vessels were the main cells that receive signals for the induction of *Col1a1* and *Col1a2* (Supplementary Fig. S9c). In contrast to injured adult kidney, in which transforming growth factor beta 1 (*TGFB1)* has been reported to be expressed mainly in tubular cells, the origins of *Tgfb1* in the developing kidney are the cells belonging to the Endo and the Mφ clusters (Fig. 8d)^30^. Because TGF-β1 was the key molecule that induced genes expressed in the ST (medulla) cluster, endothelial cells and macrophages might be also important contributors to the development of stromal cells.

## Discussion

In this study, scRNA-seq data of embryonic mouse kidney deposited by Combes et al*.* was reanalyzed^6^. Although Combes et al. intensively analyzed the scRNA-seq data, rapid developments in analytical approaches for scRNA-seq have enabled the extraction of much more information from the same data. Trajectory analysis was performed by Combes et al., generating valuable information. However, the tool used cannot infer trajectories of the disconnected type^2^. In the current study, the RNA velocity was investigated, an analysis which was not possible using the tools available to Combes et al.^6^ Intercellular communication was inferred using NicheNet, an approach that takes into account intracellular signaling in receiver cells.

Several modes of intercellular communication were inferred using the NicheNet analyses. First, *Tgfb1* was listed as a ligand to regulate characteristic genes for the Podo, Early_Henle, and ST (medulla) clusters. However, the target genes of *Tgfb1* were distinct among these three clusters. *Tgfb1* was inferred to regulate the characteristic genes for myofibroblasts in the ST (medulla) cluster, while these genes were not the targets of *Tgfb1* in the Podo and Early_Henle clusters. *Tgfb1* was inferred to regulate alpha crystalline B (*Cryab)*, a stabilizer of the cytoskeleton, integrin beta 1 (*Itgb1)*, a linker for the cytoskeleton to the extracellular matrix, and lysyl oxidase like 2 (*Loxl2)*, a crosslinker of the extracellular matrix. *Tgfb1* may therefore contribute to podocytes becoming resistant to mechanical stress caused by urinary flow^31–33^. In addition to *Tgfb1*, which is expressed mainly in macrophages and endothelial cells, neurotrophin 3 *(Ntf3)*, whose expression was enriched in endothelial cells, was inferred to regulate nestin (*Nes)*, a gene encoding the intermediate filaments of podocytes^34^. Therefore, cell-cell communication between podocytes and endothelial cells might be critically important for the proper cytoskeletal arrangement of podocytes. Second, several Wnt signaling molecules were inferred to regulate differentiation of the Podo, Early_prox, Early_Henle, and Distal + Henle clusters. Schneider et al. have reported that nephron progenitor cells exposed to high levels of Wnt form tubular components, while cells exposed to low levels of Wnt form glomerular elements^35^. Using β-catenin signaling reporter mice (T-cell factor/lymphoid enhancer factor :: histone 2B green fluorescent protein (TCF/Lef::H2B-GFP)), Lindström et al. found that TCF/Lef::H2B-GFP signaling intensities increased exponentially along the proximal, medial, and distal segments of developing nephrons^36^. Low et al. demonstrated that activation of the Wnt signaling pathway by CHIR99021, a potent and selective glycogen synthase kinase 3 inhibitor, decreases the glomerulus-to-tubule ratio and proximal-to-distal-tubular ratio in three-dimensional kidney organoids developed from human pluripotent stem cells^37^. Low et al. also showed that kidney organoids with a high glomerulus-to-tubule ratio possessed a richer vascular network than those with a low glomerulus-to-tubule ratio because of the correlative vascular endothelial growth factor A production by podocytes^37^. In accordance with these reports, NicheNet listed dickkopf WNT signaling pathway inhibitor 1 (*Dkk1)* as a regulator of podocyte differentiation. However, NicheNet also suggested that the process of Wnt signaling inducing tubular segments is not as simple as reported by Schneider et al.^35^ Wnt stimulators were inferred to be inducers for the Early_prox cluster, while the Wnt inhibitors, *Dkk1*, *Dkk2*, and *Sfrp2*, were shown to be regulators of the Early_Henle cluster. Both Wnt stimulators and inhibitors were identified as differentiational regulators for the Distal + Henle cluster. Because Wnt proteins mainly act in an autocrine and paracrine, but not in an endocrine manner, finely regulated local Wnt signals may be required for the development of each nephron segment^38^. Third, the circos plots suggested that the differentiation of both nephron and stromal cells required coordinated intercellular communication. Because collagen I acts as a structural matrix and provides organ morphology, appropriate signals for stromal cell development may be important for nephrogenesis.

Recently, Adam et al. showed that psychrophilic proteases reduce scRNA-seq artifacts^39^. However, most of the scRNA-seq data, including the data analyzed in this study, have been obtained by incubating tissues in enzymatic solution at 37 °C. Because scRNA-seq is a rapidly developing technology, continual refinements both in experimental and in analytical methods are required to maximize the utility of scRNA-seq.

In summary, we inferred the developmental process of the kidney. PAGA-initiated ForceAtlas2 coordinated cells in a way that reflected expected lineage relationships. Consequently, PAGA-initiated ForceAtlas2 enabled the identification sub-clusters that may comprise parietal epithelial cells of the Bowman’s capsule, and cells in the developing process from the S-shaped body to the proximal tubules or to the loop of Henle and distal tubules. Analysis of RNA velocity suggested the developmental sequences of proximal tubules and podocytes. In combination with a Markov chain algorithm, RNA velocity suggested the self-renewal process of the nephron progenitors. NicheNet inferred sophisticated cell-cell interactions, while organ culture of embryonic mouse kidneys revealed that NGF contributed to mitochondrial biogenesis in the distal tubules. Our approach suggested previously unrecognized aspects of the underlying mechanisms of kidney development.

## Methods

### Pre-processing of scRNA-seq data

Biologically replicated (n=3) mouse kidney scRNA-seq data at embryonic day 18.5 were obtained from the NCBI-SRA (GSE108291)^6^. SRA files were converted into FASTQ files using the SRA Toolkit (https://github.com/ncbi/sra-tools). Sequence alignment, filtering, barcode counting, and unique molecular identifier counting were performed using the Cell Ranger software version 2.2 count pipeline (10x GENOMICS, Pleasanton, CA). The Cell Ranger software count pipeline yielded an RNA count matrix, which contained 6,744 cells × 31,053 genes. Spliced and unspliced RNA count matrices were obtained by Velocyto^13^. The proportions of spliced, unspliced, and ambiguous sequences were 0.80, 0.16, and 0.04, respectively. During pre-processing, 858 cells that had less than 200 genes expressed and 13,071 genes that were detected in fewer than three cells were filtered out. Cells with unique feature counts over 5,800/cell or less than 1,000/cell, RNA counts over 35,000/cell, or a mitochondrial count of over 10% were also filtered out (Supplementary Fig. S1a). The resultant RNA count matrix contained 5,502 cells × 17,982 genes. Highly variable genes (3,000) were selected for analysis (Supplementary Fig. S1b). To assess batch effects, we visualized cells by reducing dimensionalities with uniform manifold approximation and projection (UMAP) (Supplementary Fig. S1c). Although cells from each batch were distributed similarly, there were slight differences across different batches. Therefore, the batch-effect was corrected by batch balanced *k*-nearest neighbors (BBKNN) (Supplementary Fig. S1c)^40^. The distribution of each batch after correction was also visualized using PAGA-initialized ForceAtlas2 (Supplementary Fig. S1c).

scRNA-seq data of human kidney organoid, mouse adult kidney, and human kidney at embryonic week 18 were obtained from NCBI-SRA (GSE114802, GSE107585, and GSE114530), and pre-processed in a similar manner. Because the SRA file of GSM2796989, a paired-end read scRNA-seq data of embryonic mouse kidney at day 14.5, yielded a single fastq file using the fastq-dump command with –split-files option, we analyzed the archived matrix file for the analysis of GSM2796989.

### Analyses of scRNA-seq data

The cluster annotation shown in Fig. 1 was manually performed, based on the differentially expressed features shown in Supplementary Figs. S3 and S4. Cell cycle scores were assigned based on cell cycle genes defined by Tirlsh et al.^41^ RNA velocity was calculated using scVelo^31^. Heatmap, violin plots, and ForceAtlas2 map were drawn using Scanpy^42,43^. The root of RNA velocity in nephron progenitors was inferred using CellRank (https://cellrank.readthedocs.io/en/stable/#). Intercellular communication was evaluated using NicheNet^8^.

### Animals

C57BL/6 mice at E18.5 were purchased from Japan SLC (Hamamatsu, Japan) (n=6). Mice were processed using procedures previously described^44–46^. All animal experiments were approved by the Animal Committee of Osaka University (approval number 280068-021), and the study was performed in accordance with ARRIVE guidelines (Animal Research: Reporting of *In Vivo* Experiments) and with AVMA (American Veterinary Medical Association) guidelines.

### Organ culture of embryonic mouse kidneys

Kidneys were obtained from C57BL/6 mice at embryonic day 12.5. Isolated kidneys were placed in Transwells with 3.0 µm pores (Corning, NY, USA). Kidneys were cultured in Dulbecco’s modified Eagle medium (low glucose, 1.0 g/L, Nakarai Tesque, Kyoto, Japan) supplemented with 10% fetal calf serum (Sigma-Aldrich, St. Louis, MO, USA), 100 U/mL penicillin, and 100 µg/mL streptomycin (Giibco, MA, USA), in the presence or absence of 10 ng/mL native mouse nerve growth factor (NGF) 2.5S (Almone Labs, Jerusalem, Israel) for 24 h in a humidified 37 °C incubator with 5% CO_2_ (n=6 in each group).

### Histological analyses

Antibodies against the following molecules were used: myoshin-11 (ab53219, Abcam, Cambridge, UK), α-smooth muscle actin (A2547, Sigma-Aldrich), claudin 1 (51-9000, Thermo Fisher Scientific, MA, USA), cytochrome c oxidase polypeptide IV (COX4) (PM063, Medical & Biological Laboratories, Nagoya, Japan), collagen I (ab34710, Abcam), and calbindin D28k (214004, Synaptic Systems, Goettingen, Germany). For the detection of myosin-11, α-smooth muscle actin, claudin 1, COX4, collagen I, and calbindin D28k staining, paraffin-embedded embryonic mice kidney sections were immunologically stained as described previously^46–51^. The images shown in Figs. 2c and 7 were obtained using an LSM 880 with an Airyscan microscope (Zeiss, Oberkochen, Germany).

### Statistical analysis

All RNA-sequencing data were analyzed using python or R (http://www.R-project.org/)^52^. Normalization, logarithmic transformation, identification of highly variable features, scaling, principal component analysis, extraction of differentially expressed features, and PAGA-initialized manifold learning were performed using Scanpy^43^. NicheNet analyses were performed in R.

## Supplementary Information

Supplementary Information 1.

Supplementary Information 2.

Supplementary Information 3.

Supplementary Information 4.

Supplementary Information 5.

Supplementary Information 6.

## Data Availability

The datasets generated and/or analyzed during the current study are available from the corresponding author on reasonable request. SRA files are available at NCBI Sequence Read Archive (GSE108291, GSM2796989, GSE114802, GSE107585, and GSE114530).
