# Supplementary material for: Single cell RNA sequencing uncovers cellular developmental sequences and novel potential intercellular communications in embryonic kidney
Source: Sci Rep. 2021 Jan 8;11:73. doi: 10.1038/s41598-020-80154-y (PMC7794461; doi:10.1038/s41598-020-80154-y)
Supplement: Supplementary file 1 — Supplementary Information 1. [file 41598_2020_80154_MOESM1_ESM.pdf]

Single cell RNA sequencing uncovers cellular developmental sequences and novel potential intercellular communications in embryonic kidney

Isao Matsui, Ayumi Matsumoto, Kazunori Inoue, Yusuke Katsuma, Seiichi Yasuda, Karin Shimada, Yusuke Sakaguchi, Masayuki Mizui, Jun-ya Kaimori, Yoshitsugu Takabatake, and Yoshitaka Isaka

Supplementary Material

Supplementary Table S1-1.
Differentially expressed features in each cluster (mouse embryonic kidney at day 18.5).

Supplementary Table S1-2.
Differentially expressed features in each cluster (mouse adult kidney).

Supplementary Table S1-3.
Differentially expressed features in each cluster (mouse kidney at embryonic day 14.5).

Supplementary Table S1-4.
Differentially expressed features in each cluster (human kidney organoid).

Supplementary Table S2.
Differentially expressed features in each cluster (human embryonic kidney).

Supplementary Table S3-1.
Features of the root and the non-root cells in nephron progenitors.

Supplementary Table S3-2.
Differentially expressed features between the sub-cluster NP3 and NP4.

Supplementary Table S4.

Features that define changes from the Population_A1 to the B1 in Fig. 6a.

Supplementary Table S5.
Features that define changes from the Population_A2 to the B2 in Fig. 8a.

Supplementary Figure S1.
Pre-processing of single cell RNA sequencing data obtained from mouse kidney at embryonic day 18.5.

Supplementary Figure S2.
PAGA of adult mouse kidney, embryonic mouse kidney at day 14.5, and human kidney organoid.

Supplementary Figure S3-1.
Expression patterns of marker genes for podocytes, early phase proximal tubules, and proximal tubules.

Supplementary Figure S3-2.
Expression patterns of marker genes for early phase Henle, the loop of Henle, and distal tubules.

Supplementary Figure S3-3.
Expression patterns of marker genes for the ureteric bud and collecting duct.

Supplementary Figure S3-4.
Expression patterns of marker genes for nephron progenitors, comma-, and S-shaped bodies.

Supplementary Figure S3-5.
Expression patterns of marker genes for macrophages, immune cells, and endothelial cells.

Supplementary Figure S3-6.
Expression patterns of marker genes for stromal cells.

Supplementary Figure S3-7.
Expression patterns of marker genes for pericytes.

Supplementary Figure S4.
Cluster characterization.

Supplementary Figure S5-1 to 5-94.
Expression patterns of highly variable genes.

Supplementary Figure S6.
Expression patterns of segment specific marker genes for proximal tubules.

Supplementary Figure S7.
Analyses of scRNA-seq data obtained from human embryonic kidney.

Supplementary Figure S8.
RNA velocity of genes which were highly expressed at the tip of podocyte clusters in human embryonic kidney data.

Supplementary Figure S9.
Expression patterns of platelet derived growth factor receptor beta *(Pdgfrb)*, collagen type 1 alpha 1 (*Col1a1),* and collagen type 1 alpha 2 (*Col1a2*).

Supplementary References

Supplementary Table S1-1.
Differentially expressed features in each cluster
(mouse embryonic kidney at day 18.5).

Rank	NP	NP (S)	NP (G2M)	NP (ribo)	CS shape	Podo	Early prox	Prox	Early Henle	Distal + Henle
1	*Cited1*	*C1qtnf12*	*Stmn1*	*Cited1*	*Sfrp2*	*Wt1*	*Phgdh*	*Ttc36*	*Pdgfa*	*Wfdc2*
2	*Gas1*	*Rpl7*	*Pclaf*	*Rps23*	*Lhx1*	*Igfbp7*	*Cdh6*	*Aldob*	*Irx1*	*Mal*
3	*Eef2*	*Wt1*	*Hmgb2*	*Rps27a*	*Pax8*	*Cldn5*	*Rida*	*Fxyd2*	*Epcam*	*Wfdc15b*
4	*Fxyd6*	*Pax2*	*Ptma*	*Rpl32*	*Lhx1os*	*Eif3m*	*Npnt*	*Fbp1*	*Spint2*	*Sostdc1*
5	*Eef1a1*	*Rps2*	*H2afz*	*Fxyd6*	*Sox11*	*Mafb*	*Calml4*	*Ass1*	*Fstl1*	*Epcam*
6	*Rpl3*	*Rplp0*	*Tubb5*	*Rps5*	*Stmn1*	*Gadd45a*	*Cpn1*	*Calml4*	*Irx2*	*Chchd10*
7	*Rps4x*	*Wnt4*	*Cks1b*	*Ttc28*	*Emid1*	*Rasl11a*	*Slc9a3r1*	*Dbi*	*Pou3f3*	*Atp1b1*
8	*Rpl23*	*Rpl18a*	*Cenpw*	*Crym*	*Ccnd1*	*Srgap1*	*Fxyd2*	*Pdzk1ip1*	*Cldn6*	*Spint2*
9	*Rplp0*	*Rpl14*	*Tuba1b*	*Spock2*	*Hmgb3*	*Plat*	*Ddah1*	*Rida*	*Umod*	*Tmem52b*
10	*Crym*	*Rpl32*	*H2afx*	*Rps24*	*Ptma*	*Dpp4*	*Hnf4a*	*Gsta2*	*Atp1b1*	*Atp1a1*
11	*Rps5*	*Rpsa*	*Uncx*	*Rps20*	*Ran*	*Efnb1*	*Folr1*	*Pcbd1*	*Tspan8*	*Ldhb*
12	*Rps9*	*Rpl12*	*H2afv*	*Rpl23*	*Ranbp1*	*1810037I17Rik*	*Spink1*	*Fabp3*	*D030045P18Rik*	*Tfcp2l1*
13	*Rpl32*	*Rps27a*	*Dut*	*Vcan*	*Hmgn1*	*Hist3h2ba*	*Slc39a5*	*Gatm*	*Epha4*	*Ly6a*
14	*Rps27a*	*Rps24*	*Top2a*	*Rps15a*	*Cdk4*	*Foxc2*	*Mpc2*	*Cbr1*	*Sim2*	*Cldn7*
15	*Tsc22d1*	*Npm1*	*Gas1*	*Rps13*	*Clu*	*Golim4*	*Aldob*	*Sult1d1*	*Ccnd1*	*Nudt4*
16	*Rps24*	*Cxxc5*	*Hmgb3*	*Rps4x*	*Eva1a*	*Tsc22d1*	*Fut9*	*Atpif1*	*Prss23*	*Fstl1*
17	*Spock2*	*Rpl11*	*Spock2*	*Gas1*	*Uncx*	*Cryab*	*Clu*	*Spp2*	*Rab3ip*	*Mgst1*
18	*Hsp90ab1*	*Bmper*	*Rps4x*	*Rps18*	*Hnrnpab*	*Zbtb7c*	*Dcdc2a*	*Chchd10*	*Hoxb8*	*Tmem213*
19	*Rps18*	*Rps17*	*Cdca3*	*Rplp2*	*Hey1*	*Podxl*	*Cldn6*	*Pdzk1*	*Cldn7*	*Slc25a5*
20	*Rps14*	*Rps5*	*Cenpf*	*Rspo1*	*Pclaf*	*Bcam*	*Lrpap1*	*Idh1*	*Smagp*	*Cdh1*
21	*Rpl10a*	*Rpl13a*	*Rps24*	*Rpl10a*	*Hmgb2*	*Mmp23*	*Kif12*	*Cystm1*	*Wfdc2*	*Hoxd8*
22	*Rpl23a*	*Rps15*	*Fxyd6*	*Rpl26*	*Jag1*	*Ackr3*	*Pdzk1*	*Spink1*	*Hipk2*	*Mpc2*
23	*Rpl4*	*Rpl17*	*Rplp0*	*Uncx*	*Snrpe*	*Csnk1a1*	*Tmem27*	*Ggt1*	*Pantr1*	*Slc25a3*
24	*Npm1*	*Rpl23a*	*Birc5*	*Rps14*	*Nme1*	*R3hdml*	*Akr7a5*	*Gpx3*	*Foxc1*	*Tfap2b*
25	*Rpl18a*	*Prdx6*	*Spc25*	*Rpl18a*	*Tubb5*	*Cdkn1c*	*Pcbd1*	*Atp5g1*	*Mpc2*	*Neat1*
26	*Tgfbi*	*Tmem100*	*Wt1*	*Rplp0*	*Pcp4*	*Arhgap24*	*Rab3ip*	*Gas2*	*Hoxb7*	*Rab3ip*
27	*Rpl26*	*Rpl10a*	*Ezh2*	*Rps9*	*H2afz*	*Nes*	*Ly6a*	*Tmem27*	*Chchd10*	*Mecom*
28	*Rpl9*	*Gxylt2*	*Anp32b*	*Rpl23a*	*H2afv*	*Calm2*	*Atp1b1*	*Acot13*	*Crb3*	*Cldn6*
29	*Rpl12*	*Mycn*	*Hmgn1*	*Rps16*	*H2afy*	*H3f3a*	*Adamts1*	*Cox5a*	*Spon1*	*Atp5b*
30	*Fam213a*	*Rpl3*	*Ccna2*	*Eya1*	*Ybx1*	*Rassf10*	*Cyba*	*Uqcr10*	*Ybx1*	*Aldoa*

Rank	UB	Collect	ST (G2M)	ST (sup)	ST (medulla)	ST (ureter)	Peri	Endo	Mφ	Immune
1	*Rprm*	*Foxq1*	*Hmgb2*	*Igfbp5*	*Cald1*	*Penk*	*Col1a1*	*Fabp4*	*Ctsb*	*Rac2*
2	*Calb1*	*Krt19*	*H2afx*	*Mgp*	*Fbln5*	*Tmsb4x*	*Col1a2*	*Plvap*	*C1qc*	*Arhgdib*
3	*Epcam*	*Krt8*	*Birc5*	*Ptn*	*Ace2*	*Tcf21*	*Dlk1*	*Emcn*	*Selenop*	*Sh3bgrl3*
4	*Cldn3*	*Krt18*	*Cks1b*	*Fibin*	*Gpc3*	*Csrp1*	*Col3a1*	*Crip2*	*B2m*	*Coro1a*
5	*Gata3*	*S100a6*	*Smc2*	*Hic1*	*Igfbp3*	*Lgals1*	*Sparc*	*Gng11*	*C1qb*	*Tyrobp*
6	*Wfdc2*	*Gata3*	*H2afz*	*Ccnd2*	*Sept7*	*Col1a2*	*Dcn*	*S100a16*	*Fcer1g*	*Fcer1g*
7	*Krt18*	*Spint2*	*Pclaf*	*Mest*	*Ptn*	*Col3a1*	*Meg3*	*Cdh5*	*Ms4a6c*	*Lgals3*
8	*Wfdc15b*	*Plet1*	*Lmnb1*	*Alcam*	*Col1a2*	*Cfh*	*Col6a1*	*Gimap4*	*C1qa*	*B2m*
9	*Hoxb7*	*Cystm1*	*Ccna2*	*Meis1*	*Col3a1*	*Sdc2*	*Col5a2*	*Icam2*	*Lgmn*	*H2-D1*
10	*Mgst1*	*Fxyd3*	*Tuba1b*	*Mfap2*	*Myl9*	*Cnn2*	*Col6a2*	*Gimap6*	*Pf4*	*Tmsb4x*
11	*Crlf1*	*Tmprss2*	*Hmgb1*	*Ifitm1*	*Tcf21*	*Cdkn1c*	*Lgals1*	*Cd34*	*Csf1r*	*Gpx1*
12	*Hoxb8*	*Klf5*	*Ptn*	*Plagl1*	*Fhl2*	*Sfrp1*	*Cavin3*	*Fam167b*	*Tyrobp*	*Alox5ap*
13	*Gfra1*	*Cldn4*	*Cks2*	*Maged2*	*Cnn2*	*Myl9*	*S100a6*	*Esam*	*Grn*	*Cd53*
14	*Mal2*	*Krt7*	*H2afv*	*Fhl2*	*Cfh*	*Col15a1*	*Cdkn1c*	*Fkbp1a*	*Ctsc*	*Arpc1b*
15	*Lcn2*	*Hspb1*	*Mki67*	*Eva1b*	*Lbh*	*Lhfp*	*Gsn*	*Ifitm3*	*Fcgr3*	*Laptm5*
16	*Bex4*	*S100a11*	*Stmn1*	*Meis2*	*Col1a1*	*Lbh*	*Itm2a*	*Pecam1*	*Ly86*	*Srgn*
17	*Fam162a*	*Cdh1*	*Cdca8*	*Lhfp*	*Plac8*	*Col1a1*	*Vim*	*Egfl7*	*Ctss*	*Actr3*
18	*Spint2*	*Rab25*	*Top2a*	*Mdk*	*Eva1b*	*Acta2*	*Igf1*	*Bst2*	*Cst3*	*Pfn1*
19	*Cldn7*	*Tacstd2*	*Tmpo*	*Aldh1a2*	*Pdlim2*	*Fn1*	*Ogn*	*Plpp1*	*Aif1*	*Ptpn18*
20	*Bex1*	*Rnf128*	*Tpx2*	*Mfap4*	*Ifitm1*	*Gucy1a1*	*Col5a1*	*Fabp5*	*Laptm5*	*Ly6e*
21	*Fam149a*	*Ptprf*	*Smc4*	*H19*	*Gucy1a1*	*Rarres2*	*Rarres2*	*Cd93*	*H2-D1*	*Psmb8*
22	*Hoxb9*	*Mal2*	*Cdca3*	*Col1a1*	*Col6a1*	*Mylk*	*H19*	*S100a10*	*Ctsz*	*Arpc2*
23	*Mia*	*Atox1*	*Cdk1*	*Sub1*	*Acta2*	*Olfml3*	*Nenf*	*Slc9a3r2*	*Lyz2*	*Tspo*
24	*Tpd52l1*	*Mal*	*Spc24*	*Rgs5*	*Tpm4*	*Vim*	*Col14a1*	*B2m*	*Unc93b1*	*Ostf1*
25	*Sox9*	*Tspan8*	*Tubb6*	*Selenom*	*G0s2*	*Selenom*	*Pcolce*	*Elk3*	*Fcrls*	*Taldo1*
26	*Tesc*	*Cd9*	*Fhl2*	*Ebf1*	*Emilin1*	*Nkd1*	*Nfib*	*Cldn5*	*Lst1*	*Cyba*
27	*Cldn8*	*Mgst1*	*Lhfp*	*Ifitm3*	*Cdh11*	*Thy1*	*Fbln2*	*Ecscr*	*Ftl1*	*Plac8*
28	*Cd24a*	*Vamp8*	*Spc25*	*Pdlim2*	*Anxa2*	*Ccnd2*	*Mfap5*	*Rsad2*	*Ms4a6b*	*Npc2*
29	*Ldhb*	*Cldn7*	*Racgap1*	*Gdnf*	*S100a6*	*Akap12*	*Postn*	*Myct1*	*Cd68*	*Slfn2*
30	*Rab25*	*Lmo4*	*Cenpa*	*Il11ra1*	*Lgals1*	*Plac8*	*Pmp22*	*Calm1*	*Lamp1*	*Psap*

Differentially expressed features in each cluster are shown. Features were extracted using Scanpy version 1.4.4.post1 (https://scanpy.readthedocs.io/en/stable/) (Wilcoxon

rank-sum test). Abbreviations: NP, nephron progenitor; NP (S), S-phase nephron progenitor; NP (G2M), G2M-phase nephron progenitor; NP (ribo), ribosome-enriched nephron progenitor; CS_shape, comma and S shaped bodies; Podo, podocyte; Early_prox, early phase proximal tubule; Prox, proximal tubule; Early_Henle, early phase Henle; Distal + Henle, distal tubule and the loop of Henle; UB, ureteric bud; Collect, collecting duct; ST (G2M), G2M-phase stroma; ST (sup), superficial stroma; ST (medulla), medullary stroma; ST (ureter), ureter-associated stroma; Peri, pericyte; Endo, endothelium; Mφ, macrophage; Immune, immune cell.

Supplementary Table S1-2.
Differentially expressed features in each cluster (mouse adult kidney).

Rank	Cluster 0	Cluster 1	Cluster 2	Cluster 3	Cluster 4	Cluster 5	Cluster 6	Cluster 7	Cluster 8
1	*Gpx3*	*Zbtb20*	*Kap*	*Cyb5a*	*Slc12a1*	*Ftl1*	*Tmsb10*	*Slc12a3*	*Gpx3*
2	*Snhg11*	*Slc22a30*	*mt-Cytb*	*Gpx1*	*Ppp1r1a*	*Fxyd2*	*Tmsb4x*	*Wnk1*	*mt-Nd1*
3	*Malat1*	*Slc27a2*	*Ttc36*	*Ttc36*	*Mt1*	*Gatm*	*Cd52*	*Defb1*	*mt-Atp6*
4	*Slc5a12*	*Slc34a1*	*mt-Atp6*	*Dbi*	*Umod*	*Aldob*	*Rps27*	*Tmem52b*	*mt-Co3*
5	*Maf*	*Mat2a*	*mt-Nd1*	*Rida*	*Egf*	*Fth1*	*Rps24*	*Atp1a1*	*mt-Nd4*
6	*Slc34a1*	*Cyp4b1*	*Cndp2*	*Fth1*	*Wfdc15b*	*Cela1*	*Rac2*	*Egf*	*mt-Cytb*
7	*Gatm*	*Gm45792*	*Acy3*	*Aldob*	*Sostdc1*	*Ass1*	*Pfn1*	*Ppp1r1a*	*Ftl1*
8	*Slc6a19*	*Ces1f*	*Gpx1*	*Akr1a1*	*Atp1a1*	*2010107E04Rik*	*Rps16*	*Mt1*	*Gatm*
9	*Slc5a2*	*Chpt1*	*mt-Nd4*	*Akr1c21*	*Ly6a*	*Mif*	*B2m*	*Tmem213*	*mt-Nd2*
10	*Fut9*	*Malat1*	*Dbi*	*Fxyd2*	*Mt2*	*Ftl1-ps1*	*Rplp2*	*Wfdc15b*	*mt-Co2*
11	*Tnfaip8*	*Pck1*	*mt-Co2*	*Prdx5*	*Atp1b1*	*Gpx1*	*H2-D1*	*Pvalb*	*Spink1*
12	*Slc4a4*	*Acsm2*	*Cyb5a*	*Ass1*	*Kng2*	*Atp6v1g1*	*Rpl18a*	*Kng2*	*Aldob*
13	*Neat1*	*Atp11a*	*Cyp4b1*	*Inmt*	*Wfdc2*	*Uqcr10*	*Rps15a*	*Atp1b1*	*mt-Co1*
14	*Nox4*	*Slc22a28*	*mt-Co3*	*Kap*	*Klk1*	*Gpx3*	*Rps11*	*Umod*	*Fth1*
15	*Timp3*	*Kap*	*Slc27a2*	*Ftl1*	*Nudt4*	*Rpl41*	*AW112010*	*Slc16a7*	*Ass1*
16	*Spp2*	*Slc17a3*	*Selenop*	*Prdx1*	*Spp1*	*Rps2*	*Rpl12*	*Cox6c*	*Spp2*
17	*Ftl1*	*Polr3e*	*Prdx5*	*Cndp2*	*Mrps6*	*Hint1*	*Rpl19*	*Klhl3*	*Mif*
18	*Miox*	*Timp3*	*Chpt1*	*Atpif1*	*Mal*	*Akr1a1*	*Rps13*	*Wnk4*	*Cela1*
19	*Car12*	*Nat8f6*	*Spink1*	*Gpx4*	*Sfrp1*	*Atp5e*	*Rpl17*	*Sfrp1*	*Lgmn*
20	*Lgmn*	*Mlxipl*	*mt-Co1*	*Acy3*	*Slc5a3*	*Pcbd1*	*Actb*	*Gabarapl1*	*Fxyd2*
21	*Dab2*	*Ddx5*	*Guca2b*	*AI314278*	*Tmem213*	*Rpl28*	*Rps4x*	*Calb1*	*Gpx1*
22	*Zbtb20*	*Lrp2*	*Fth1*	*Chchd2*	*Cd24a*	*Atox1*	*Rps5*	*Pgam2*	*Atox1*
23	*Spink1*	*Slc47a1*	*Acsm2*	*Scp2*	*Gm47708*	*Chchd2*	*Rps23*	*Mal*	*mt-Nd3*
24	*Ank3*	*Slc22a6*	*Tmem27*	*Khk*	*Ckb*	*Ndufc1*	*Fau*	*Kcnj1*	*Pdzk1ip1*
25	*Ndrg1*	*Pde4d*	*Inmt*	*Miox*	*Fabp3*	*Atp5l*	*Rps3*	*Lhx1*	*Hint1*
26	*Polr3e*	*Cyp2e1*	*Napsa*	*Hspe1*	*Ldhb*	*Cox6c*	*Rpl13*	*Sgms2*	*Tmem27*
27	*Snx29*	*Neat1*	*Aldob*	*Txn1*	*1700011H14Rik*	*Atp5g1*	*Rpsa*	*Trpm7*	*mt-Nd5*
28	*Slc22a28*	*Kcnj15*	*Gpx4*	*Chpt1*	*Clcnkb*	*Rida*	*Rps7*	*Ndufa1*	*Uqcr10*
29	*Errfi1*	*Acaa1b*	*Mep1a*	*Park7*	*Cox7b*	*Uqcrq*	*Rps29*	*Cox7b*	*Cox6c*
30	*Nmrk1*	*Slc17a1*	*Nat8*	*Cda*	*Tfap2b*	*Tpt1*	*Rpl39*	*S100g*	*Dbi*

Rank	Cluster 9	Cluster 10	Cluster 11	Cluster 12	Cluster 13	Cluster 14	Cluster 15	Cluster 16	Cluster 17	Cluster 18
1	*Egfl7*	*S100g*	*mt-Atp6*	*Apela*	*Fau*	*Atp6v1g3*	*Atp6v1g3*	*Rps27*	*Ccl5*	*Cd74*
2	*Ifitm3*	*Defb1*	*Slc34a1*	*Fxyd4*	*Tmsb4x*	*Atp6v1c2*	*Tmem213*	*Rps18*	*Nkg7*	*H2-Aa*
3	*Emcn*	*Calb1*	*mt-Cytb*	*Hsd11b2*	*Rps9*	*Itm2b*	*Slc4a1*	*Rps19*	*Gzma*	*H2-Ab1*
4	*Ly6c1*	*Klk1*	*mt-Nd1*	*Cdh16*	*Rps27*	*Pgrmc1*	*Aqp6*	*Rpl18a*	*AW112010*	*C1qa*
5	*Tm4sf1*	*Tmem52b*	*mt-Nd4*	*Aqp2*	*Cd52*	*Tmem213*	*Car2*	*Rps24*	*Tmsb4x*	*C1qc*
6	*Meis2*	*Pgam2*	*mt-Co3*	*Npnt*	*Cd74*	*Car2*	*Rhbg*	*Rps15a*	*Fcer1g*	*C1qb*
7	*Klf2*	*Mt1*	*mt-Co2*	*Aqp3*	*Tmsb10*	*S100a1*	*Atp6v1a*	*Rps7*	*Lgals1*	*Cst3*
8	*Sparc*	*Wnk1*	*mt-Nd2*	*Serinc3*	*Rpl18a*	*Spink8*	*Tfcp2l1*	*Rps3*	*Tyrobp*	*Fcer1g*
9	*Ly6e*	*Spp1*	*mt-Co1*	*Mal*	*Coro1a*	*Hmx2*	*Adgrf5*	*Rps16*	*Pfn1*	*H2-Eb1*
10	*Slc9a3r2*	*S100a1*	*Slc27a2*	*Cd24a*	*Rps27a*	*Atp6v0d2*	*Defb1*	*Rpl32*	*Cd52*	*Tyrobp*
11	*Plpp1*	*Clu*	*Lrp2*	*Aif1l*	*Rps16*	*Slc26a4*	*Atp6v1e1*	*Rps6*	*Tmsb10*	*B2m*
12	*Pbx1*	*Wfdc2*	*Acsm2*	*S100a11*	*Rpl34*	*Atp6v0e*	*Mme*	*Rps23*	*H2-D1*	*Apoe*
13	*Id3*	*Cox7b*	*Slc22a30*	*Gstm2*	*Rps14*	*Atp6v1a*	*Atp6v0d2*	*Rpl17*	*Rac2*	*Ctss*
14	*Nrp1*	*Tmem213*	*mt-Nd5*	*Scd2*	*B2m*	*Foxi1*	*Oxgr1*	*Rps14*	*H2afz*	*Tmsb4x*
15	*Crip2*	*Atp1a1*	*Timp3*	*Pdzk1ip1*	*Rpl13*	*Atp6v0b*	*Foxi1*	*Rplp2*	*Actb*	*Cd52*
16	*Kdr*	*Ppp1r1a*	*Fut9*	*Adgrg1*	*Rps24*	*Atp6v1e1*	*Uqcrb*	*Rps4x*	*B2m*	*Aif1*
17	*Plat*	*Ldhb*	*Cyp4b1*	*Wfdc2*	*H3f3a*	*Atp6v1f*	*Atp6v0e*	*Rplp0*	*Klrd1*	*Lyz2*
18	*B2m*	*Nudt4*	*mt-Nd3*	*Atp1b1*	*Rps11*	*Car12*	*Atp6v1f*	*Rps13*	*Prf1*	*Lst1*
19	*Ifitm2*	*Atp5h*	*Slc47a1*	*Nudt4*	*Rps29*	*Ociad2*	*Cox7a1*	*Rplp1*	*Klf2*	*Ly86*
20	*Ptma*	*Cox6c*	*Galnt11*	*Aplp2*	*Rps4x*	*Krt7*	*Pam*	*Rpl12*	*Arpc1b*	*H2-DMa*
21	*Ptprb*	*Atp5o-1*	*Ugt2b38*	*Tmsb4x*	*Rps5*	*Ckmt1*	*Tmem61*	*Rps5*	*S100a10*	*Cd81*
22	*Cd81*	*Atp5j*	*mt-Nd4l*	*Cdh1*	*Rps13*	*Hexb*	*Serpinb9*	*Rps29*	*Rps11*	*Fau*
23	*Cd24a*	*Atp5e*	*Slc22a6*	*Epcam*	*H2-D1*	*Atp6v1d*	*Atp6v1b1*	*Rps3a1*	*Actg1*	*H2-D1*
24	*Ehd3*	*Cox5b*	*Neat1*	*Defb1*	*Rps19*	*Ldhb*	*Atp6v0b*	*Rpl13*	*Rps27*	*H2-DMb1*
25	*H2-D1*	*Mt2*	*Zbtb20*	*Tspan8*	*Ly6e*	*Hepacam2*	*Efhd1*	*Rpl39*	*Cfl1*	*Rpl18a*
26	*Plpp3*	*Slc12a3*	*Tcn2*	*Sh3bgrl3*	*Rpl37a*	*Aldh1l1*	*Kit*	*Rpl23a*	*H2-K1*	*Rps29*
27	*Pecam1*	*Atp5k*	*Pck1*	*Tfcp2l1*	*Rpl37*	*Emb*	*Chchd10*	*Rps8*	*Crip1*	*Ifi27l2a*
28	*Eng*	*Atp1b1*	*Neu1*	*Hes1*	*Rps3a1*	*Tfcp2l1*	*Aldh1l1*	*Tmsb10*	*Gzmb*	*Wfdc17*
29	*Gimap4*	*Kl*	*Mat2a*	*Rhcg*	*Rps23*	*Lsm6*	*Pgrmc1*	*Rps9*	*Klrk1*	*Cxcl16*
30	*Igfbp5*	*Atp5g1*	*Kcnj15*	*Cldn8*	*Rps15a*	*Insrr*	*Ckmt1*	*Rps20*	*Ptprc*	*Fxyd5*

Differentially expressed features in each cluster of adult mouse kidney (Supplementary Fig. S2**a**) are shown. Features were extracted using Scanpy version 1.4.4.post1 (https://scanpy.readthedocs.io/en/stable/) (Wilcoxon rank-sum test).

Supplementary Table S1-3.
Differentially expressed features in each cluster
(mouse kidney at embryonic day 14.5).

Rank	Cluster 0	Cluster 1	Cluster 2	Cluster 3	Cluster 4	Cluster 5	Cluster 6	Cluster 7	Cluster 8	Cluster 9	Cluster 10	Cluster 11
1	*Fabp4*	*Col1a2*	*Aldh1a2*	*Clu*	*Cited1*	*Meg3*	*Krt18*	*Wfdc2*	*Ndufa4l2*	*Wt1*	*Lum*	*Fcer1g*
2	*Egfl7*	*Zeb2*	*Ccnd2*	*Fxyd2*	*Crym*	*Sfrp1*	*Epcam*	*Epcam*	*Ebf1*	*Pcp4*	*Dcn*	*Rac2*
3	*Icam2*	*Gucy1a3*	*Fhl2*	*Phgdh*	*Robo2*	*Dlk1*	*Cldn3*	*Spint2*	*Tm4sf1*	*Gm266*	*Col1a1*	*Coro1a*
4	*Plvap*	*Col3a1*	*Maged2*	*Atp1b1*	*Uncx*	*Igfbp5*	*Wfdc2*	*Tfap2b*	*Cald1*	*Mafb*	*Sfrp2*	*Tyrobp*
5	*Crip2*	*Ecm1*	*Lgals1*	*Npnt*	*Gas1*	*Col1a1*	*Cldn7*	*Cldn6*	*Lhfp*	*Cldn5*	*Col3a1*	*Arhgdib*
6	*S100a16*	*Lgals1*	*Cdkn1c*	*Stard10*	*Fxyd6*	*Cdkn1c*	*Gata3*	*Ldhb*	*Pdgfrb*	*Pax8*	*Lgals1*	*Tmsb4x*
7	*Emcn*	*Lbh*	*Pbx1*	*Pdzk1*	*Rspo1*	*Alcam*	*Krt8*	*Atp1b1*	*Nrp1*	*Plat*	*Tbx18*	*Lst1*
8	*Rasip1*	*Tmsb4x*	*Hic1*	*Tst*	*Six2*	*Crabp1*	*Cldn6*	*Rab3ip*	*Ace2*	*Tmem37*	*Car3*	*Arpc1b*
9	*Gimap4*	*Ace2*	*Gpc3*	*Steap2*	*Capn6*	*Lgals1*	*Spint2*	*Fstl1*	*Fhl2*	*Bcam*	*Col1a2*	*Gmfg*
10	*Cldn5*	*Bgn*	*Polr2m*	*Pcbd1*	*Spock2*	*Foxd1*	*Wfdc15b*	*Chchd10*	*Mgp*	*Eif3m*	*Mgp*	*Cd53*
11	*Cd34*	*Pcolce*	*Foxd1*	*Mpc2*	*Vcan*	*Mfap2*	*Rprm*	*Pantr1*	*Kcnj8*	*Tspan13*	*Fzd1*	*Laptm5*
12	*Pecam1*	*Meg3*	*Crabp1*	*Clec18a*	*Ttc28*	*Olfml3*	*Cldn8*	*Cldn7*	*Heyl*	*Foxc2*	*Col6a1*	*Sat1*
13	*Cdh5*	*Col1a1*	*Shisa3*	*Calml4*	*Tpm2*	*Tgfbi*	*Calb1*	*Pdgfa*	*Tpm1*	*Siva1*	*Sparc*	*Cyba*
14	*Esam*	*Emilin1*	*Lsp1*	*Hrsp12*	*Eya1*	*Gpc3*	*Mgst1*	*Ly6a*	*Mef2c*	*Cmtm7*	*Mylk*	*Cd52*
15	*Fabp5*	*Ptn*	*Dlk1*	*Cyba*	*Nnat*	*6330403K07Rik*	*Hoxb7*	*Cd24a*	*7-Sep*	*Hist3h2ba*	*Myl9*	*B2m*
16	*Lmo2*	*Olfml3*	*Ptn*	*Ly6a*	*Zbtb20*	*Maged2*	*Cdh16*	*Mecom*	*Rasl11a*	*Epb41l5*	*Phlda1*	*Ptpn18*
17	*Ecscr*	*Lhfp*	*Meis2*	*Folr1*	*Espn*	*Hic1*	*Cldn4*	*Sostdc1*	*Akr1b7*	*Golim4*	*Rcn3*	*Ctsc*
18	*Gchfr*	*Gpc3*	*Emp3*	*Osr2*	*Fam213a*	*Igfbp6*	*Fam149a*	*Ezr*	*Gucy1b3*	*Ncam1*	*Cldn11*	*Fcgr3*
19	*Ramp2*	*Emp3*	*Tgfbi*	*Ddah1*	*Tsc22d1*	*Mfap4*	*Kcnk1*	*Pou3f3*	*Actn1*	*Igfbp7*	*Nfib*	*Psmb8*
20	*Gimap6*	*Snai2*	*Mest*	*Chchd10*	*Meox2*	*Plagl1*	*Mal2*	*Pcbd1*	*Zeb2*	*Ctnnal1*	*Fbln2*	*Fyb*
21	*Kdr*	*Cnn2*	*Meis1*	*Hnf4a*	*Bcl2*	*Col3a1*	*Plac8*	*Atp1a1*	*Acta2*	*Nme4*	*Col9a1*	*Clec4a2*
22	*Vamp5*	*Cald1*	*Cald1*	*Emid1*	*Pax2*	*Crabp2*	*Tacstd2*	*Mal*	*Cox4i2*	*Srgap1*	*Col6a2*	*Spi1*
23	*Gimap1*	*Nr2f2*	*Anxa2*	*Ezr*	*Osr1*	*Selm*	*Bex1*	*Crb3*	*Col3a1*	*Osr2*	*Meg3*	*Aif1*
24	*Cd93*	*Pid1*	*Tuba1b*	*Ivns1abp*	*Hs3st6*	*Smoc2*	*Tbx3*	*Hoxd9*	*Col1a2*	*Cxx1a*	*Atp2b1*	*Lyz2*
25	*Gng11*	*Cfh*	*Col1a1*	*Cldn6*	*Gm266*	*Aldh1a2*	*Mpped2*	*Hipk2*	*Asb4*	*Fxyd6*	*Tpm2*	*Actr3*
26	*Prkcdbp*	*Lsp1*	*Cxcl12*	*Gm266*	*Cd24a*	*Postn*	*Pcbd1*	*Emb*	*Maged2*	*Vdac1*	*Lsp1*	*Apoe*
27	*Aplnr*	*Maged2*	*Fibin*	*Slc39a5*	*Hmcn1*	*Rbp1*	*Ldhb*	*Hoxb8*	*Lgals1*	*Proser2*	*Bambi*	*Cx3cr1*
28	*Gnai2*	*Ccdc80*	*Eva1b*	*Igfbp7*	*Cenpw*	*Ccnd2*	*Bex4*	*Hoxd8*	*Dkk2*	*Scube3*	*Gata2*	*Ms4a6c*
29	*Eng*	*Cdh11*	*Arpc1b*	*Rab3ip*	*Wt1*	*C1qtnf2*	*Bcam*	*Wfdc15b*	*Gm13889*	*Med28*	*Mfap4*	*Gpx1*
30	*Ctla2a*	*Marcks*	*Emilin1*	*Slc9a3r1*	*Kif26b*	*Peg3*	*Hoxd4*	*Lamb1*	*Fn1*	*Efnb1*	*Selm*	*Tspo*

Features in each cluster of embryonic mouse kidney at day 14.5 (Supplementary Fig.

S2**b**) are shown. Features were extracted using Scanpy version 1.4.4.post1 (https://scanpy.readthedocs.io/en/stable/) (Wilcoxon rank-sum test).

Supplementary Table S1-4.
Differentially expressed features in each cluster (human kidney organoid).

Rank	Cluster 0	Cluster 1	Cluster 2	Cluster 3	Cluster 4	Cluster 5	Cluster 6	Cluster 7	Cluster 8	Cluster 9
1	*COL1A1*	*IGFBP5*	*HNRNPA1*	*HMGB2*	*TMSB4X*	*MAGI2-AS3*	*PAX8*	*FIBIN*	*TUBB2B*	*NNAT*
2	*COL3A1*	*BST2*	*PRRX1*	*H2AFZ*	*GNG11*	*MAFB*	*KRT18*	*COL2A1*	*SOX2*	*MYL1*
3	*LGALS1*	*ZEB2*	*MAB21L2*	*HMGB1*	*EGFL7*	*CLIC5*	*BCAM*	*COL9A2*	*AP1S2*	*PITX2*
4	*CRABP1*	*NRK*	*ZFHX4*	*CENPF*	*CALM1*	*PODXL*	*EMX2*	*COL9A3*	*TTYH1*	*PDLIM3*
5	*COL1A2*	*NR2F1*	*RPL21*	*HMGN2*	*FKBP1A*	*WT1*	*IGFBP7*	*MEST*	*RFX4*	*CKB*
6	*TPM1*	*RPS18*	*NPM1*	*UBE2C*	*TMSB10*	*NPHS2*	*MIF*	*SFRP2*	*FZD3*	*NEB*
7	*PCOLCE*	*CXCL12*	*MDK*	*BIRC5*	*CAV1*	*DUSP23*	*ATP5IF1*	*FN1*	*MSX1*	*CDH15*
8	*IL11RA*	*RPL10A*	*MAB21L1*	*TOP2A*	*FSCN1*	*PTPRO*	*CLU*	*MIA*	*SRP14*	*HES6*
9	*MEIS2*	*COL1A1*	*H3F3A*	*TUBA1B*	*ESAM*	*TCF21*	*KRT19*	*PTN*	*AC004540.2*	*TPM2*
10	*RPL10*	*PCDH9*	*NNAT*	*CKS1B*	*PLXND1*	*CPXM1*	*VAMP8*	*CNMD*	*FABP7*	*RPL35*
11	*MEIS1*	*NR2F2*	*CXCL14*	*MKI67*	*PLVAP*	*SPINT2*	*CD24*	*CPE*	*C4orf48*	*MYOD1*
12	*MEST*	*NR2F2-AS1*	*RPL13A*	*PCLAF*	*RAMP2*	*MPP5*	*PAX2*	*RPL10*	*PAX3*	*ACTC1*
13	*CALD1*	*RPL12*	*COL9A2*	*NUSAP1*	*RPS27L*	*ITM2B*	*SPINT2*	*PMP22*	*WNT1*	*PPP1R14B*
14	*VIM*	*PDGFRB*	*RPS13*	*PTTG1*	*ARHGAP29*	*ANXA1*	*S100A16*	*RPL13A*	*APLP1*	*CELF2*
15	*CXCL12*	*RPL13*	*RPL6*	*SMC4*	*MYL6*	*VAMP8*	*WT1*	*COL9A1*	*PCSK1N*	*EEF1G*
16	*NR2F2*	*GAS5*	*RPS3A*	*CENPW*	*TP53I11*	*MXRA8*	*GSTP1*	*CCN1*	*MAP1B*	*PDLIM4*
17	*EDNRA*	*TPM1*	*EIF3E*	*H2AFX*	*ACTB*	*BCAM*	*SNCA*	*RPL15*	*PTN*	*RPL18A*
18	*MGP*	*PDGFRA*	*RPS11*	*TPX2*	*PECAM1*	*AIF1*	*ATP1B1*	*OGN*	*BEX1*	*AL589740.1*
19	*FSTL1*	*SELENOP*	*RPL35A*	*CKS2*	*S100A16*	*BST2*	*CSTB*	*MEOX2*	*DMD*	*RPL4*
20	*TAGLN*	*PNRC1*	*CRABP2*	*TMSB15A*	*CLDN5*	*TGFBR3*	*CDH6*	*COL12A1*	*APCDD1*	*RPS2*
21	*COL6A3*	*NPW*	*RPS15A*	*NUCKS1*	*PFN1*	*SBSPON*	*FTL*	*RPL3*	*TUBA1A*	*SIX1*
22	*FIBIN*	*SNHG8*	*RPL30*	*AURKB*	*ECSCR*	*SOST*	*LHX1*	*RPS4X*	*WLS*	*ZNF106*
23	*RPL28*	*SNHG29*	*PTMA*	*STMN1*	*GYPC*	*EIF3M*	*IFITM3*	*ECRG4*	*ZIC2*	*PDGFC*
24	*PCDH18*	*COL21A1*	*HOXA10*	*MAD2L1*	*TM4SF18*	*SPOCK2*	*UBL5*	*COL11A1*	*LYPD1*	*RPS5*
25	*FLRT2*	*SESN3*	*RPL22*	*GTSE1*	*CD34*	*NPHS1*	*CLDN3*	*GAS2*	*CDH6*	*ARPP21*
26	*TPM2*	*IFI27L2*	*VIM*	*CDK1*	*APLN*	*LINC00472*	*COX7C*	*RPL18A*	*RMST*	*RPL29*
27	*COL6A2*	*RACK1*	*EEF1A1*	*ASPM*	*COL4A1*	*SDC2*	*KRT8*	*CNN3*	*NTRK2*	*H19*
28	*NFIB*	*RPS8*	*CD24*	*H2AFV*	*CDH5*	*GADD45A*	*QPRT*	*IL11RA*	*ID2*	*DES*
29	*PLAC9*	*RPL28*	*MIR100HG*	*ZWINT*	*CFL1*	*TYRO3*	*LYPD1*	*PLAC9*	*CKB*	*KLHL41*
30	*RPS18*	*RPS7*	*RPS14*	*HMGB3*	*CCDC85B*	*TJP1*	*LHX1-DT*	*RPS15*	*TTC3*	*ERBB3*

Differentially expressed features in each cluster of human kidney organoid (Supplementary Fig. S2**c**) are shown. Features were extracted using Scanpy version

1.4.4.post1 (https://scanpy.readthedocs.io/en/stable/) (Wilcoxon rank-sum test).

Supplementary Table S2.
Differentially expressed features in each cluster (human embryonic kidney).

Rank	Cluster 0	Cluster 1	Cluster 2	Cluster 3	Cluster 4	Cluster 5	Cluster 6	Cluster 7
1	*MYL9*	*COL1A1*	*OLFM3*	*PODXL*	*PAX8*	*NPHS2*	*SMIM24*	*H2AFZ*
2	*SPOCK2*	*COL1A2*	*MAFB*	*TPPP3*	*IGFBP7*	*NR4A1*	*APOE*	*TUBA1B*
3	*PTH1R*	*COL3A1*	*EIF3M*	*MME*	*RPLP0*	*ARID5B*	*FTL*	*HMGB2*
4	*MME*	*LGALS1*	*BST2*	*WT1*	*ADAMTS1*	*CTGF*	*MPC2*	*HMGB1*
5	*AIF1*	*PCOLCE*	*IGFBP4*	*THSD7A*	*RPL5*	*IDI1*	*RIDA*	*MAD2L1*
6	*ANXA2*	*PTN*	*LOXL1*	*MPP5*	*RPL35A*	*CPXM1*	*LINC01781*	*NUSAP1*
7	*PODXL*	*MOXD1*	*PLOD2*	*MAFB*	*RACK1*	*THSD7A*	*ACAA2*	*TUBB*
8	*S100A6*	*TGFBI*	*CTGF*	*SOST*	*QPRT*	*MXRA8*	*CUBN*	*TYMS*
9	*PTPRO*	*COL6A3*	*TSC22D3*	*MXRA8*	*TSPAN12*	*NEAT1*	*DSEL*	*LMNB1*
10	*SPARC*	*TNC*	*TPM1*	*SELENOP*	*RPL21*	*BST2*	*MT-CO3*	*SMC4*
11	*THSD7A*	*MARCKS*	*HSP90B1*	*NPHS2*	*LDHB*	*MYL9*	*LDHB*	*TOP2A*
12	*MPP5*	*EMILIN1*	*FBLN2*	*S100A6*	*RPS3A*	*PODXL*	*MSRB1*	*TMSB15A*
13	*ST6GALNAC3*	*GPC3*	*SLC25A6*	*TMSB4X*	*RPS2*	*NES*	*LGALS2*	*UBE2C*
14	*TMSB4X*	*RPS12*	*TSPAN8*	*MYL9*	*CDH6*	*MME*	*TPT1*	*TK1*
15	*TNNI1*	*AKAP12*	*PPIB*	*DDN*	*TPM1*	*AIF1*	*ETFB*	*RAN*
16	*TPPP3*	*CPE*	*WT1*	*PTPRO*	*ID1*	*CSRP1*	*MT-ND1*	*UBE2T*
17	*NPHS2*	*MDK*	*ITIH5*	*PLTP*	*RPL3*	*CITED2*	*APOM*	*RANBP1*
18	*VIM*	*SERPINH1*	*ORC4*	*AIF1*	*RPSA*	*PTPRO*	*ATP1B1*	*BIRC5*
19	*HTRA1*	*PCDH18*	*ATP5F1A*	*SEMA3B*	*RPS7*	*INSIG1*	*TGIF1*	*ZWINT*
20	*ACTN4*	*RBP1*	*TSPAN3*	*LTBP4*	*RPL29*	*ANXA2*	*MT-ND4*	*MCM7*
21	*TNNT2*	*ALX1*	*SOST*	*PTH1R*	*RPL19*	*SPARC*	*GNG11*	*PBK*
22	*TUBB2A*	*CTSC*	*EFNB1*	*NES*	*AKAP12*	*SARAF*	*DBI*	*PTTG1*
23	*NES*	*IGFBP5*	*SLC16A1*	*SHISA2*	*SLIT3*	*SPOCK2*	*MT-CO1*	*CKS1B*
24	*PLTP*	*RPL13A*	*ITM2C*	*KLRB1*	*RPL11*	*MSMO1*	*SLC3A1*	*CDK1*
25	*GSN*	*RPL6*	*C17orf58*	*SPOCK2*	*RPL10A*	*HERPUD1*	*DNPH1*	*CENPF*
26	*TGFBR3*	*RPS15A*	*DSC2*	*BST2*	*RPL8*	*HES4*	*CLEC18B*	*PTMA*
27	*DDN*	*RPL30*	*PDIA4*	*UBB*	*RPS13*	*CRB2*	*CYB5A*	*AURKB*
28	*LOX*	*FN1*	*PCDH9*	*TGFBR3*	*LYPD1*	*HSPA1A*	*ANXA4*	*HMGB3*
29	*HMGN3*	*FBLN5*	*CLDN5*	*RIPOR1*	*EEF1A1*	*COL4A4*	*MT-ND3*	*GAPDH*
30	*VASN*	*BGN*	*PAX8*	*CPXM1*	*CLEC18B*	*DNAJA1*	*VCAN*	*H2AFY*

Rank	Cluster 8	Cluster 9	Cluster 10	Cluster 11	Cluster 12	Cluster 13	Cluster 14
1	*CRABP2*	*CD24*	*CPXM1*	*GNAS*	*CLDN4*	*CD74*	*ARHGDIB*
2	*NNAT*	*TUBB2B*	*BST2*	*LGALS1*	*KRT18*	*RAMP2*	*LSP1*
3	*TMEM100*	*RPL8*	*MAFB*	*TPM2*	*WFDC2*	*EGFL7*	*SRGN*
4	*LYPD1*	*RPSA*	*MXRA8*	*RPS12*	*CLU*	*PRCP*	*RPLP1*
5	*MARCKSL1*	*RPS6*	*GADD45A*	*COL1A2*	*KRT19*	*GNG11*	*CD74*
6	*RPL5*	*RPS2*	*DUSP1*	*RPLP1*	*KRT8*	*PLVAP*	*RPLP2*
7	*RPL3*	*CLDN4*	*NPHS2*	*PCOLCE*	*MMP7*	*IGFBP5*	*LAPTM5*
8	*FAM213A*	*HMGA1*	*SOST*	*NPM1*	*CTSH*	*TM4SF1*	*SH3BGRL3*
9	*MDK*	*RPL3*	*SEMA3B*	*ACTA2*	*S100A11*	*TMSB10*	*CORO1A*
10	*TUBA1A*	*RBM47*	*CTGF*	*POSTN*	*CYB5A*	*HLA-E*	*LST1*
11	*SIX1*	*EFNA1*	*ITIH5*	*MDK*	*SCIN*	*RNASE1*	*PFN1*
12	*EYA1*	*CLDN3*	*THSD7A*	*SH3BGRL*	*CLDN3*	*COTL1*	*HLA-DRA*
13	*MEST*	*ELF3*	*EIF3M*	*EMCN*	*AOC1*	*CCDC85B*	*RPL28*
14	*EEF1A1*	*RPS19*	*PODXL*	*RPL10*	*ELF3*	*ITM2A*	*TYROBP*
15	*RPL13*	*PHGDH*	*TSC22D3*	*RPL23A*	*MT-CO1*	*TIE1*	*CYBA*
16	*RPL35A*	*RPLP0*	*ITM2C*	*MAGED2*	*TNFRSF12A*	*HSPB1*	*HLA-DRB1*
17	*RPL6*	*PAX8*	*WT1*	*RPL6*	*SAT1*	*MARCKS*	*PTPRE*
18	*RPL7A*	*MT-CO3*	*NEAT1*	*COL1A1*	*GSTM3*	*EMCN*	*RPS19*
19	*RACK1*	*RPL7*	*COL9A1*	*MECOM*	*MT-CO3*	*CDH5*	*HCST*
20	*RPS7*	*LDHB*	*JUN*	*RPL7*	*PHLDA2*	*PECAM1*	*TMSB10*
21	*RPS3A*	*KRT8*	*MPP5*	*RPS14*	*GMNN*	*S100A10*	*ITGB2*
22	*RPL7*	*LIMCH1*	*APLP2*	*RPS27A*	*CAPS*	*GYPC*	*SPI1*
23	*RPL27A*	*KRT18*	*PTPRO*	*RPL27A*	*ZNF44*	*ICAM2*	*HLA-DPB1*
24	*RPS6*	*RPL12*	*TPPP3*	*TMSB10*	*FXYD5*	*ELK3*	*GMFG*
25	*SINHCAF*	*MAL*	*SNCA*	*RPL13A*	*MT-ND4*	*IFITM3*	*HLA-C*
26	*PHLDA1*	*CARMIL1*	*VASN*	*MYH10*	*PRPS2*	*HSPG2*	*FTH1*
27	*UCHL1*	*CCND1*	*SPARC*	*EBF1*	*KLF5*	*KANK3*	*ANXA1*
28	*WASF3*	*RPL31*	*CITED2*	*RPL34*	*ADH1C*	*IGFBP4*	*RILPL2*
29	*RPSA*	*RPL7A*	*PSAP*	*RPL28*	*AGR2*	*RPL36*	*COTL1*
30	*TUBB*	*ODC1*	*NPHS1*	*RPL14*	*AC129507.4*	*CAVIN2*	*LCP1*

Features in each cluster of human embryonic kidney at week 18 (Supplementary Fig. S7) are shown. Features were extracted using Scanpy version 1.4.4.post1

(https://scanpy.readthedocs.io/en/stable/) (Wilcoxon rank-sum test).

Supplementary Table S3-1.

Features of the root and the non-root cells in nephron progenitors.

Rank	Non-root	Root
1	*Fxyd6*	*Id2*
2	*Phgdh*	*Igfbp5*
3	*Mest*	*H19*
4	*Ttc28*	*Hbb-bs*
5	*Tgfbi*	*Spop*
6	*Fam213a*	*Prrc2b*
7	*Arl6ip1*	*Hbb-bt*
8	*Vcan*	*Rps2*
9	*Rpl39*	*Cd24a*
10	*Tpm2*	*C1qtnf12*
11	*Ubb*	*Ftl1*
12	*Rem1*	*Lman2*
13	*Meox1*	*Mrpl30*
14	*Tcf4*	*Cdc26*
15	*Espn*	*Nedd8*
16	*Cited1*	*Nenf*
17	*Smarcb1*	*Fth1*
18	*Gapdh*	*Sh3bgrl3*
19	*Rpl34*	*Hba-a2*
20	*Rps15a*	*Wfdc2*
21	*Tril*	*Fam107a*
22	*Fzd2*	*Slc25a39*
23	*Son*	*Azin1*
24	*U2af1*	*Frzb*
25	*Jpt1*	*Csrp2*
26	*Tubb2b*	*Znhit1*
27	*Rpl36*	*Zmym5*
28	*Fgfr1*	*Pdgfa*
29	*Utp11*	*Kdelr2*
30	*Jund*	*Gpx7*

Features of the root and the non-root cells are shown. Cells shown in Fig. 4**e** was

analyzed. Top 10% cells having higher root cell probability were defined as the root of nephron progenitors. The rest 90% cells were defined as non-root of nephron progenitors. Features were extracted using Scanpy version 1.4.4.post1 (https://scanpy.readthedocs.io/en/stable/) (Wilcoxon rank-sum test).

Supplementary Table S3-2.
Differentially expressed features between the sub-cluster NP3 and NP4.

Sub-cluster	NP3	NP4
1	*Atp5b*	*Malat1*
2	*Prmt1*	*Rpl23*
3	*Ppia*	*Egr1*
4	*Hmgb1*	*Eef1a1*
5	*Eif5a*	*Rpl9*
6	*Hnrnpk*	*Fxyd6*
7	*Ptma*	*Rpl32*
8	*Ldhb*	*Rps14*
9	*Uqcrc1*	*Rps3a1*
10	*Hsp90aa1*	*Eef2*
11	*Atp5f1*	*Jund*
12	*Mdh1*	*Rps19*
13	*Ddost*	*Rpl26*
14	*Hnrnpm*	*Rps5*
15	*Tmem59*	*Rps16*

Differentially expressed features between the sub-cluster NP3 and NP4 in Fig. 5**a** are shown. Features were extracted using Scanpy version 1.4.4.post1 (https://scanpy.readthedocs.io/en/stable/) (Wilcoxon rank-sum test).

Supplementary Table S4.

Features that define changes from the Population_A1 to the B1 in Fig. 6a.

Rank	Podo vs CS_shape	Early_prox vs CS_shape	Early_Henle vs CS_shape	Distal + Henle vs CS_shape
1	*Igfbp7*	*Fxyd2*	*Irx1*	*Itm2b*
2	*Myl6*	*Calml4*	*Fstl1*	*Sostdc1*
3	*Dpp4*	*Cyba*	*Atp1b1*	*Wfdc15b*
4	*Malat1*	*Rida*	*Epcam*	*Chchd10*
5	*Anxa2*	*Mpc2*	*Mpc2*	*Mal*
6	*Arhgap24*	*Igfbp7*	*Irx2*	*Atp1b1*
7	*Gadd45a*	*Npnt*	*Umod*	*Ly6a*
8	*Sparc*	*Atp1b1*	*Cyba*	*Wfdc2*
9	*Plat*	*Spink1*	*D030045P18Rik*	*Mpc2*
10	*Selenop*	*Aldob*	*Tspan8*	*Epcam*
11	*Cryab*	*Folr1*	*Cldn7*	*Atp1a1*
12	*Cdkn1c*	*Ly6a*	*Cldn6*	*Tfcp2l1*
13	*Rasl11a*	*Cpn1*	*Smagp*	*Nudt4*
14	*Golim4*	*Lrpap1*	*Spint2*	*Tmem52b*
15	*Mmp23*	*Slc9a3r1*	*Sim2*	*Aldoa*
16	*Mafb*	*Pdzk1*	*Rps19*	*Cldn7*
17	*Podxl*	*Fut9*	*Sostdc1*	*Fstl1*
18	*Loxl2*	*Hnf4a*	*Dcdc2a*	*Prdx5*
19	*Eif3m*	*Dcdc2a*	*Pdgfa*	*Tpi1*
20	*Itm2b*	*Igfbp4*	*Rps7*	*Neat1*
21	*Wt1*	*Phgdh*	*Ly6a*	*Cdh1*
22	*Eci2*	*Slc39a5*	*Rbm47*	*Tmem213*
23	*1810037I17Rik*	*Tmem27*	*Pou3f3*	*Spint2*
24	*R3hdml*	*Rps19*	*Serpinh1*	*Ldhb*
25	*Slc9a3r2*	*Ttc36*	*Col18a1*	*Gpi1*
26	*Srgap1*	*Ggt1*	*Paqr5*	*Hoxd8*
27	*Gabarap*	*Adamts1*	*Chchd10*	*Cox8a*
28	*Ctsl*	*Akr7a5*	*Kif21a*	*Tspo*
29	*Csnk1a1*	*Ddah1*	*Itm2b*	*Cdkl1*
30	*Cldn5*	*Tmbim6*	*Ggt1*	*Hoxd9*
31	*Tcf21*	*Lrp2*	*Rps14*	*Ly6e*

32	*Mme*	*Amn*	*Crb3*	*Cldn8*
33	*Map1lc3a*	*Dab2*	*Itga1*	*Cd63*
34	*Enpep*	*Rbm47*	*Prr15l*	*Slc25a3*
35	*Neat1*	*0610005C13Rik*	*Cdh1*	*Atp5g1*
36	*Tmem37*	*Slc34a1*	*Kif12*	*Tesc*
37	*Ackr3*	*Glud1*	*Rpl13*	*Tfap2b*
38	*Hist3h2ba*	*Csad*	*Foxc1*	*Mt1*
39	*Cd59a*	*Sephs2*	*Rpl10*	*Hoxb5os*
40	*Gpx8*	*Keg1*	*Rpl21*	*Atp5c1*
41	*Bsg*	*Sema3c*	*Iah1*	*Col18a1*
42	*Itgb1*	*Pcbd1*	*Saraf*	*Cldn6*
43	*Actn4*	*Dao*	*Glud1*	*Krt8*
44	*Nes*	*Kif12*	*Adamts1*	*Sod2*
45	*Rassf10*	*Gpc4*	*Igfbp7*	*Ndufa6*
46	*Palld*	*Rab11a*	*Spon1*	*Paqr5*
47	*Bcam*	*Cryz*	*Folr1*	*Mecom*
48	*Clic5*	*Dpp4*	*Wnt7b*	*S100g*
49	*Atp6v0e*	*Gm19950*	*Mapk13*	*Rbm47*
50	*Rtl8b*	*Acadm*	*Pantr1*	*Krt18*
51	*Nphs1*	*Cdh6*	—	*Umod*
52	*Nphs2*	—	—	*Cldn10*
53	—	—	—	*Cldn16*
54	—	—	—	*Slc12a3*

To define changes from the Population_A1 to the B1 in Fig. 6, differentially expressed features in the Population_B1 in comparison with the A1 were extracted using Scanpy version 1.4.4.post1 (https://scanpy.readthedocs.io/en/stable/) (rank 1-50). If not included in the rank 1-50 genes, genes used in the cluster annotation (Supplementary Fig. S3) were also applied for the NicheNet analyses (rank 51-).

Supplementary Table S5.
Features that define changes from the Population_A2 to the B2 in Fig. 8a.

Rank	ST (sup) vs ST (G2M)	ST (medulla) vs ST (G2M)
1	*Igfbp5*	*Sparc*
2	*Rps14*	*Ogn*
3	*Ccnd2*	*Fbln5*
4	*Aprt*	*Igfbp3*
5	*Rpl17*	*G0s2*
6	*Rpl9*	*S100a11*
7	*Rpl6*	*Ace2*
8	*Eef2*	*Myl6*
9	*Rps18*	*Col1a2*
10	*Rpl18a*	*Acta2*
11	*Rps27a*	*Col6a1*
12	*Rpl32*	*Myl9*
13	*Rps7*	*Plac8*
14	*Rpl26*	*Rpl10*
15	*Aldoa*	*Dcn*
16	*Rpl11*	*Pcolce*
17	*Eef1a1*	*Lox*
18	*H19*	*S100a6*
19	*Rpl23*	*Tpm1*
20	*Rpl24*	*Tmsb4x*
21	*Rps6*	*Col6a2*
22	*Rps3a1*	*Vim*
23	*Naca*	*Laptm4a*
24	*Rpl10*	*Serping1*
25	*Eef1g*	*Col14a1*
26	*Rps10*	*Igfbp7*
27	*Rps13*	*Cfh*
28	*Mfap4*	*Col3a1*
29	*Rpl3*	*Vegfd*
30	*Rps9*	*Rcn3*
31	*Mest*	*Col1a1*

32	*Rps5*	*Ctsl*
33	*Rps8*	*Cdc42ep3*
34	*Rpl19*	*Pmp22*
35	*Rpl14*	*Aspn*
36	*Rpl13*	*Cald1*
37	*Rpl4*	*Serpinh1*
38	*Rps26*	*Anxa2*
39	*Fau*	*Myl12a*
40	*Rpl8*	*Eef2*
41	*Rps2*	*Cd81*
42	*Rpl10a*	*Gpc3*
43	*Rpl12*	*S100a10*
44	*Rps17*	*Gnas*
45	*Rps15a*	*Prdx5*
46	*Crabp2*	*Itm2b*
47	*Ogn*	*7-Sep*
48	*Fth1*	*Lbh*
49	*Rpl7*	*App*
50	*Rps23*	*Bst2*
51	*Foxd1*	*Clca3a1*
52	*Gdnf*	—

Differentially expressed features are summarized. Top 50 features were extracted using Scanpy version 1.4.4.post1 (https://scanpy.readthedocs.io/en/stable/) (Wilcoxon rank-sum test). Genes used in the cluster annotation (Supplementary Fig. S3) were also listed (Rank 51-).
