# Supplementary material for: Single cell RNA sequencing uncovers cellular developmental sequences and novel potential intercellular communications in embryonic kidney
Source: Sci Rep. 2021 Jan 8;11:73. doi: 10.1038/s41598-020-80154-y (PMC7794461; doi:10.1038/s41598-020-80154-y)
Supplement: Supplementary file 2 — Supplementary Information 2. [file 41598_2020_80154_MOESM2_ESM.pdf]

Supplementary Figure S1.

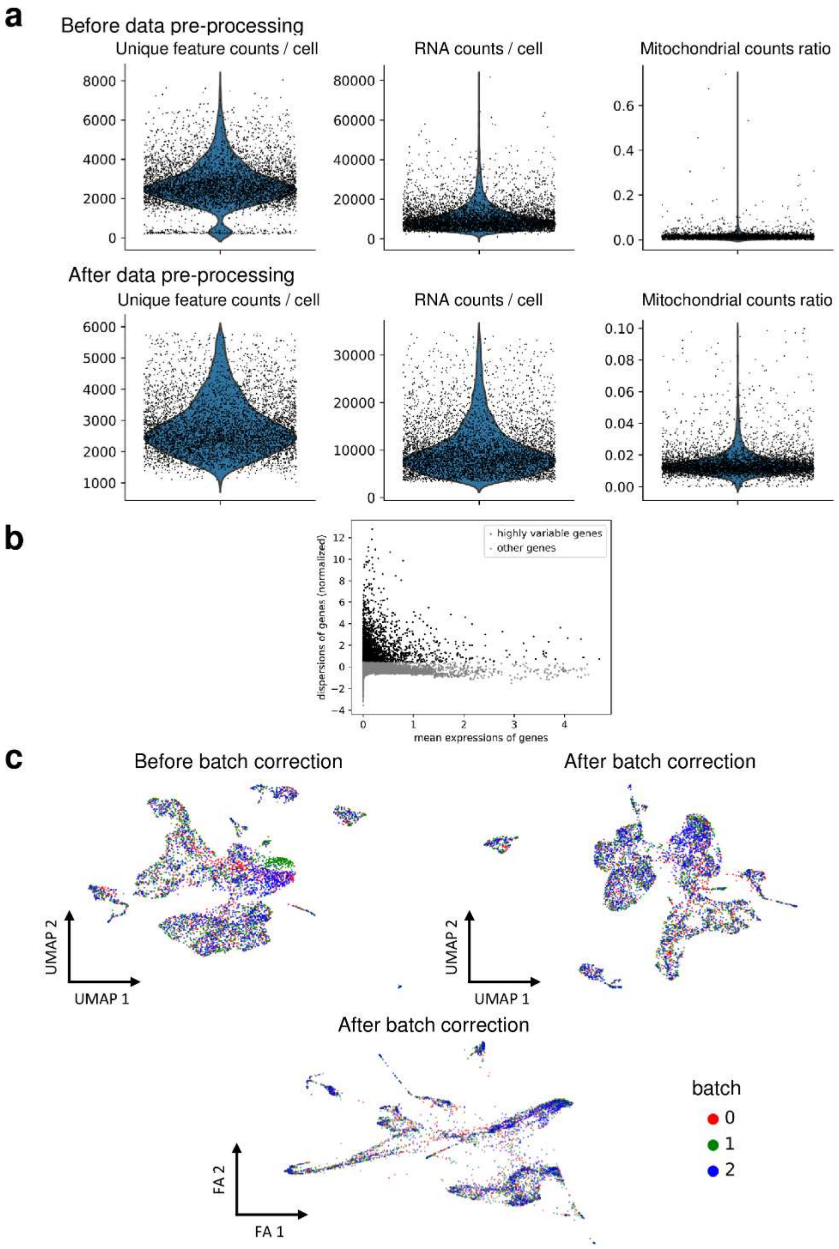

Pre-processing of single cell RNA sequencing data obtained from mouse kidney at embryonic day 18.5.

(**a**) Cells with unique feature counts over 5,800/cell or less than 1,000/cell, RNA counts over 35,000/cell, or mitochondrial counts ratio over 10% were filtered out in the data pre-processing. (**b**) Dispersions and mean expressions of genes. Highly variable 3,000 genes are indicated by black dots. (**c**) Batch effects were corrected by batch balanced k nearest neighbors (BBKNN). For visualization in a two-dimentional space, dimensionality was reduced by UMAP. Three colors indicate three batches. The distribution of each batch was also visualized by PAGA-initialized ForceAtlas2. All data were analyzed using SRA Toolkit version 2.10.1

(https://github.com/ncbi/sra-tools), Cell Ranger software version 2.2 count pipeline (10x GENOMICS, Pleasanton, CA), and Scanpy version 1.4.4.post1 (https://scanpy.readthedocs.io/en/stable/).

Supplementary Figure S2.

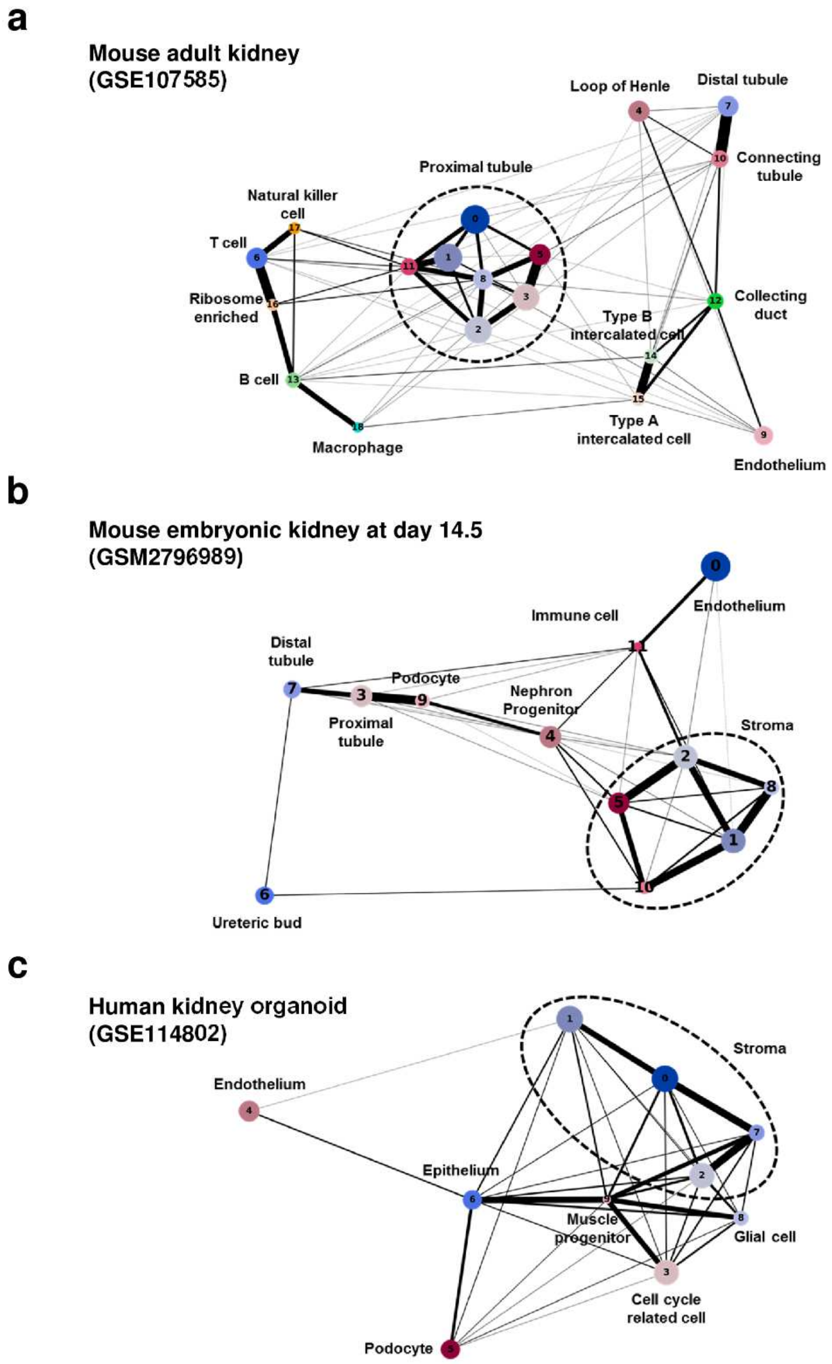

PAGA of adult mouse kidney, embryonic mouse kidney at day 14.5, and human kidney organoid.

PAGA of mouse adult kidney (GSE107585), mouse embryonic kidney at day 14.5 (GSM2796989), and human kidney organoid (GSE114802) are shown. Clusters were annotated by differentially expressed features shown in Supplementary Tables S1-2, 1-3, and 1-4, respectively. All data were analyzed using using SRA Toolkit version 2.10.1

(https://github.com/ncbi/sra-tools), Cell Ranger software version 2.2 count pipeline (10x GENOMICS, Pleasanton, CA), and Scanpy version 1.4.4.post1 (https://scanpy.readthedocs.io/en/stable/).

Supplementary Figure S3-1.

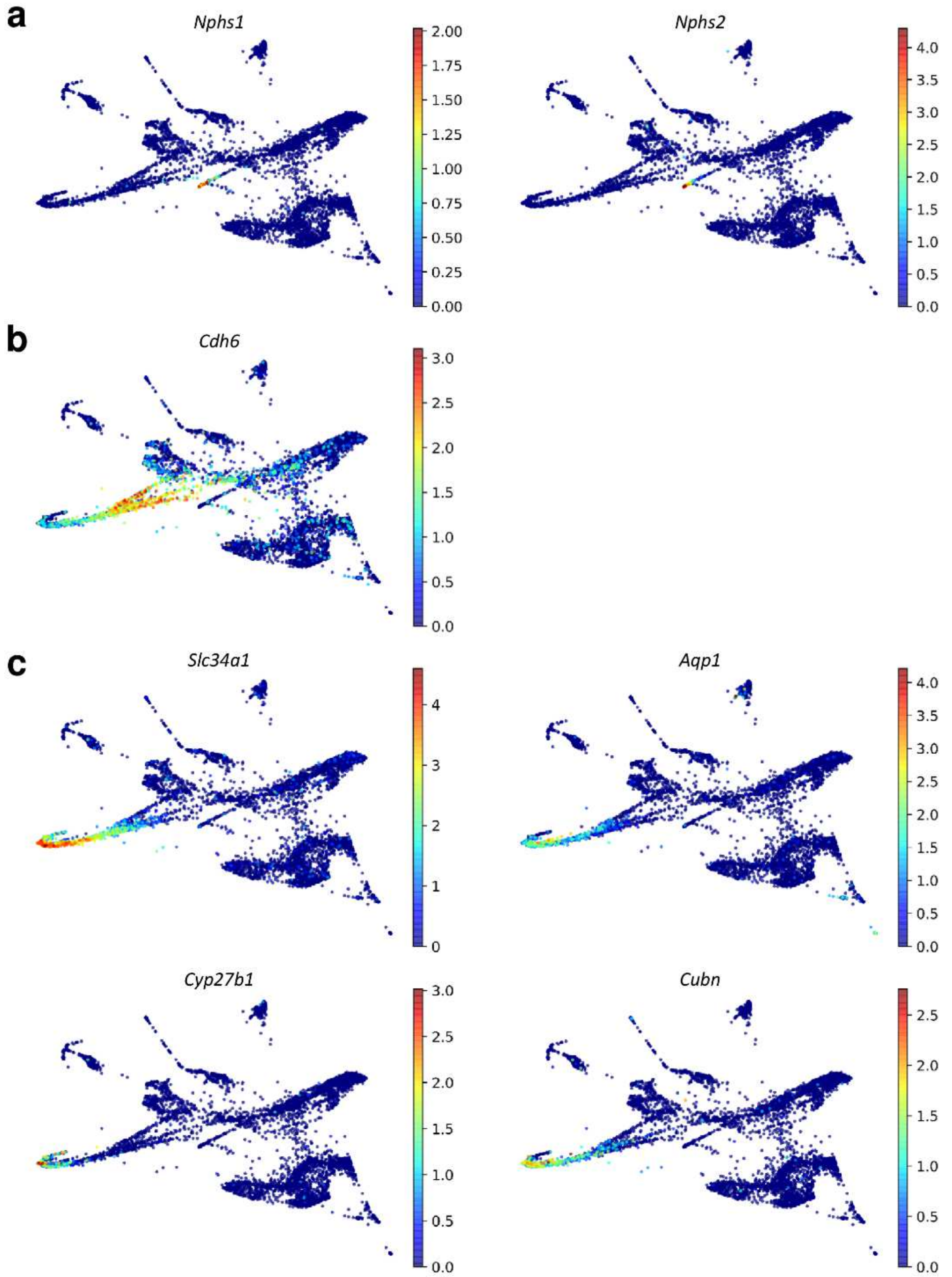

Expression patterns of marker genes for podocytes, early phase proximal tubules, and proximal tubules.

Cluster annotation in Fig. 1 was manually performed based on the expression patterns of the following marker genes: (**a**) *Nphs1* and *Nphs2* for podocytes (Podo in Fig. 1); (**b**) *Cdh6* for early phase proximal tubules (Early_prox); (**c**) *Slc34a1*, *Aqp1*, *Cyp27b1*, and *Cubn* for proximal tubules (Prox). Early_prox cluster in Fig. 1 was annotated based on a previous report, in which cadherin 6 was shown to be temporarily expressed in the proximal tubule progenitors.[1] All data were analyzed using Scanpy version 1.4.4.post1 (https://scanpy.readthedocs.io/en/stable/).

Supplementary Figure S3-2.

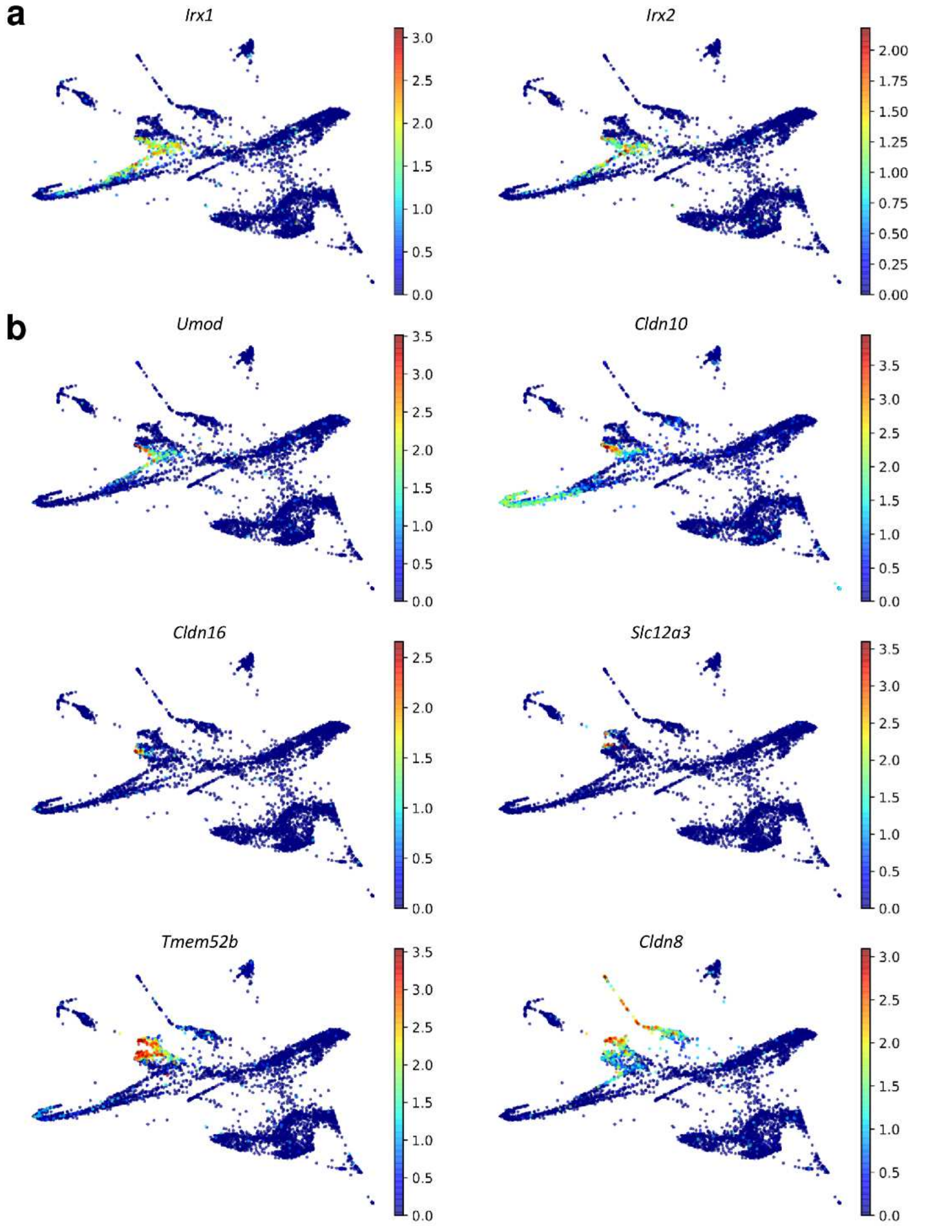

Expression patterns of marker genes for early phase Henle, the loop of Henle, and distal tubules.

Cluster annotation in Fig. 1 was performed based on the following marker genes: (**a**) *Irx1* and *Irx2* for early phase Henle (Early_Henle); (**b**) *Umod*, *Cldn10*, and *Cldn16* for the loop of Henle (Henle); *Scl12a3*, *Tmem52b*, and *Cldn8* for distal tubules (Distal). Several *Cldn* genes were used for the annotation according to the report by Kikuchi-Saishin *et al*.[2] A few of genes were detected across several clusters. *Cldn10* expression in the proximal tubules and *Cldn8* expression in the collecting ducts were observed in accordance with previous reports.[2] All data were analyzed using Scanpy version 1.4.4.post1 (https://scanpy.readthedocs.io/en/stable/).

Supplementary Figure S3-3.

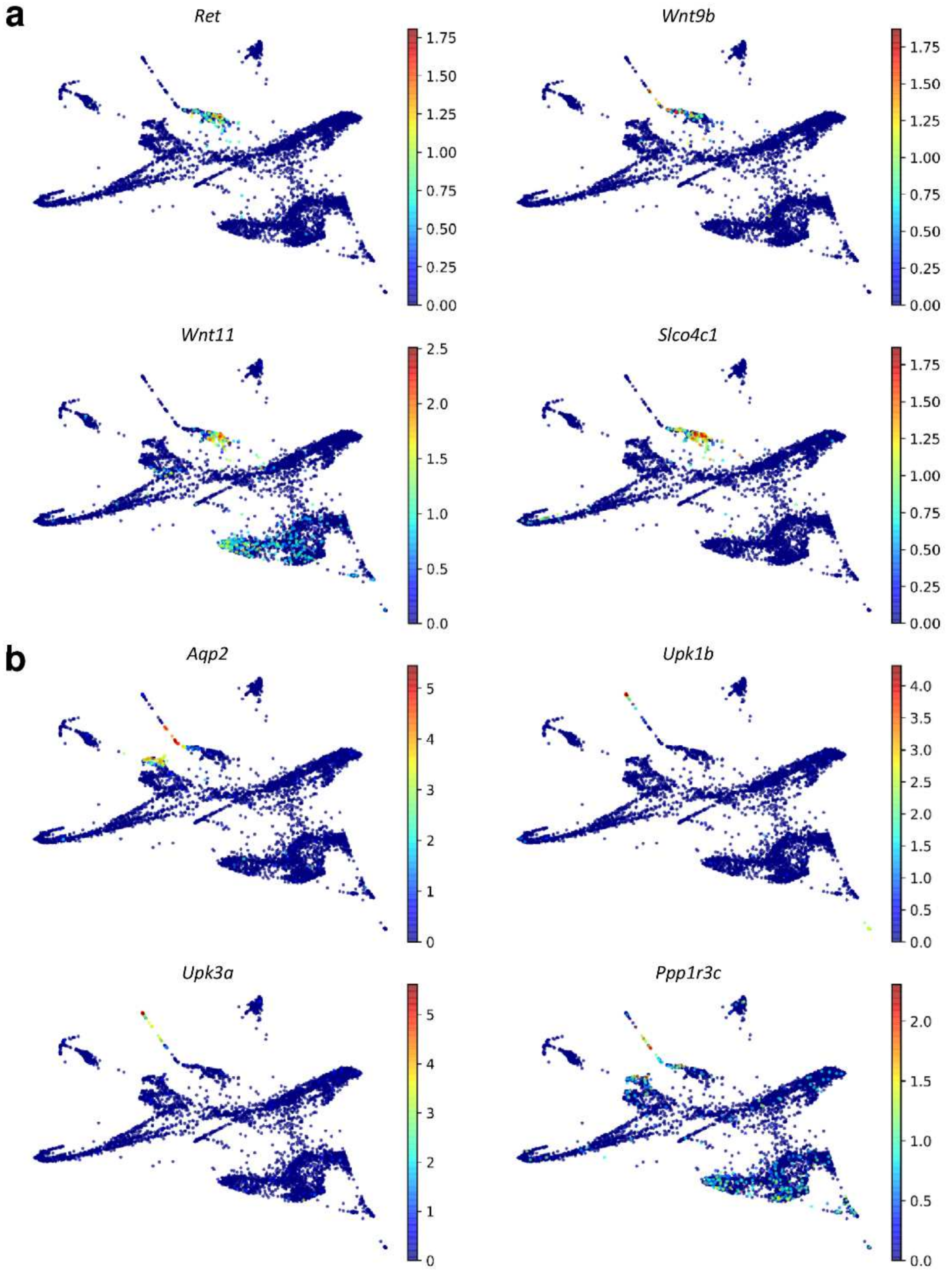

Expression patterns of marker genes for the ureteric bud and collecting duct. Cluster annotation in Fig. 1 was performed based on the following marker genes: (**a**) *Ret*, *Wnt9b*, *Wnt11*, *Slco4c1* for ureteric bud (UB); (**b**) *Aqp2*, *Upk1b*, *Upk3a*, and *Ppp1r3c* for collecting duct (Collect). All data were analyzed using Scanpy version 1.4.4.post1 (https://scanpy.readthedocs.io/en/stable/).

Supplementary Figure S3-4.

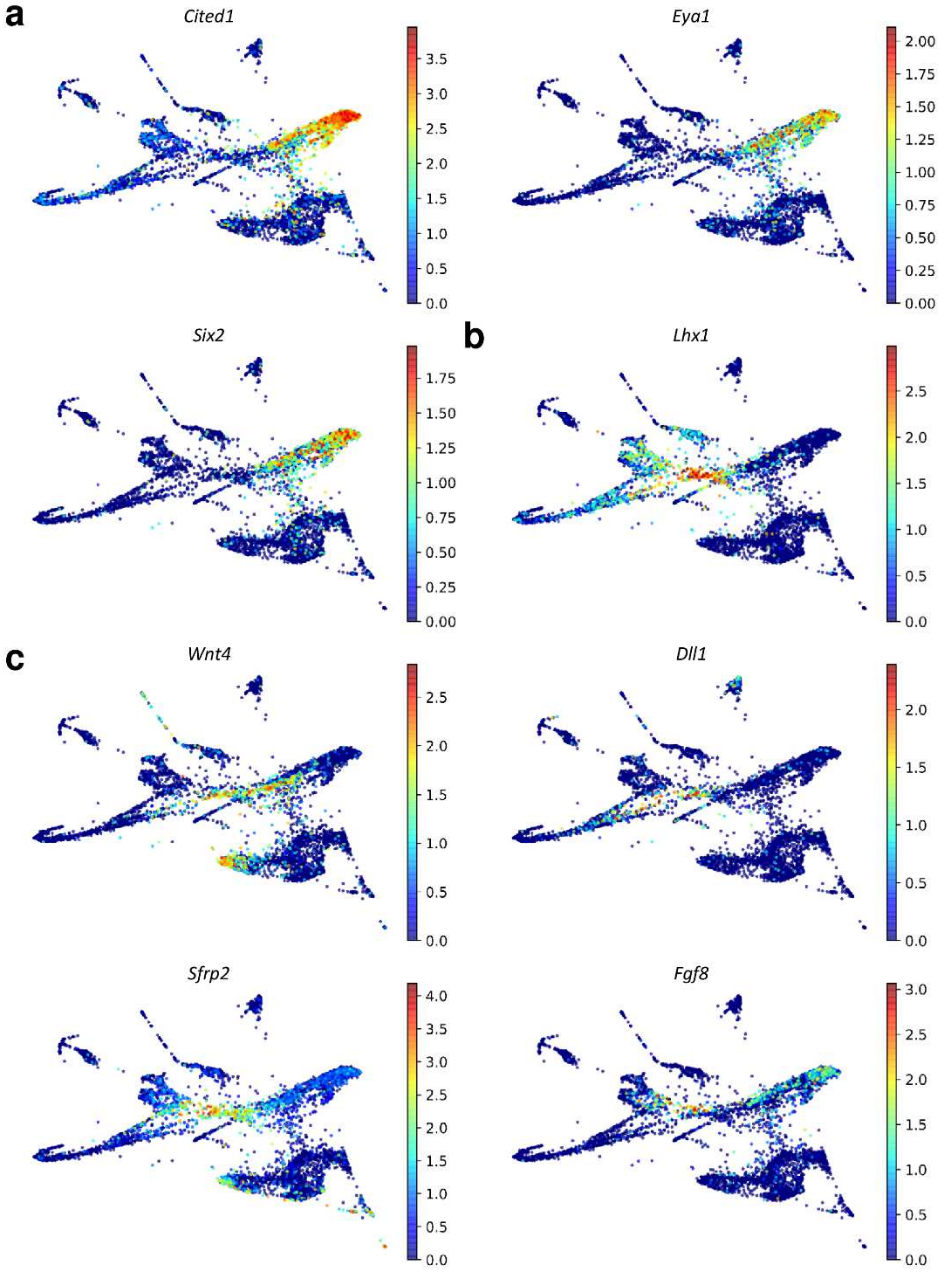

Expression patterns of marker genes for nephron progenitors, comma-, and S-shaped bodies.

Cluster annotation in Fig. 1 was performed based on the following marker genes: (**a**) *Cited1*, *Eya1*, and *Six2* for nephron progenitors; (**b**) *Lhx1*, (**c**) *Wnt4*, *Dll1*, *Sfrp2*, and *Fgf8* for comma- and S-shaped bodies (CS_shape). *Wnt4* was expressed not only in the CS_shape cluster but also in the ureter-associated stroma (ST (ureter)) cluster in accordance with a previous report.[3] All data were analyzed using Scanpy version 1.4.4.post1 (https://scanpy.readthedocs.io/en/stable/).

Supplementary Figure S3-5.

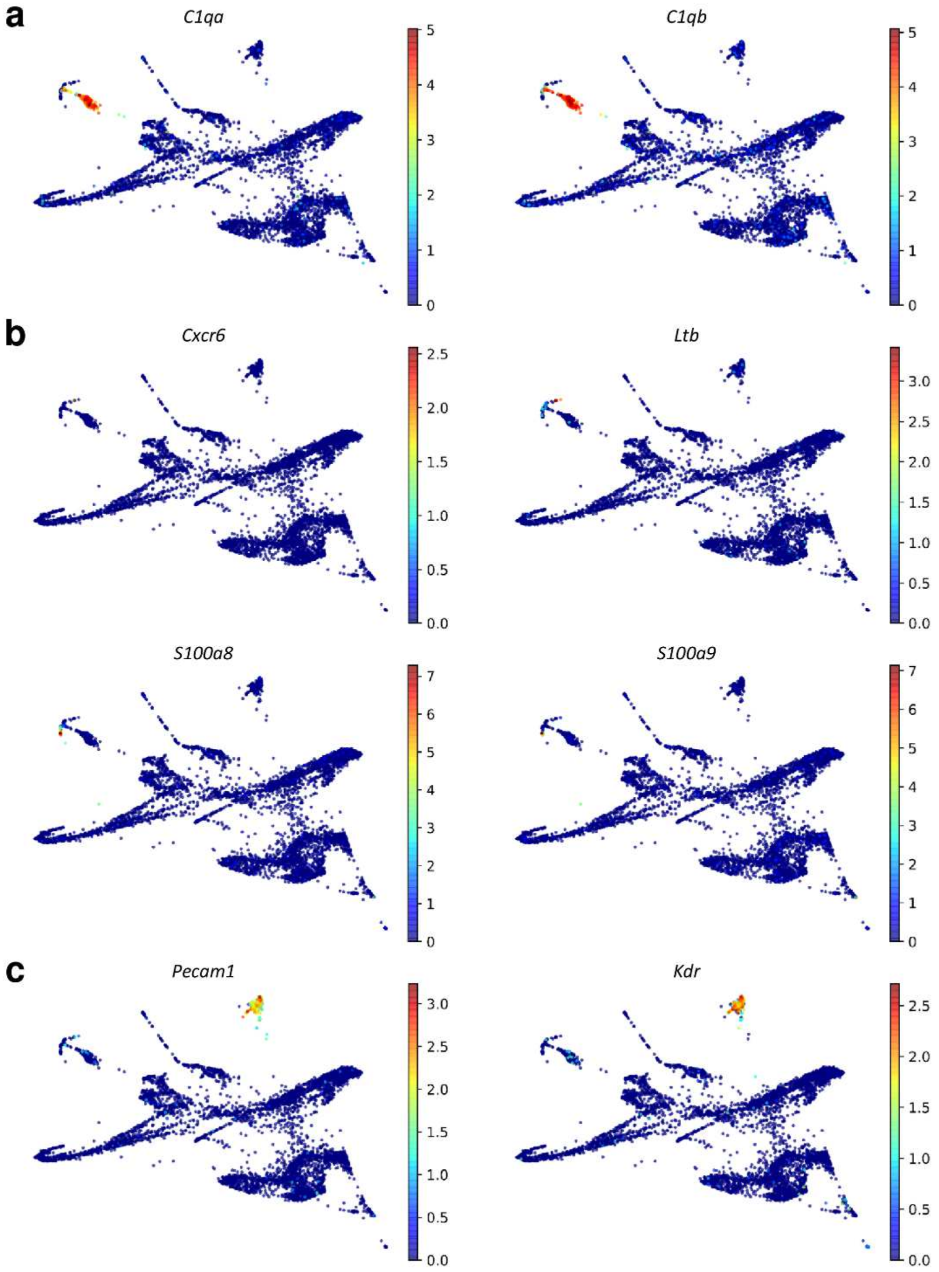

Expression patterns of marker genes for macrophages, immune cells, and endothelial cells.

Cluster annotation in Fig. 1 was performed based on the following marker genes: (**a**) *C1qa* and *C1qb* for macrophages (Mφ); (**b**) *Cxcr6*, *Ltb*, *S100a8*, and *S100a9* for immune cells (Immune); (**c**) *Pecam1* and *Kdr* for endothelial cells (Endo). All data were analyzed using Scanpy version 1.4.4.post1 (https://scanpy.readthedocs.io/en/stable/).

Supplementary Figure S3-6.

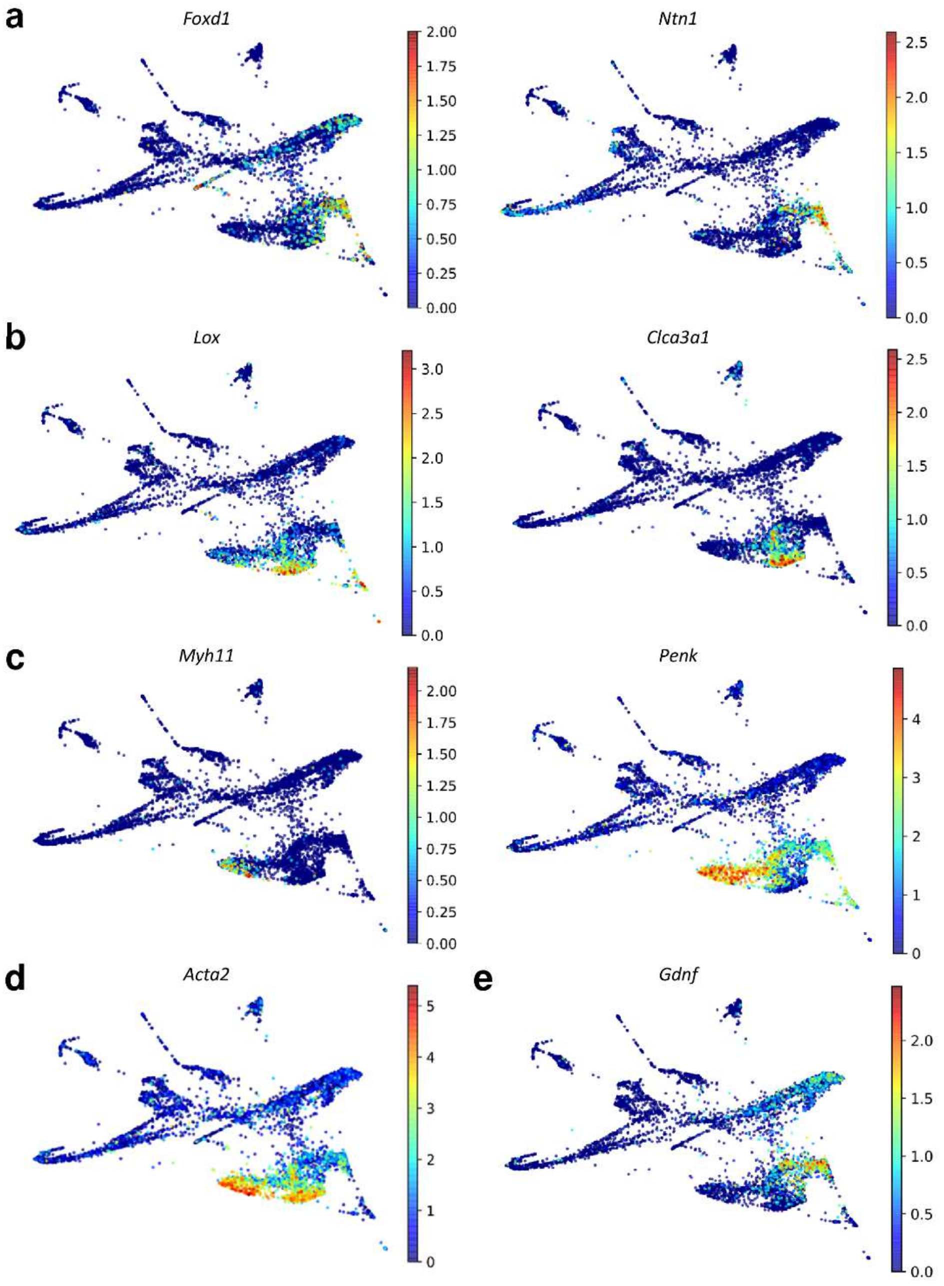

Expression patterns of marker genes for stromal cells.

Cluster annotation in Fig. 1 was performed based on the following marker genes: (**a**) *Foxd1* and *Ntn1* for superficial stroma (ST (sup)); (**b**) *Lox* and *Clca3a1* for medullary stroma (ST (medulla)); (**c**) *Myh11* and *Penk* for ureter associated stroma (ST (ureter)); (**d**) *Acta2* for medullary and ureter associated stroma. (**e**) Expression of *Gdnf* is shown. All data were analyzed using Scanpy version 1.4.4.post1 (https://scanpy.readthedocs.io/en/stable/).

Supplementary Figure S3-7.

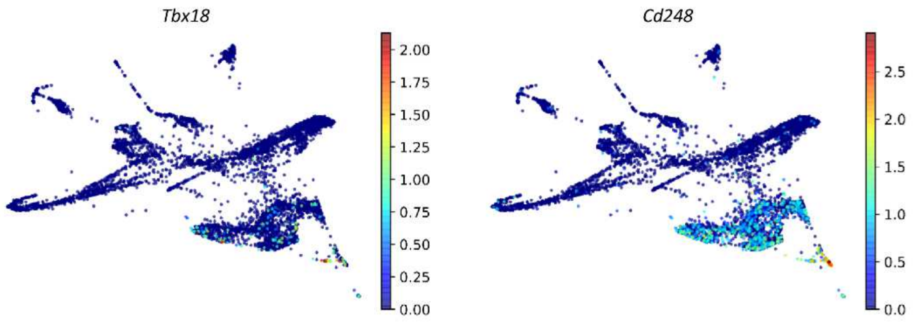

Expression patterns of marker genes for pericytes.

Cluster annotation in Fig. 1 was performed based on the following marker genes: *Tbx18* and *Cd248* for pericytes (Peri). Because Guimaraēs-Camboa *et al*. and Chang-Panesso *et al*. have reported that *Tbx18* and *Cd248* are markers for pericytes, the cluster at the lower right edge of PAGA-initiated ForceAtlas2 map was named as the Peri cluster.[4,5] All data were analyzed using Scanpy version 1.4.4.post1 (https://scanpy.readthedocs.io/en/stable/).

Supplementary Figure S4.

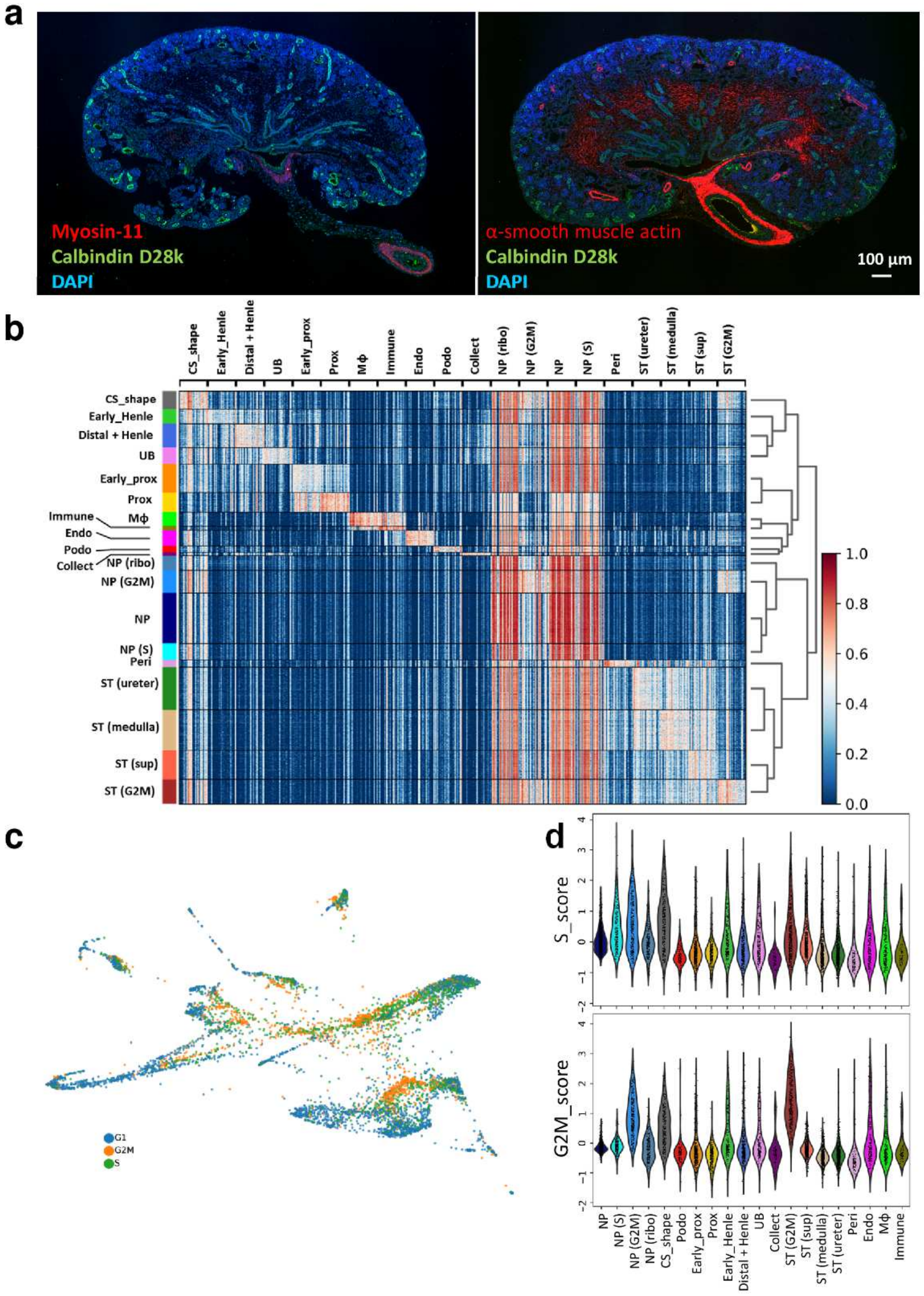

Cluster characterization.

(**a**) Embryonic kidney tissues at day 18.5 were analyzed. Kidney sections were immunohistochemically stained with myosin-11 or α-smooth muscle actin in red, calbindin D28k in green, and 4’,6-diamidino-2-phenylindole (DAPI) in blue (scale bar: 100 µm). Immunofluorescence analysis confirmed that myosin-11 encoded by *Myh11* was expressed in

the ureter associated stromal cells, while α-smooth muscle actin encoded by *Acta2* were expressed in both medullary and ureter associated stromal cells (Supplementary Fig. S3-6**c** and S3-6**d**). (**b**) The Louvain algorithm separated *Cited1*$^+$, *Eya1*$^+$, and *Six2*$^+$ nephron progenitor cells into four clusters to which we named nephron progenitors (NP), S-phase nephron progenitors (NP (S)), G2M-phase nephron progenitors (NP (G2M)), and ribosome-enriched nephron progenitors (NP (ribo)) (Fig. 1 and Supplementary Fig. S3-4**a**). (**b**) For the annotation of these clusters, a heat map showing differentially expressed top 30 genes in each cluster was drawn. The NP, NP (S), and NP (ribo) clusters had similar gene expression pattern. The names of differently expressed genes are summarized in Supplementary Table S1-1. Differentially expressed genes were extracted using Scanpy (Wilcoxon rank-sum test). (**c**) Cell cycle phase scores were visualized in order to clarify the difference among the NP, NP (S), and NP (ribo) clusters. Each cell was assigned cell cycle score based on its expression of S and G2M phase markers.[6] (**d**) Cell cycle phase scores were visualized by violin plots. S phase score was high not only in the NP (S) cluster, but also in the NP (G2M) cluster. The name of NP (ribo) cluster was derived from the enriched expression levels of ribosomal RNAs, *Rps23*, *Rps27a* and *Rpl32* (Supplementary Table S1-1). The ST (G2M) cluster was named from the observation that the cells in this cluster belonged to stromal cells in the hierarchical clustering and had high G2M score. All data were analyzed using Scanpy version 1.4.4.post1 (https://scanpy.readthedocs.io/en/stable/).
