# Supplementary material for: Single cell RNA sequencing uncovers cellular developmental sequences and novel potential intercellular communications in embryonic kidney
Source: Sci Rep. 2021 Jan 8;11:73. doi: 10.1038/s41598-020-80154-y (PMC7794461; doi:10.1038/s41598-020-80154-y)

Supplementary Figure S5-1.

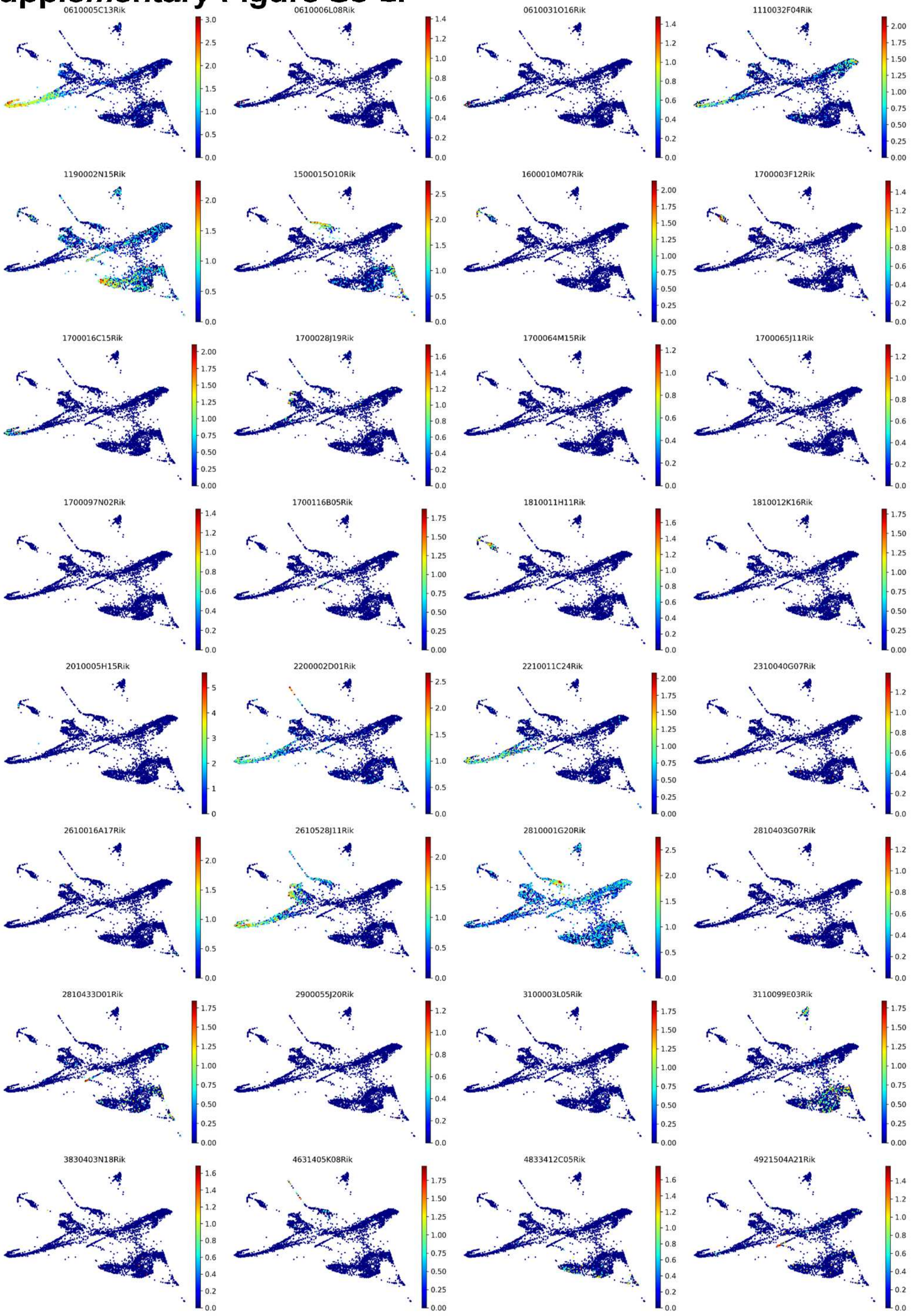

Supplementary Figure S5-2.

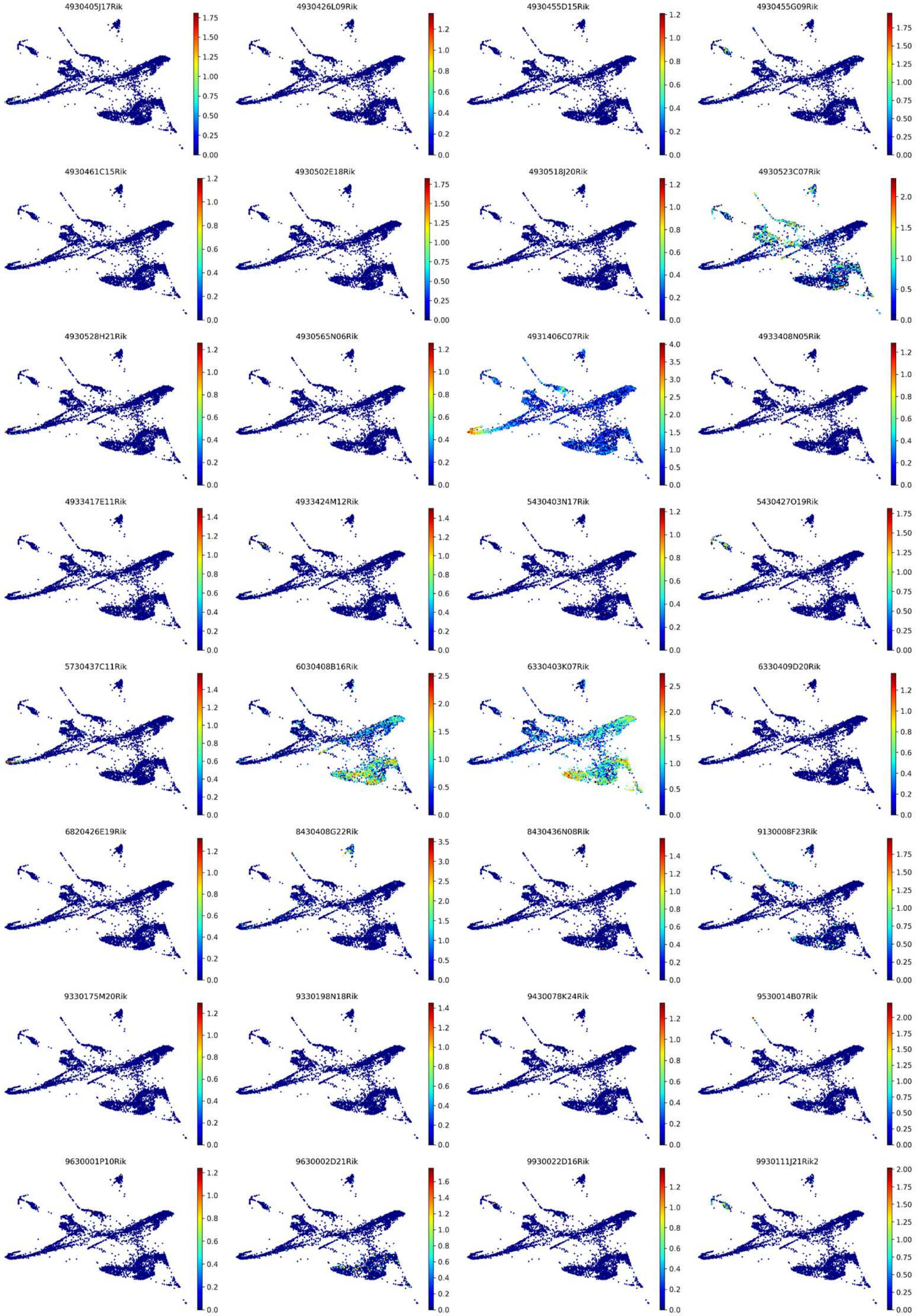

Supplementary Figure S5-3.

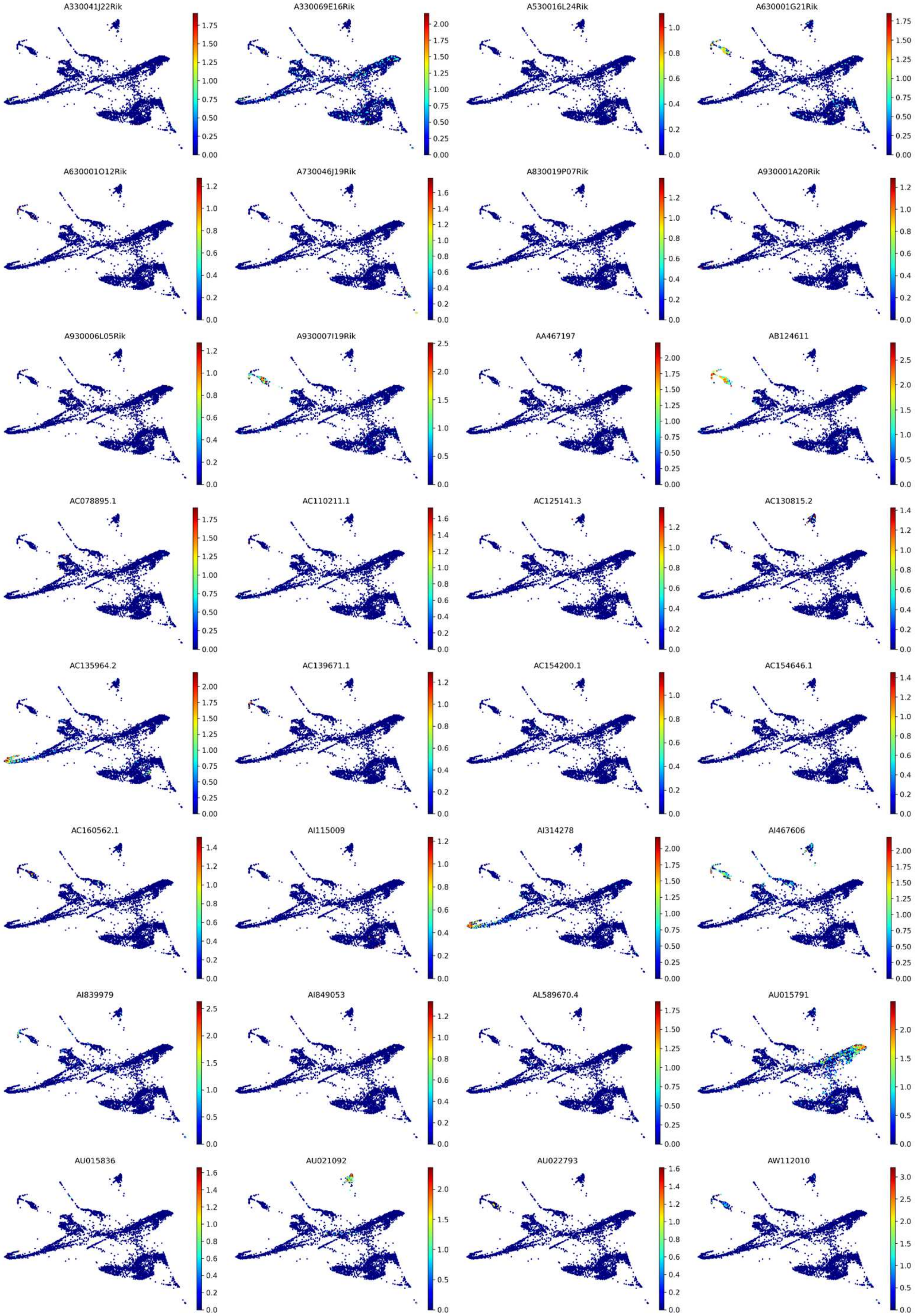

Supplementary Figure S5-4.

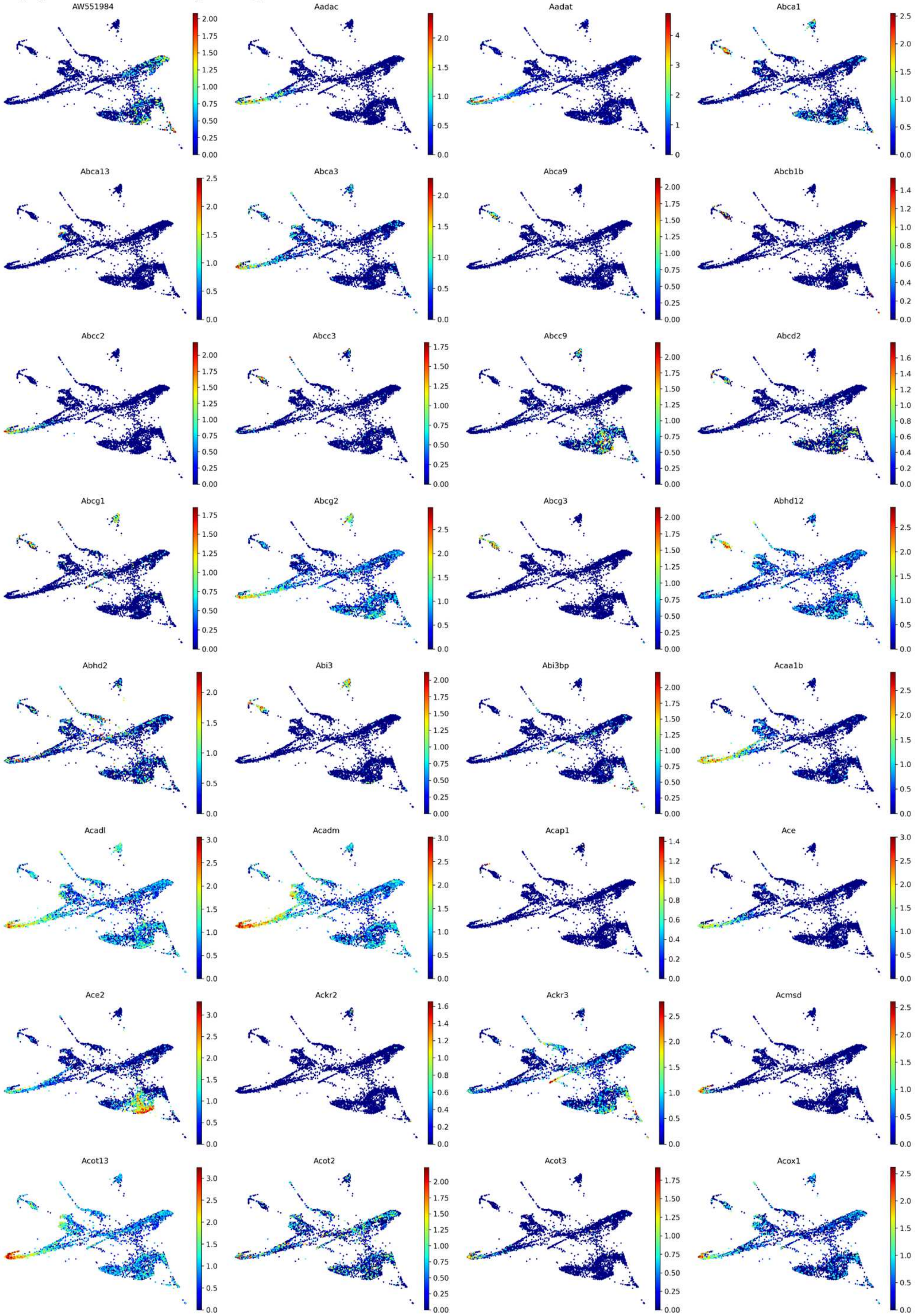

Supplementary Figure S5-5.

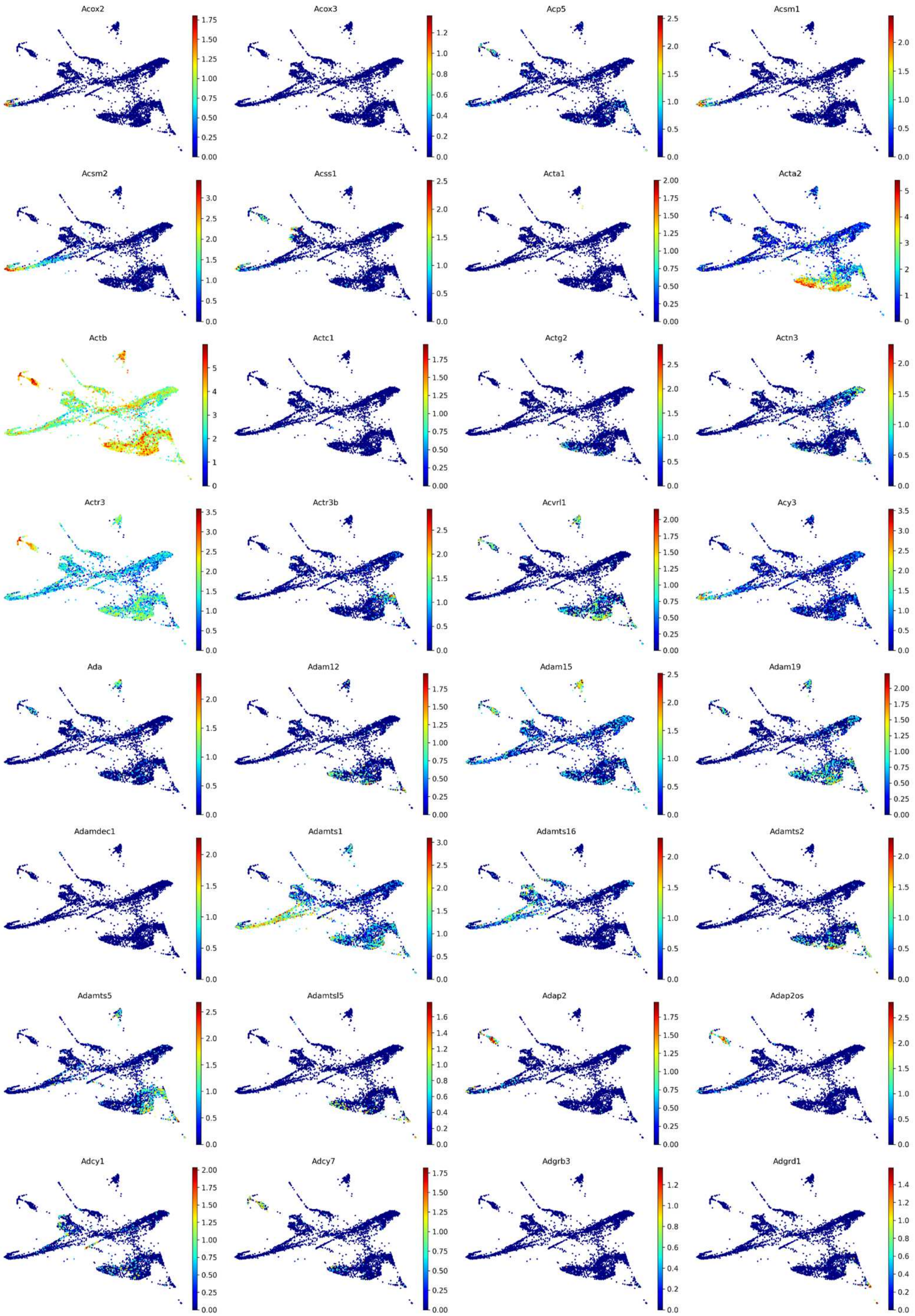

Supplementary Figure S5-6.

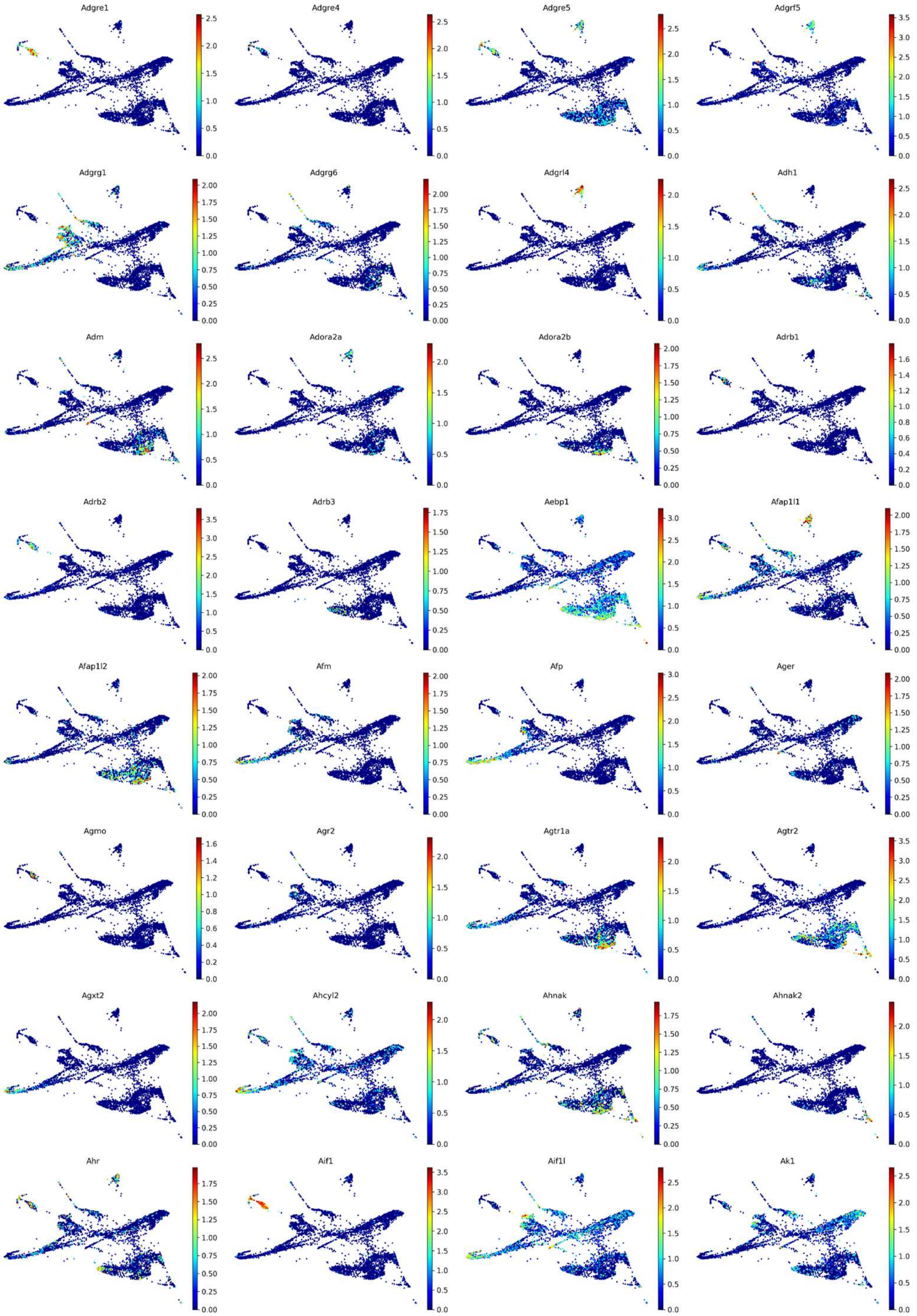

Supplementary Figure S5-7.

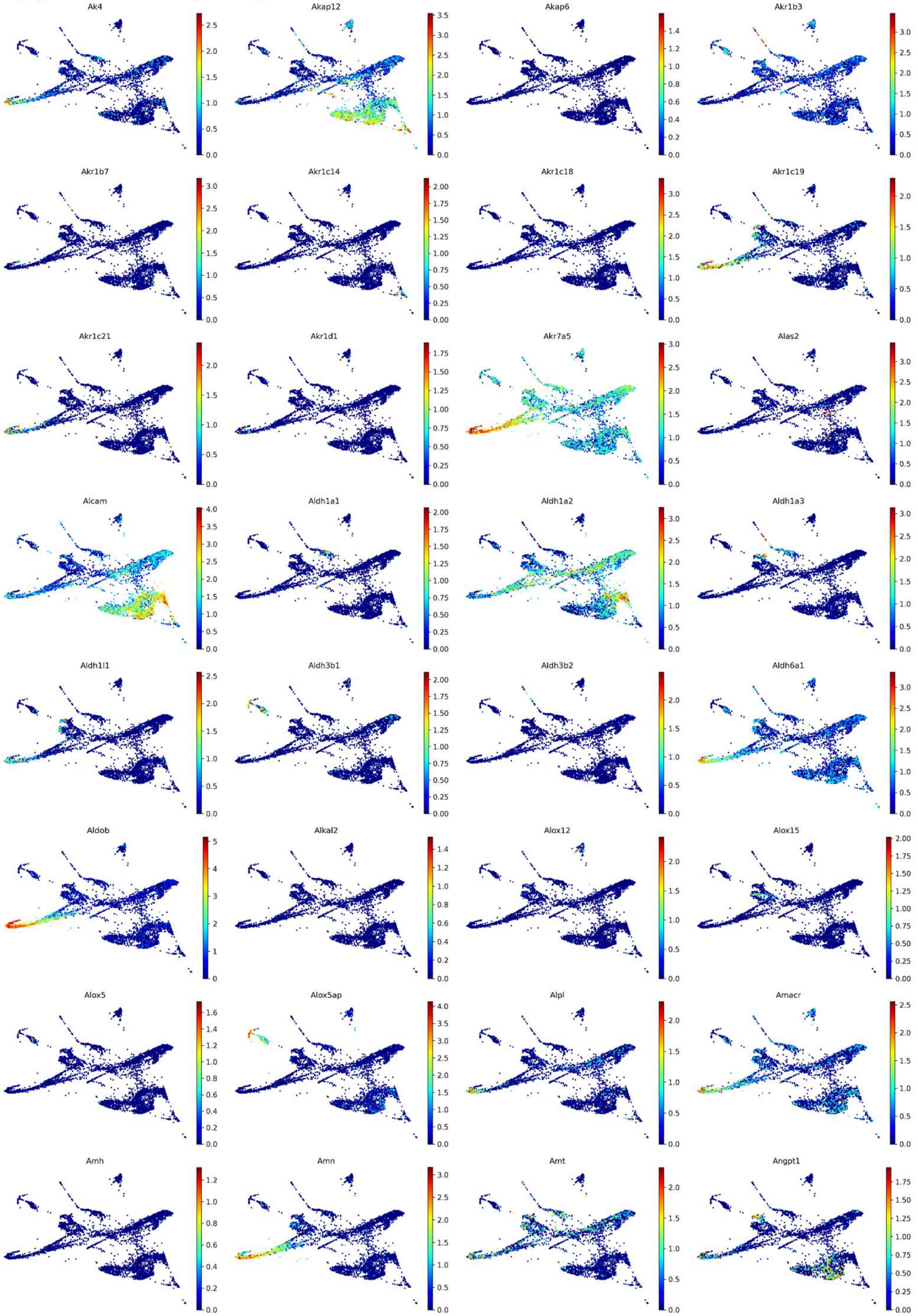

Supplementary Figure S5-8.

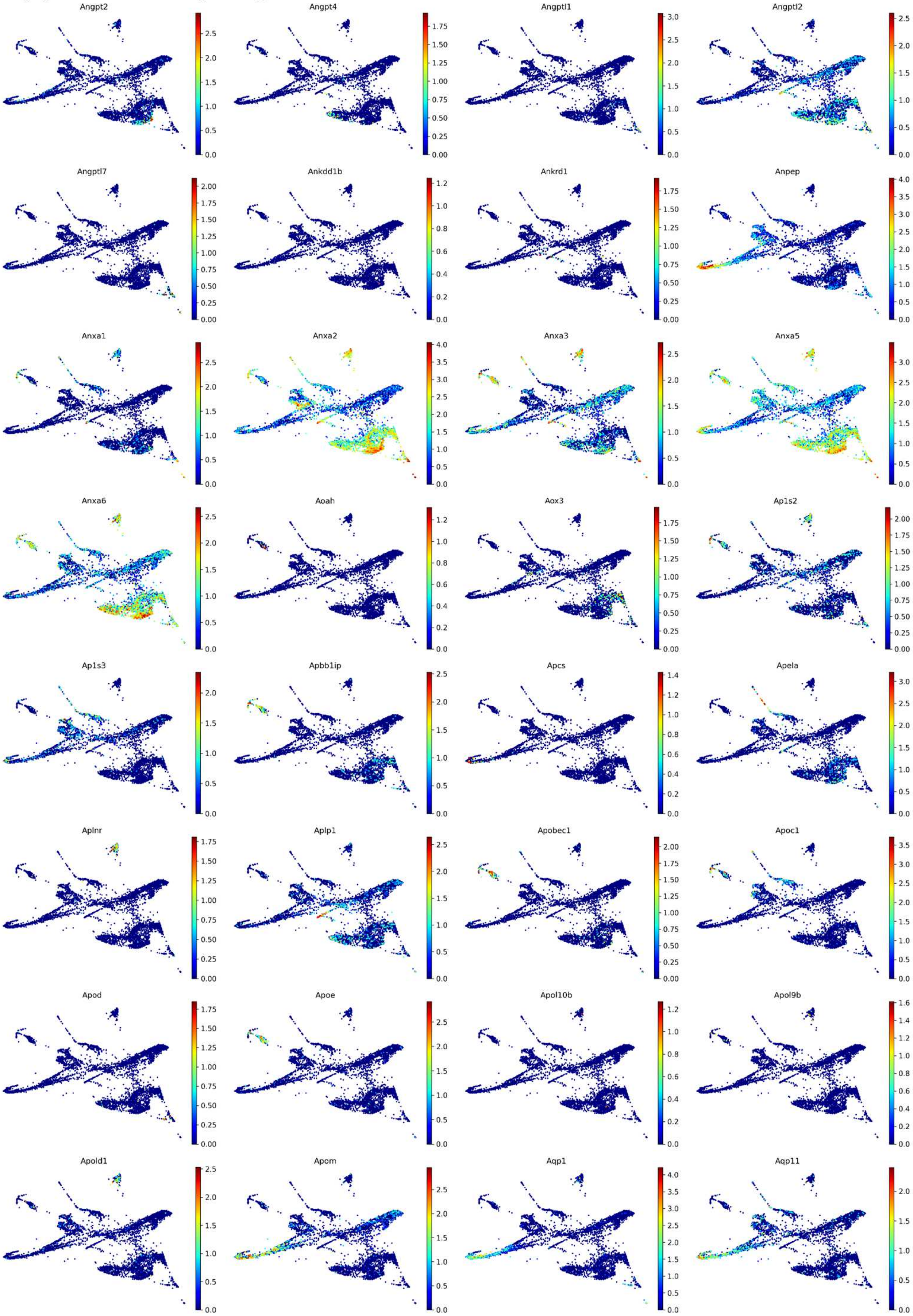

Supplementary Figure S5-9.

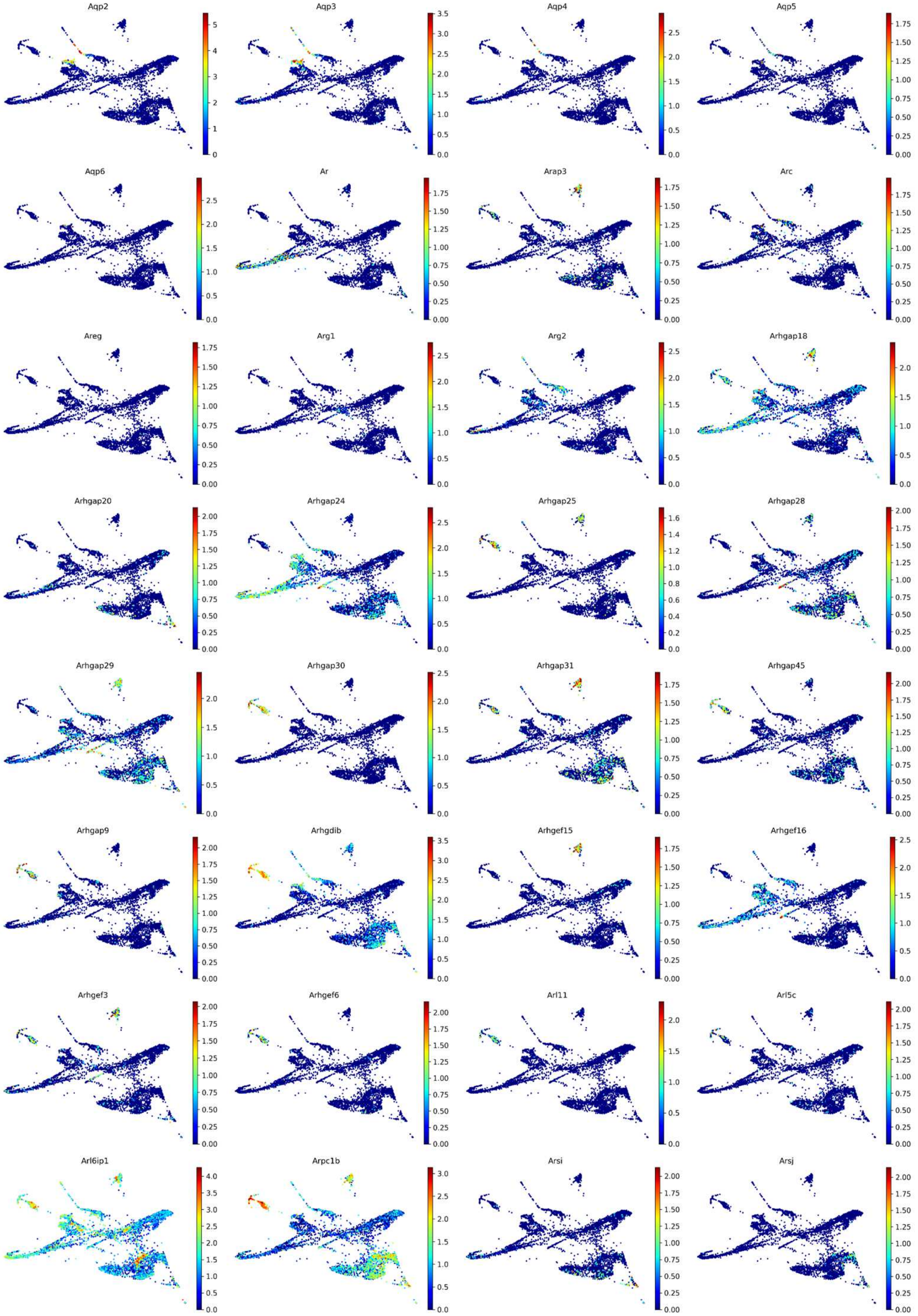

Supplementary Figure S5-10.

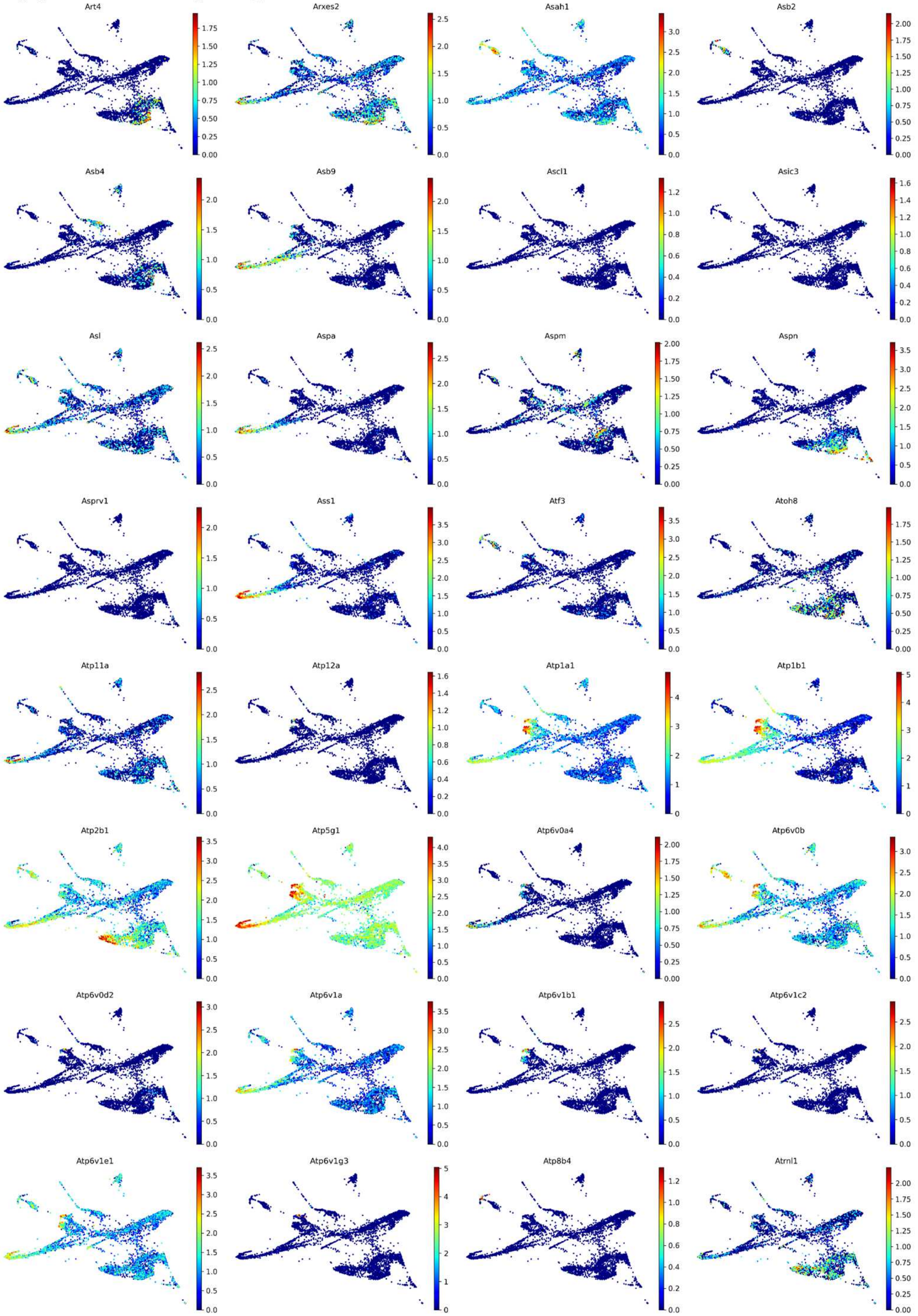

Supplementary Figure S5-11.

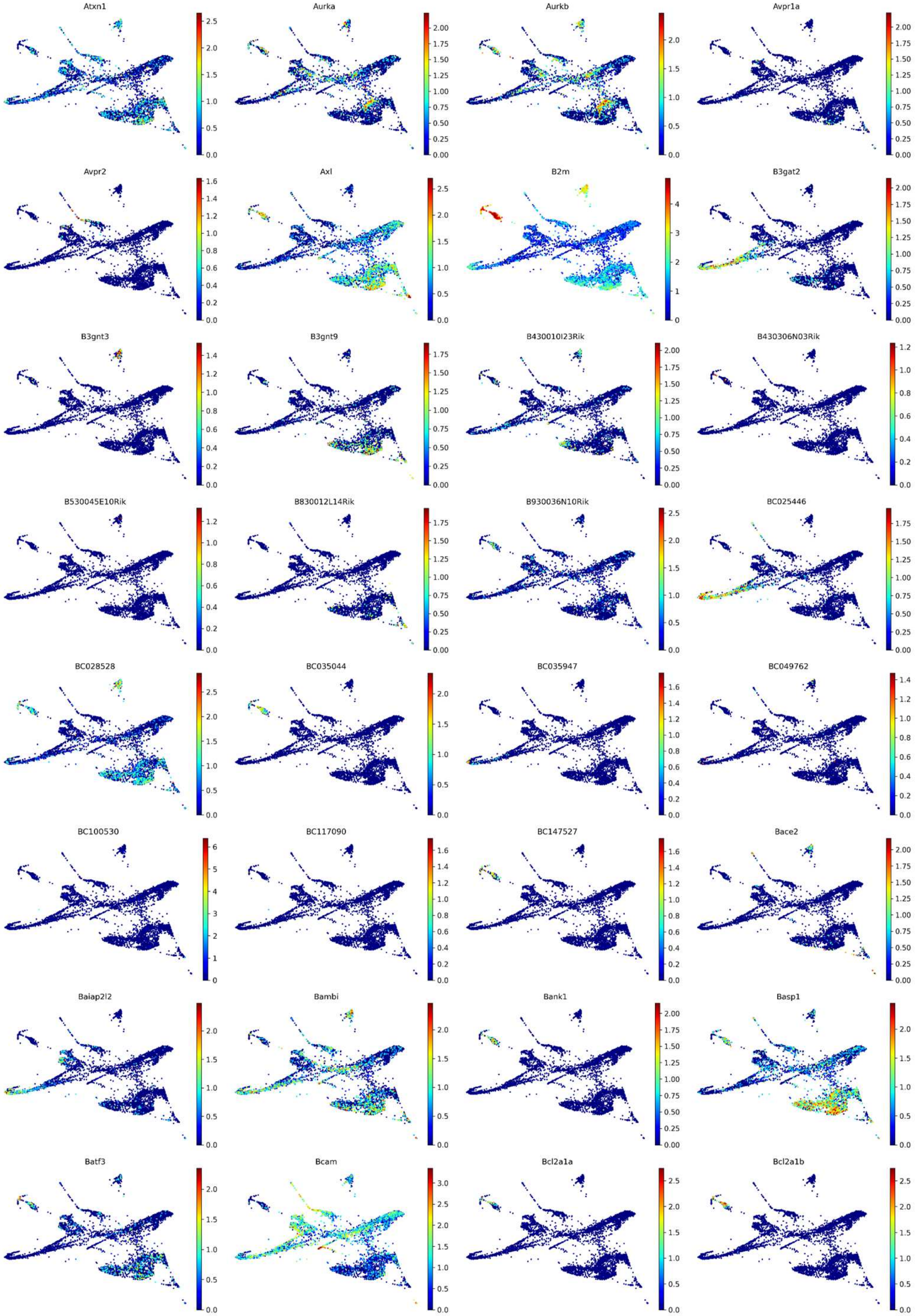

Supplementary Figure S5-12.

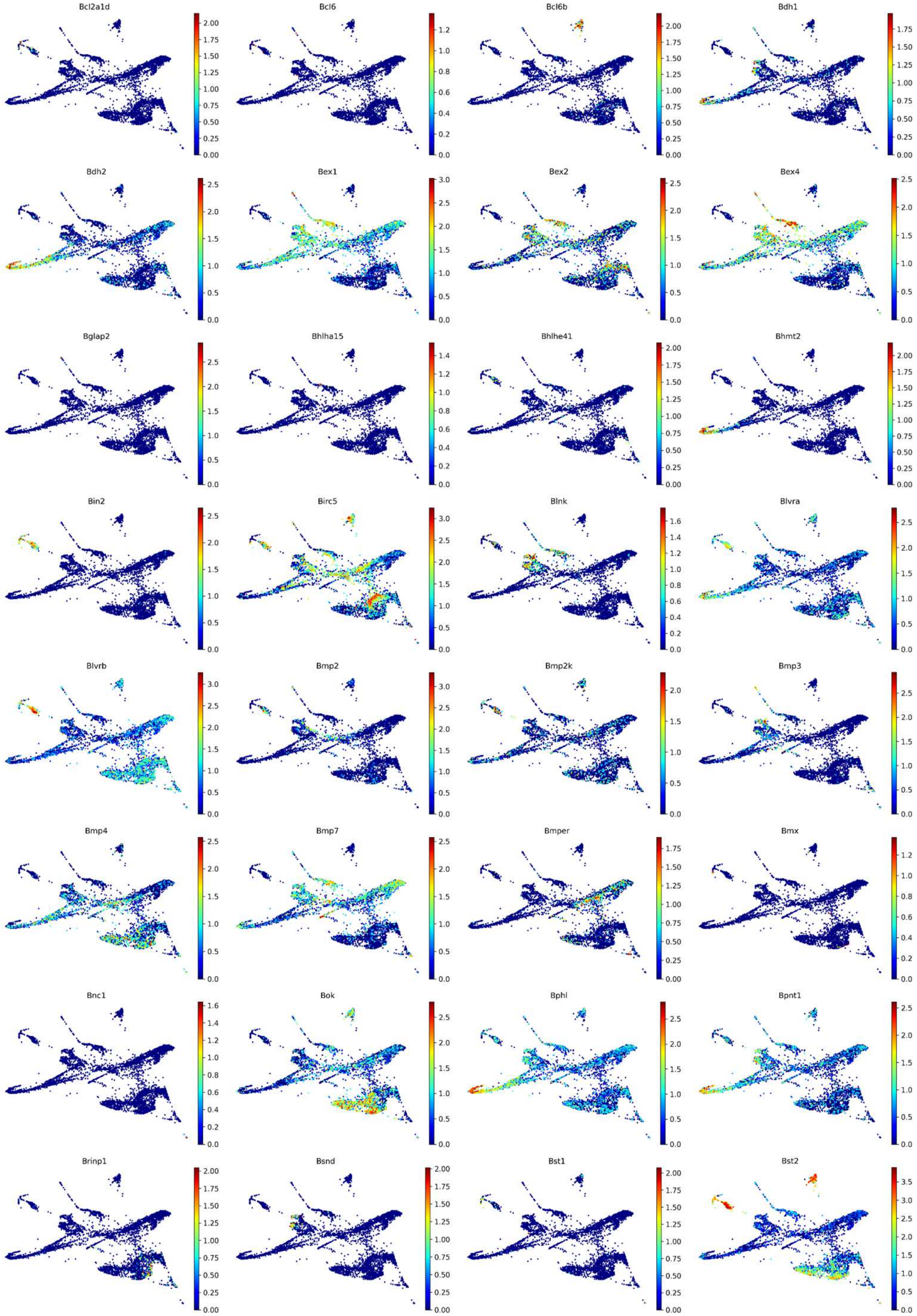

Supplementary Figure S5-13.

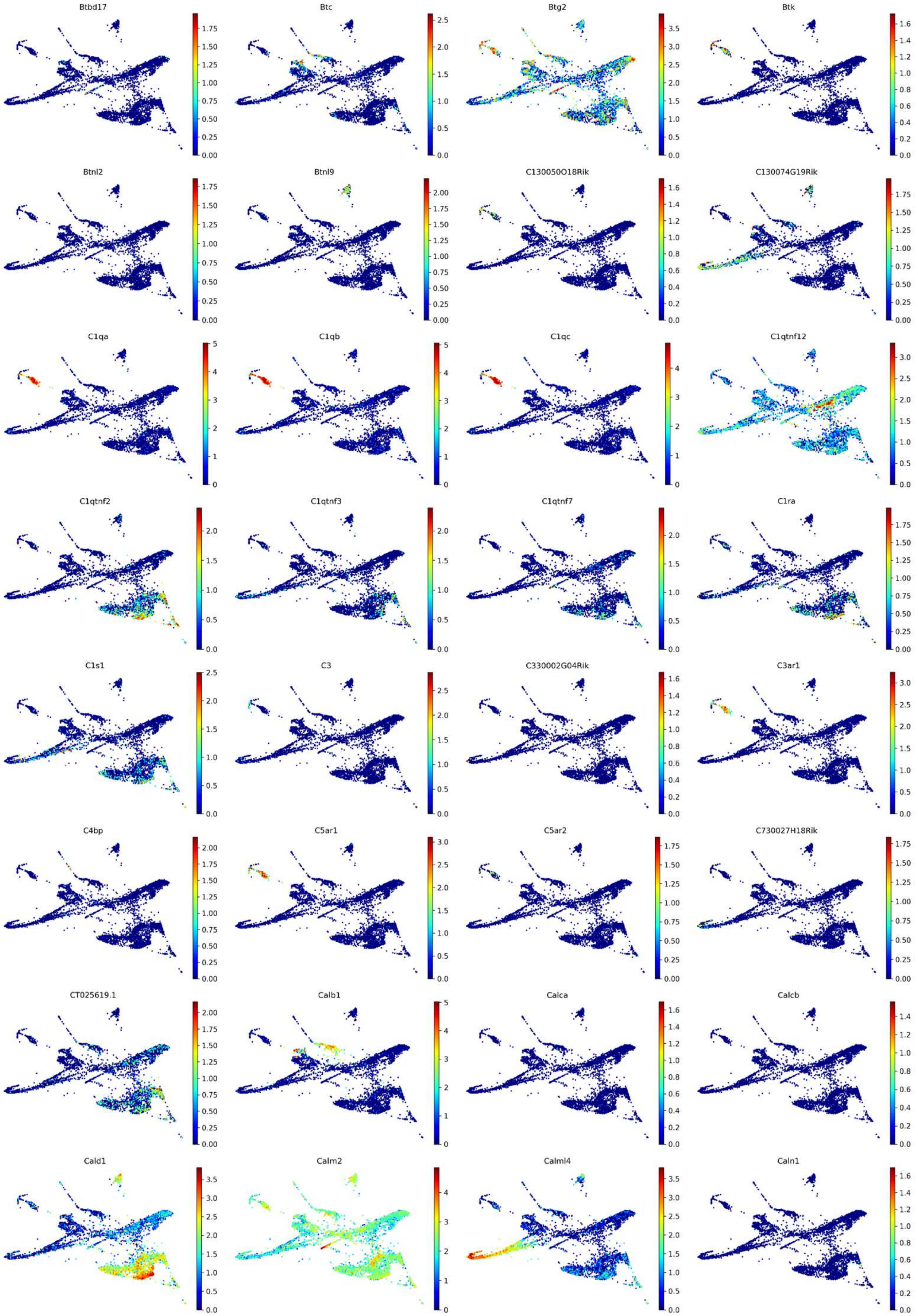

Supplementary Figure S5-14.

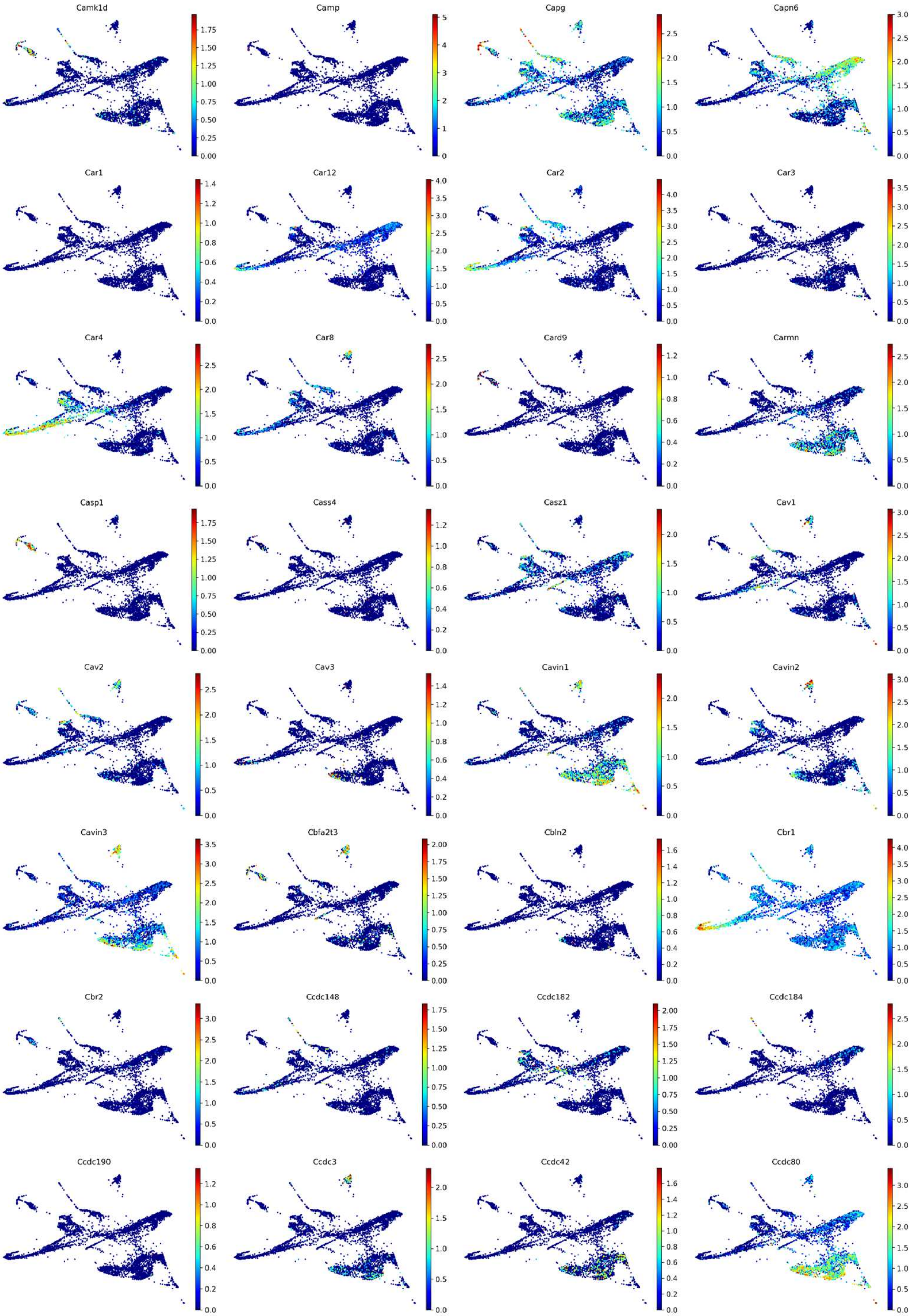

Supplementary Figure S5-15.

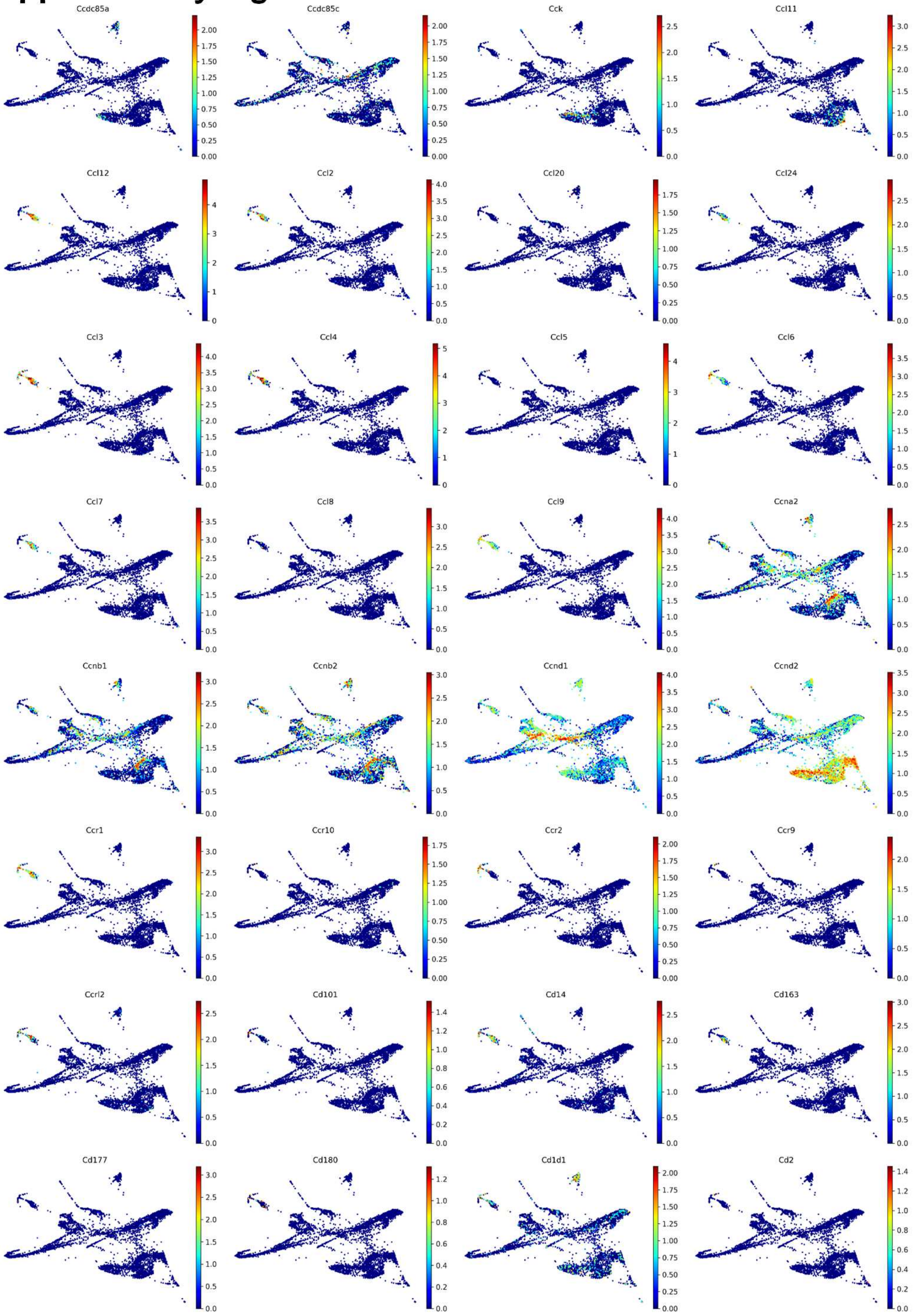

Supplementary Figure S5-16.

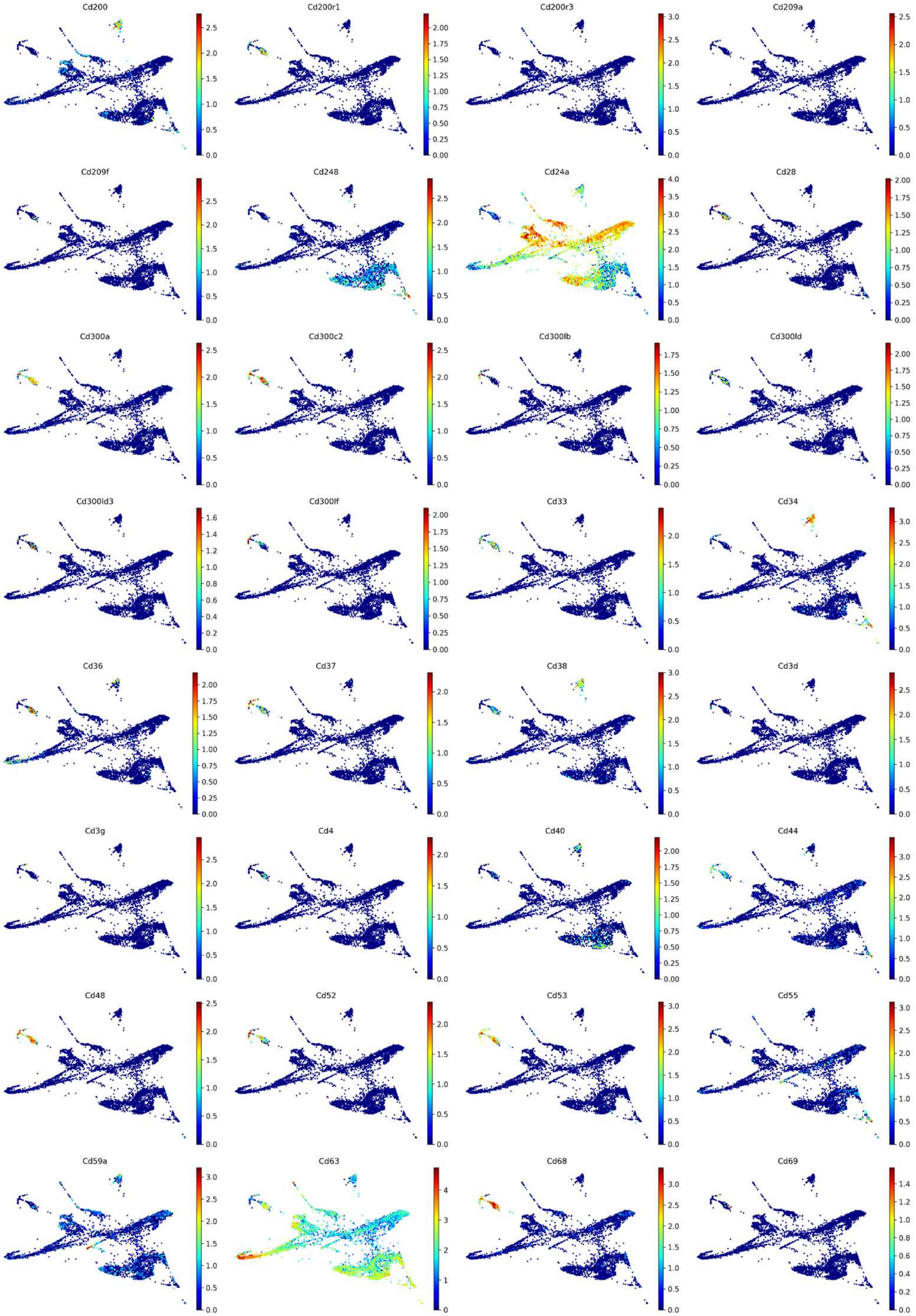

Supplementary Figure S5-17.

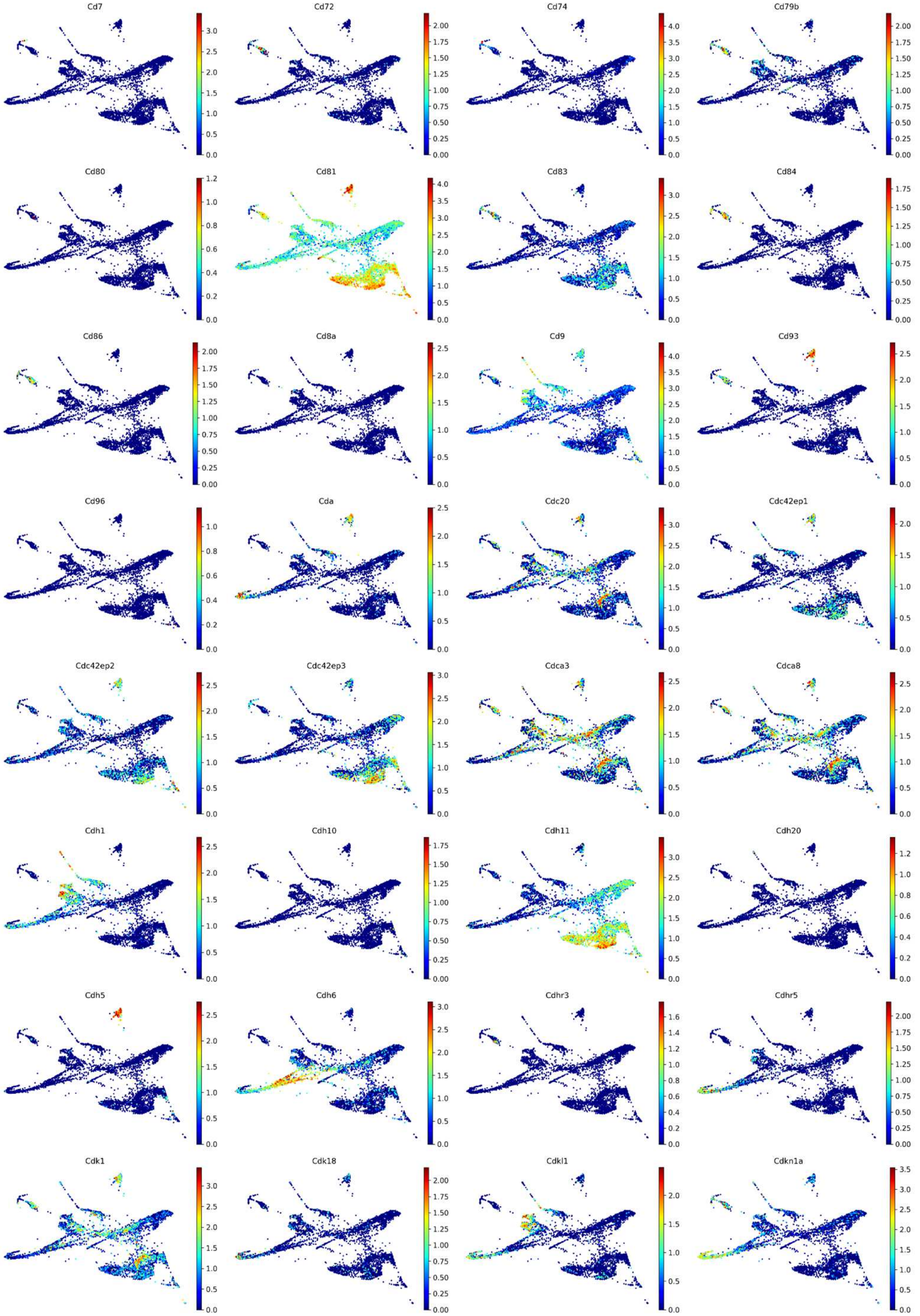

Supplementary Figure S5-18.

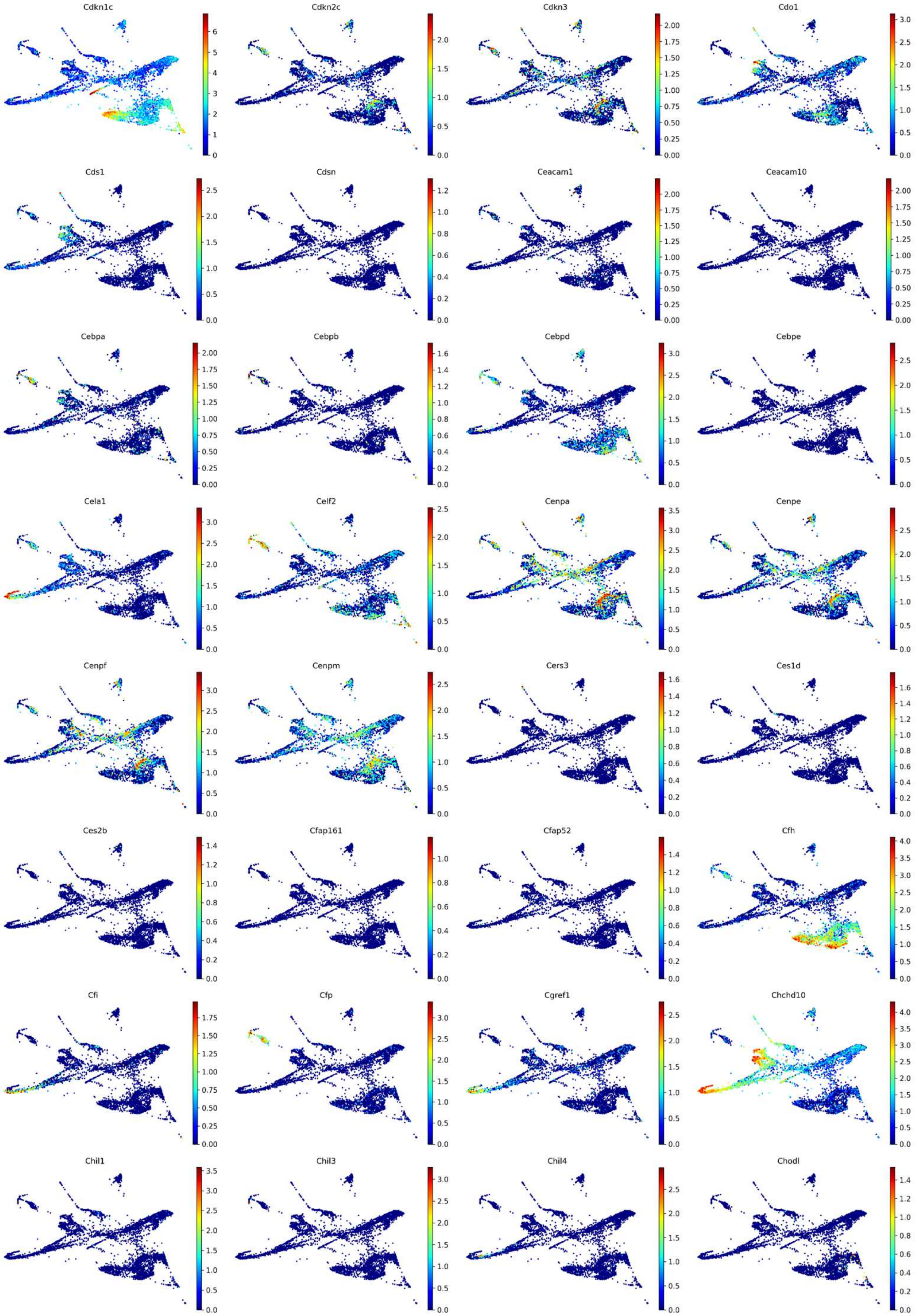

Supplementary Figure S5-19.

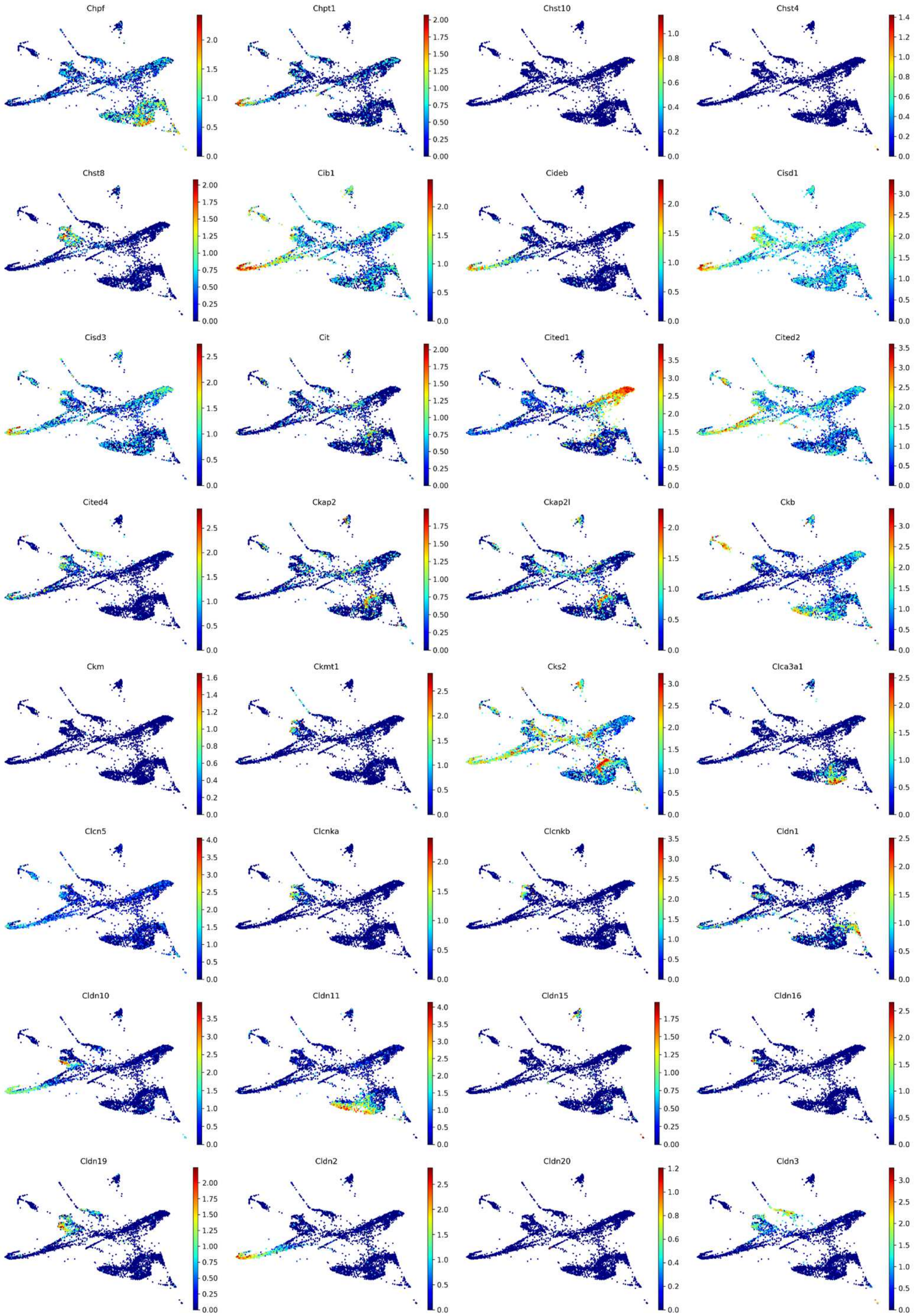

Supplementary Figure S5-20.

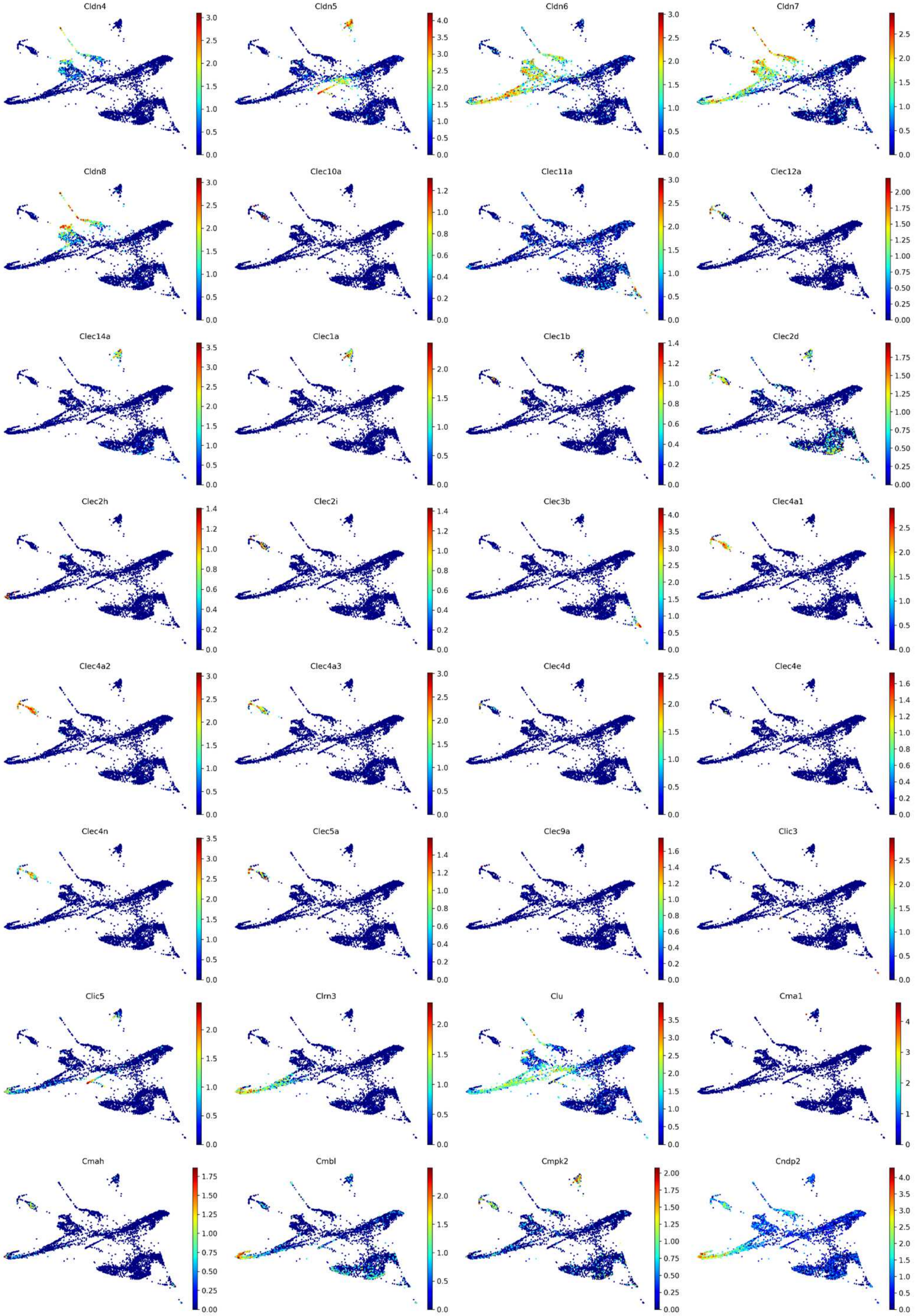

Supplementary Figure S5-21.

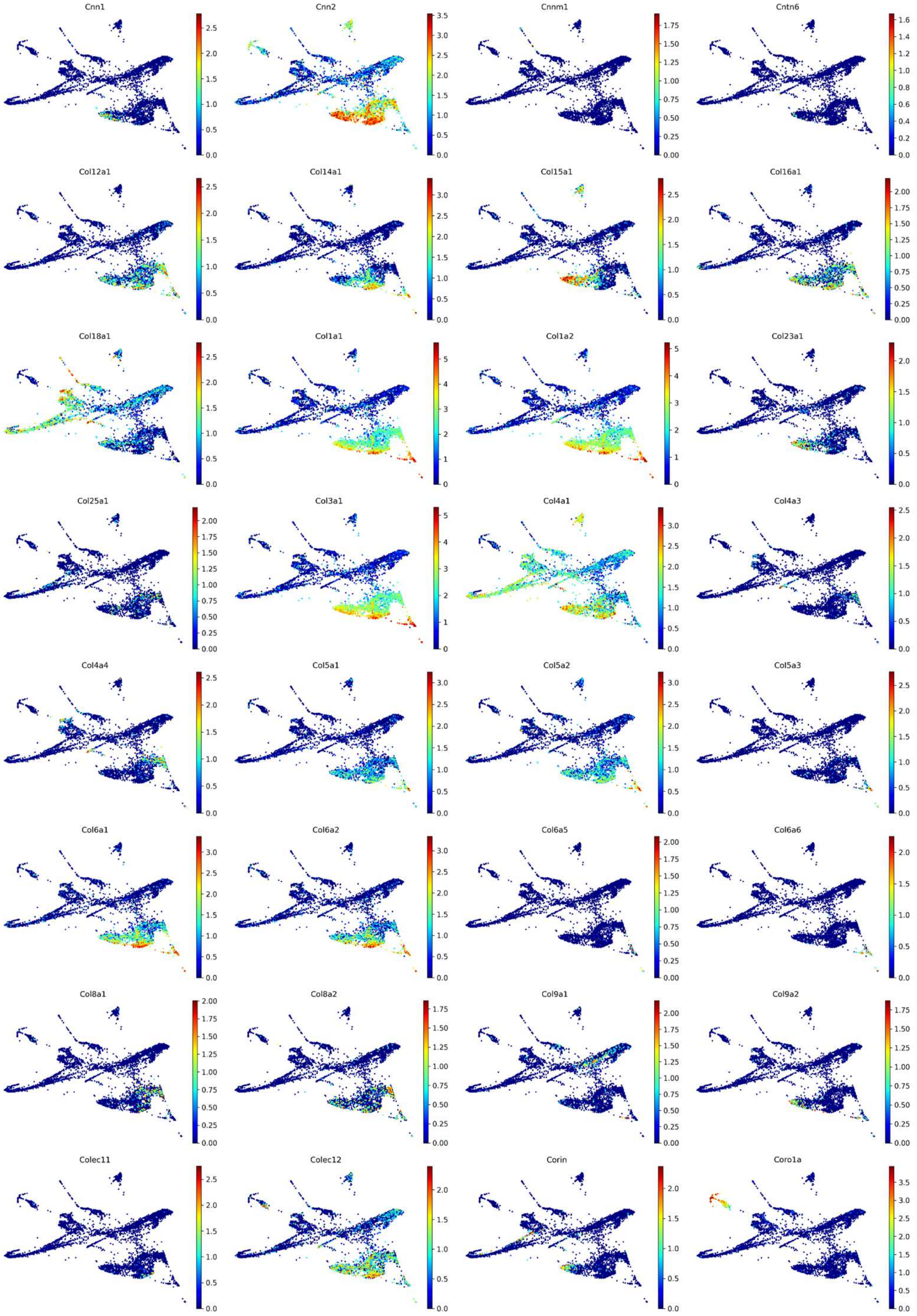

Supplementary Figure S5-22.

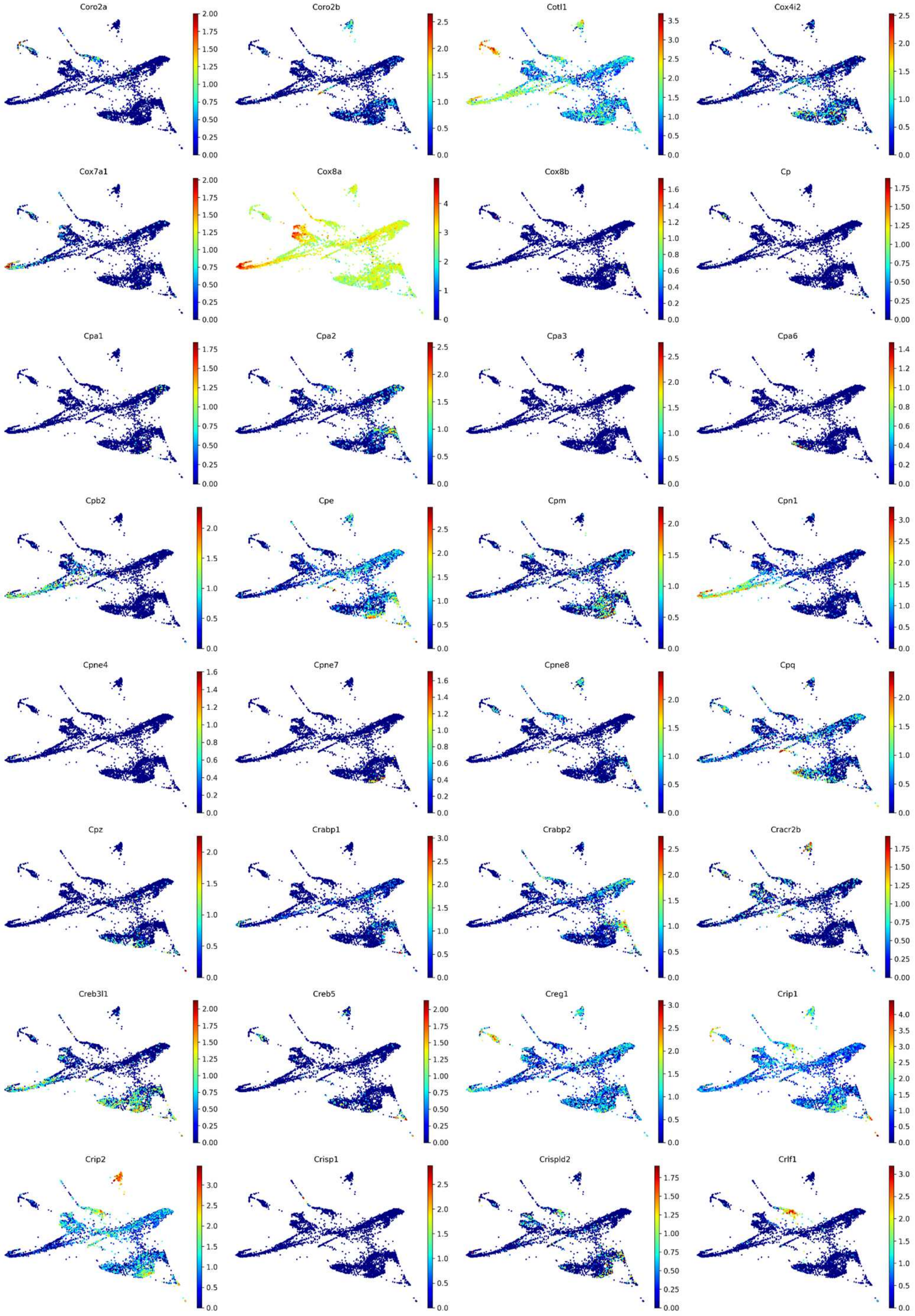

Supplementary Figure S5-23.

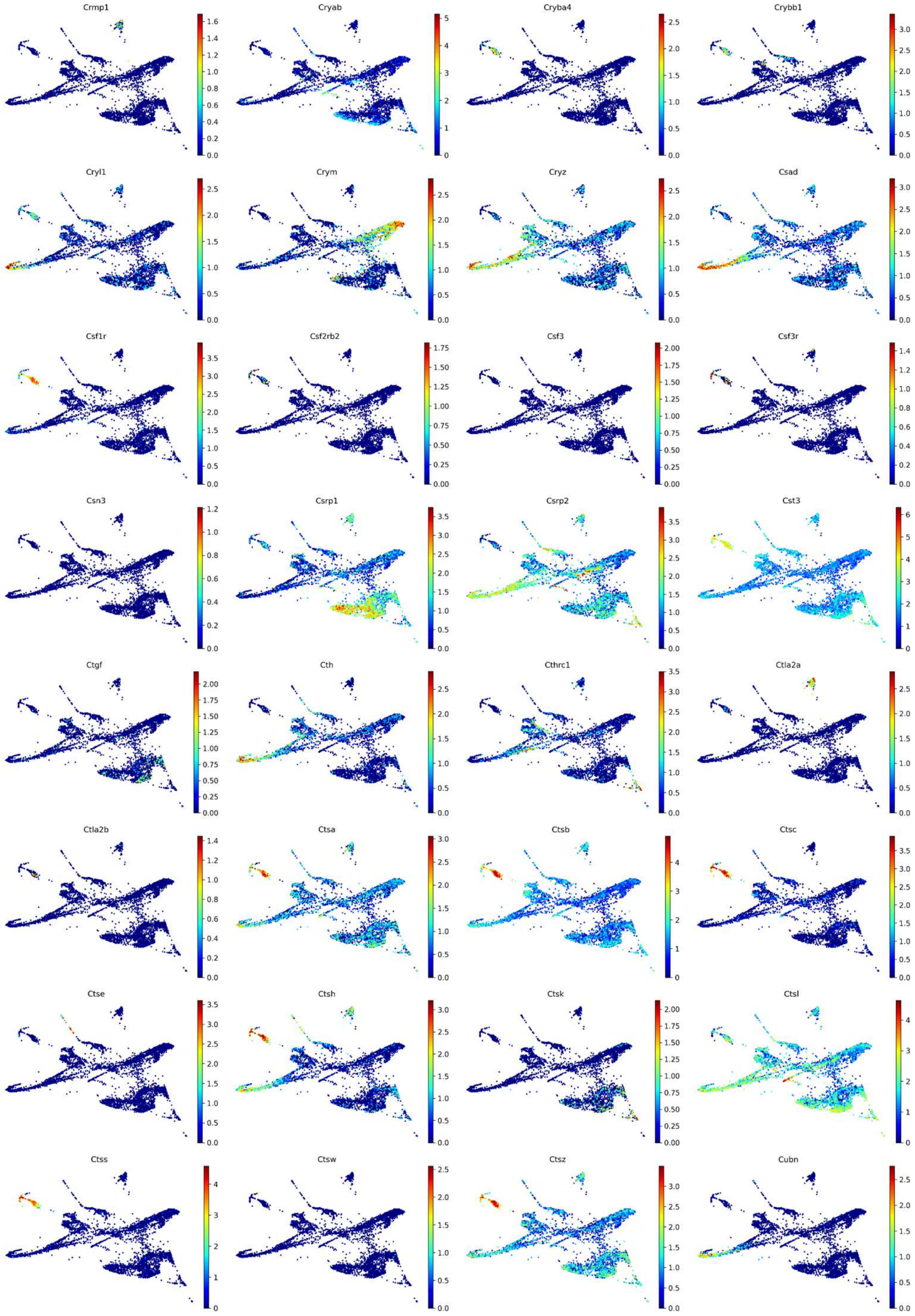

Supplementary Figure S5-24.

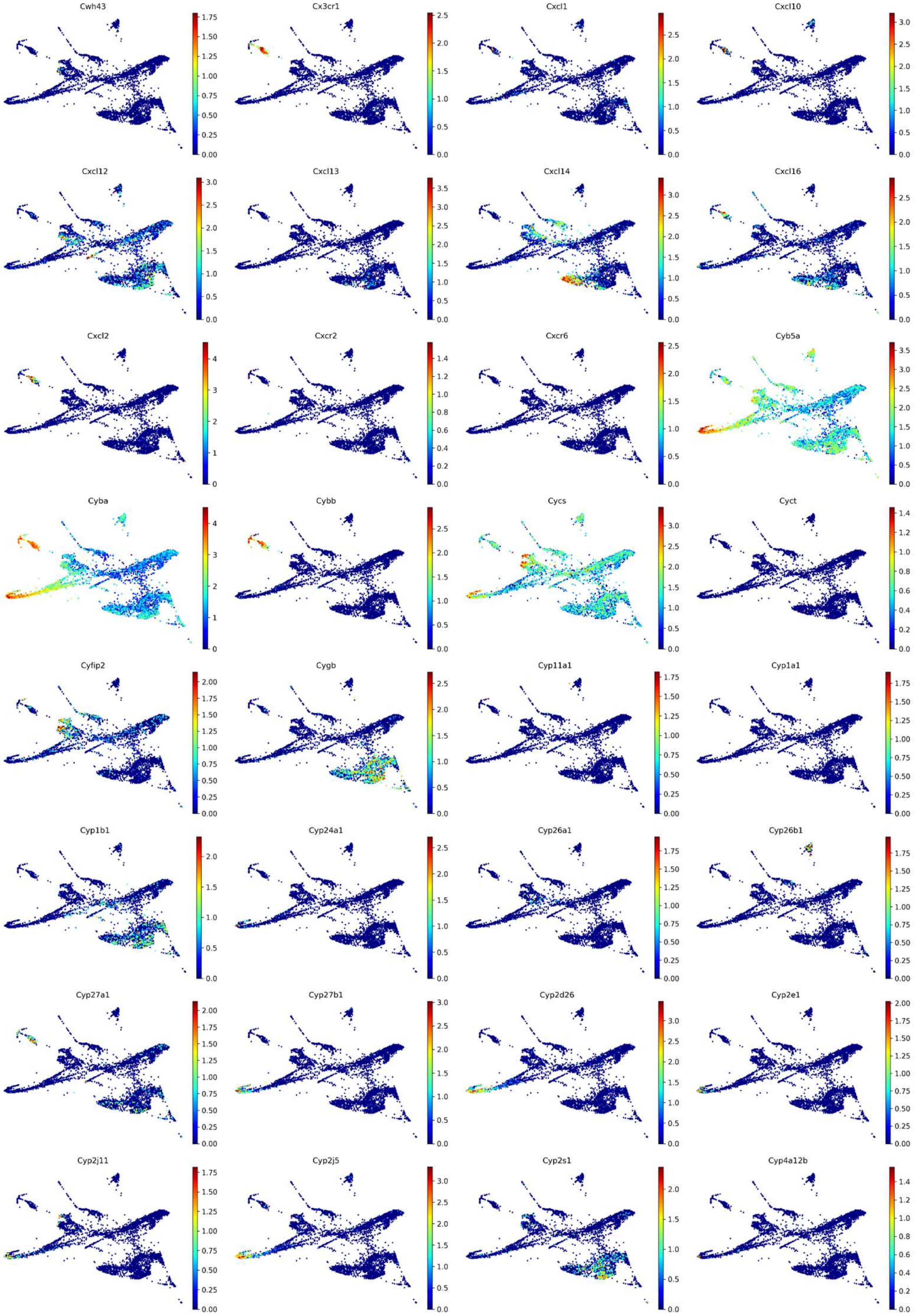

Supplementary Figure S5-25.

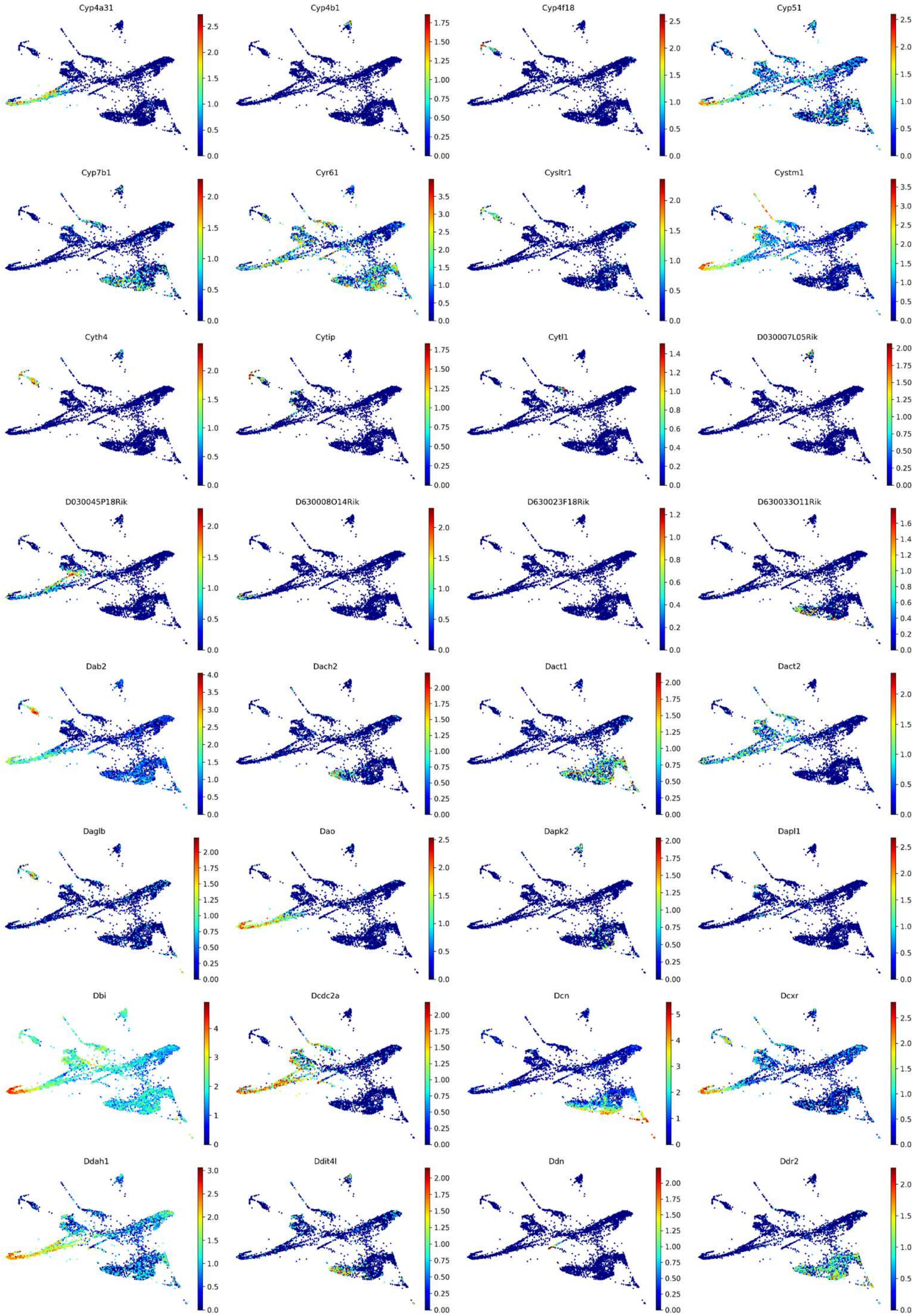

Supplementary Figure S5-26.

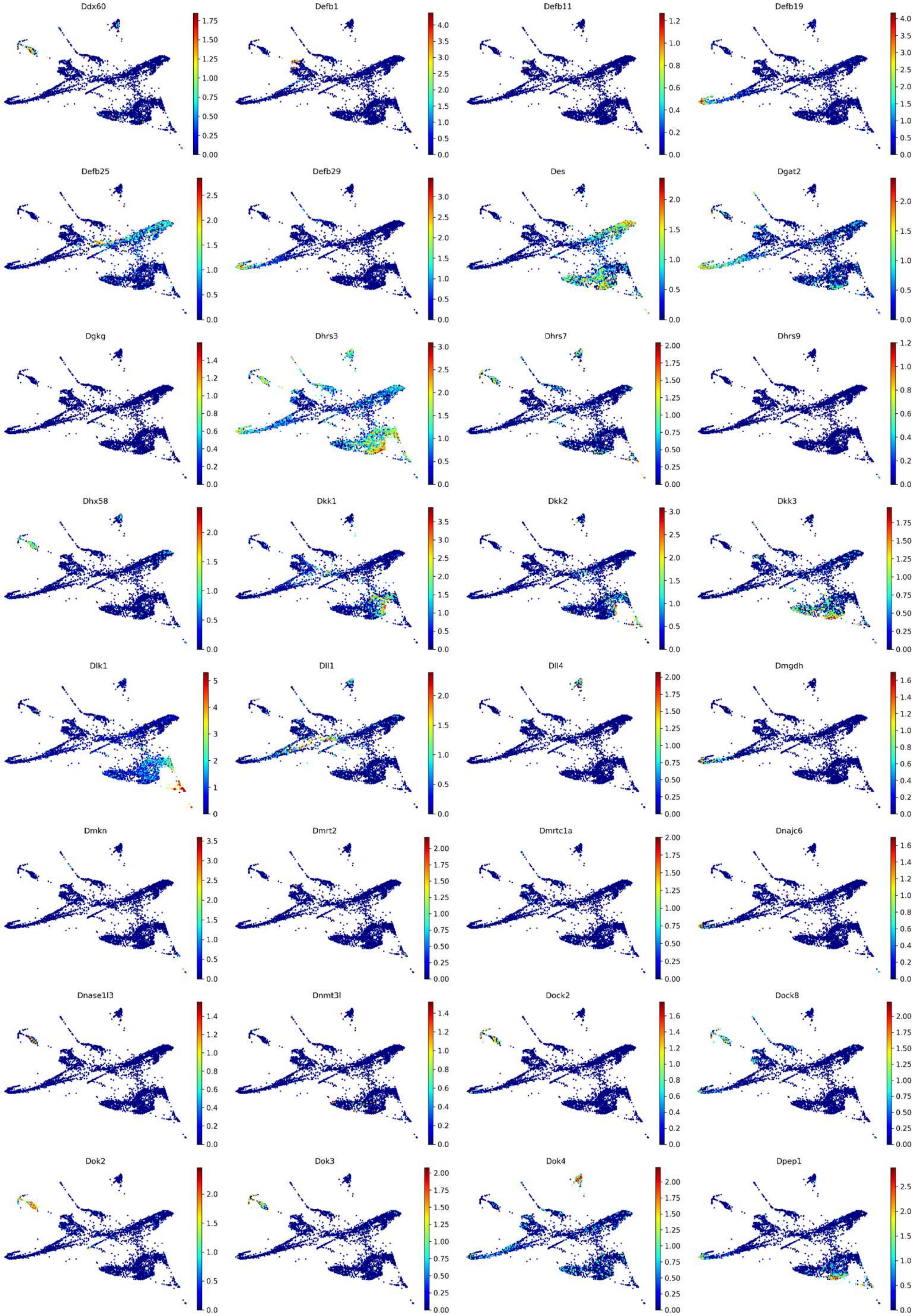

Supplementary Figure S5-27.

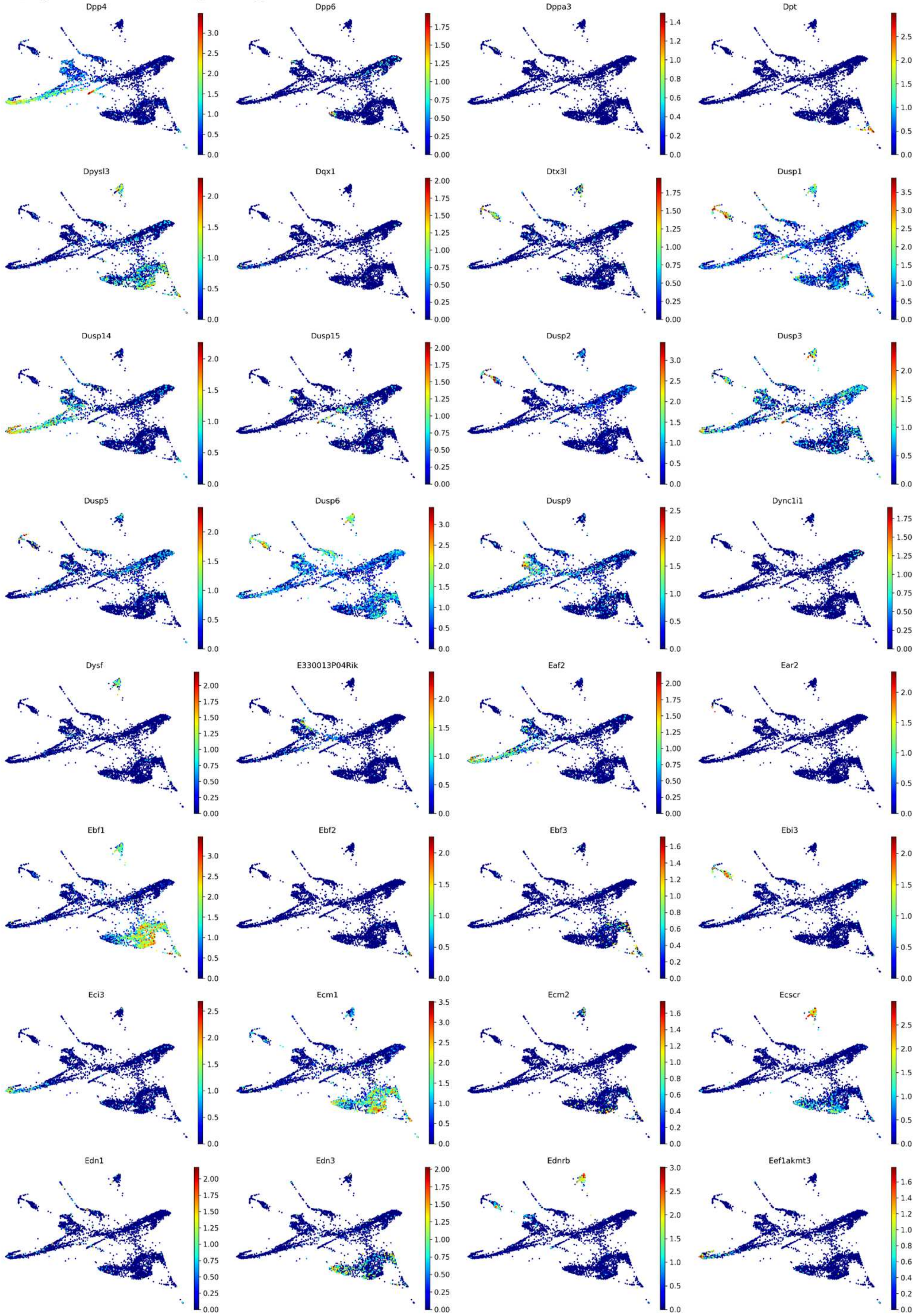

Supplementary Figure S5-28.

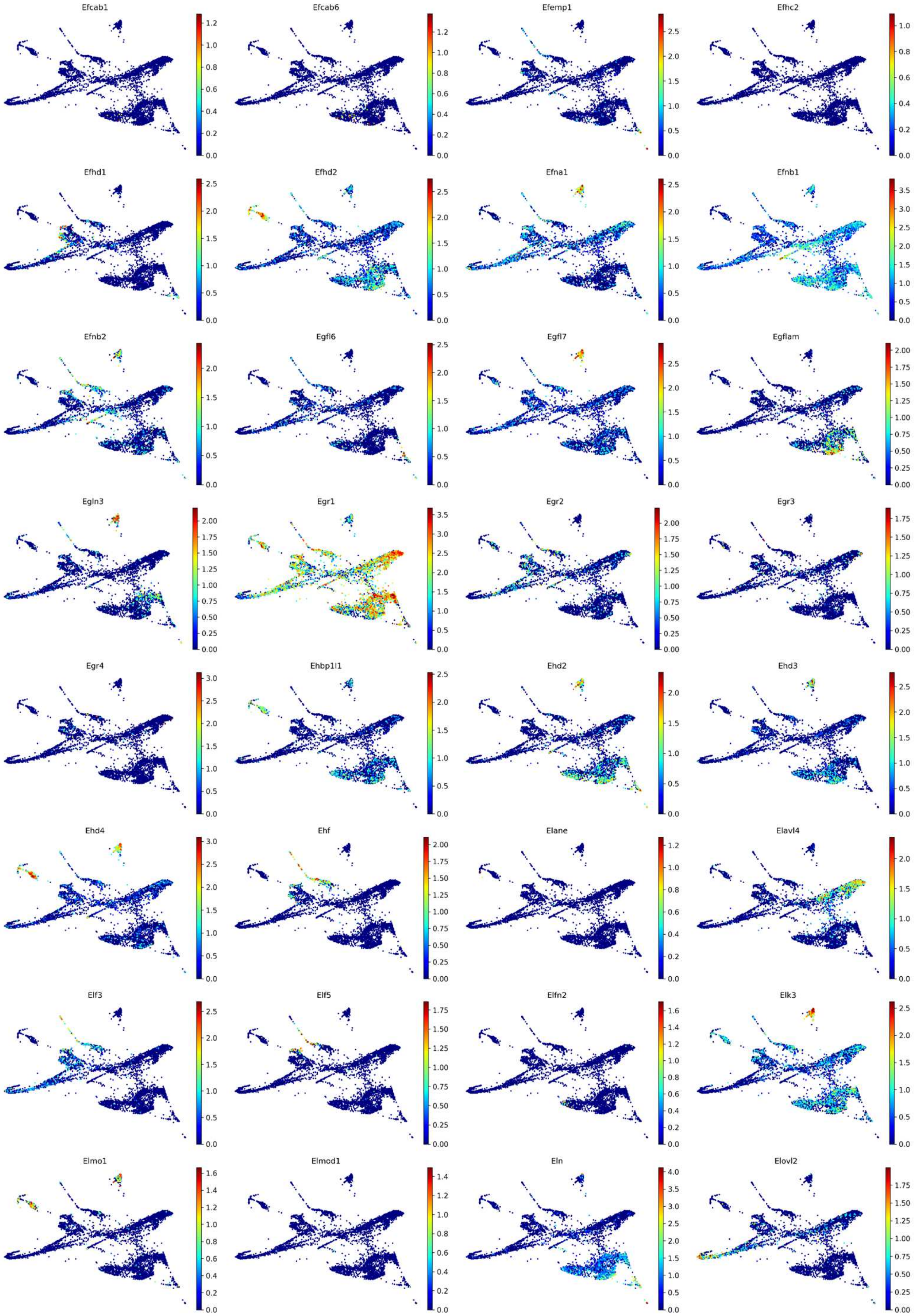

Supplementary Figure S5-29.

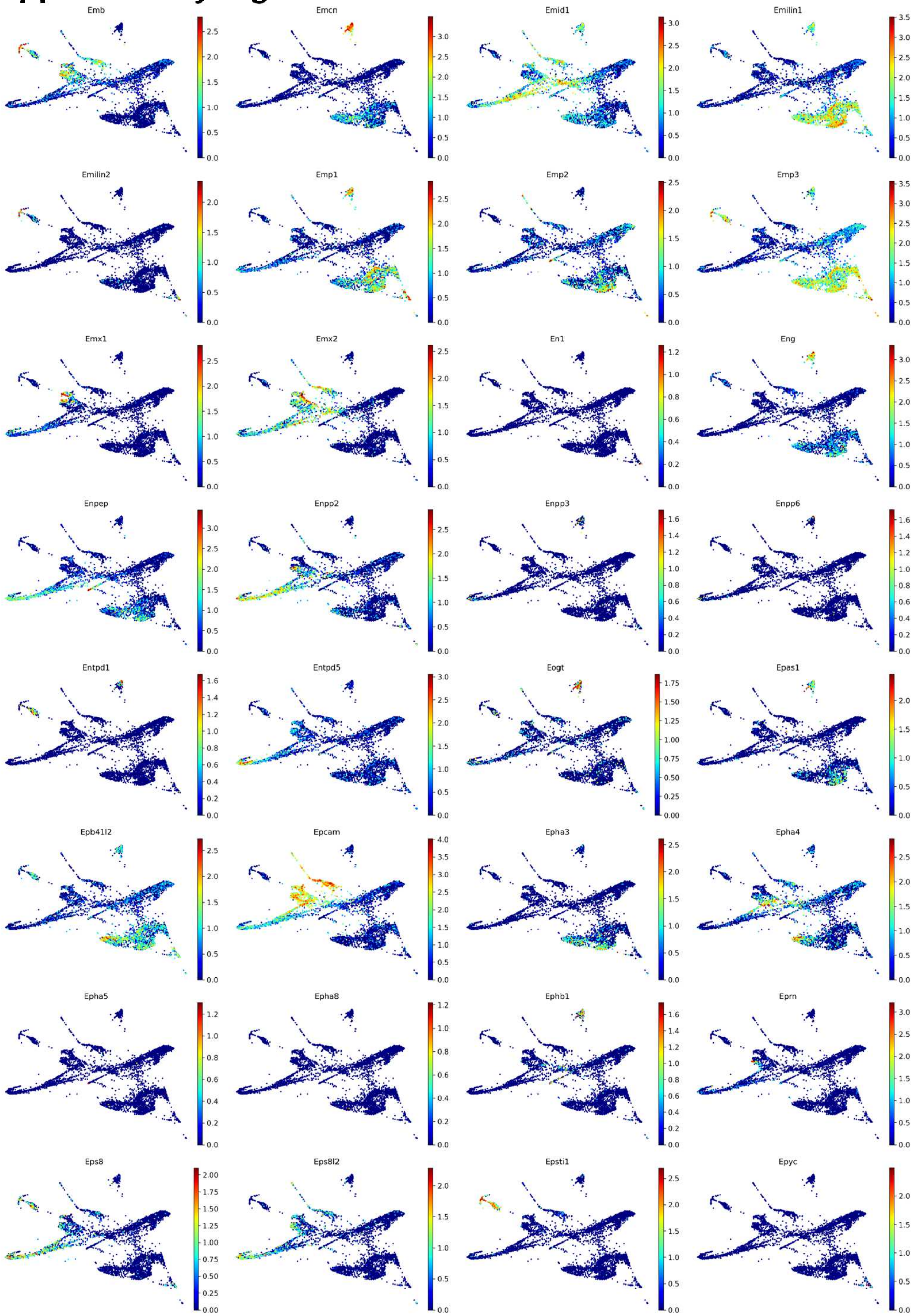

Supplementary Figure S5-30.

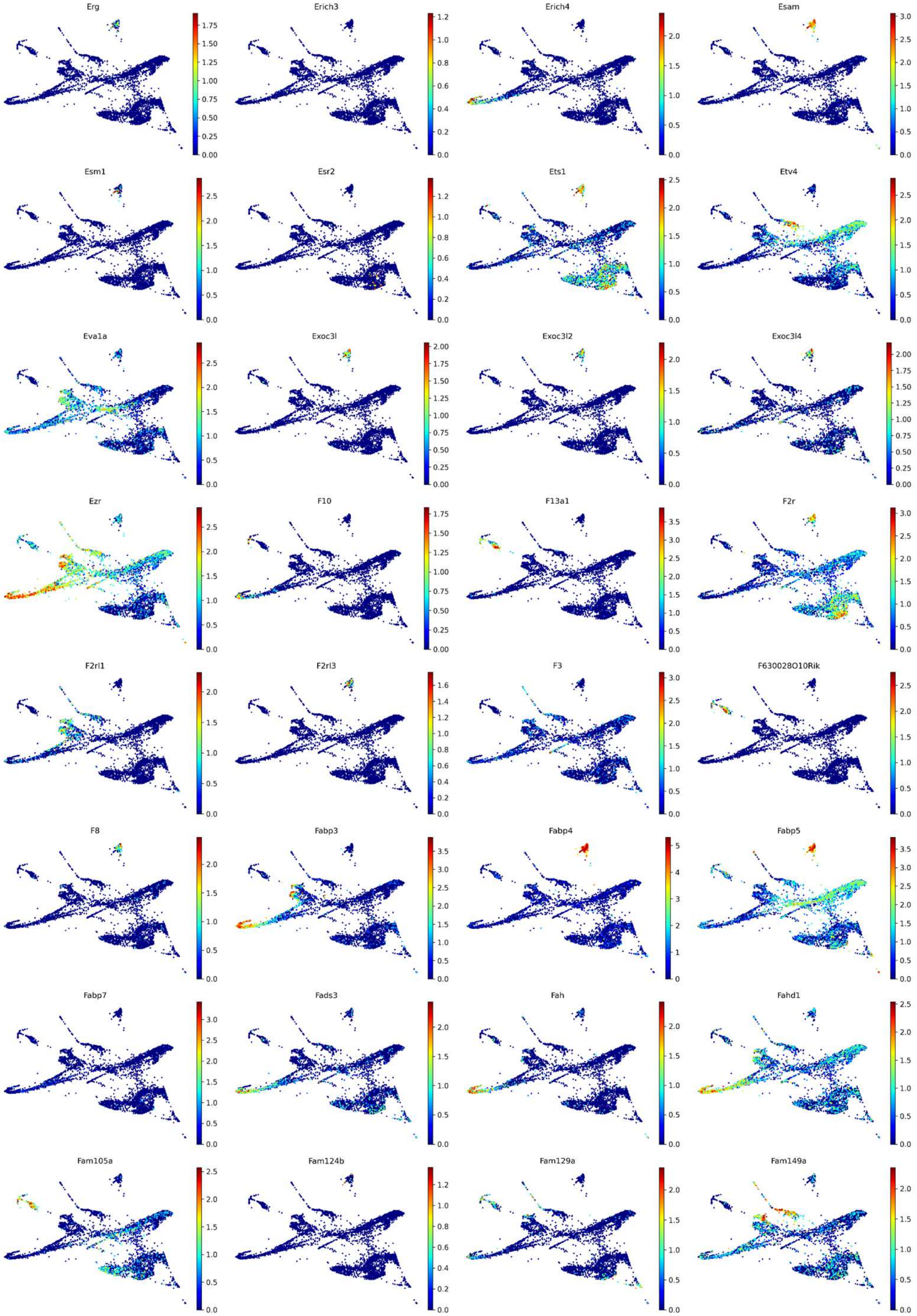

Supplement: Supplementary file 3 — Supplementary Information 3. [file 41598_2020_80154_MOESM3_ESM.pdf]
