# Supplementary material for: Single cell RNA sequencing uncovers cellular developmental sequences and novel potential intercellular communications in embryonic kidney
Source: Sci Rep. 2021 Jan 8;11:73. doi: 10.1038/s41598-020-80154-y (PMC7794461; doi:10.1038/s41598-020-80154-y)

Supplementary Figure S5-31.

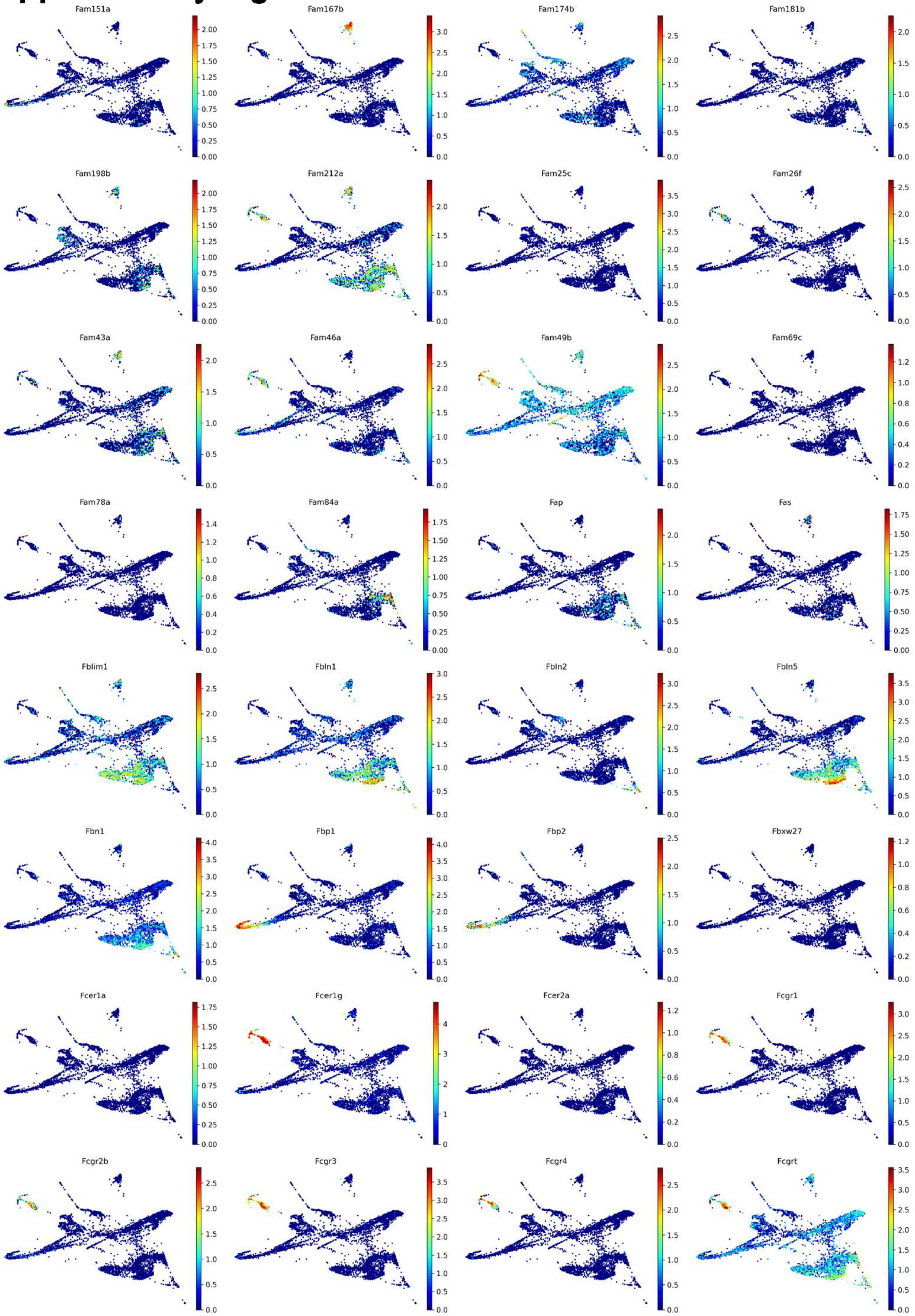

Supplementary Figure S5-32.

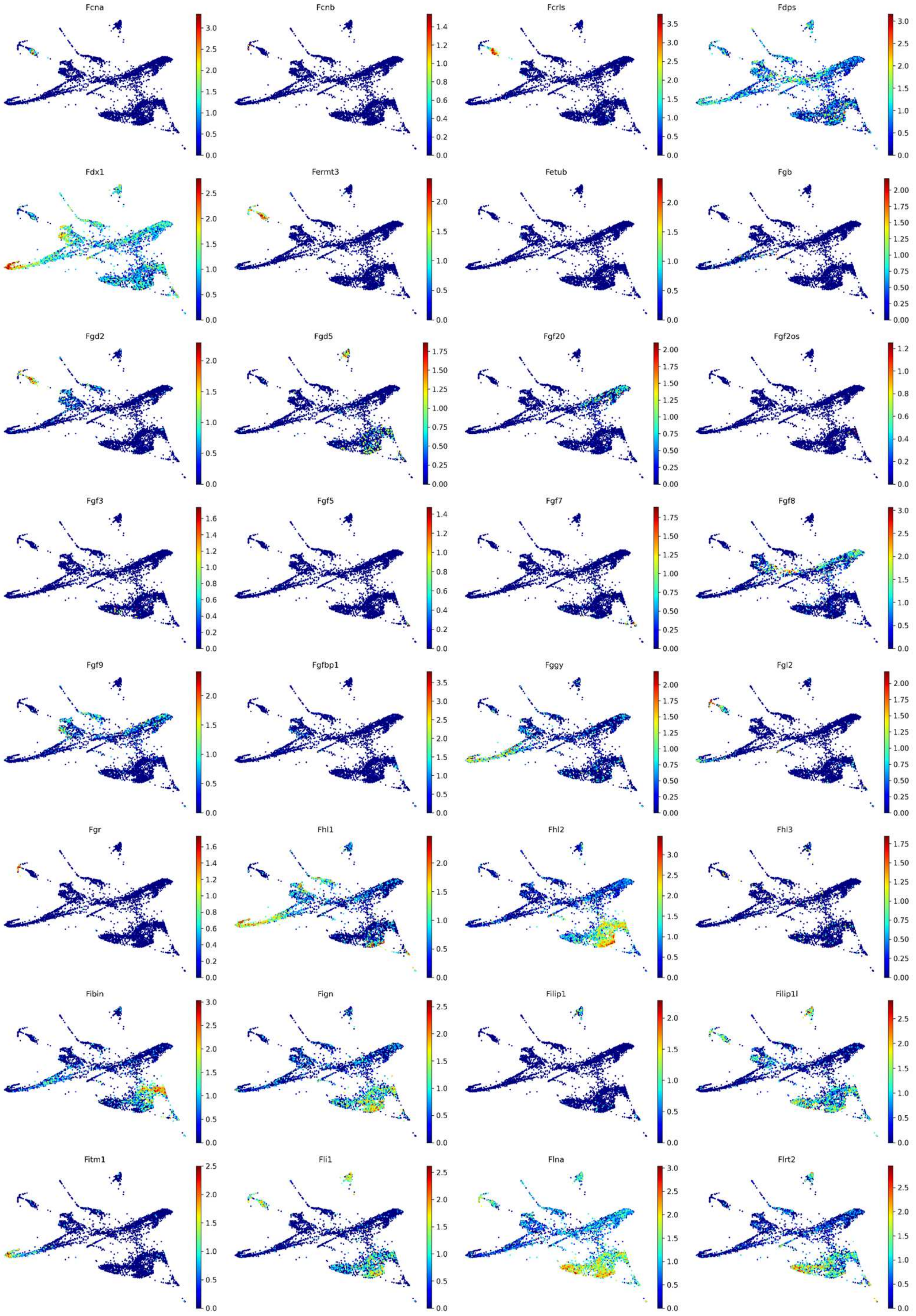

Supplementary Figure S5-33.

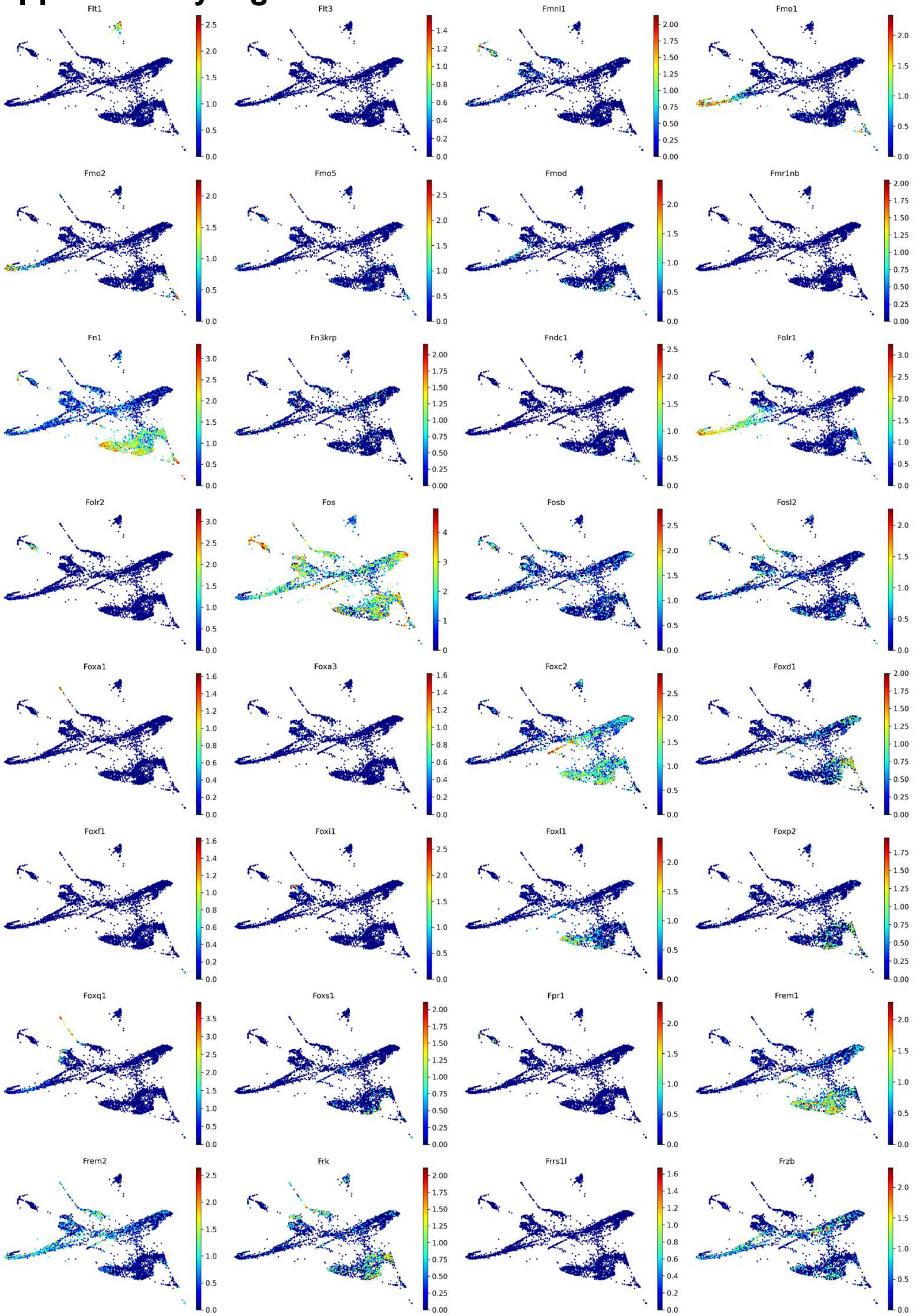

Supplementary Figure S5-34.

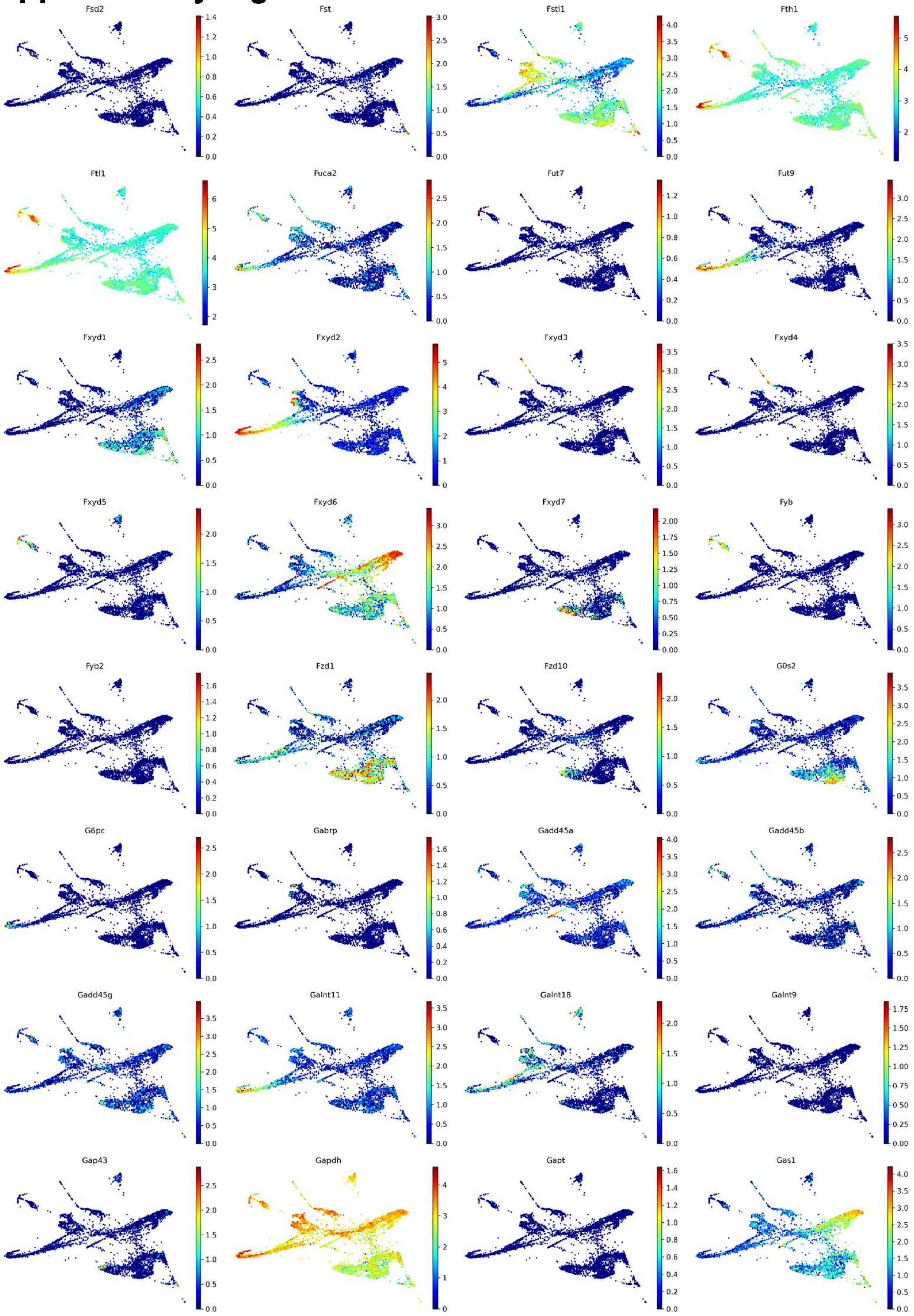

Supplementary Figure S5-35.

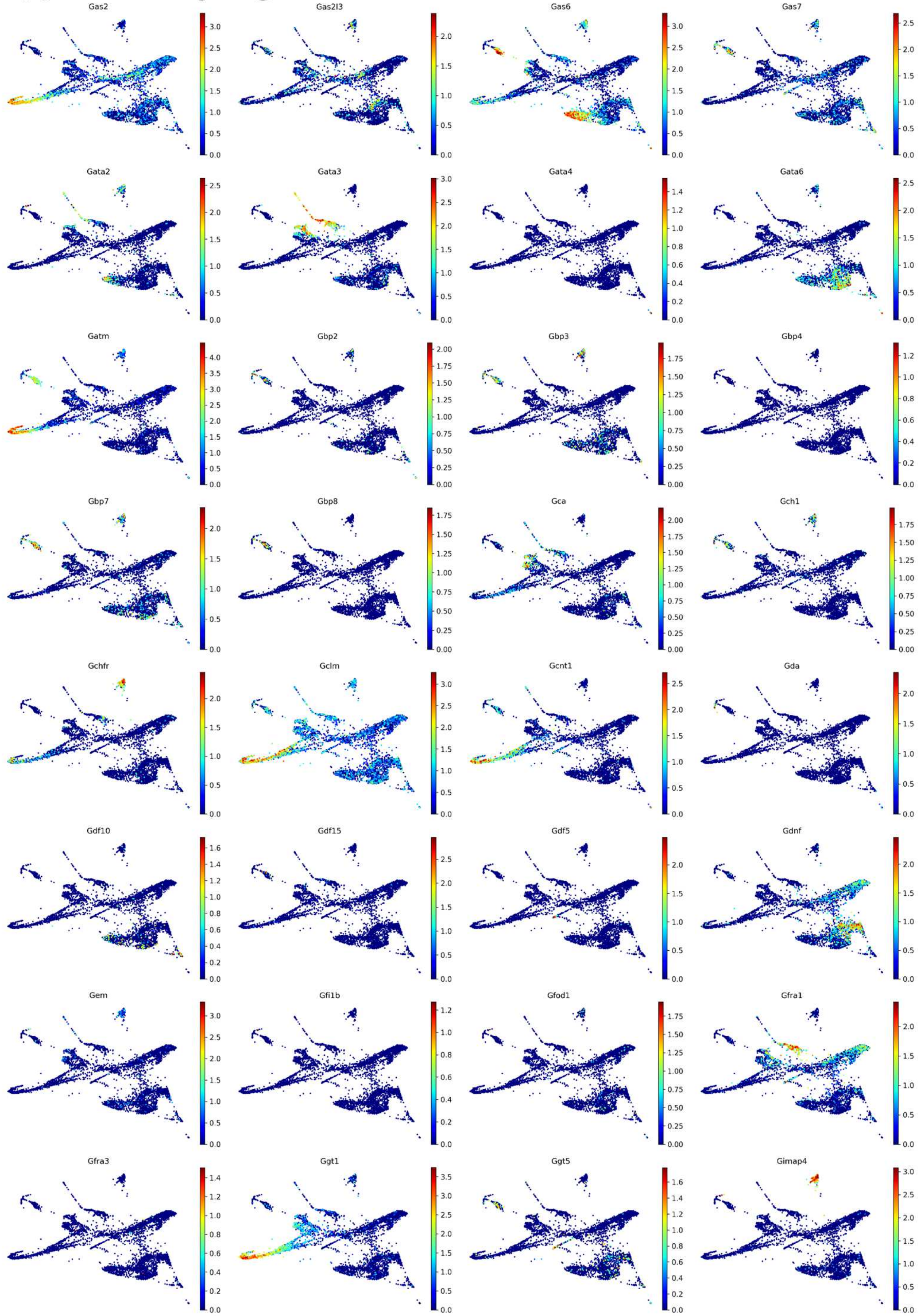

Supplementary Figure S5-36.

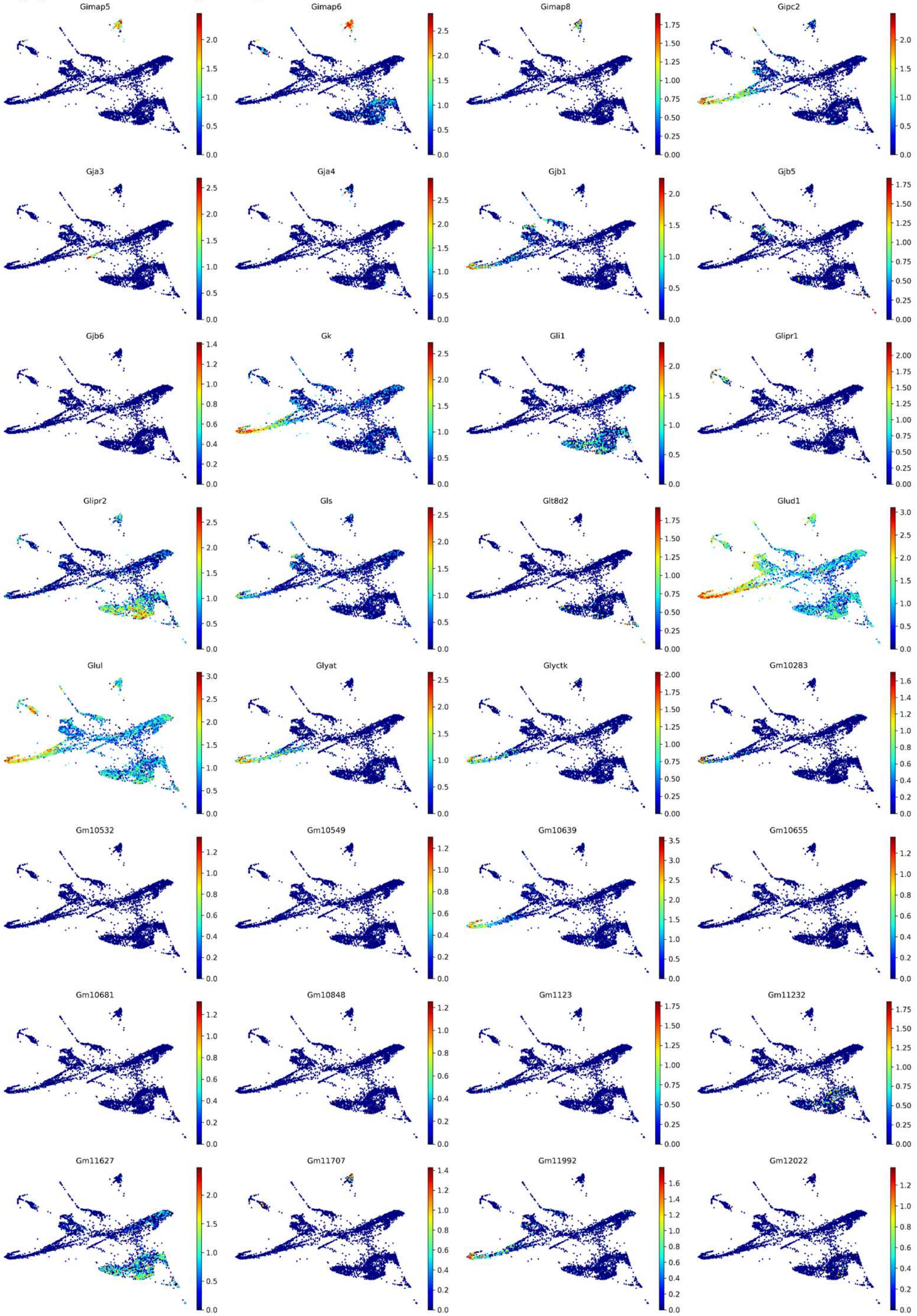

Supplementary Figure S5-37.

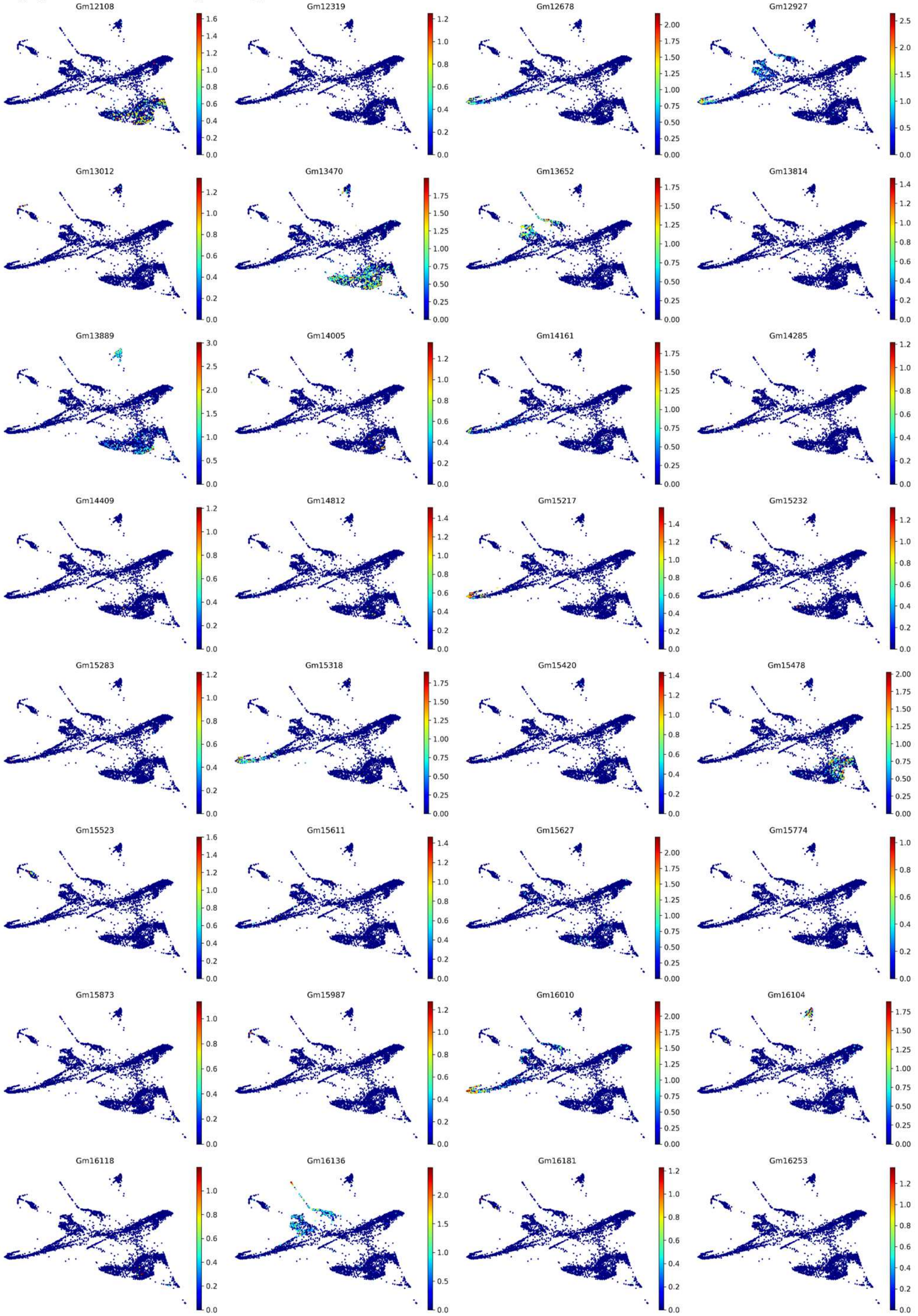

Supplementary Figure S5-38.

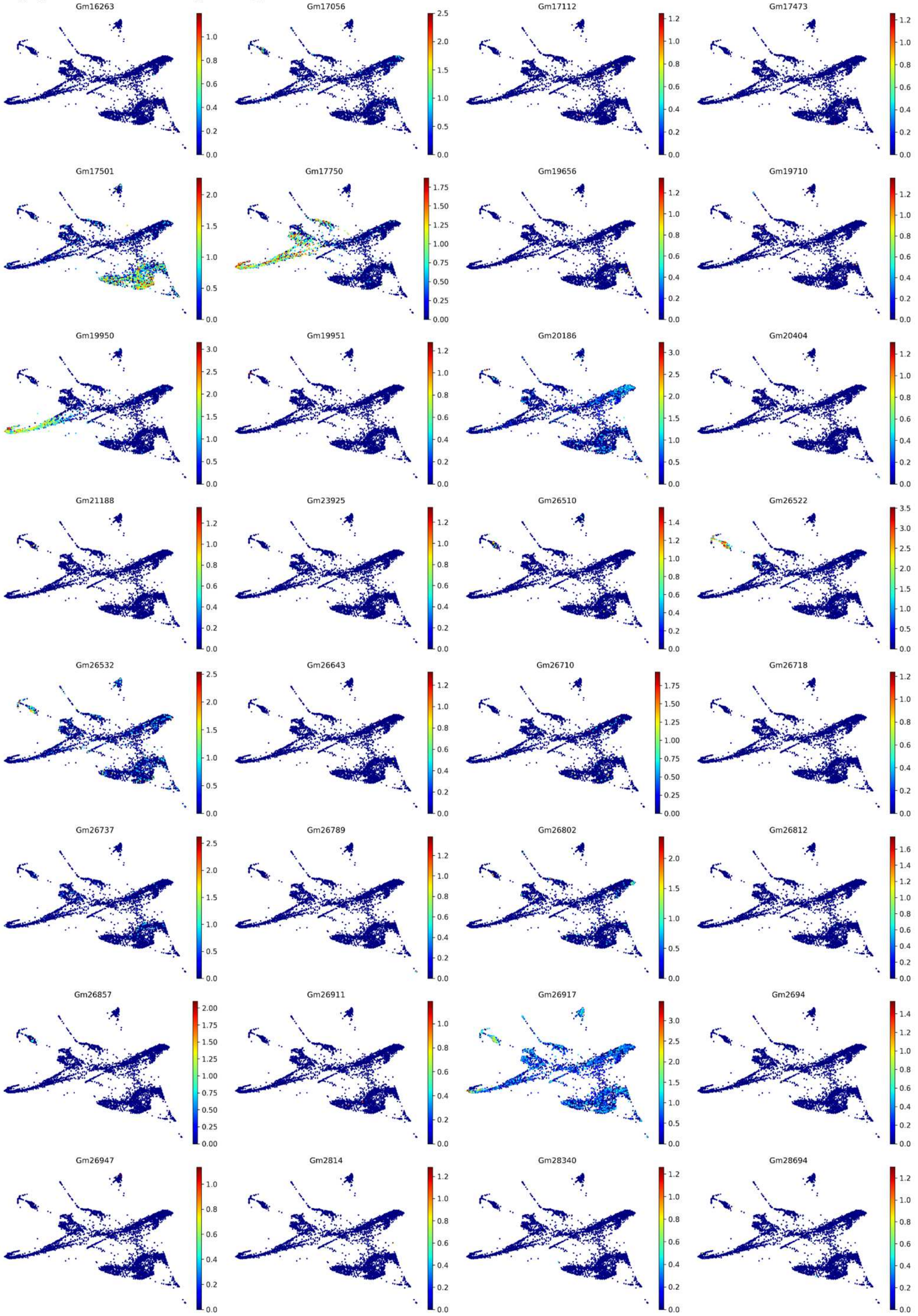

Supplementary Figure S5-39.

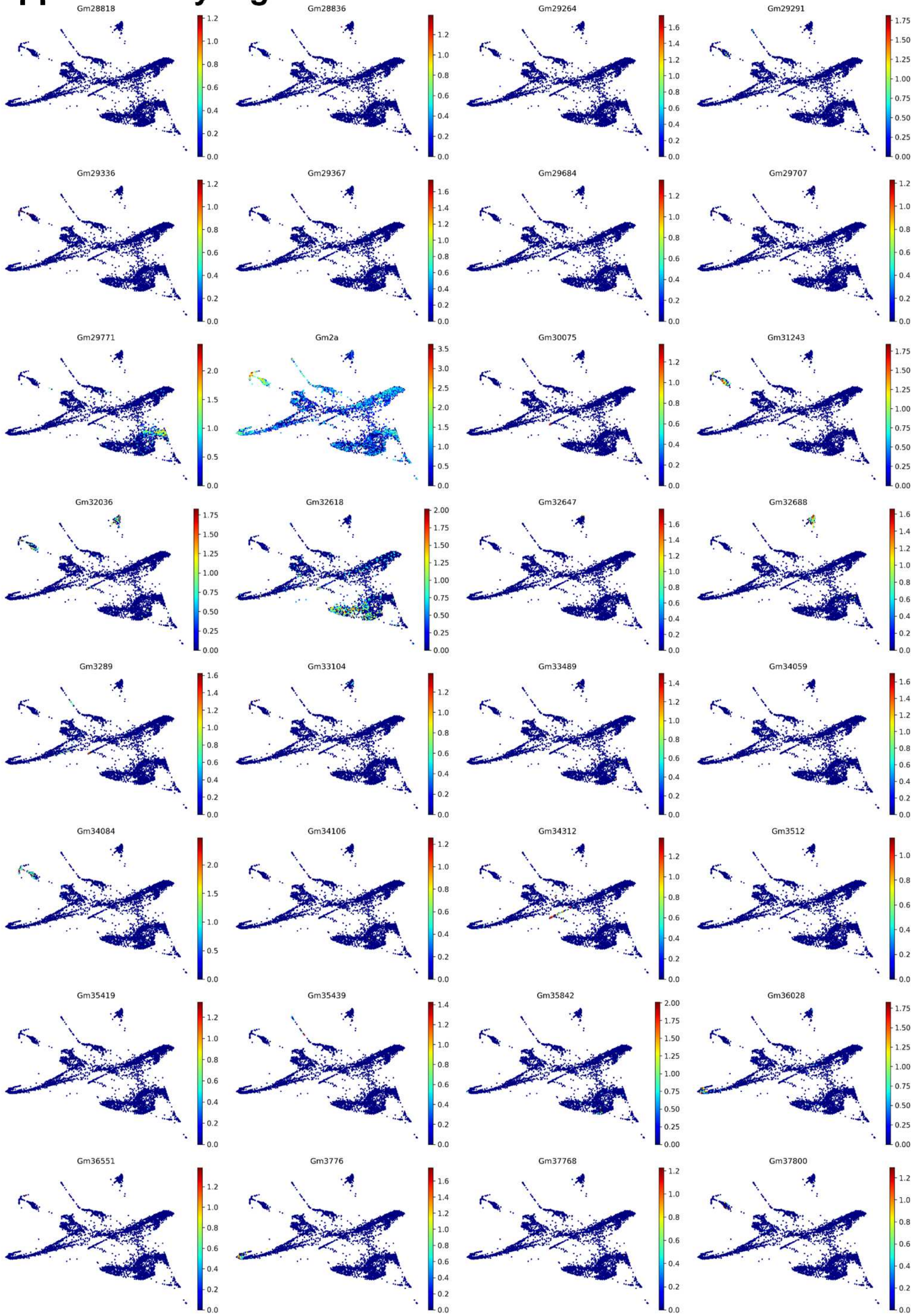

Supplementary Figure S5-40.

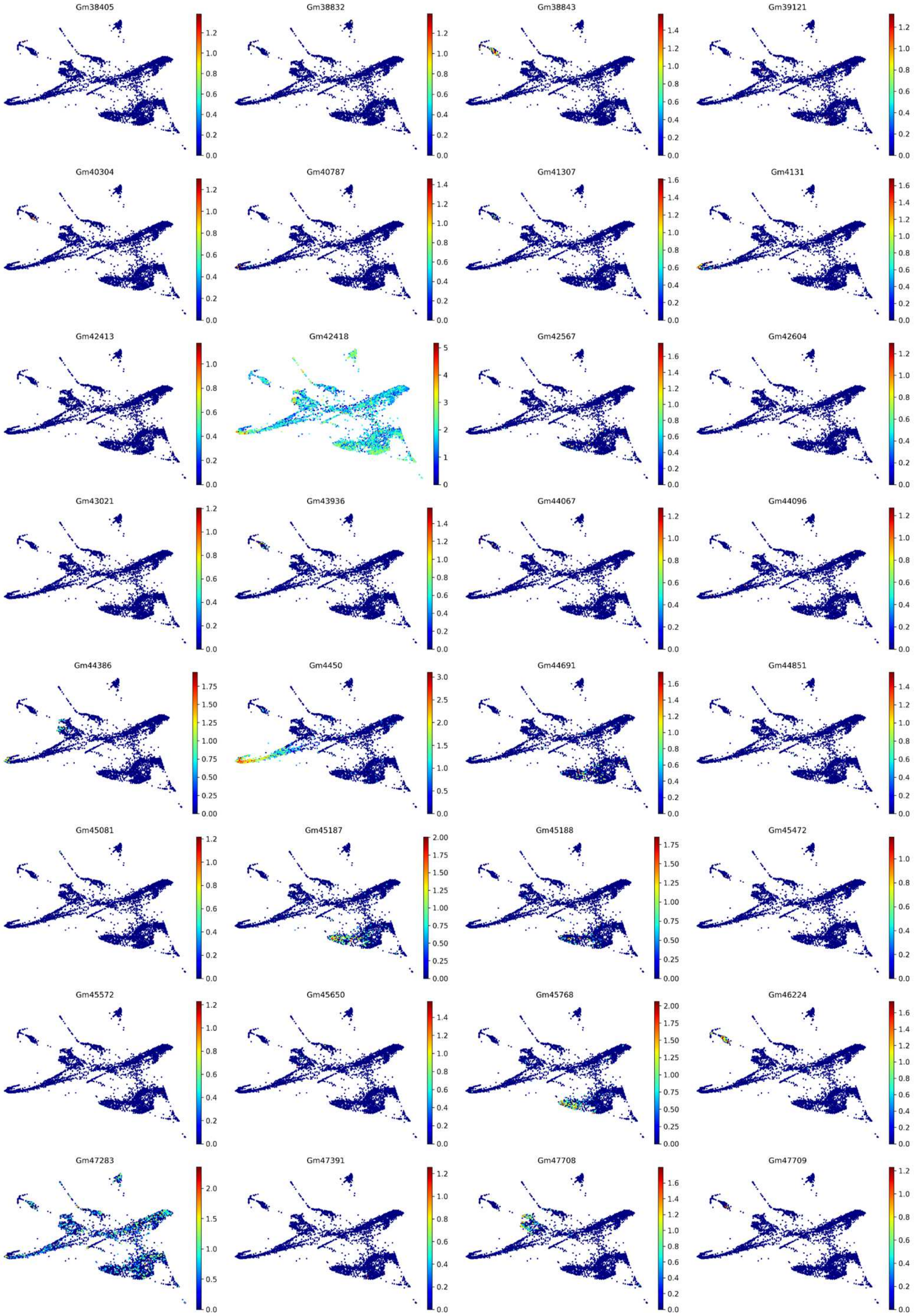

Supplementary Figure S5-41.

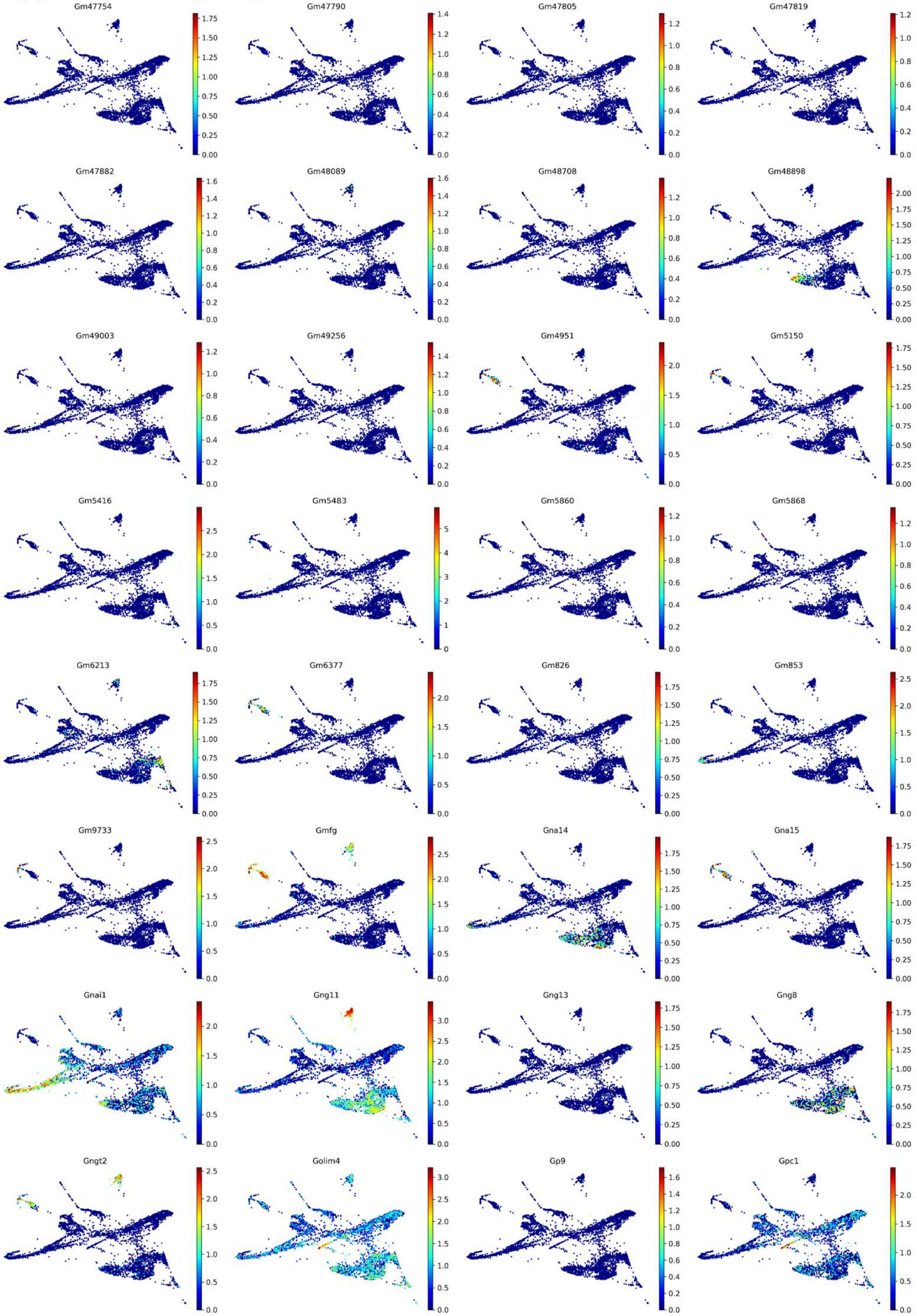

Supplementary Figure S5-42.

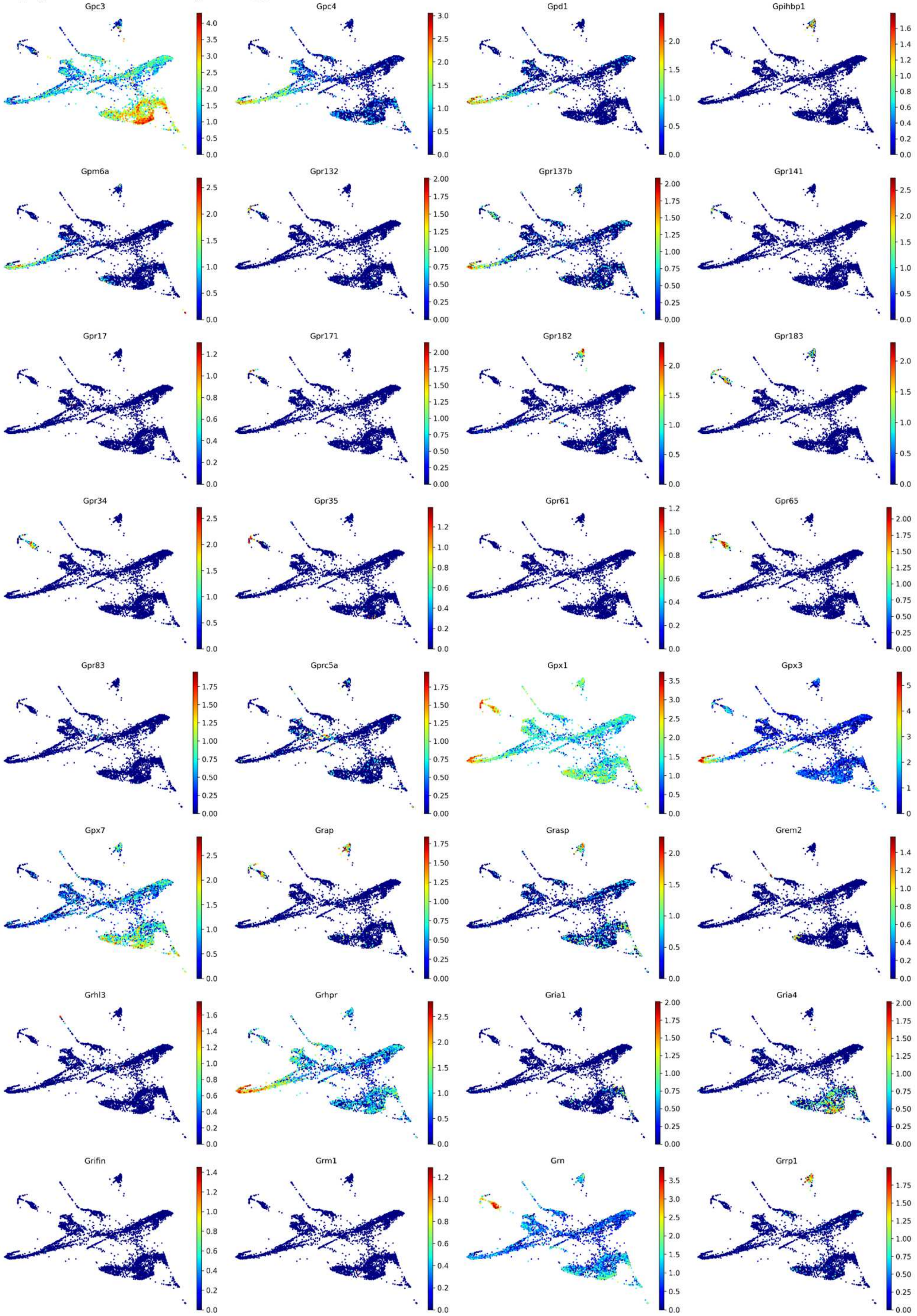

Supplementary Figure S5-43.

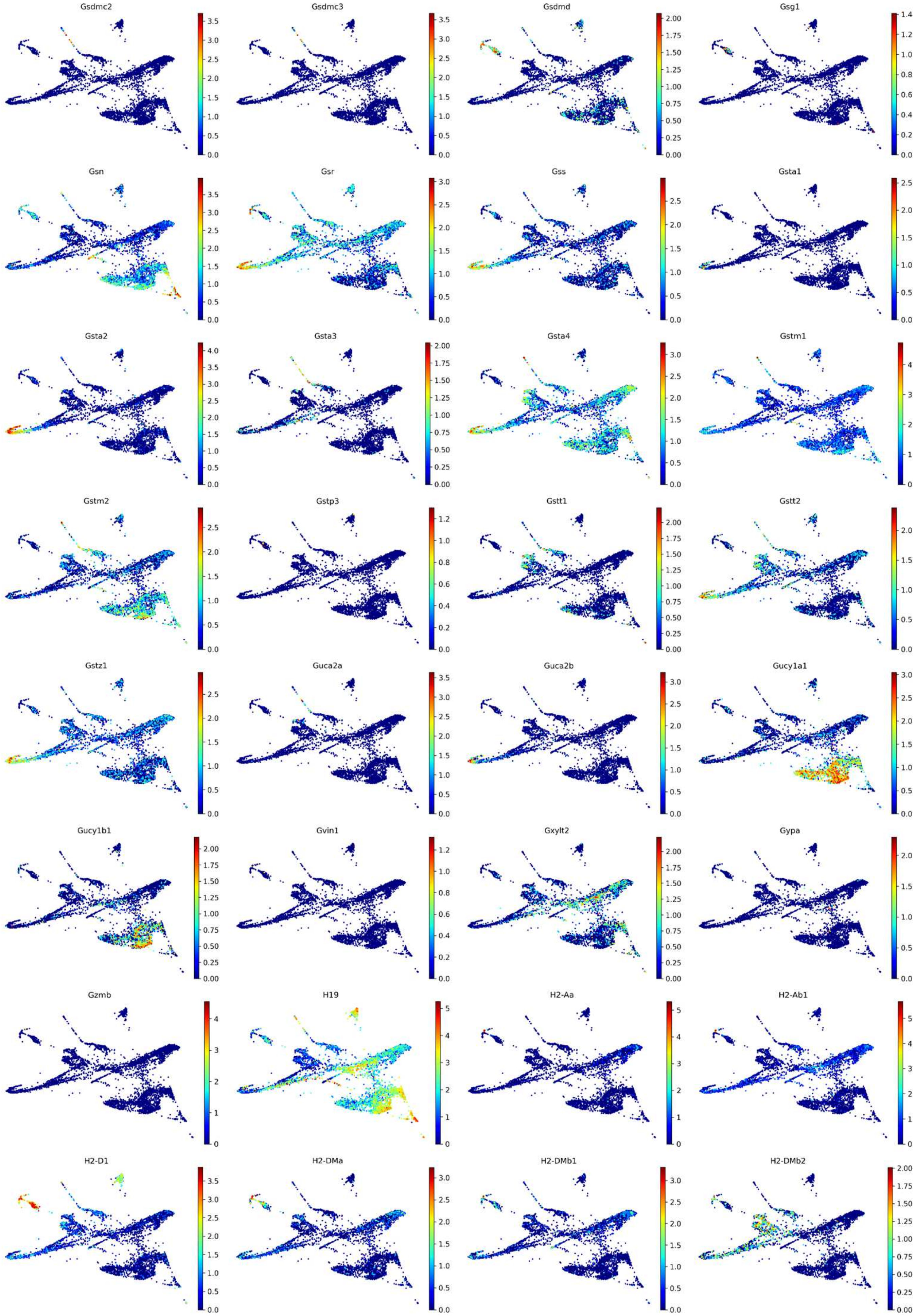

Supplementary Figure S5-44.

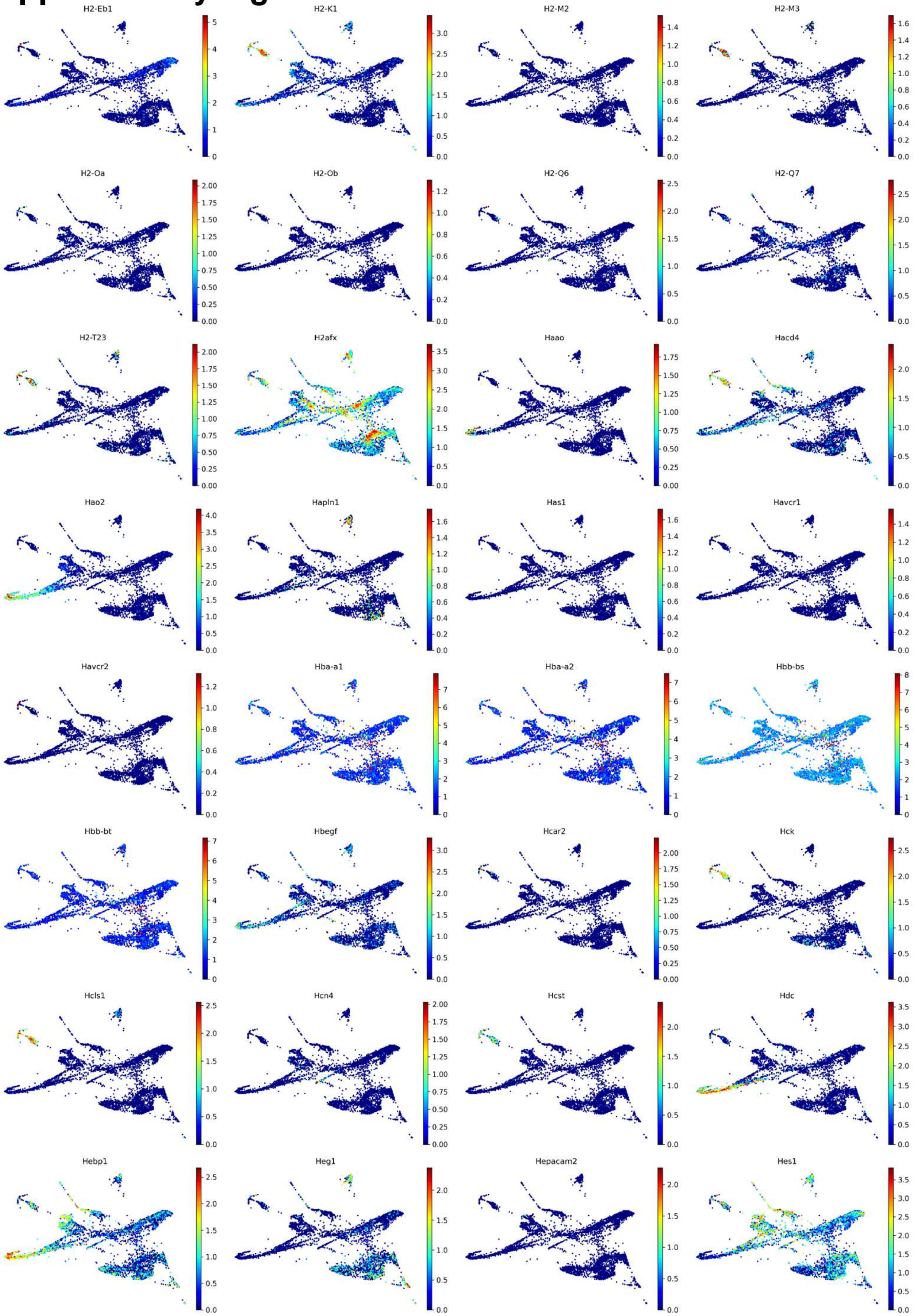

Supplementary Figure S5-45.

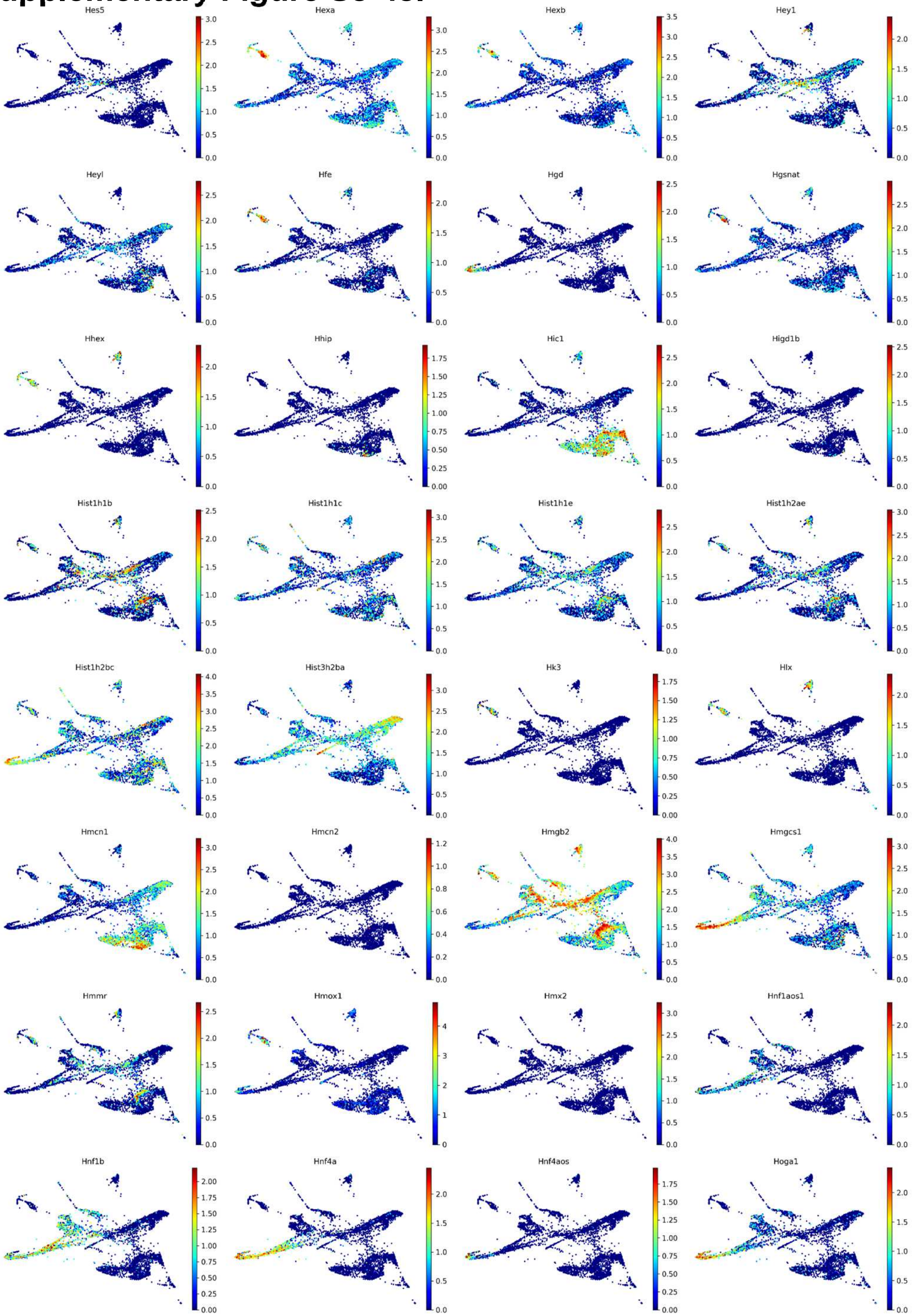

Supplementary Figure S5-46.

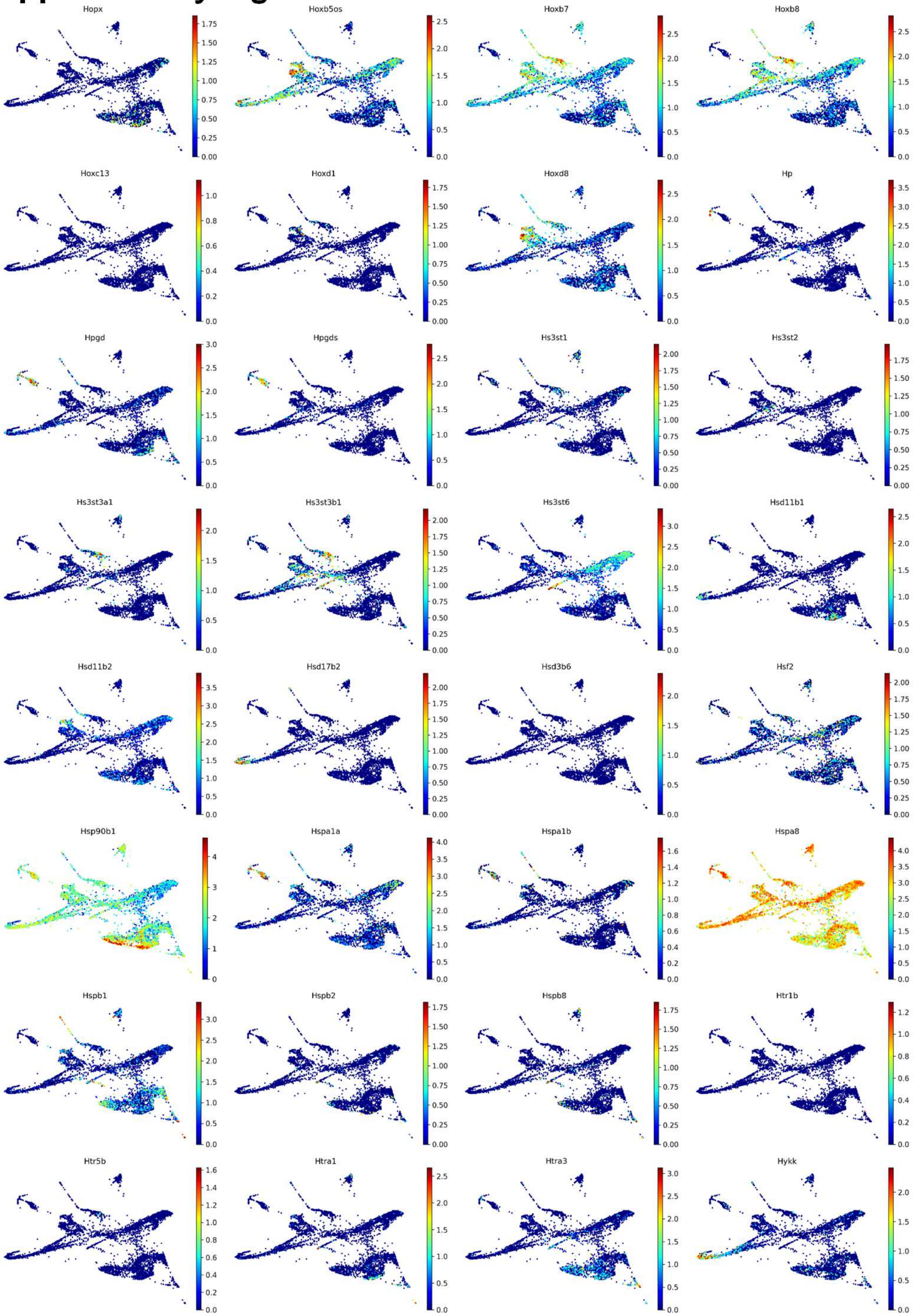

Supplementary Figure S5-47.

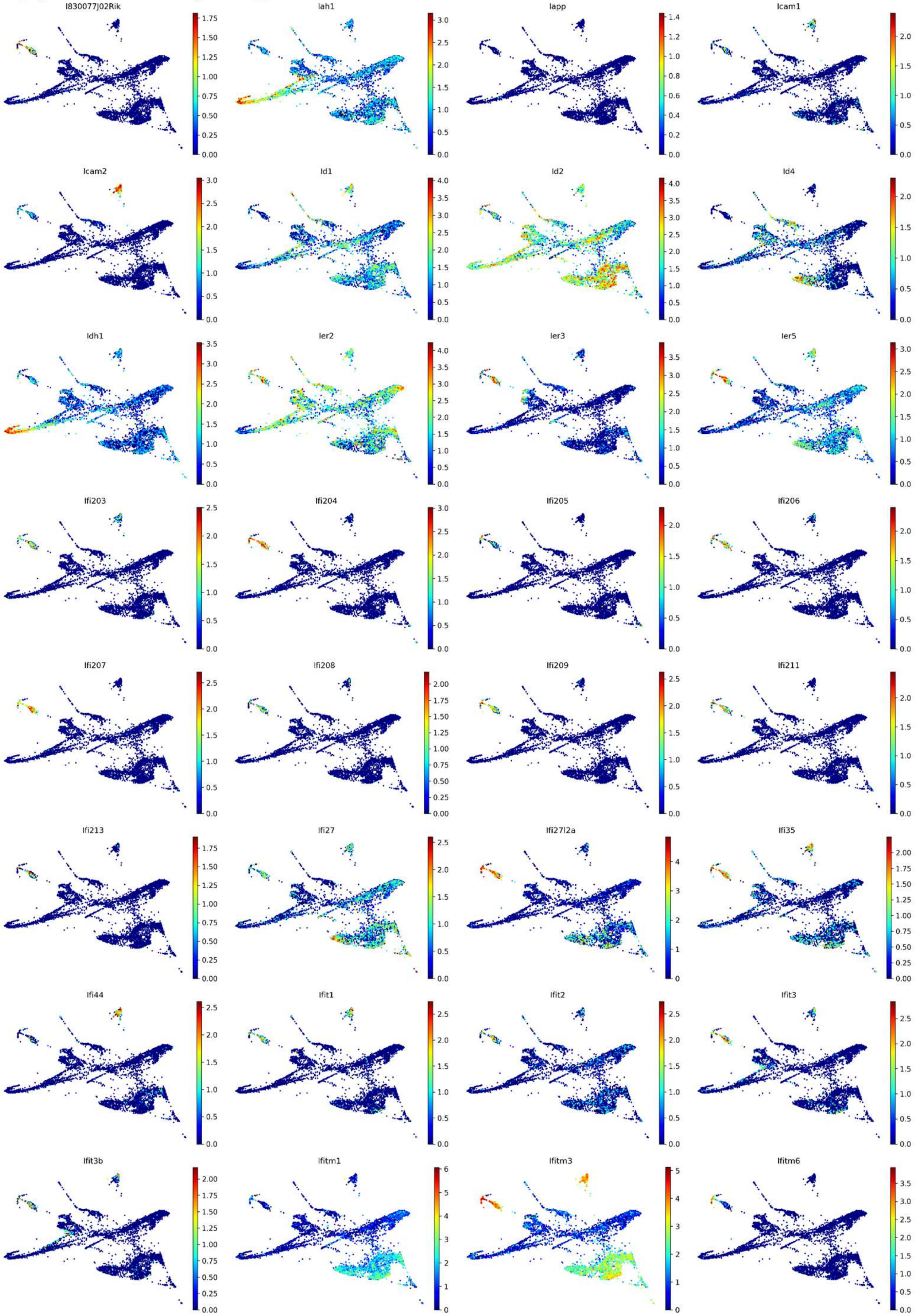

Supplementary Figure S5-48.

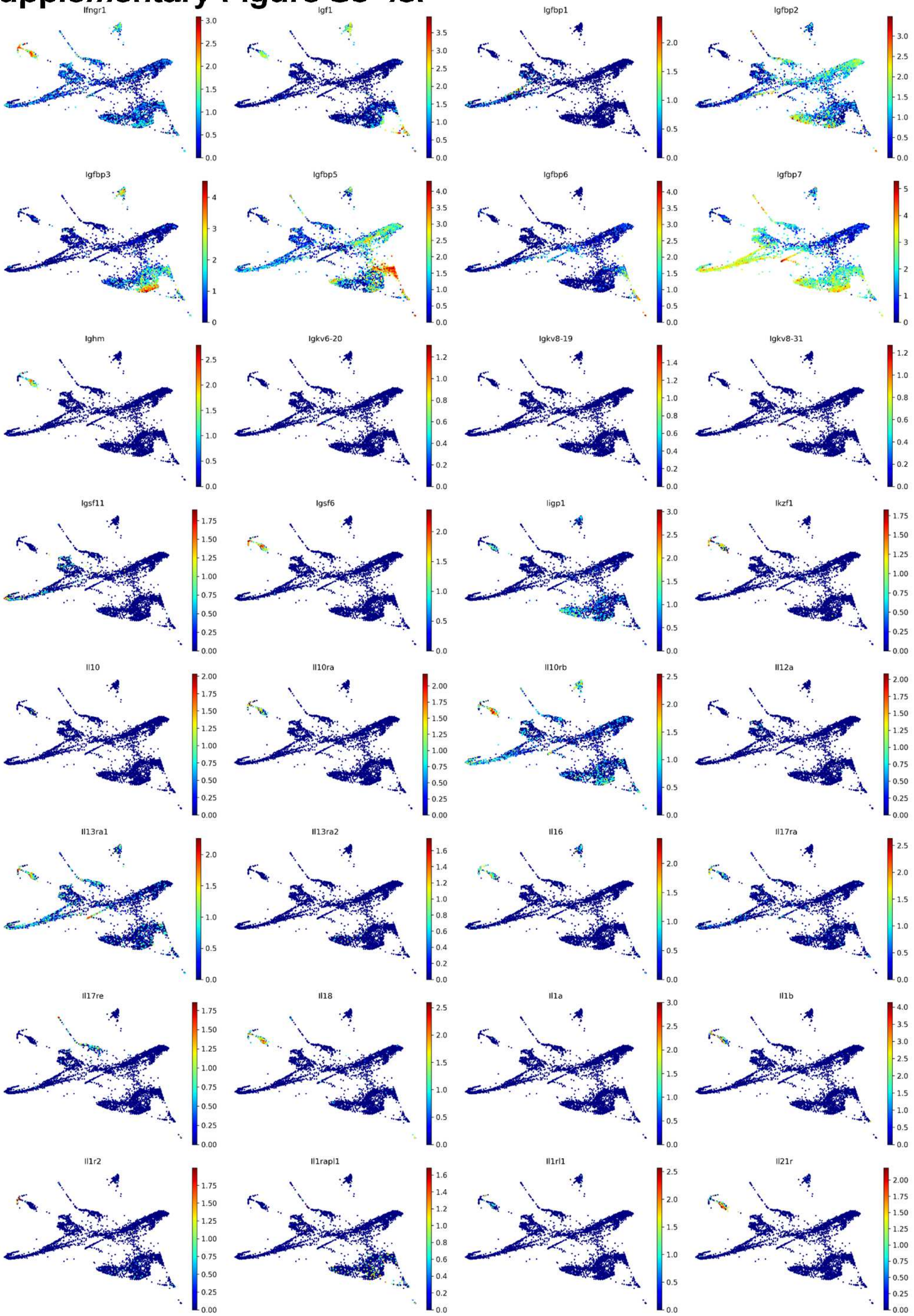

Supplementary Figure S5-49.

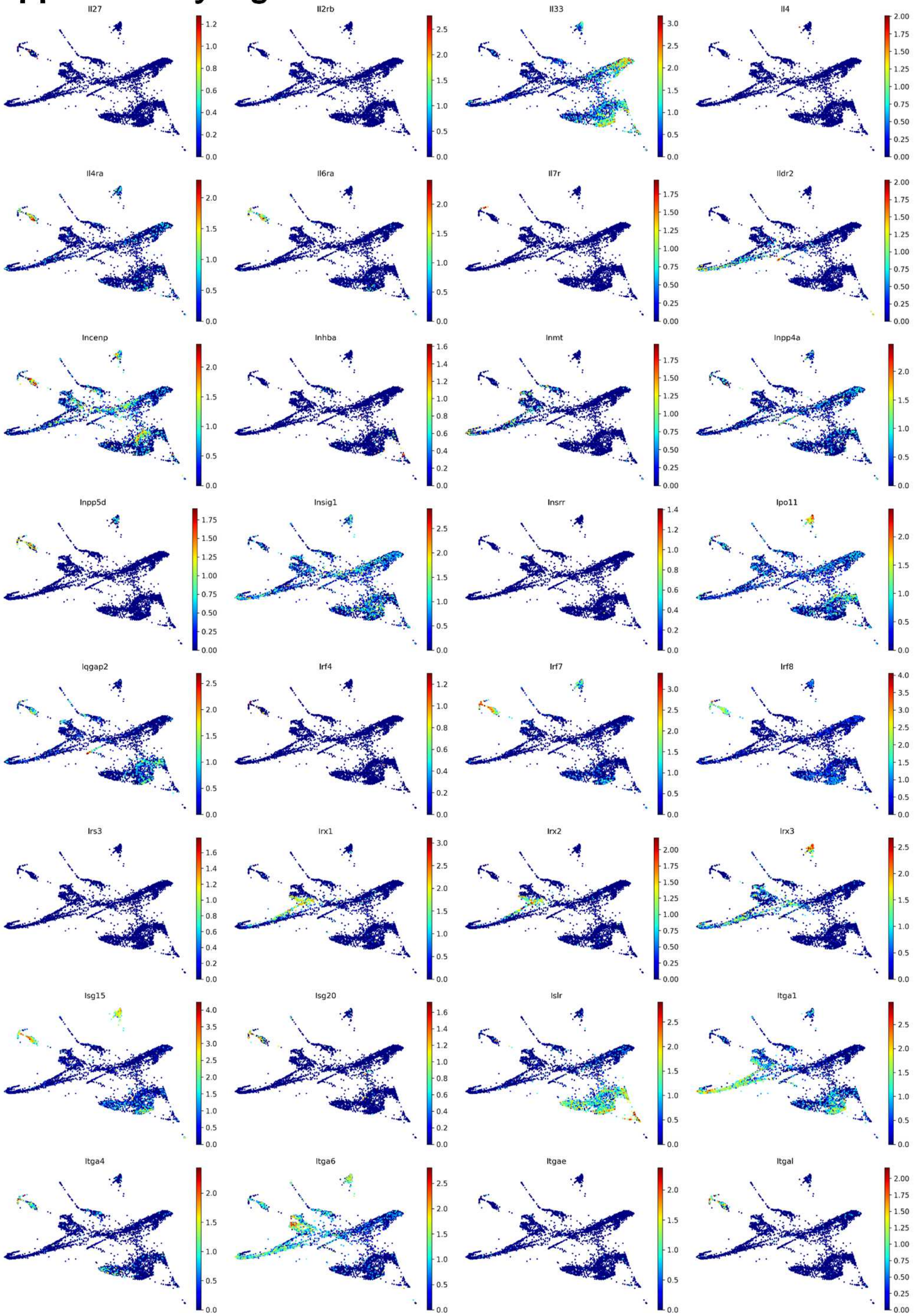

Supplementary Figure S5-50.

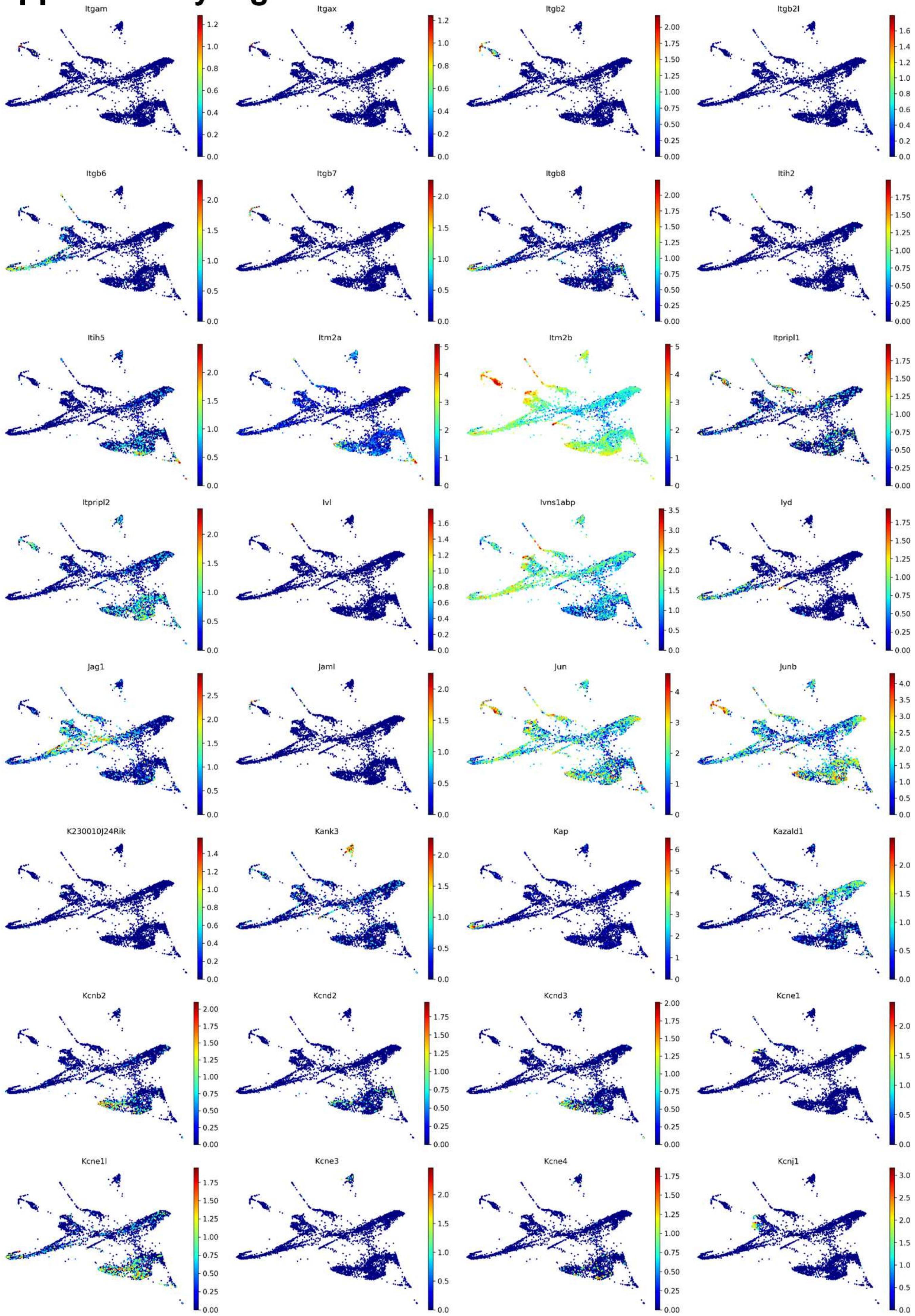

Supplementary Figure S5-51.

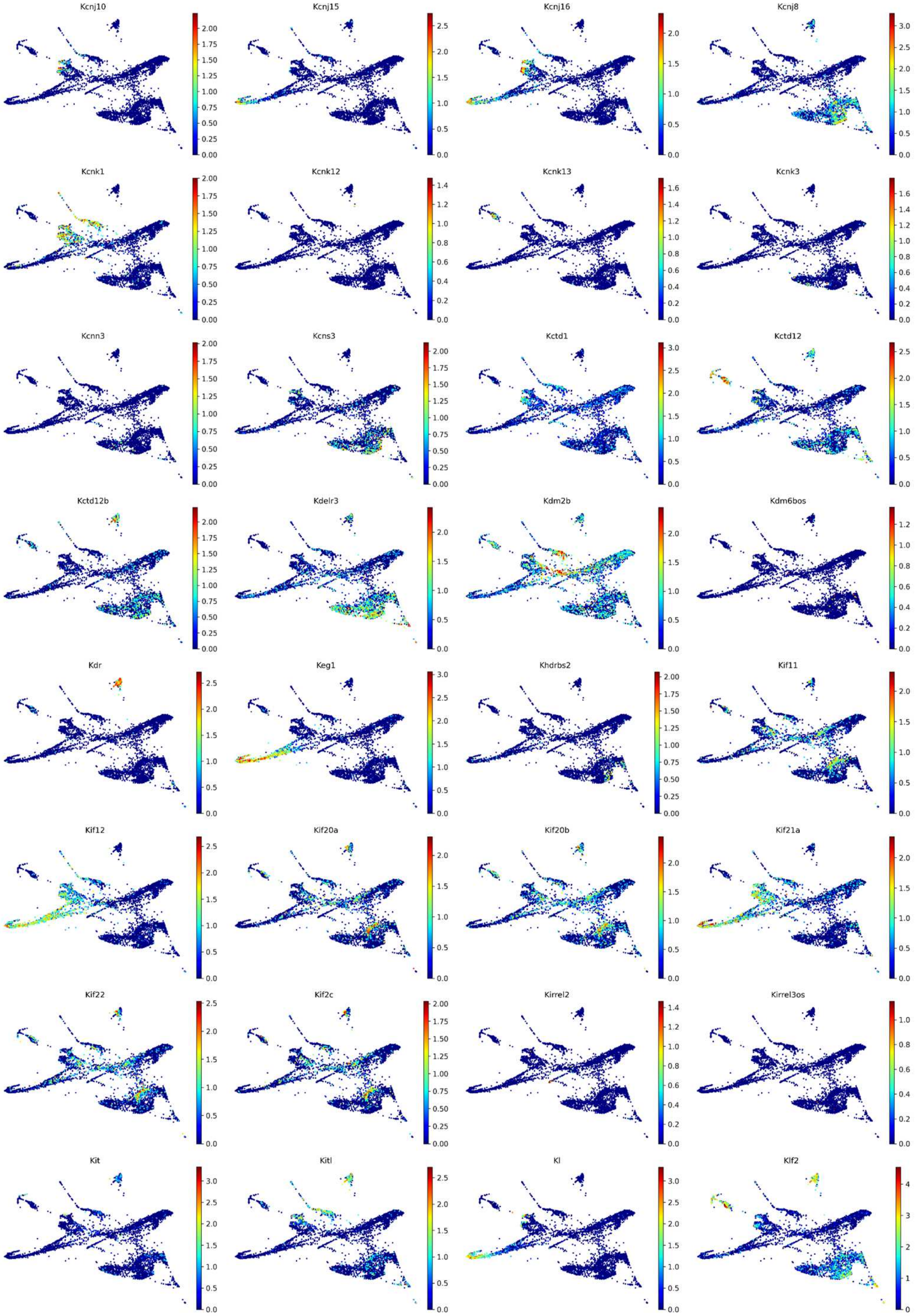

Supplementary Figure S5-52.

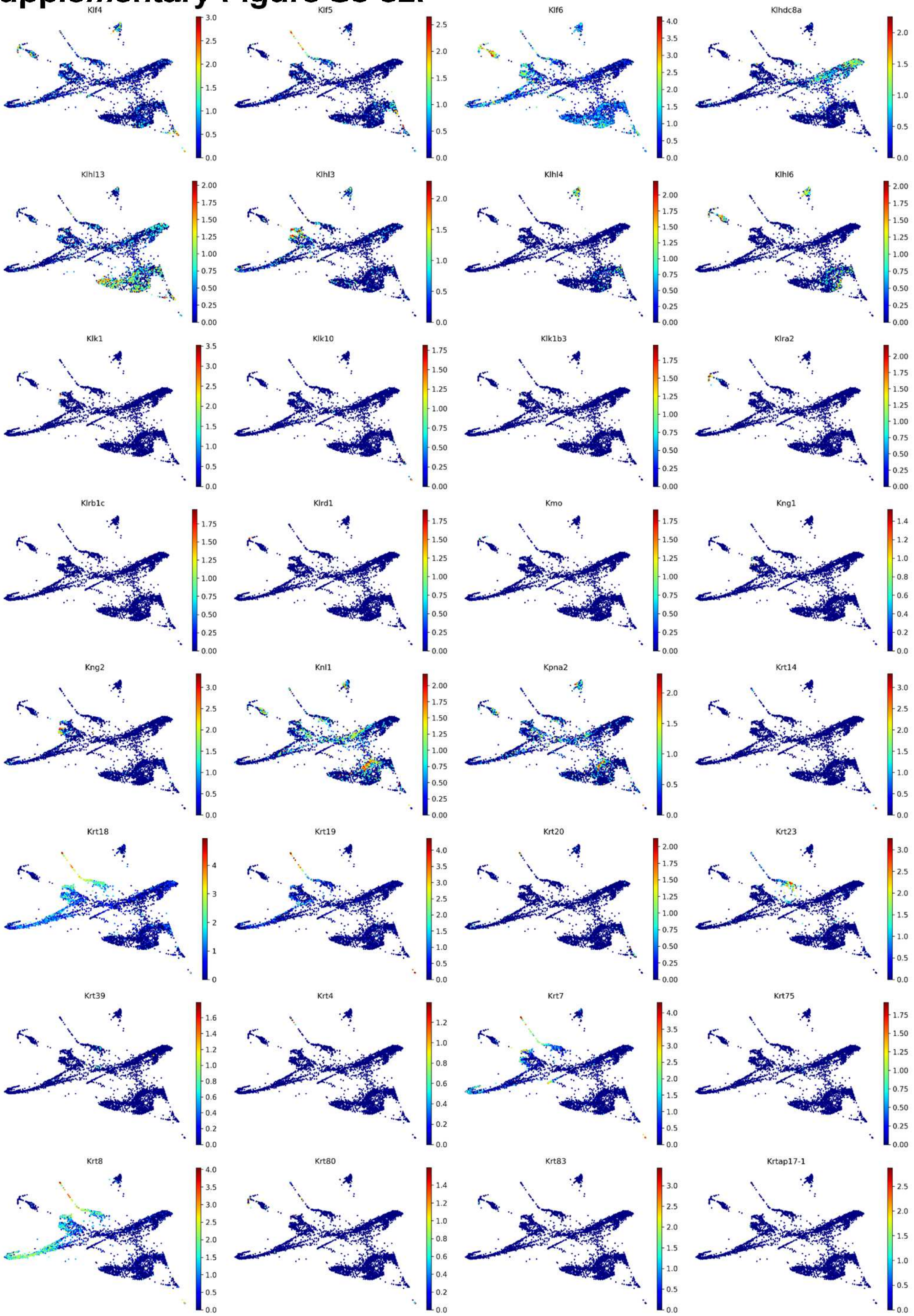

Supplementary Figure S5-53.

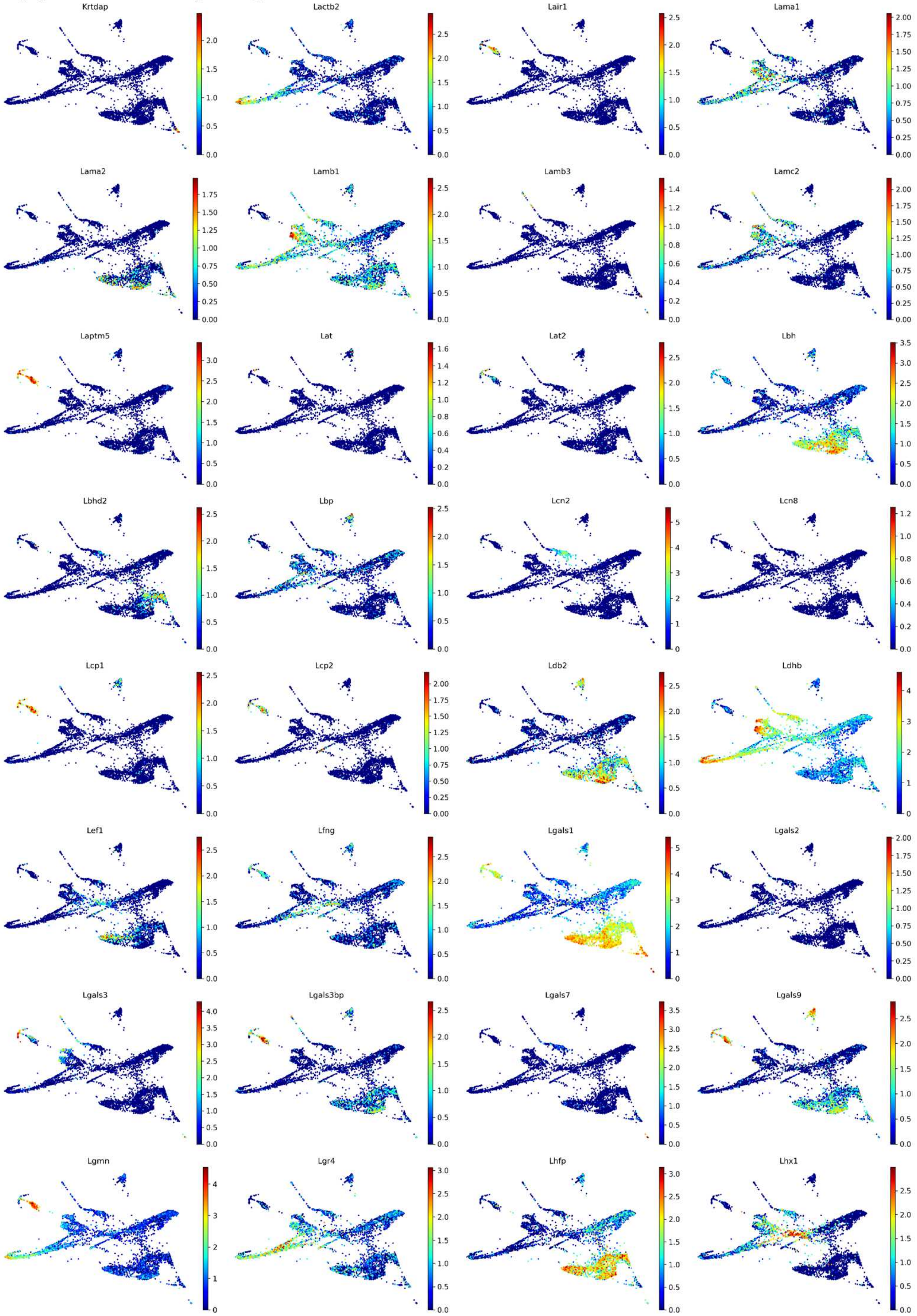

Supplementary Figure S5-54.

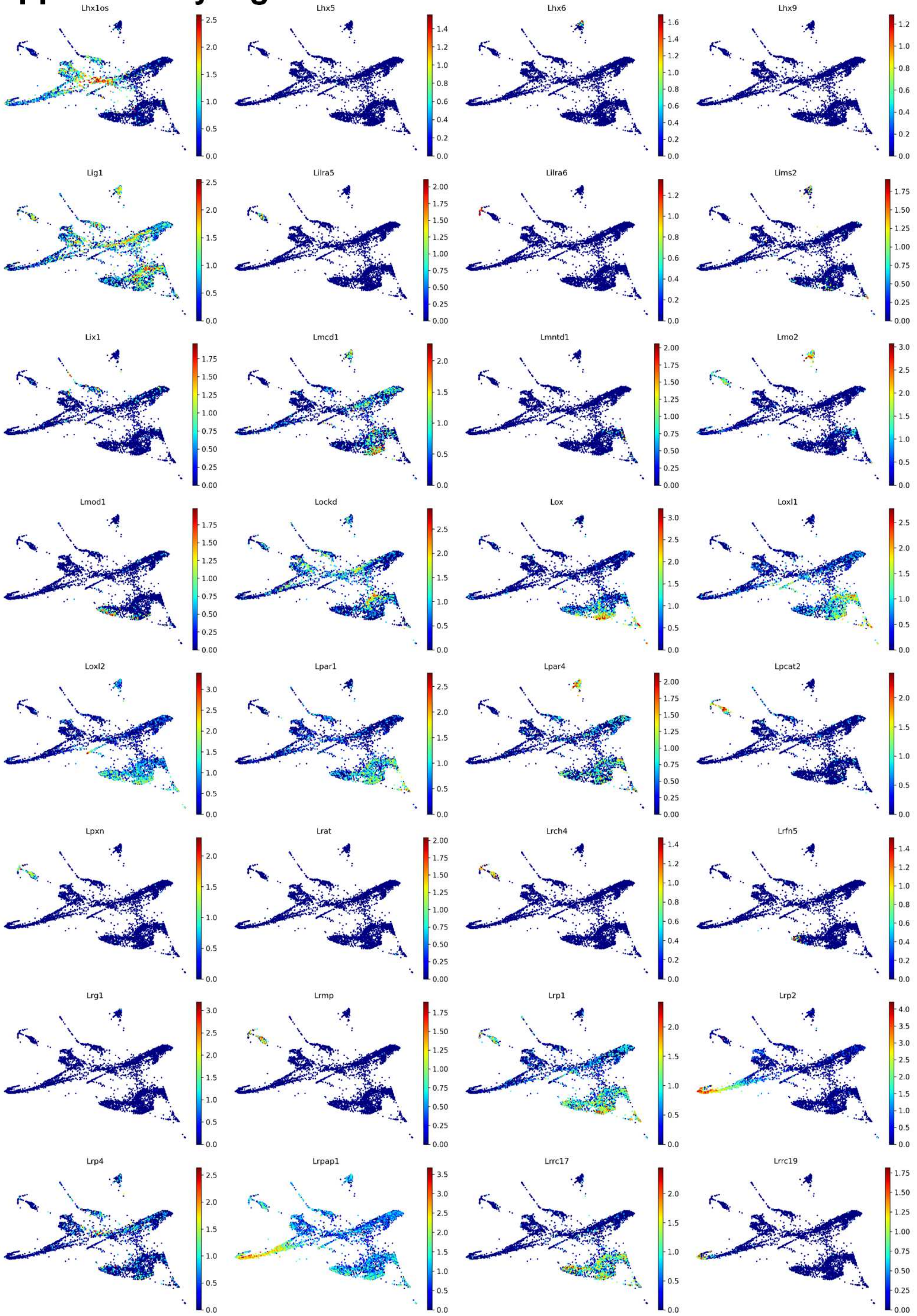

Supplementary Figure S5-55.

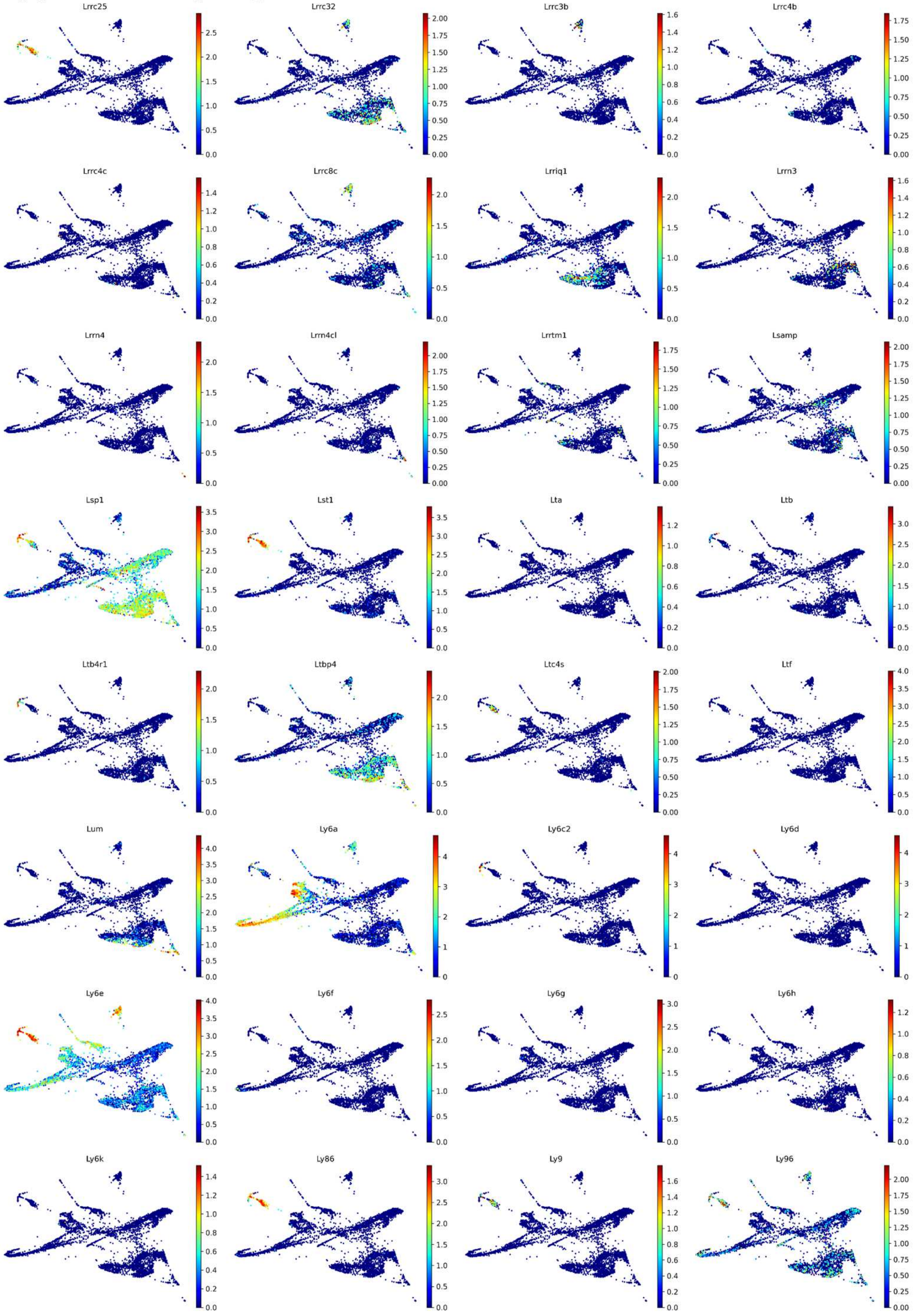

Supplementary Figure S5-56.

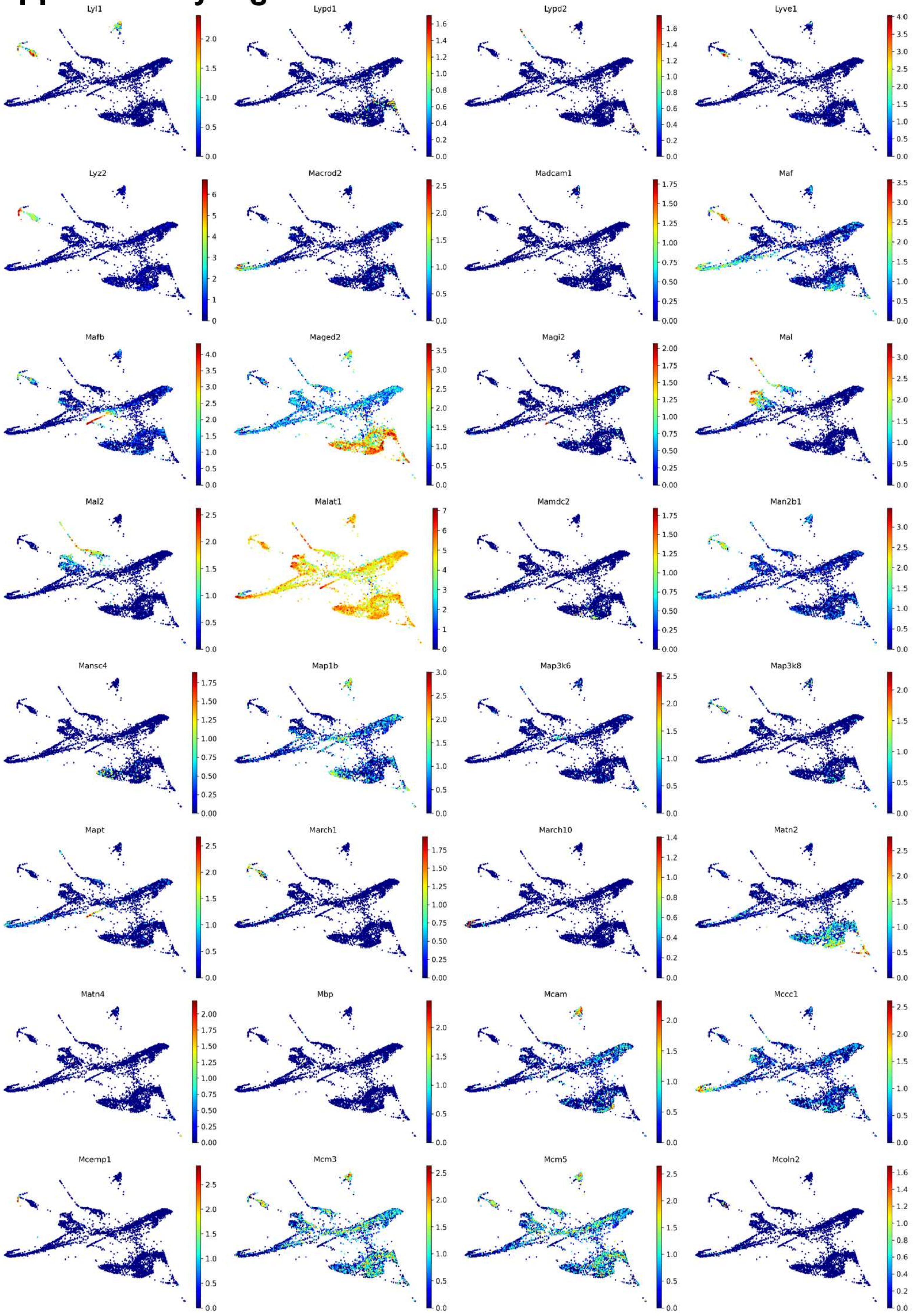

Supplementary Figure S5-57.

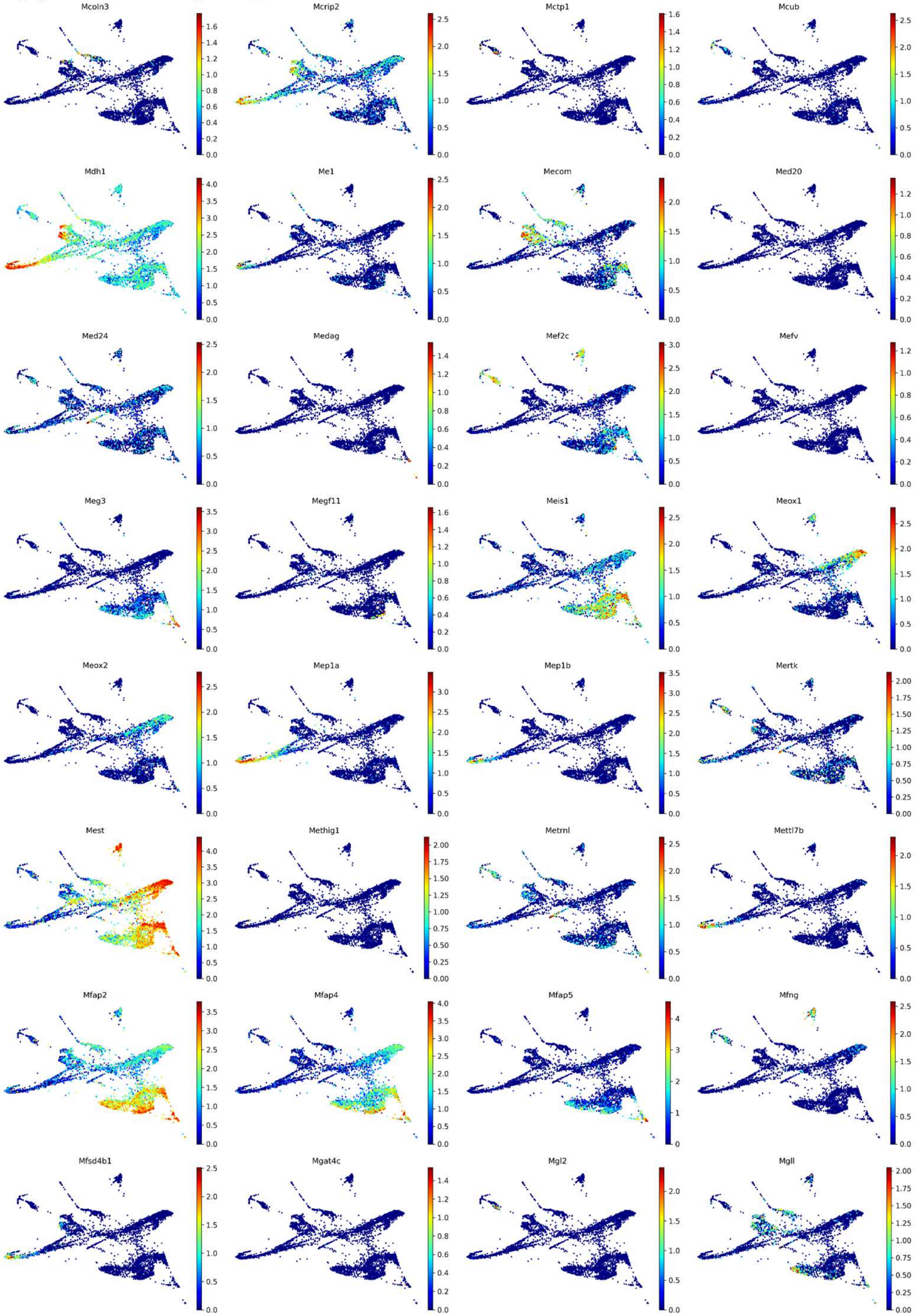

Supplementary Figure S5-58.

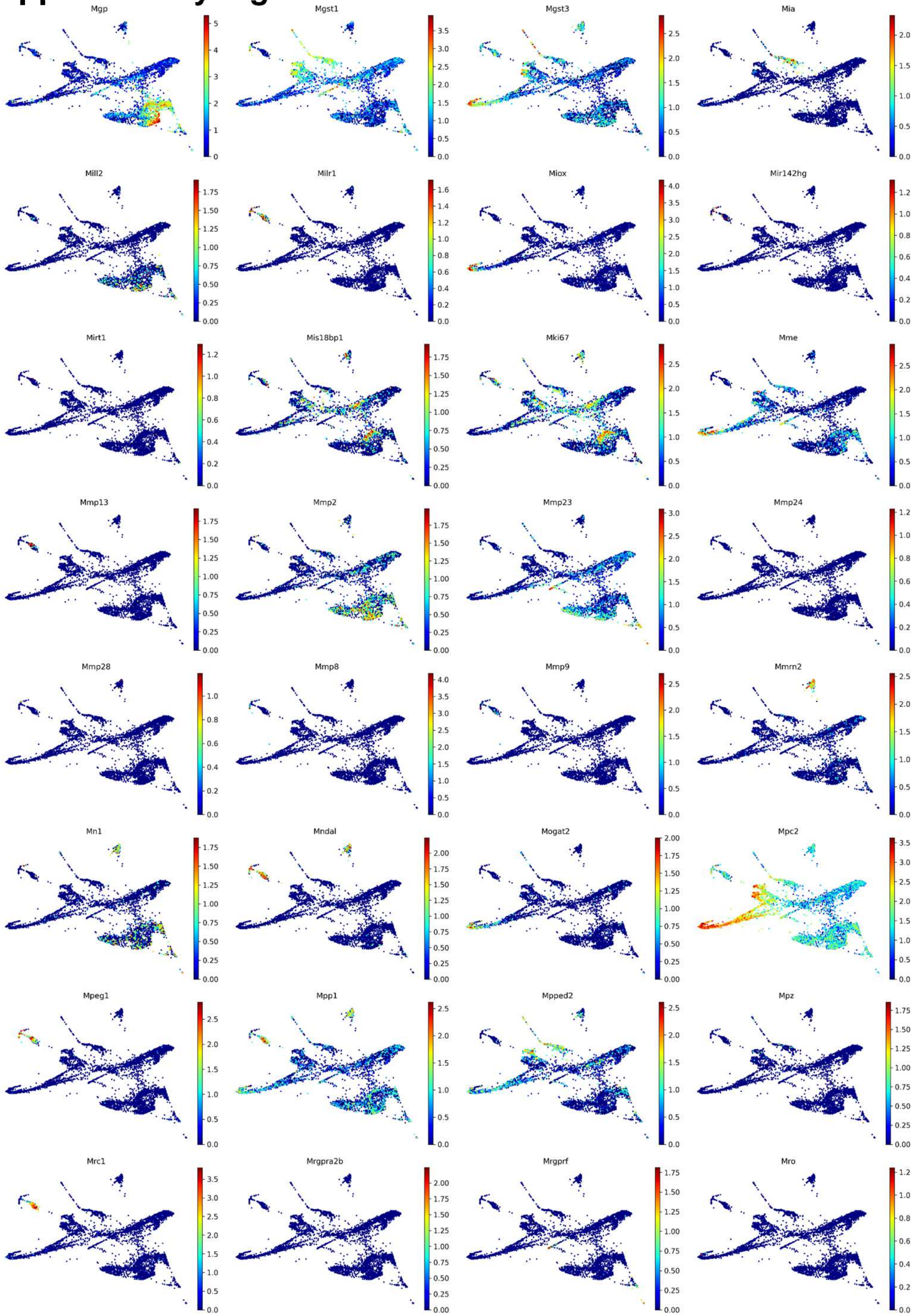

Supplementary Figure S5-59.

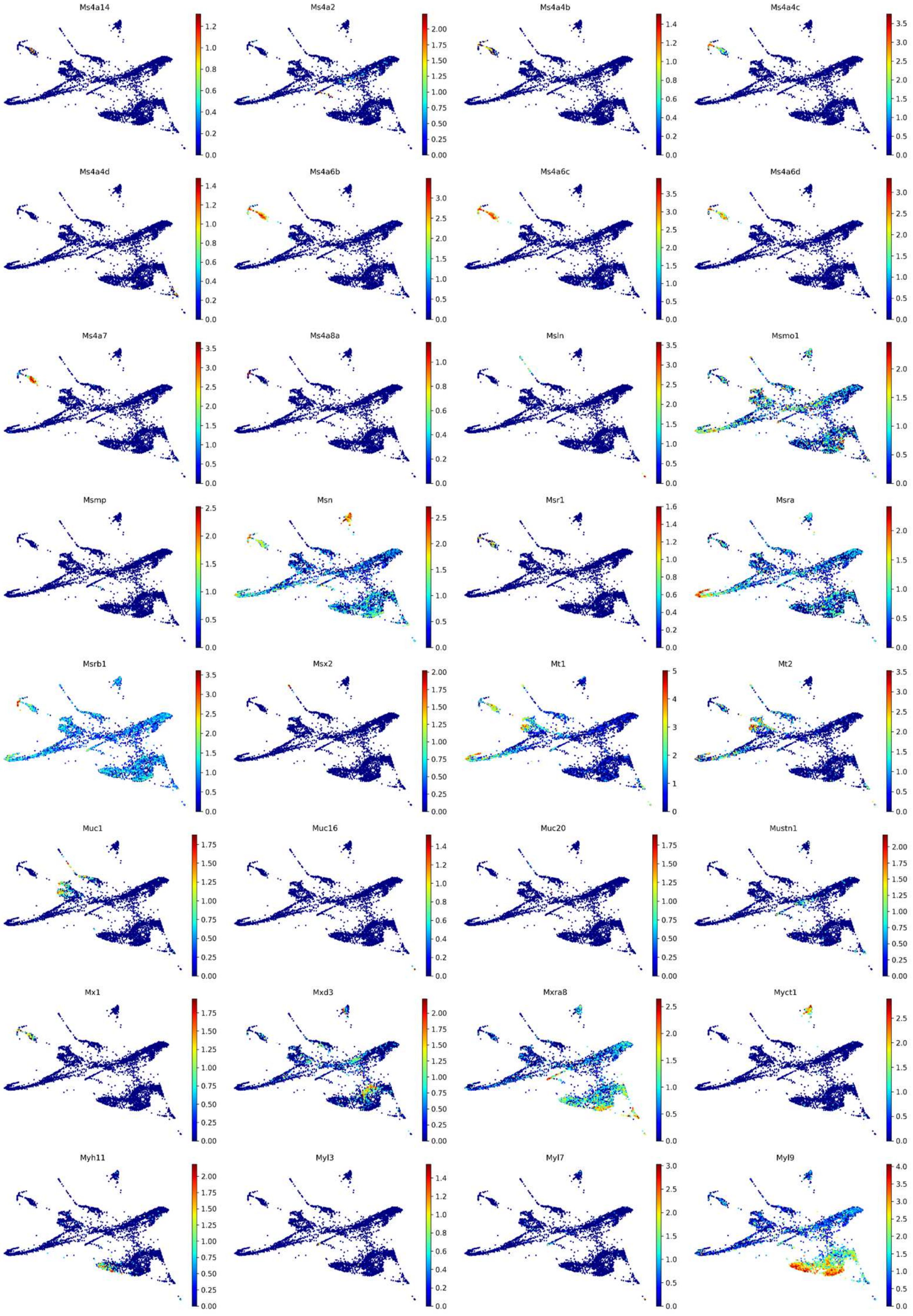

Supplementary Figure S5-60.

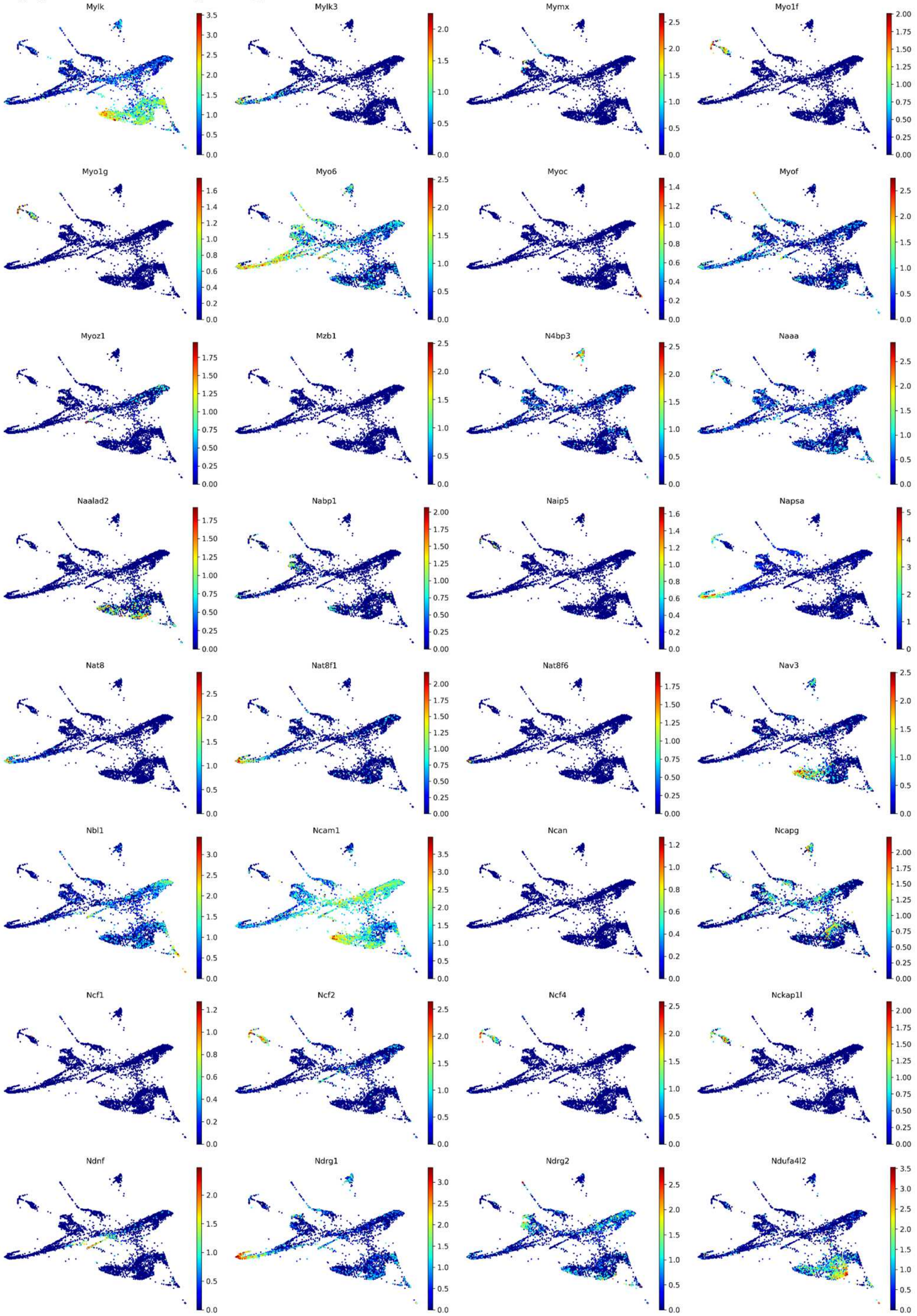

Supplement: Supplementary file 4 — Supplementary Information 4. [file 41598_2020_80154_MOESM4_ESM.pdf]
