# Supplementary material for: Single cell RNA sequencing uncovers cellular developmental sequences and novel potential intercellular communications in embryonic kidney
Source: Sci Rep. 2021 Jan 8;11:73. doi: 10.1038/s41598-020-80154-y (PMC7794461; doi:10.1038/s41598-020-80154-y)
Supplement: Supplementary file 5 — Supplementary Information 5. [file 41598_2020_80154_MOESM5_ESM.pdf]

Supplementary Figure S5-61.

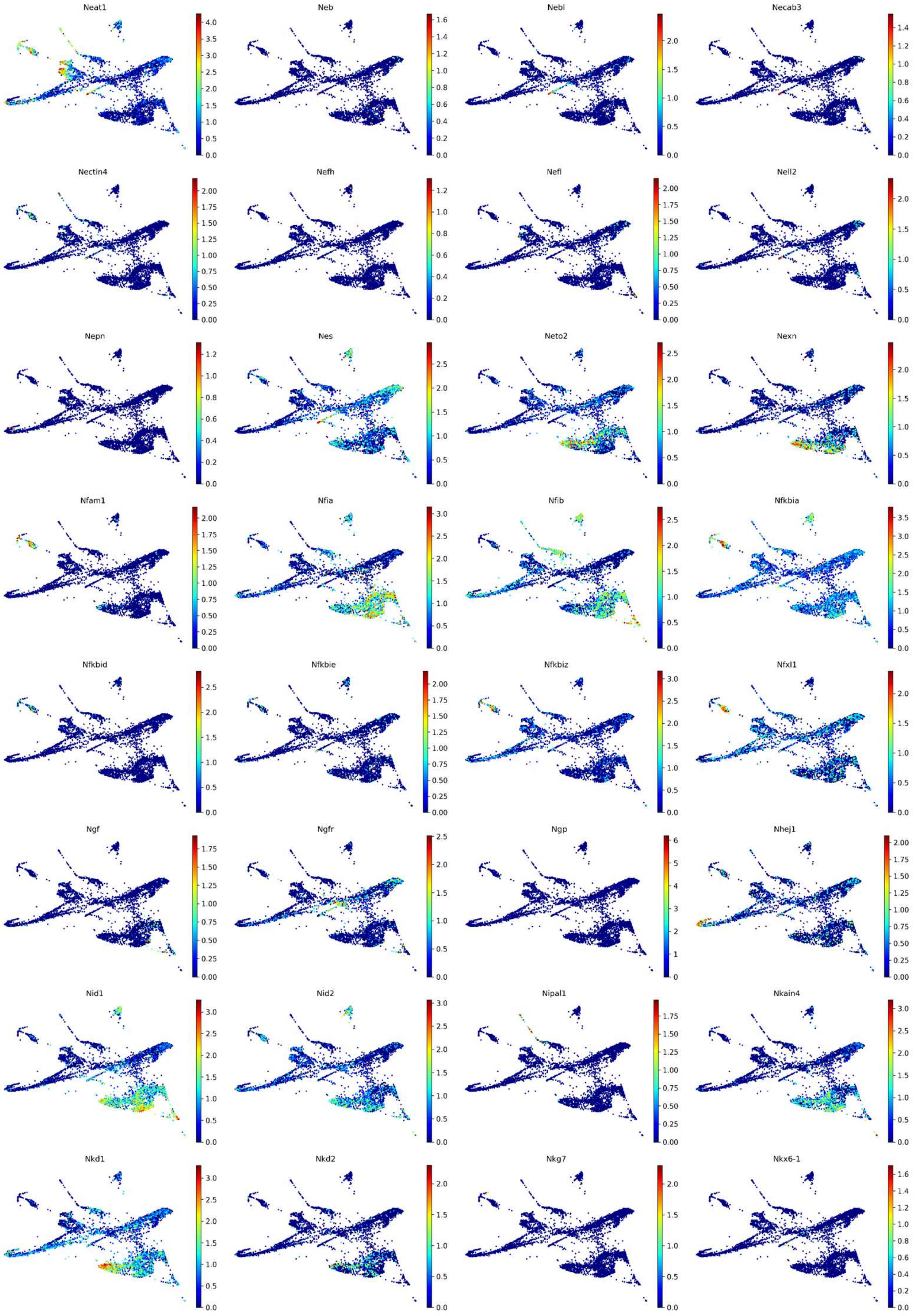

Supplementary Figure S5-62.

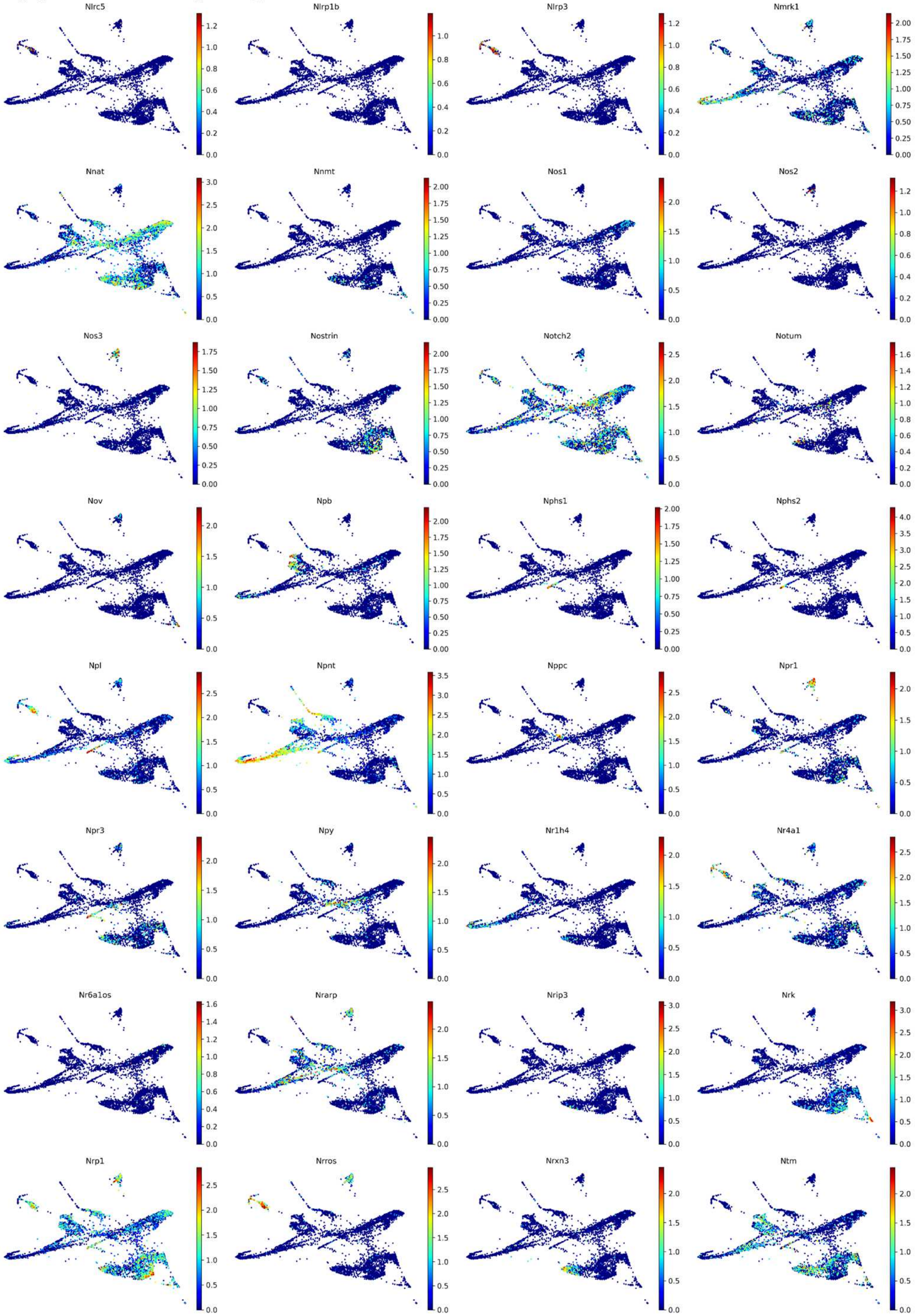

Supplementary Figure S5-63.

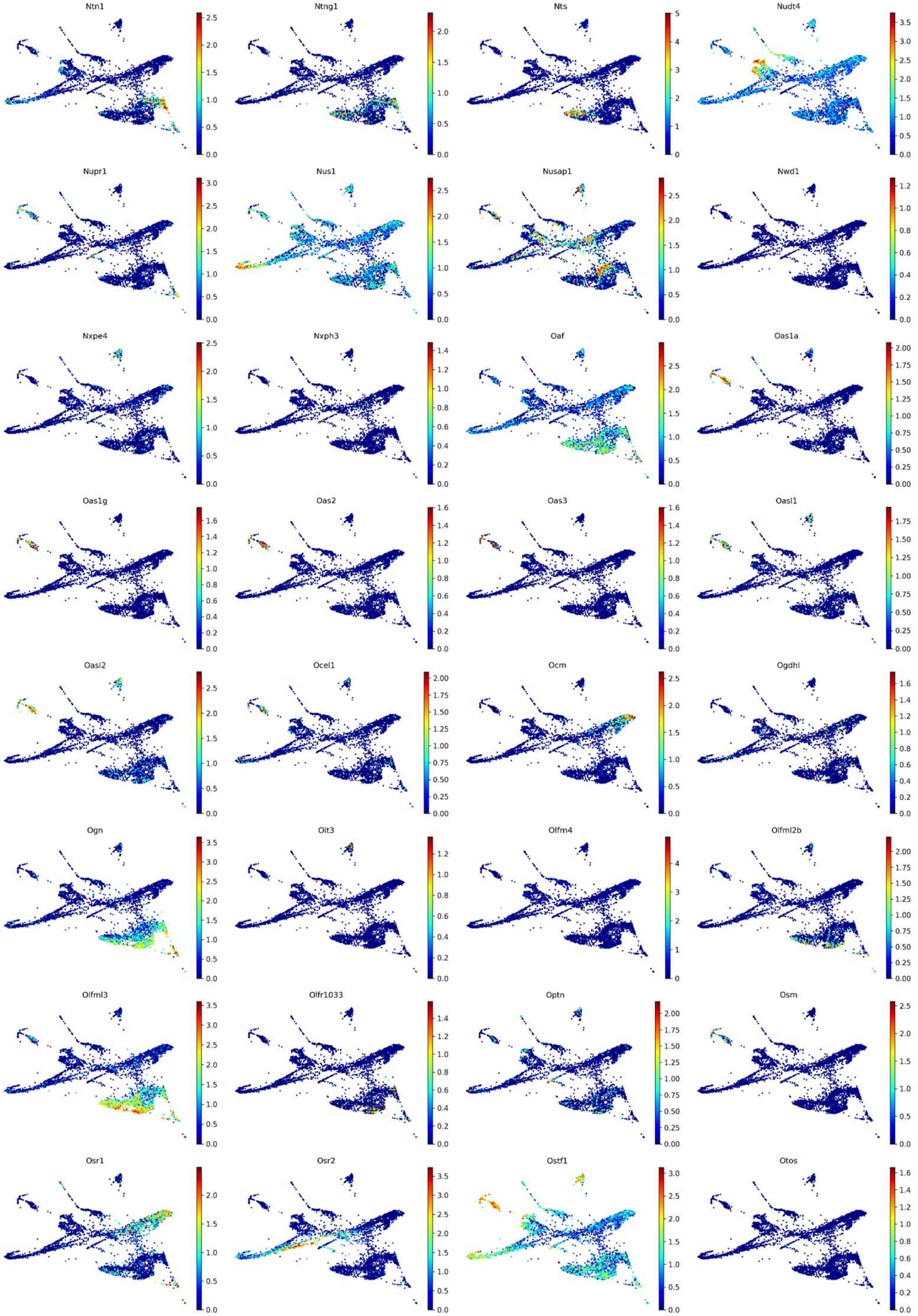

Supplementary Figure S5-64.

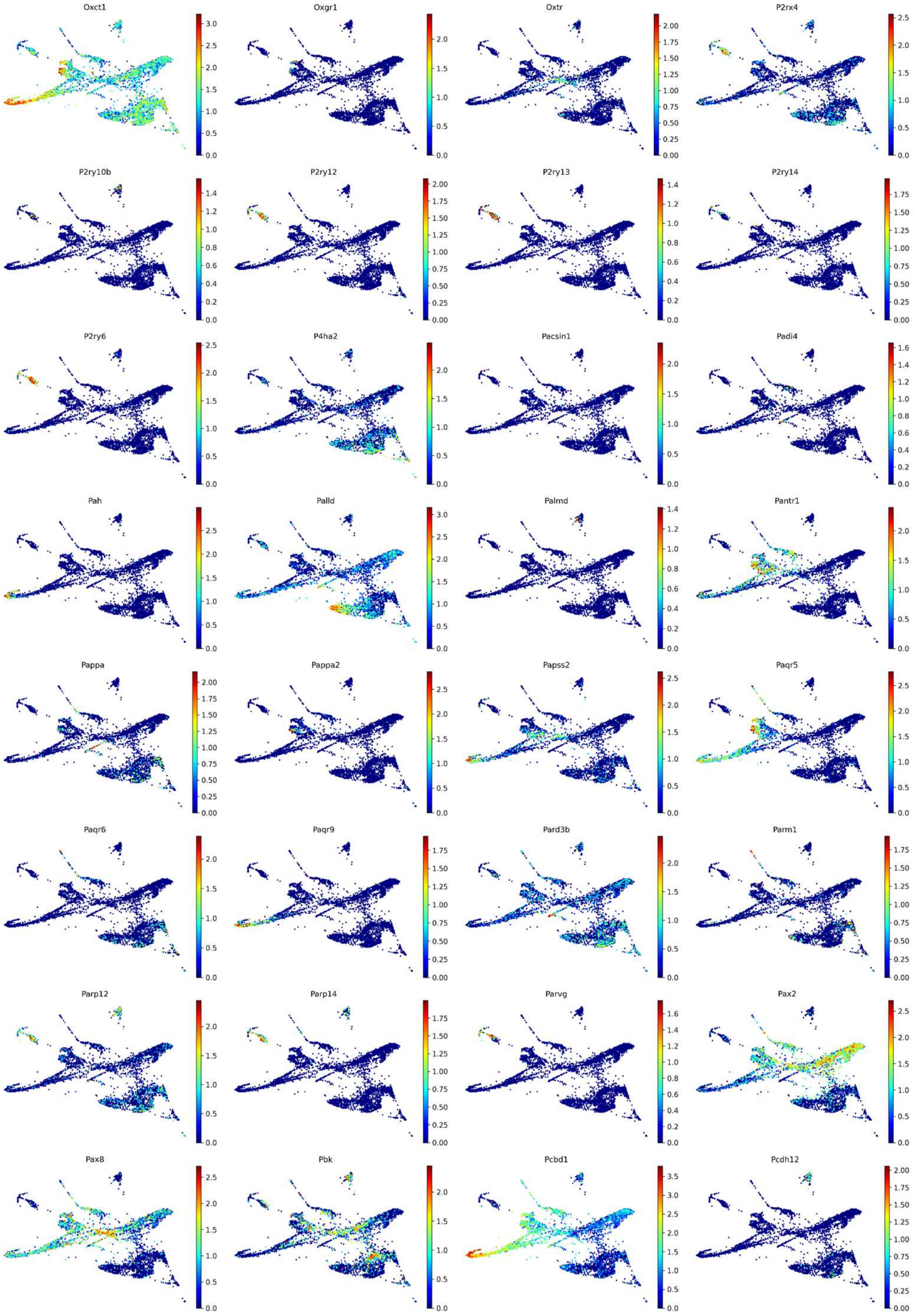

Supplementary Figure S5-65.

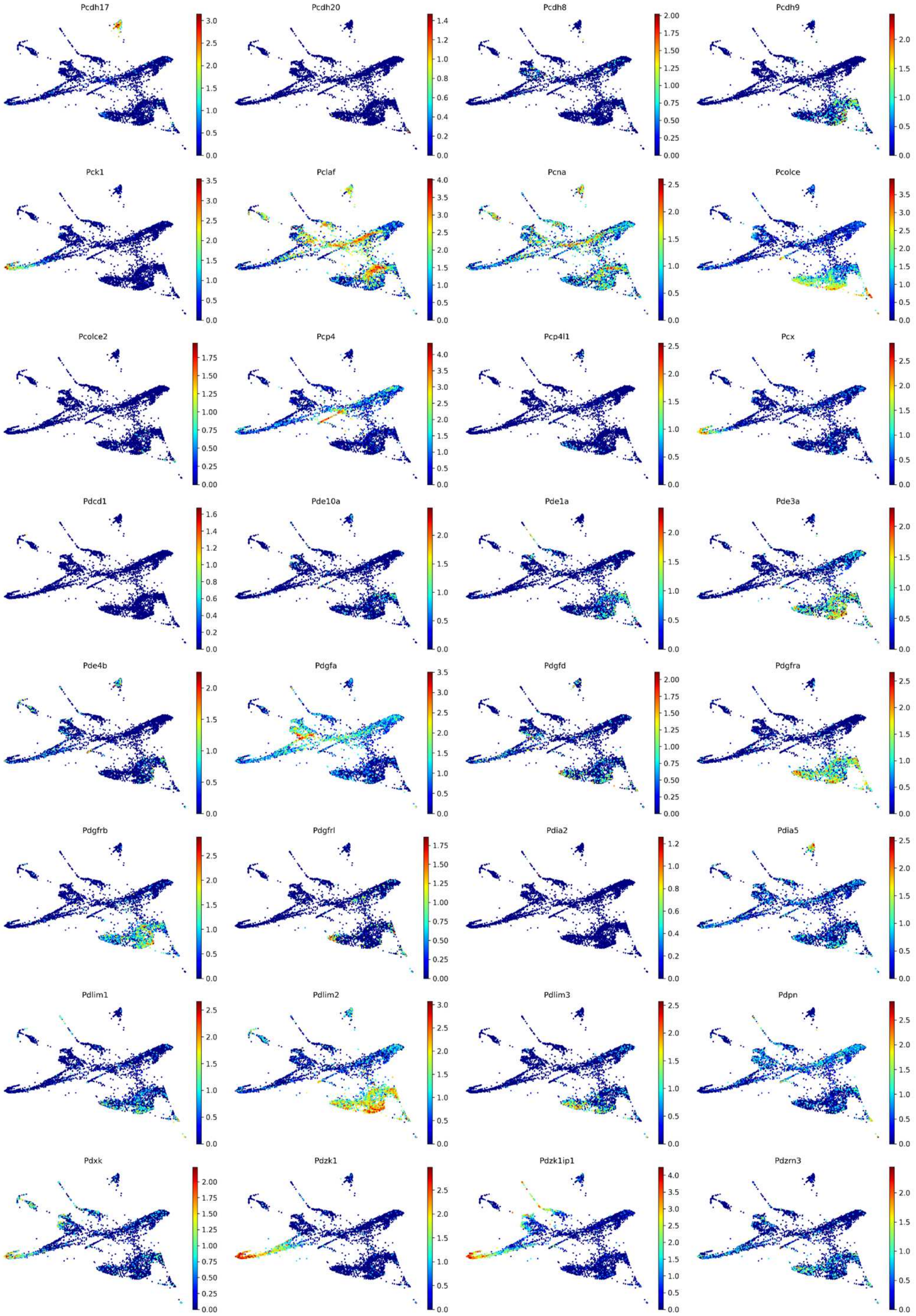

Supplementary Figure S5-66.

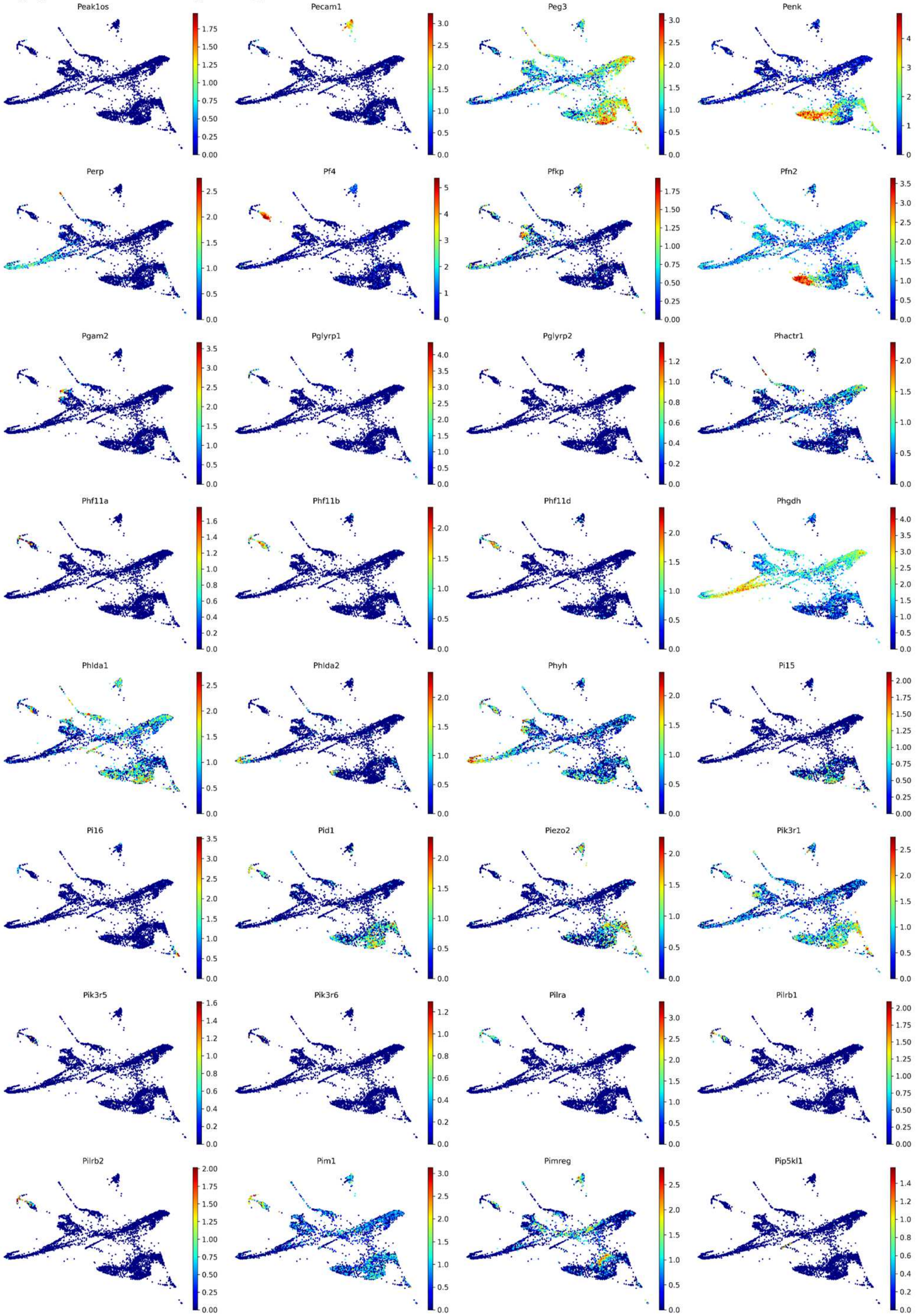

Supplementary Figure S5-67.

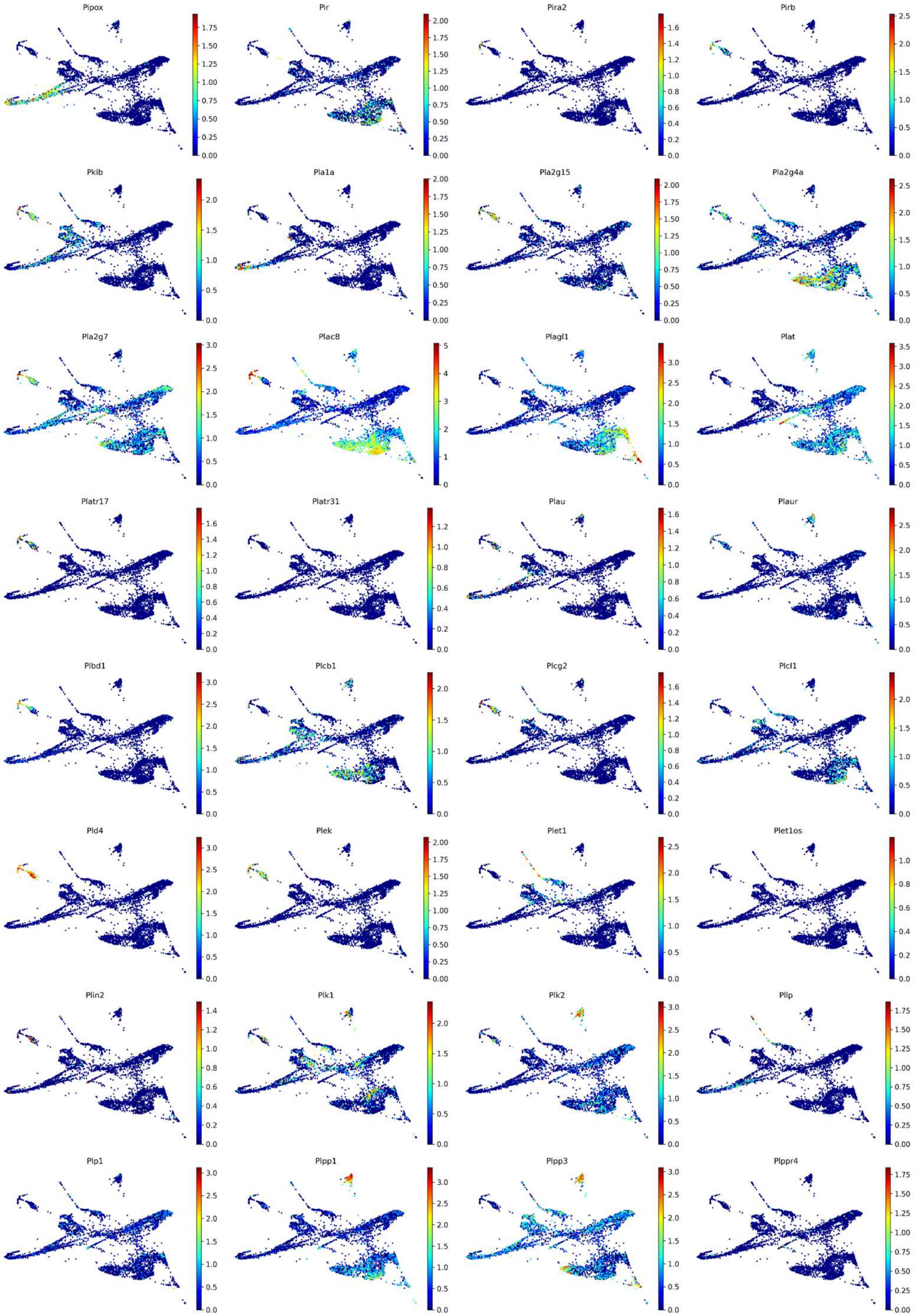

Supplementary Figure S5-68.

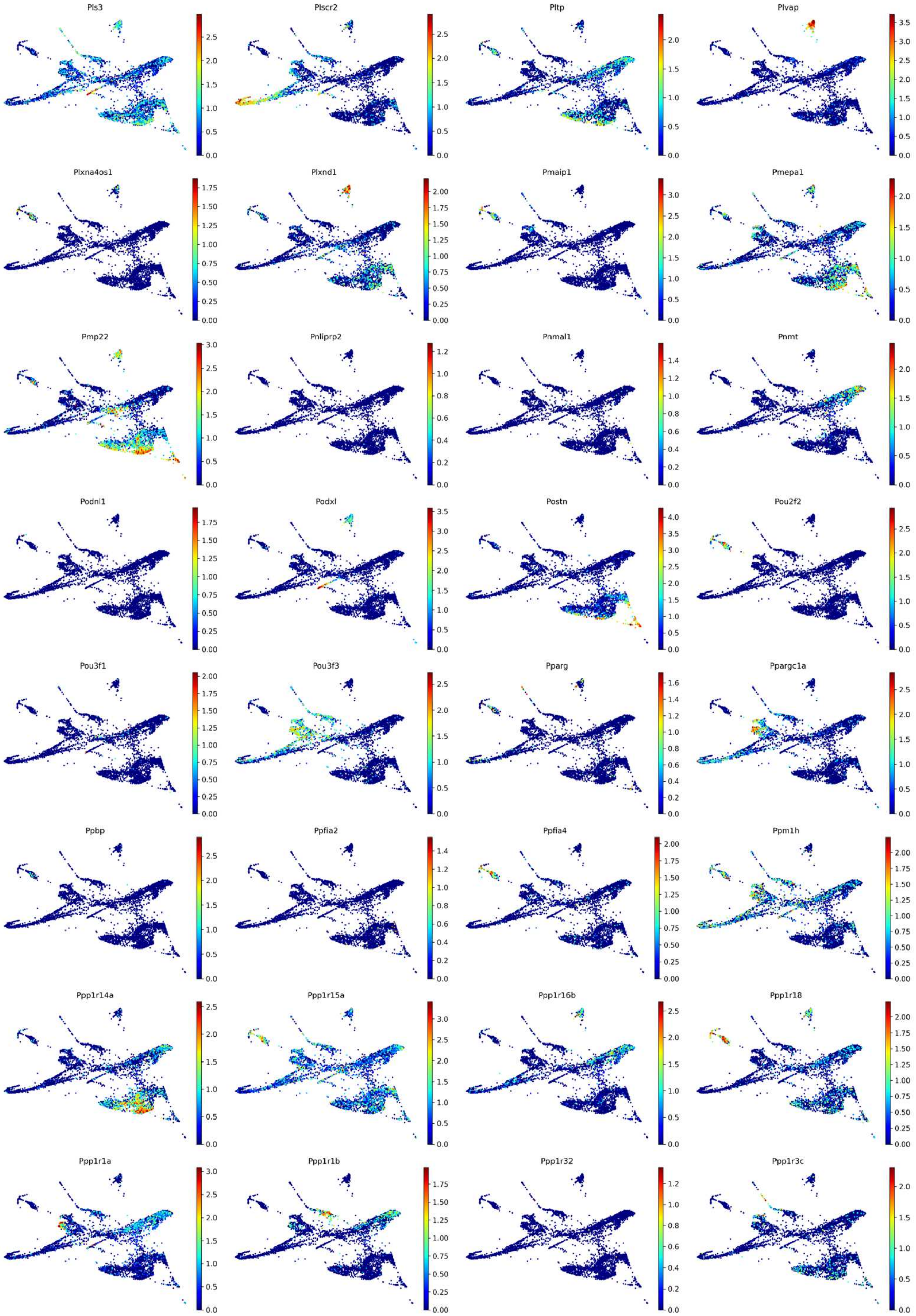

Supplementary Figure S5-69.

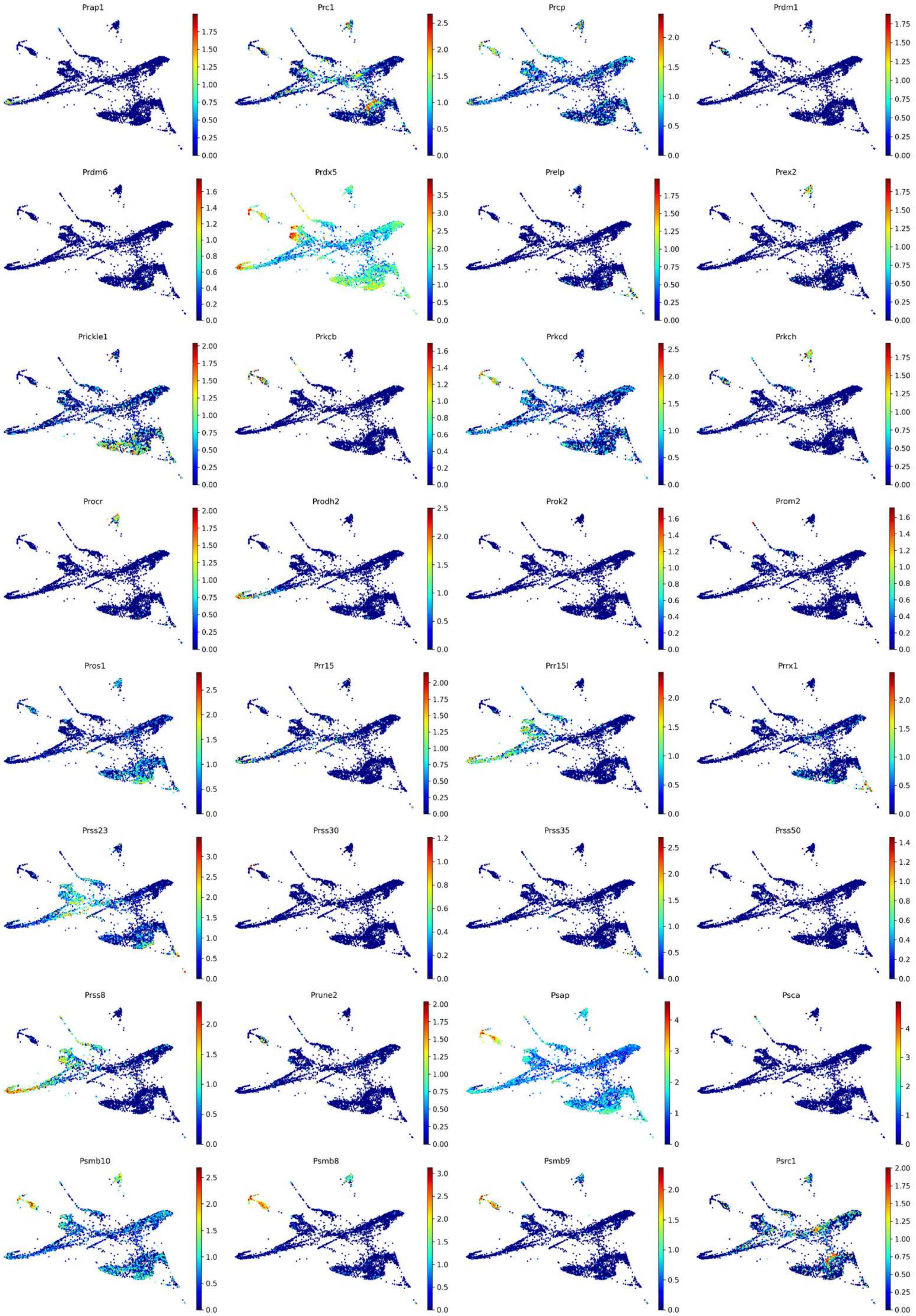

Supplementary Figure S5-70.

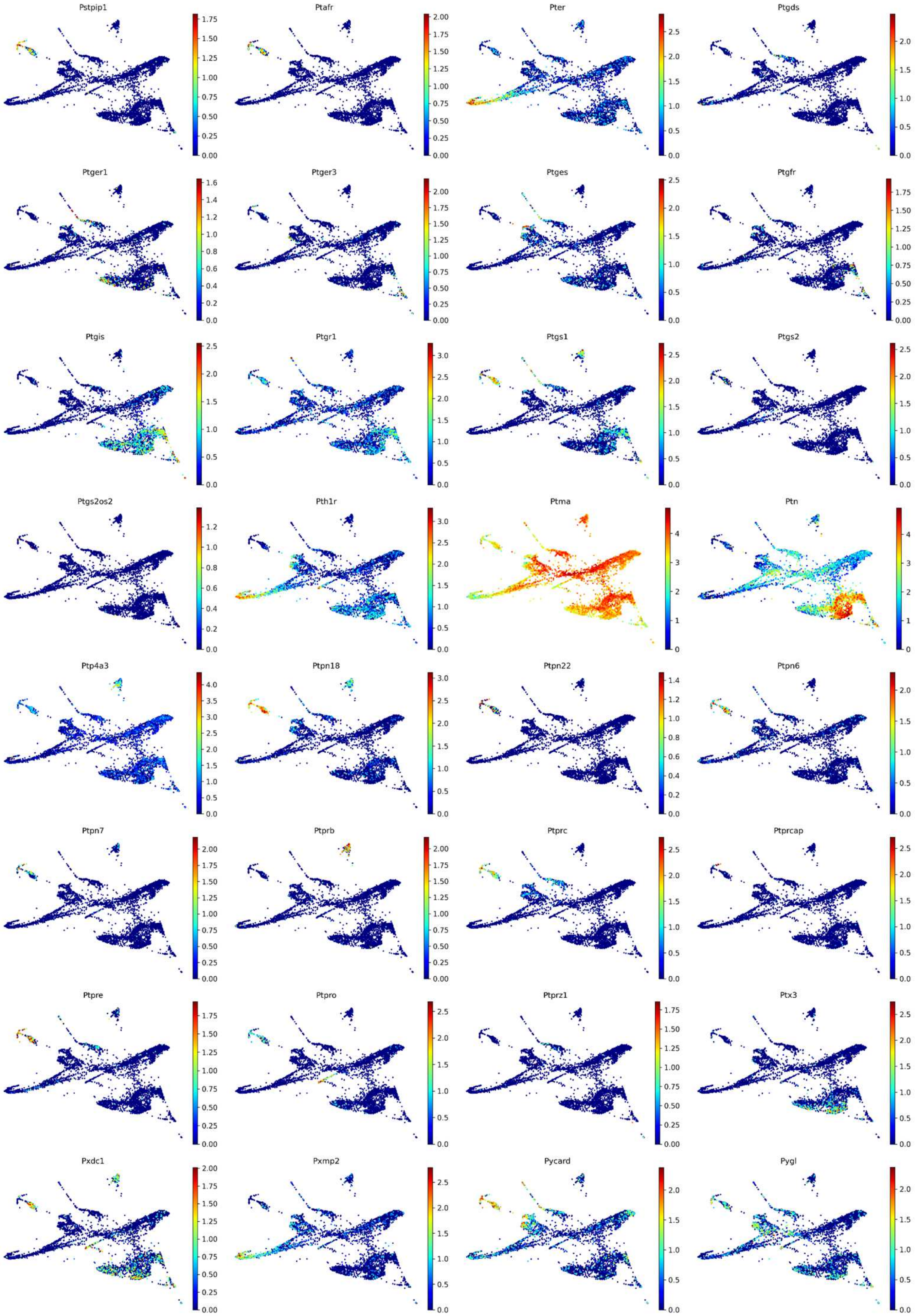

Supplementary Figure S5-71.

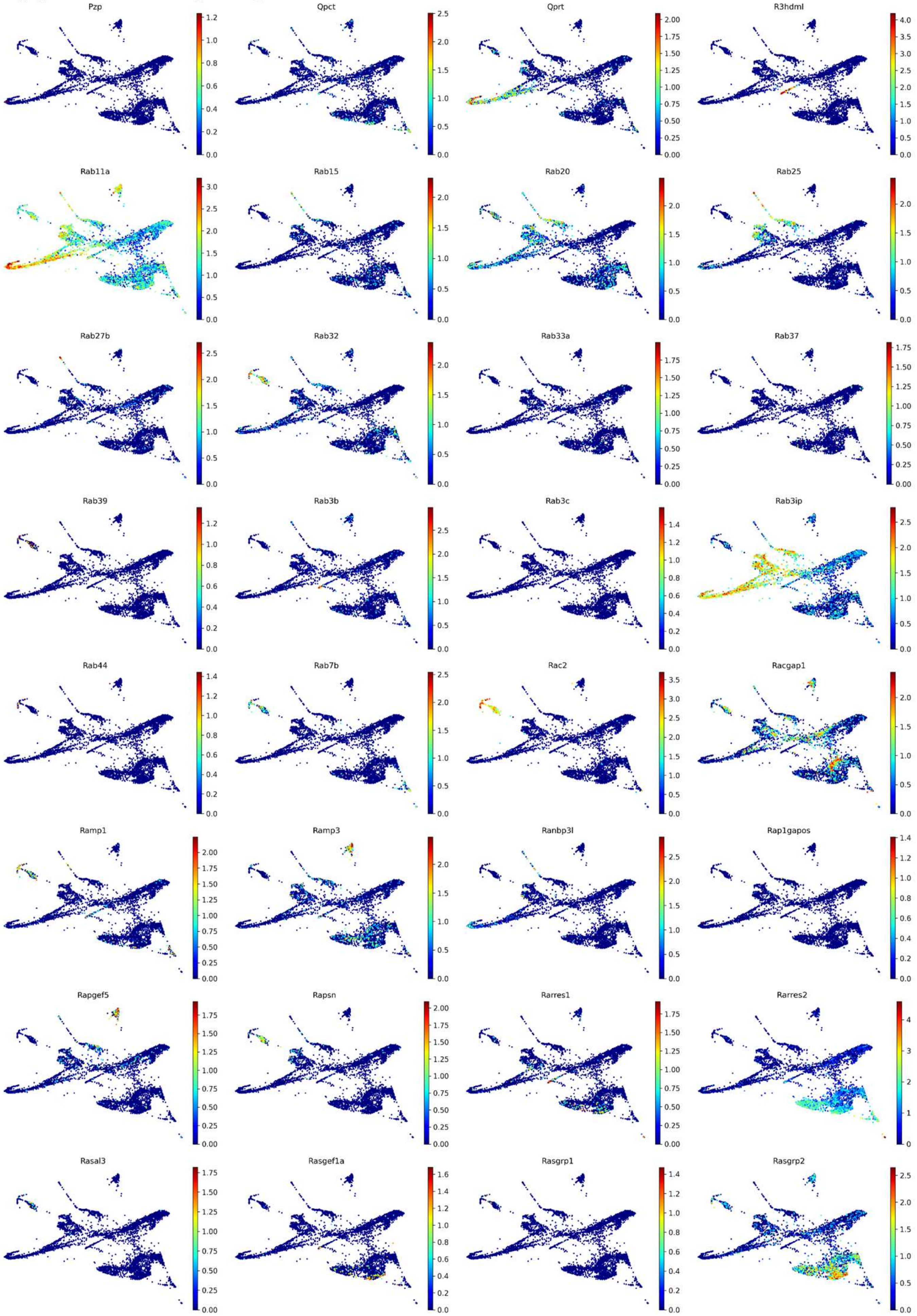

Supplementary Figure S5-72.

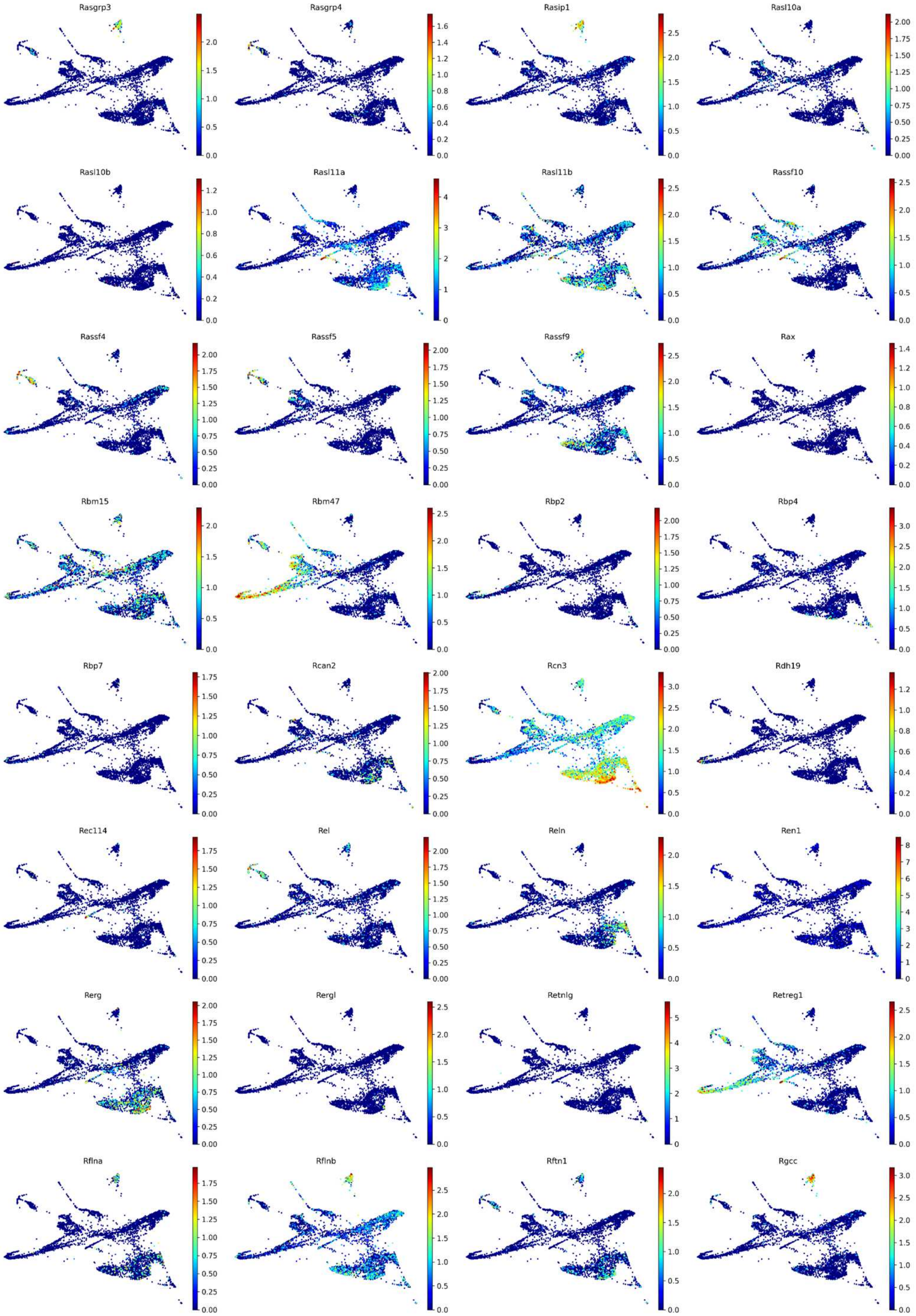

Supplementary Figure S5-73.

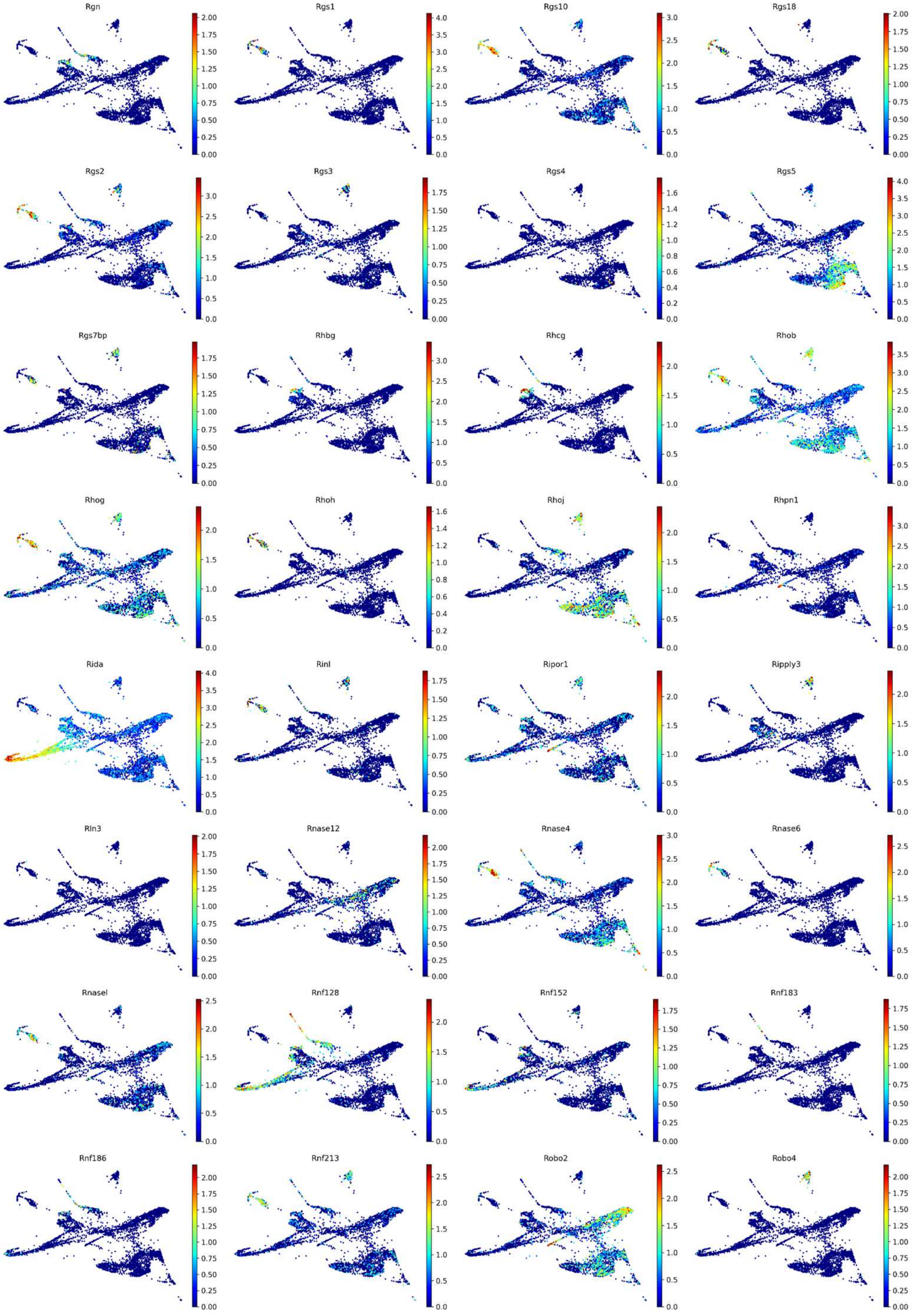

Supplementary Figure S5-74.

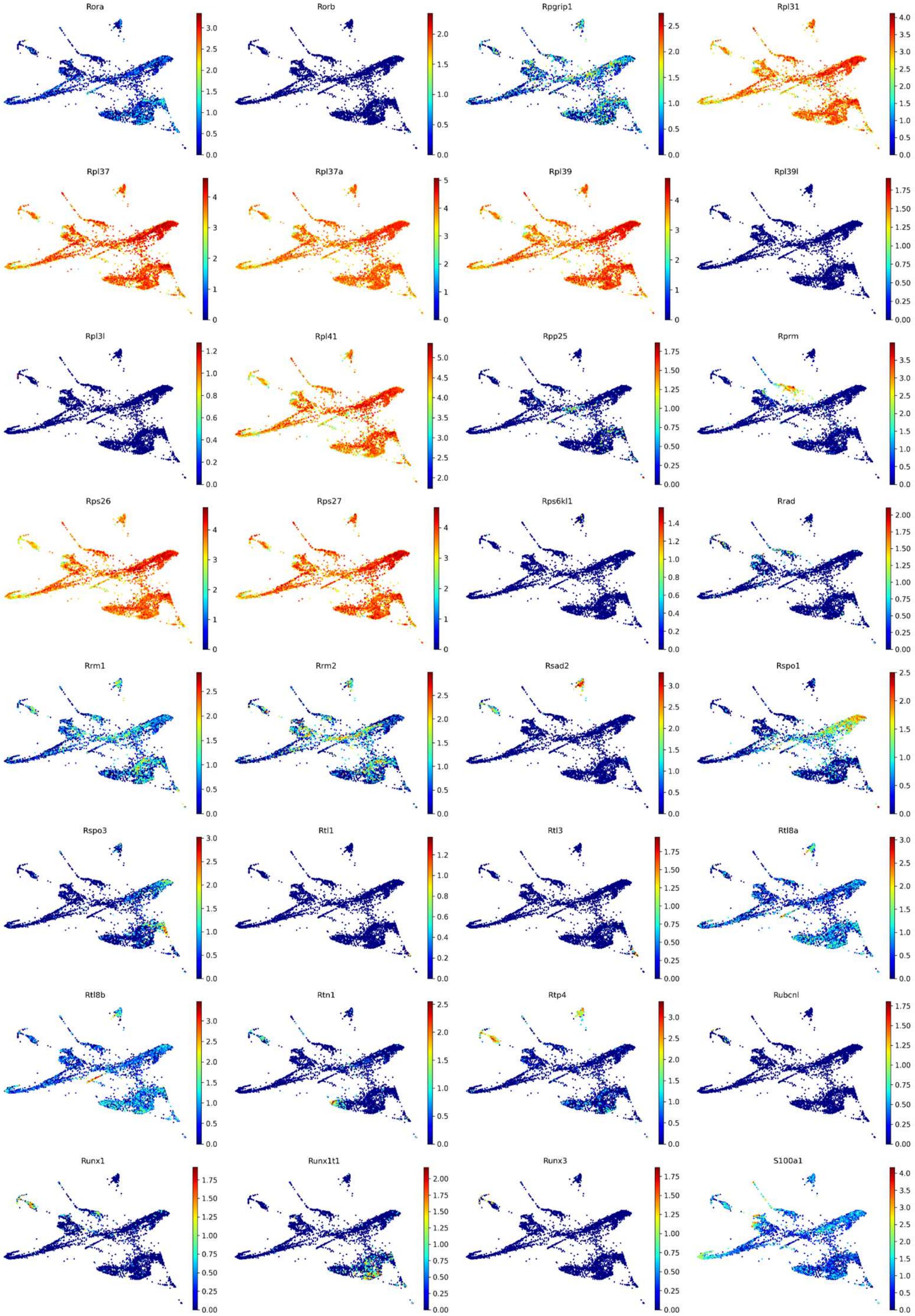

Supplementary Figure S5-75.

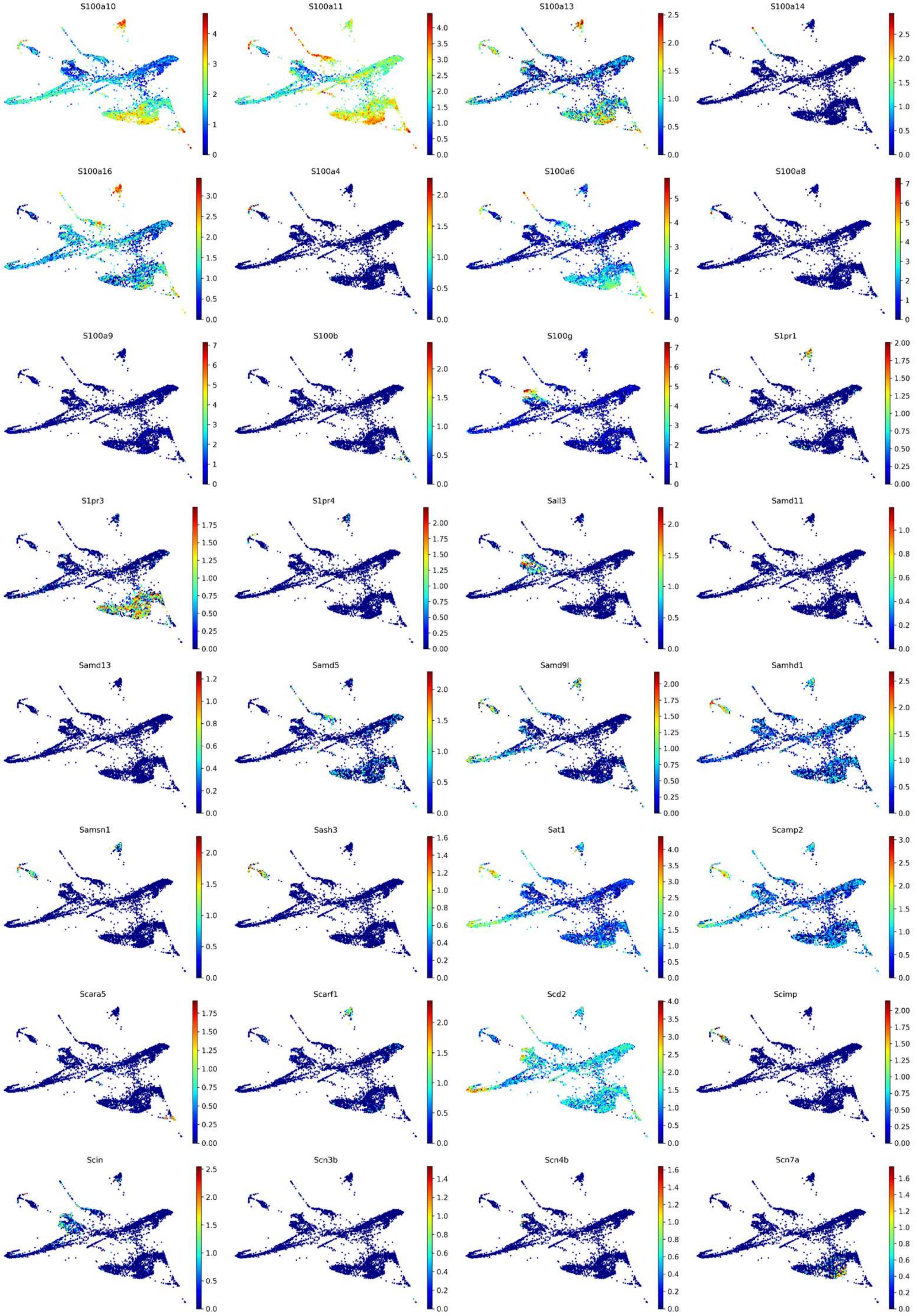

Supplementary Figure S5-76.

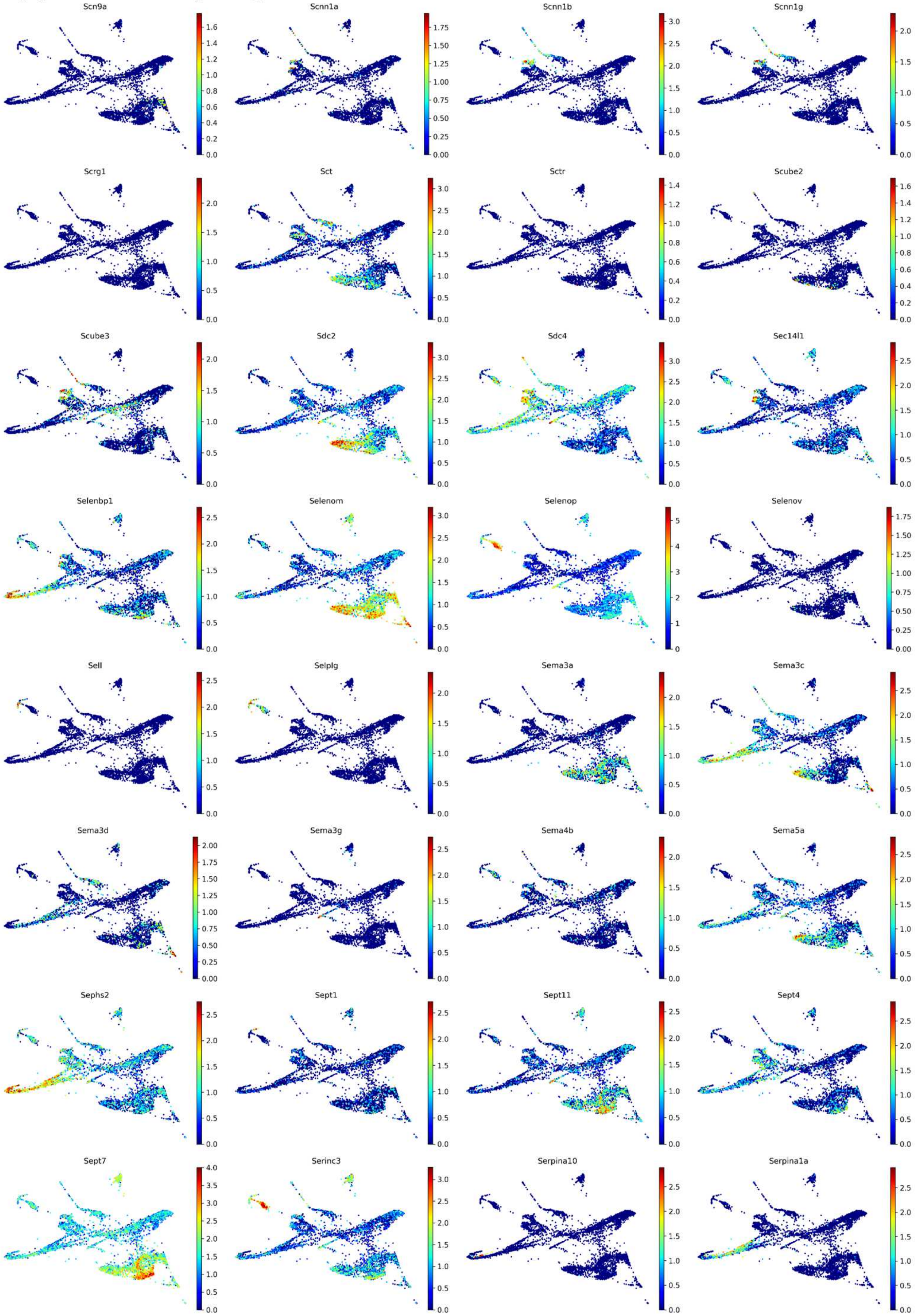

Supplementary Figure S5-77.

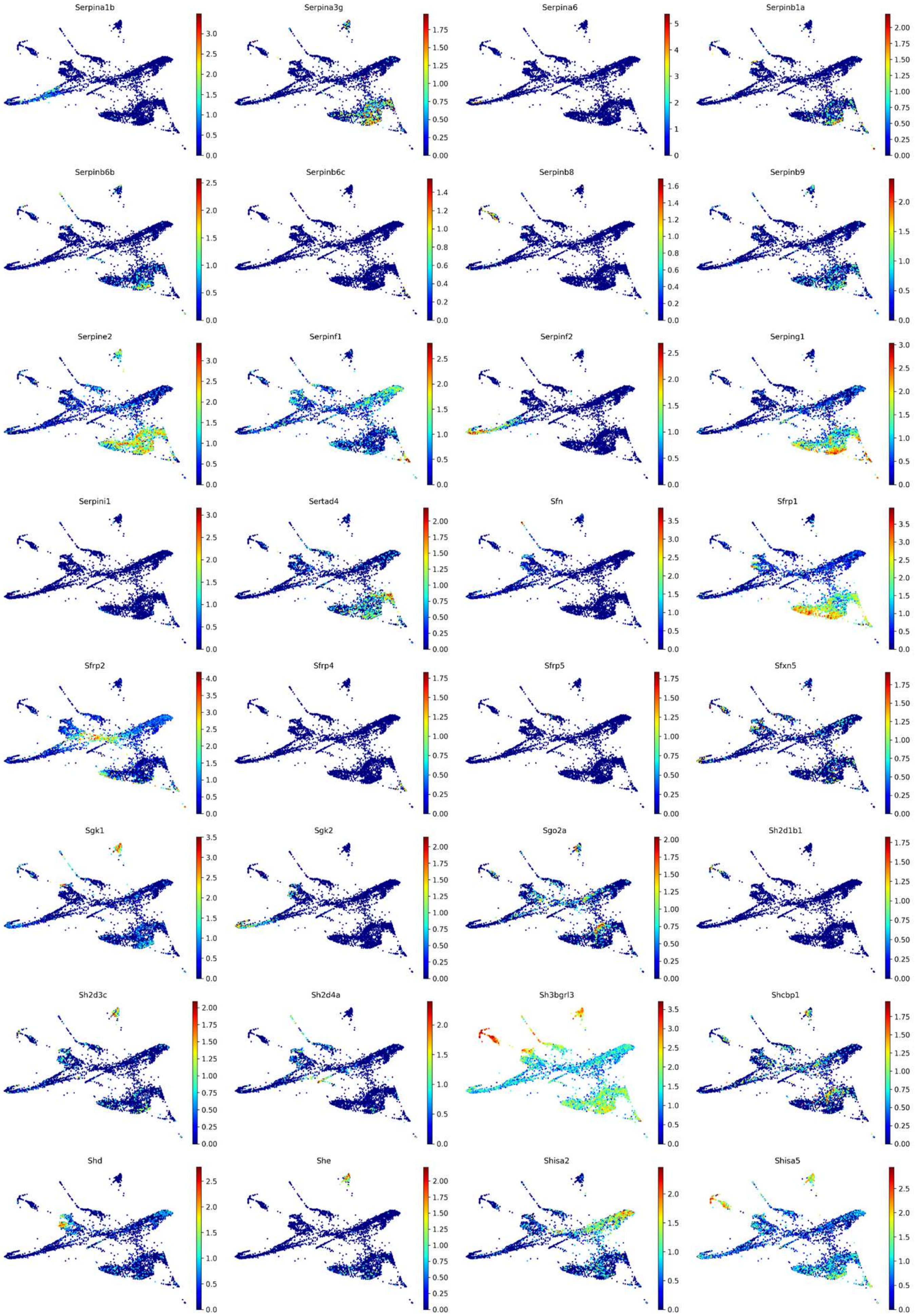

Supplementary Figure S5-78.

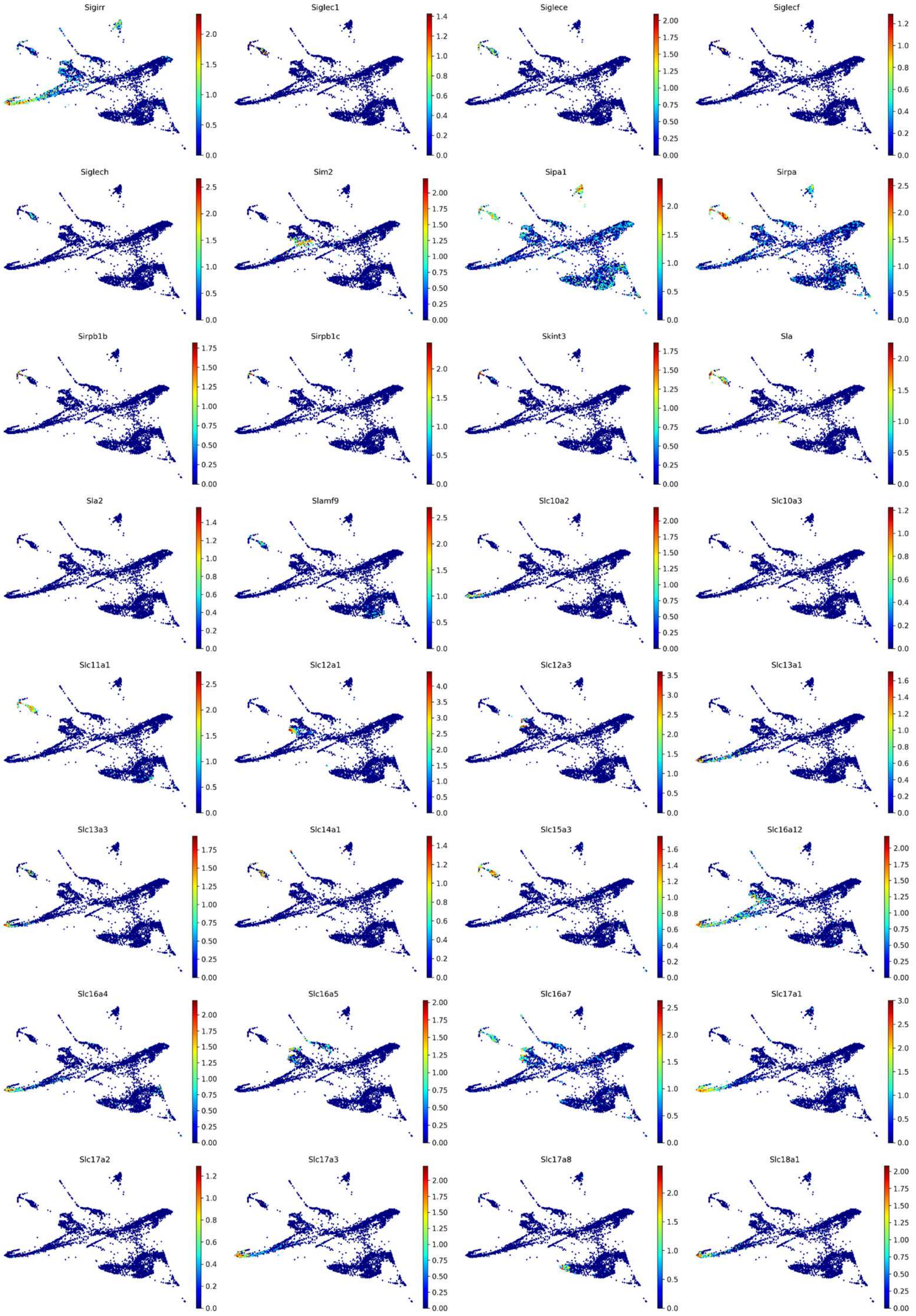

Supplementary Figure S5-79.

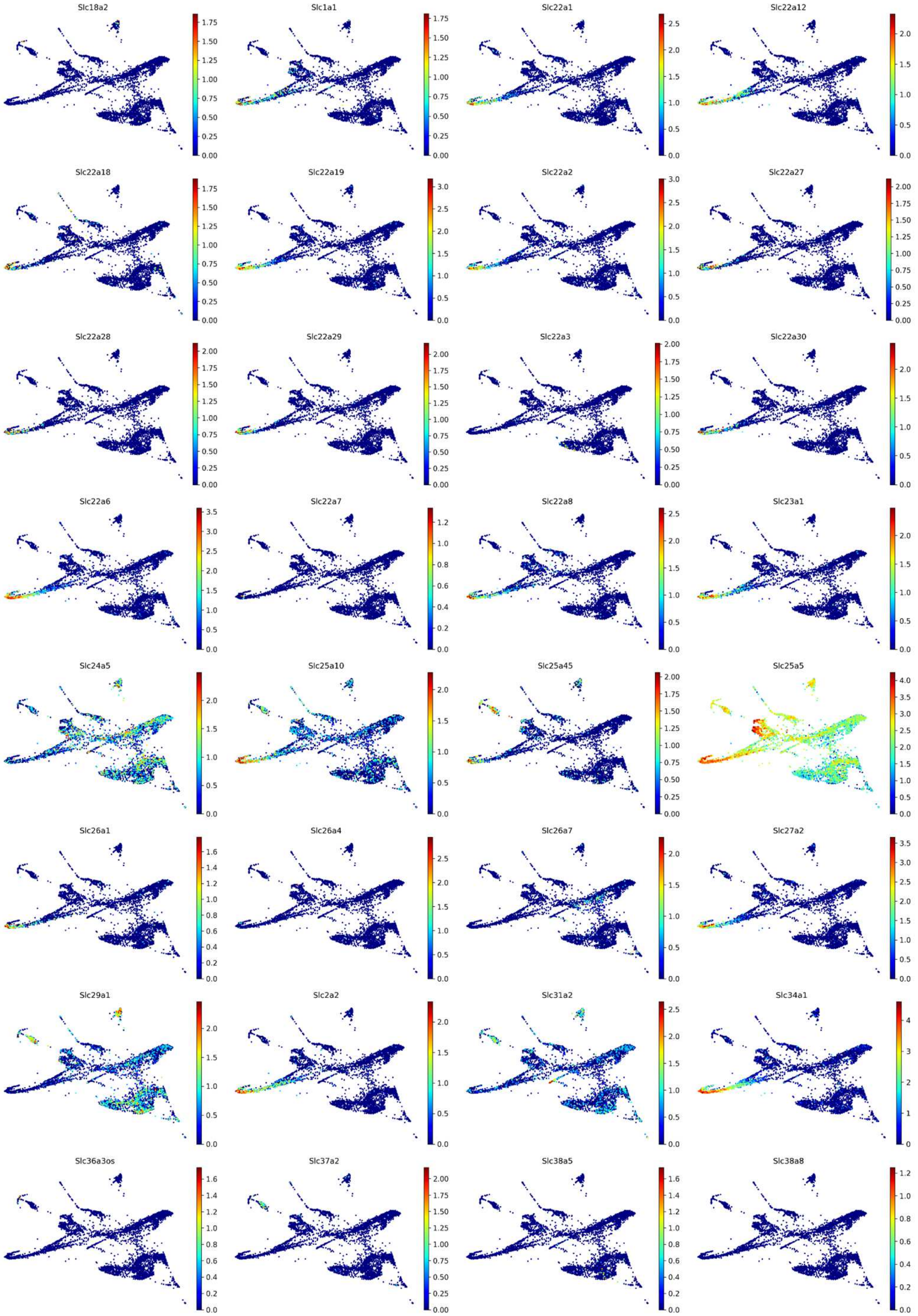

Supplementary Figure S5-80.

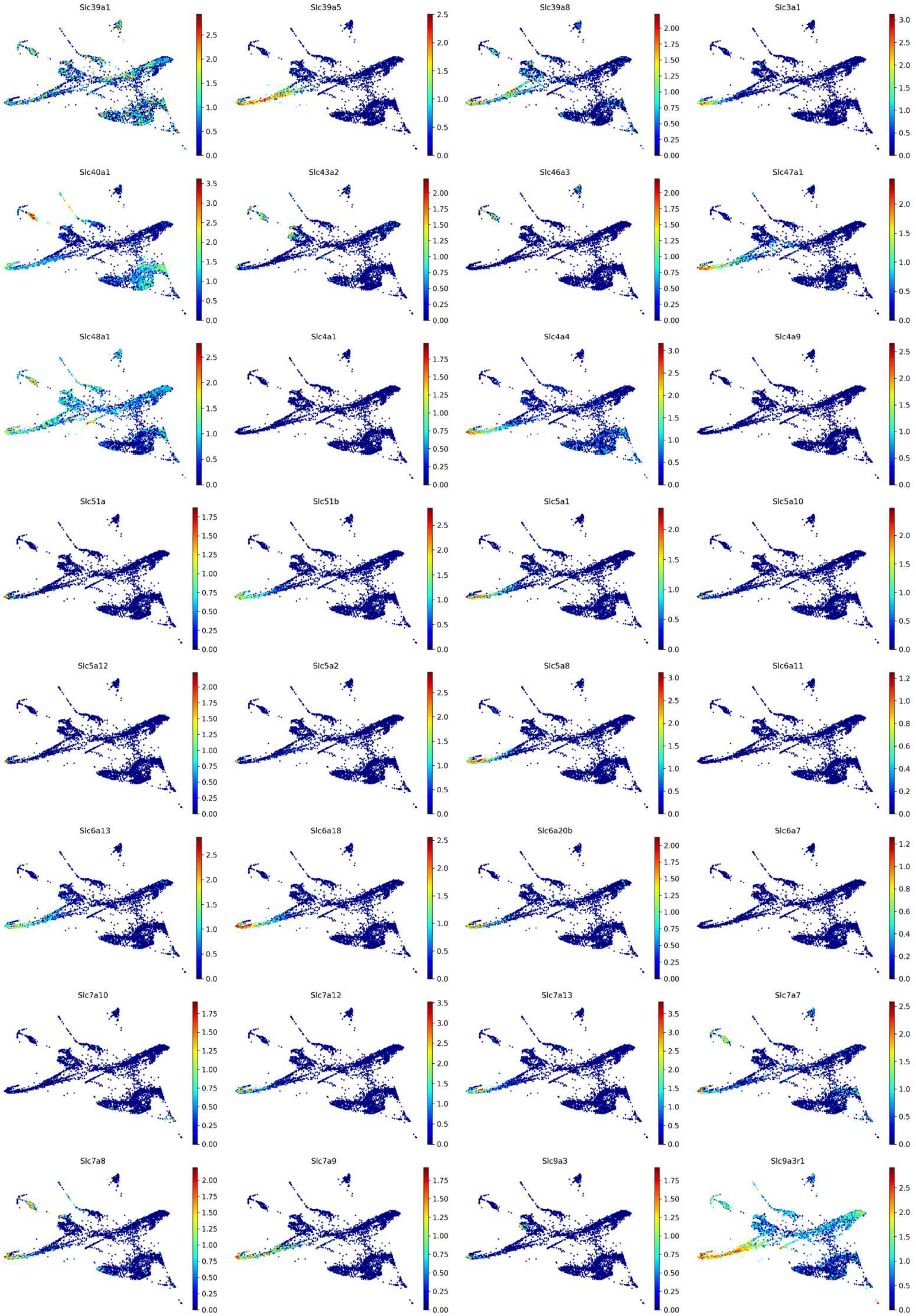

Supplementary Figure S5-81.

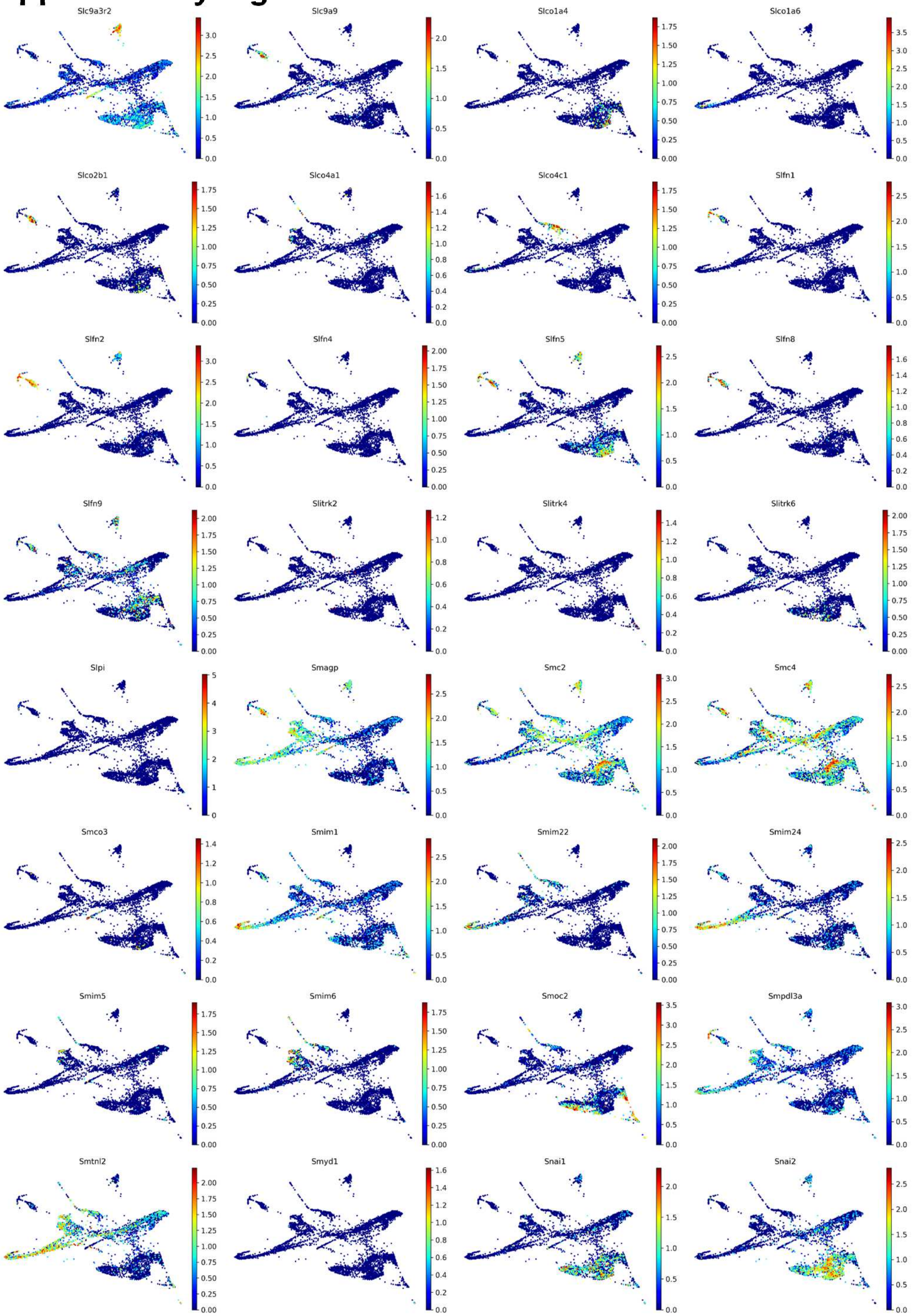

Supplementary Figure S5-82.

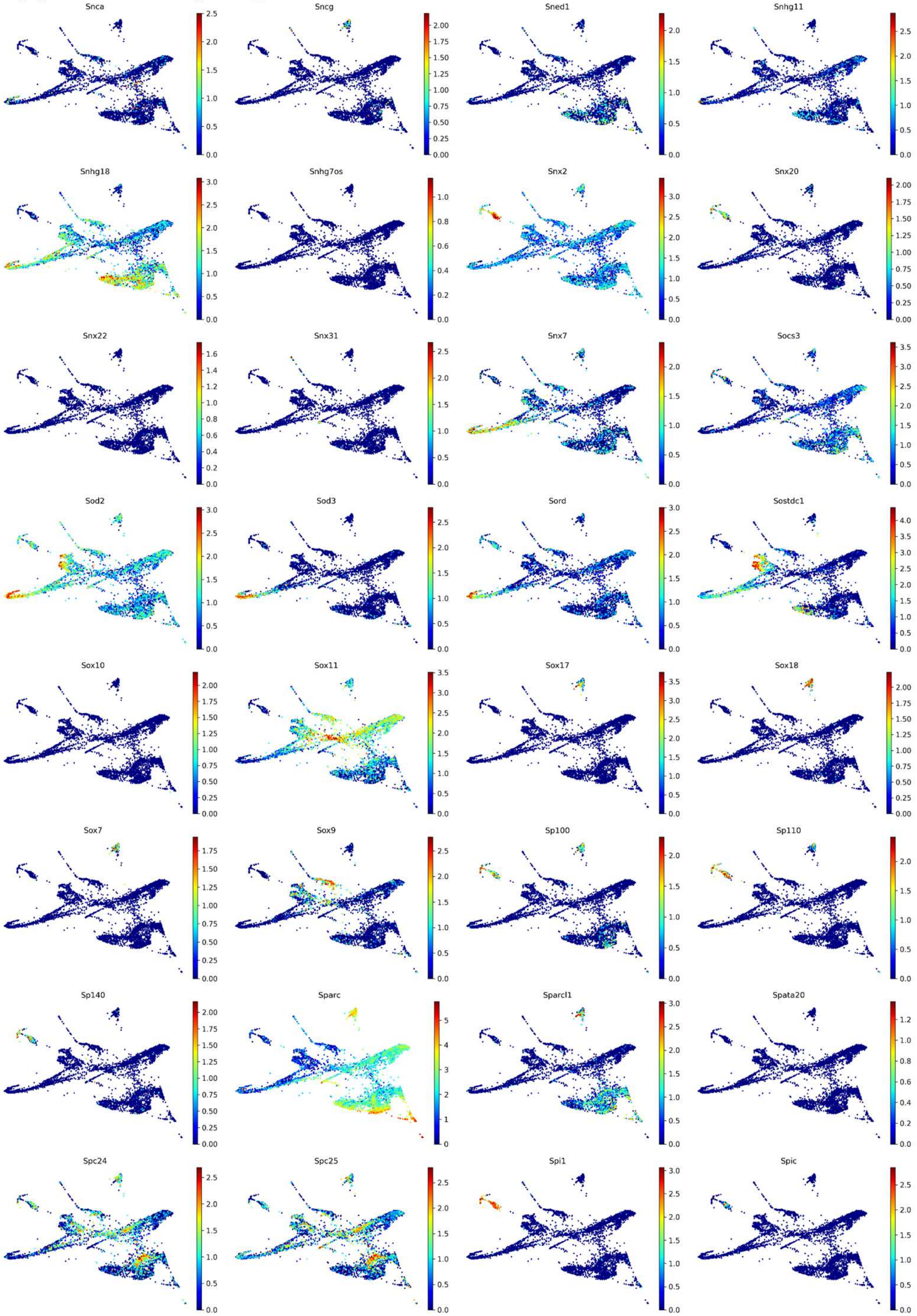

Supplementary Figure S5-83.

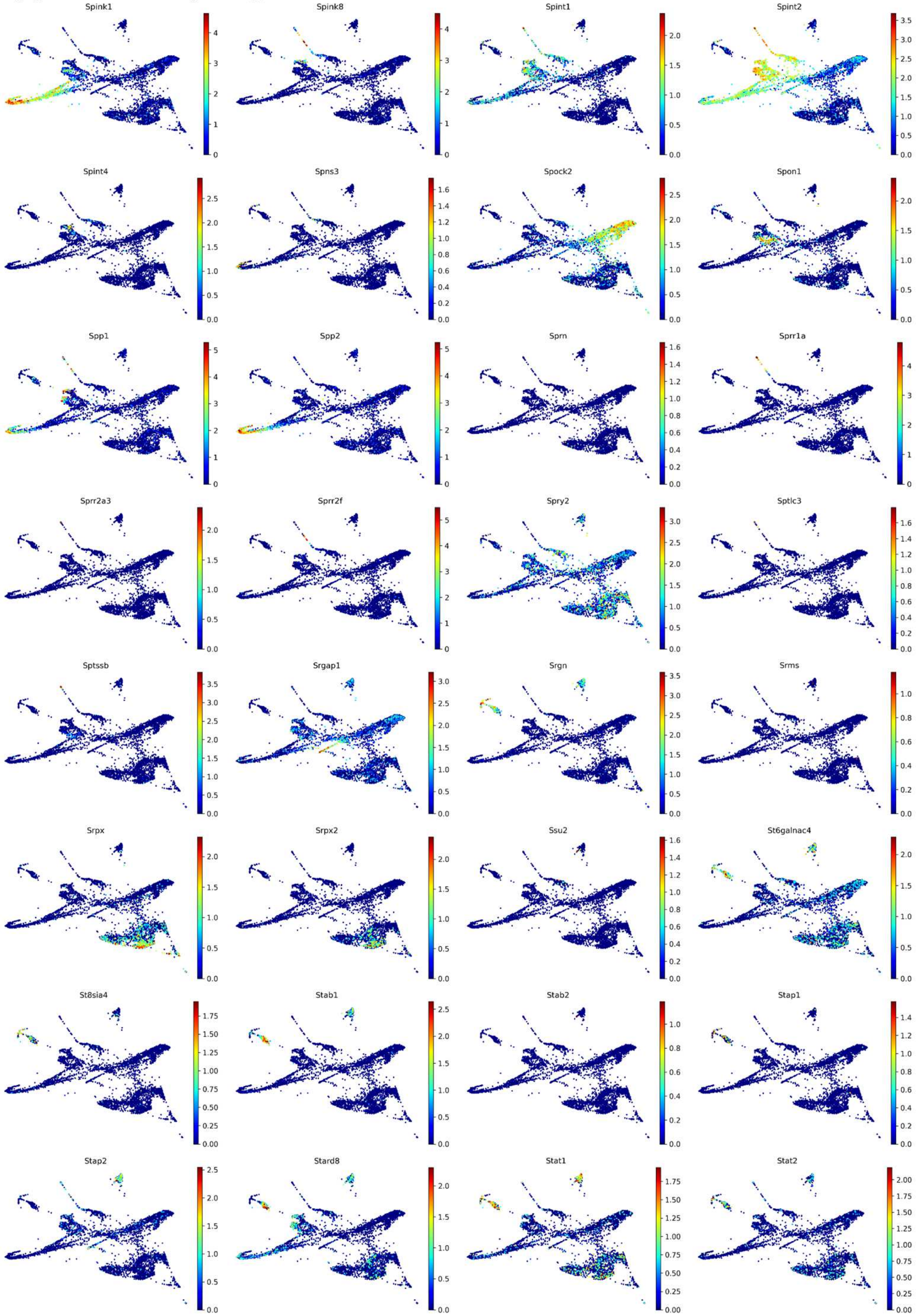

Supplementary Figure S5-84.

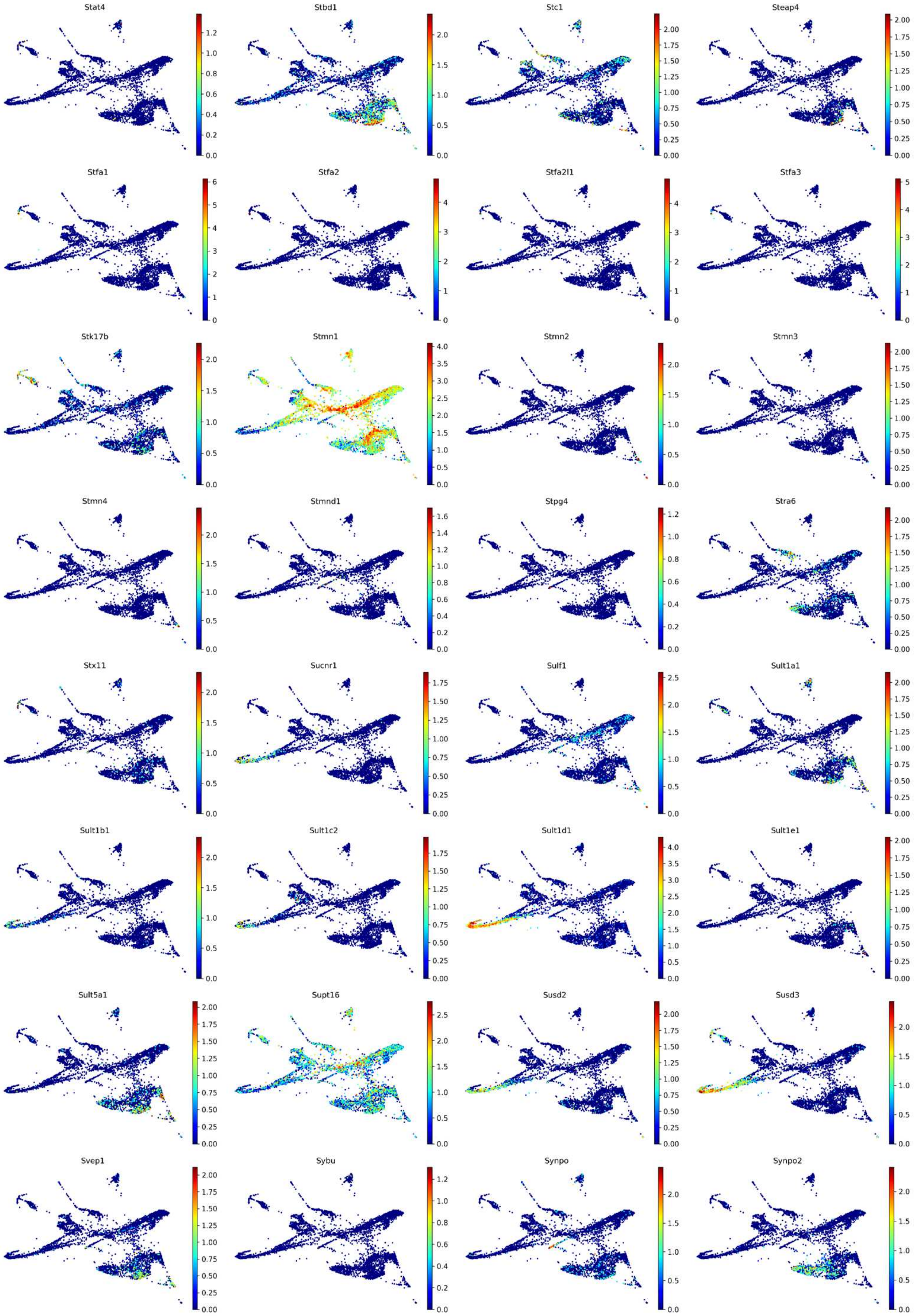

Supplementary Figure S5-85.

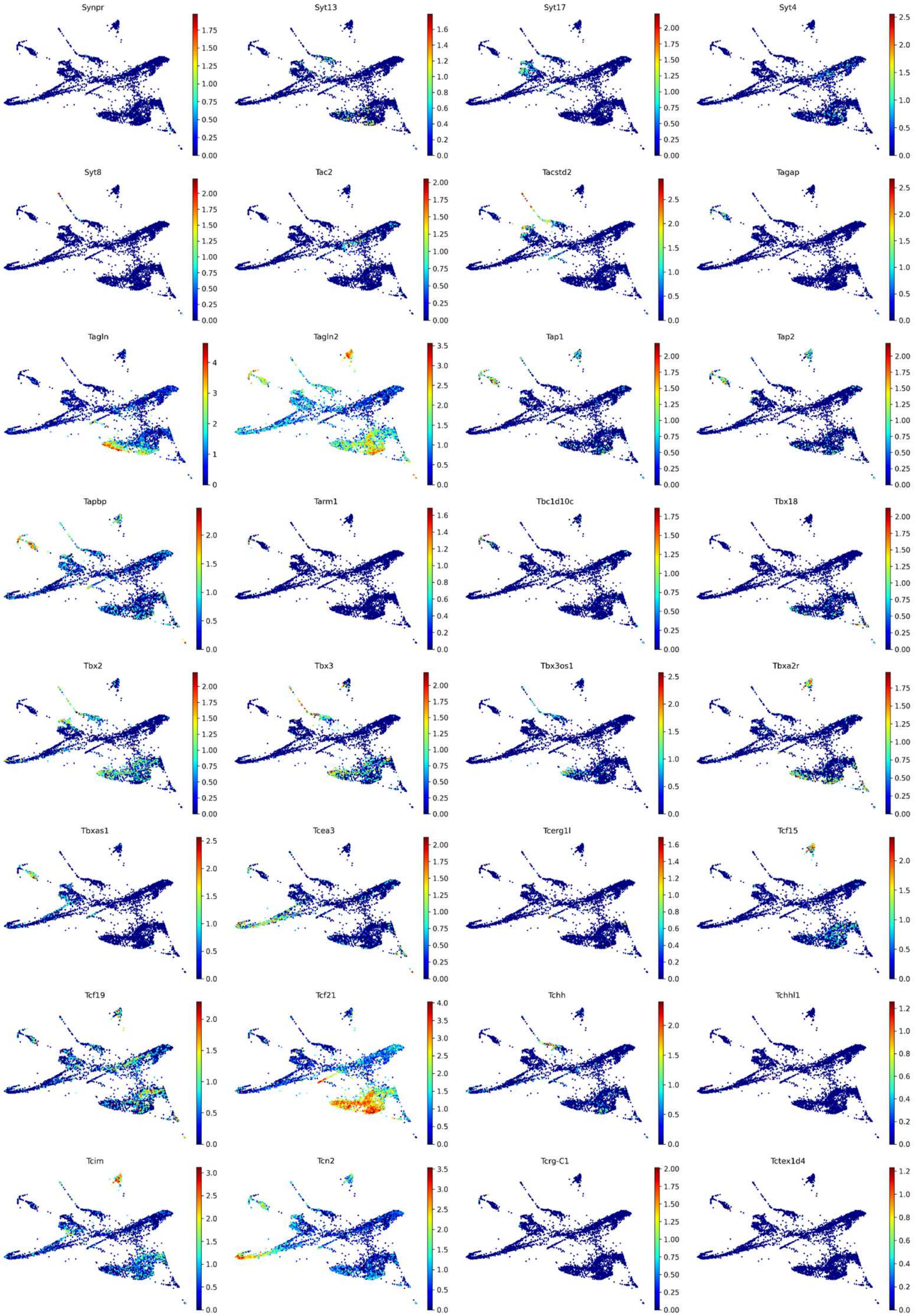

Supplementary Figure S5-86.

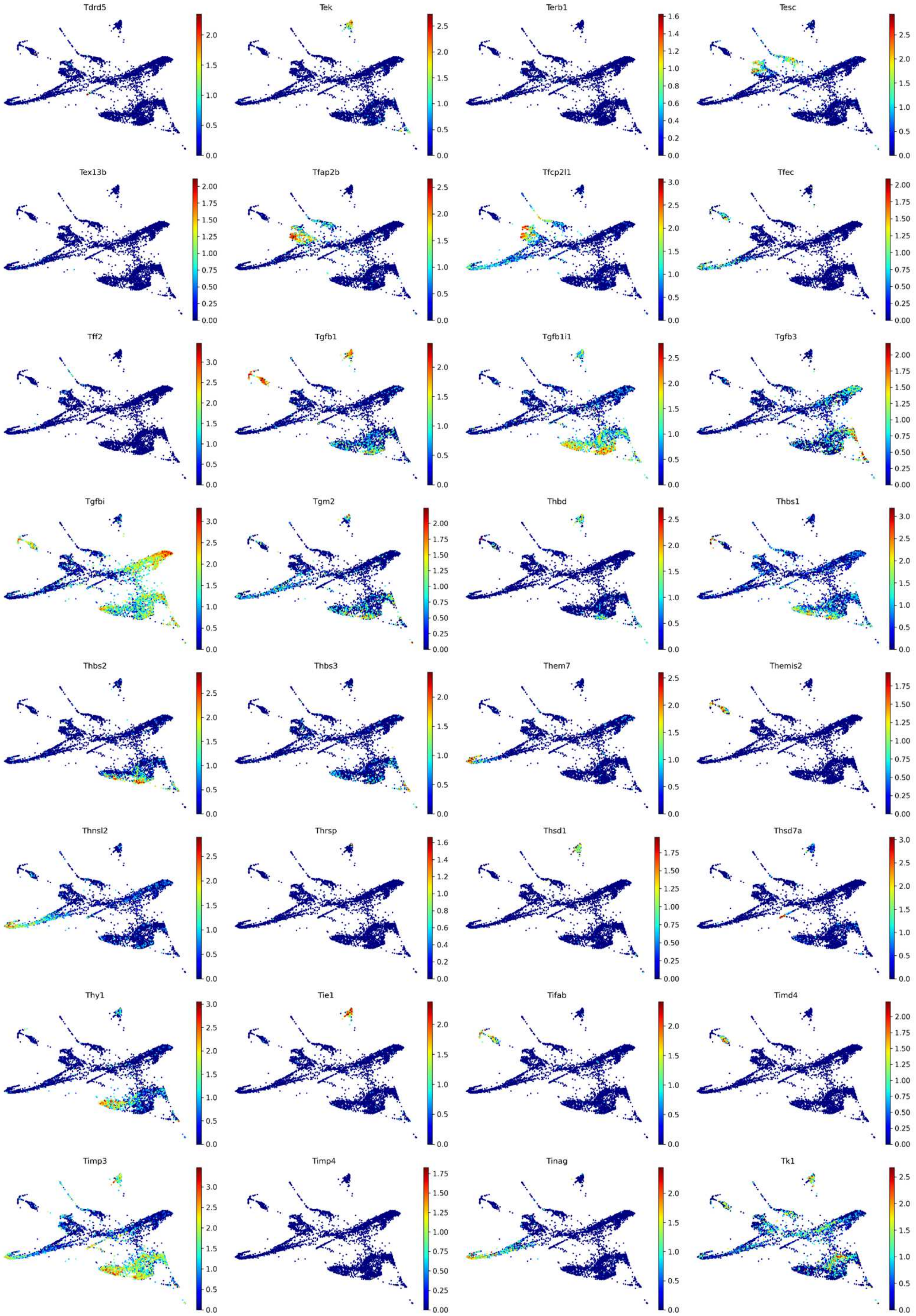

Supplementary Figure S5-87.

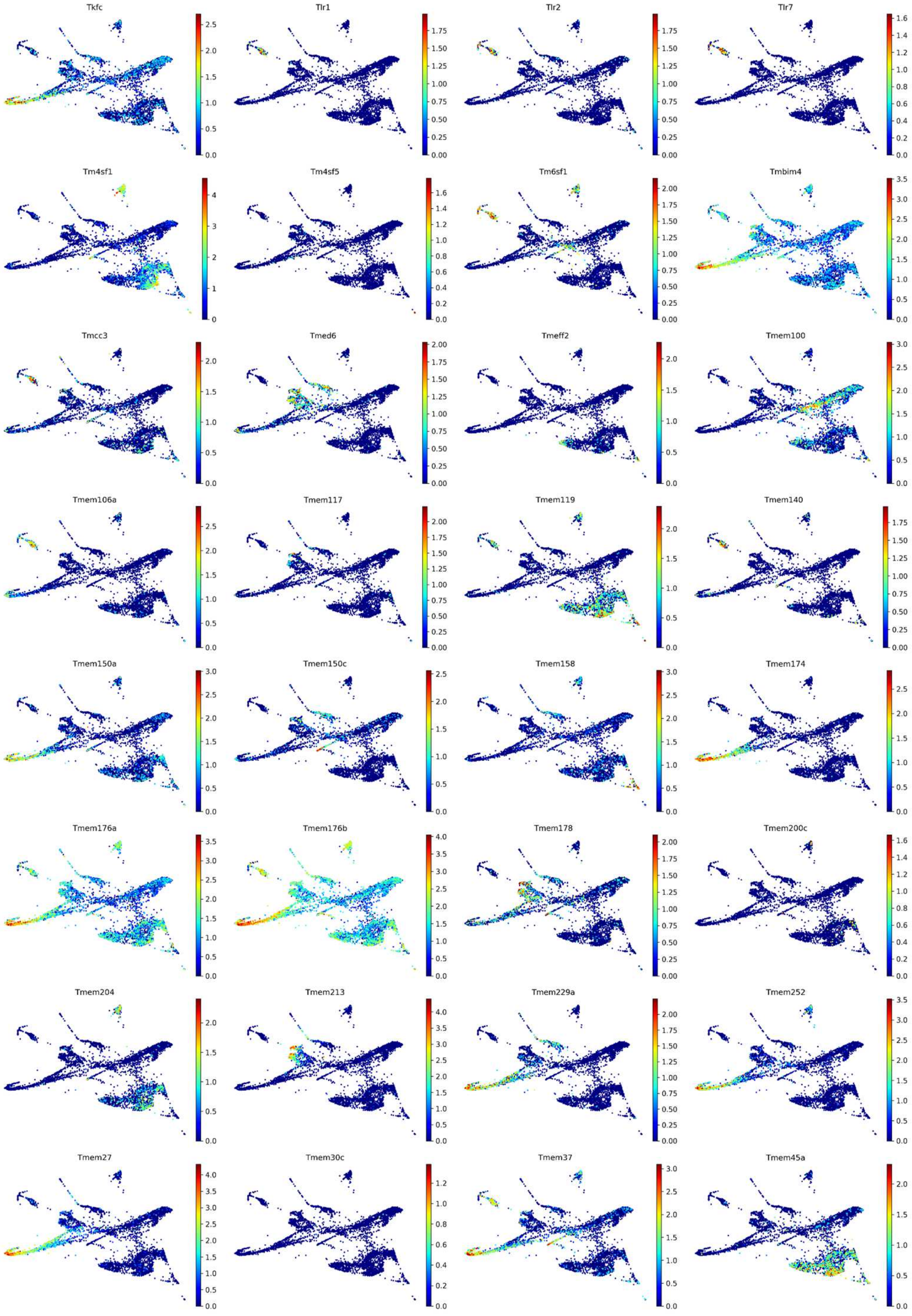

Supplementary Figure S5-88.

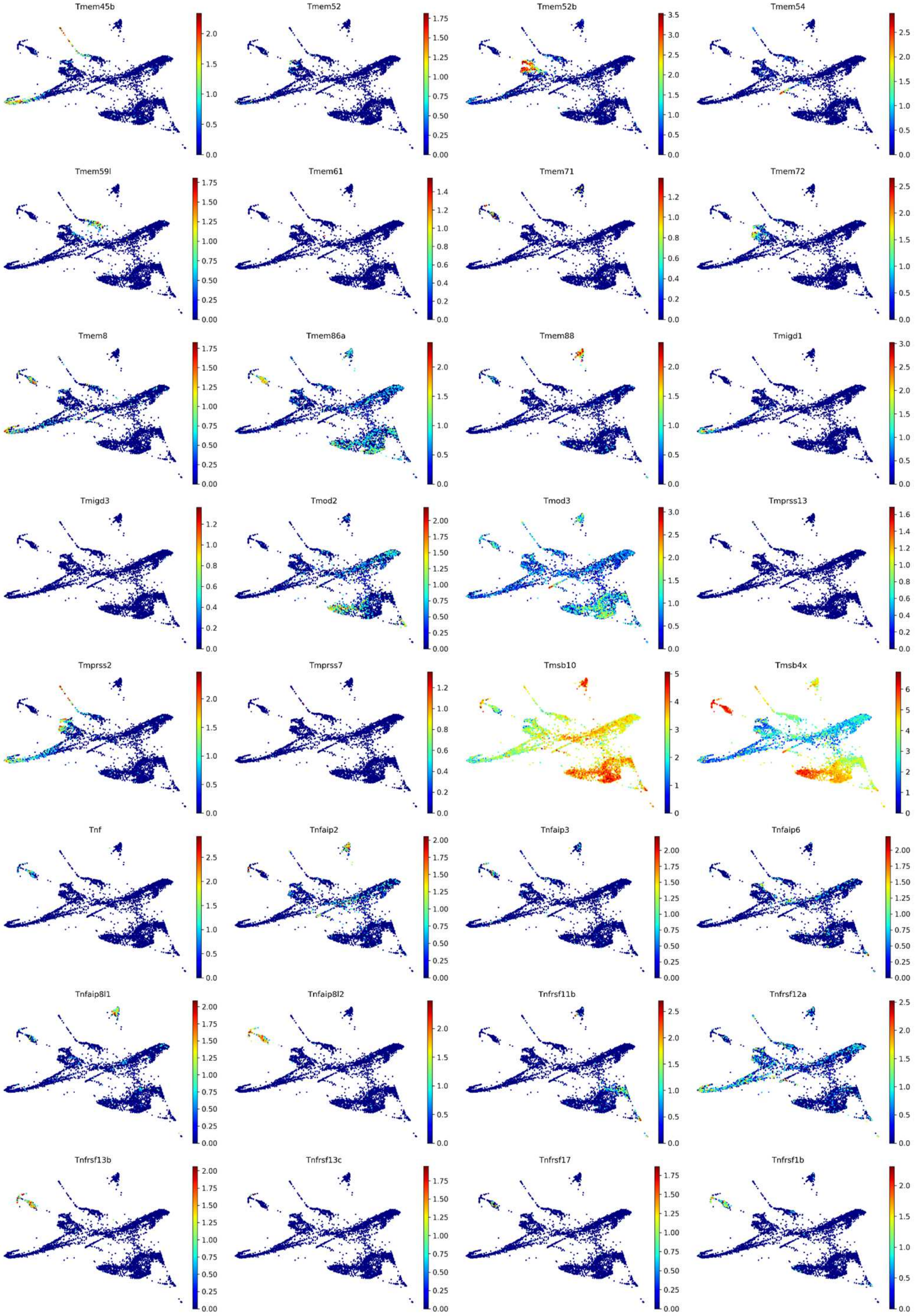

Supplementary Figure S5-89.

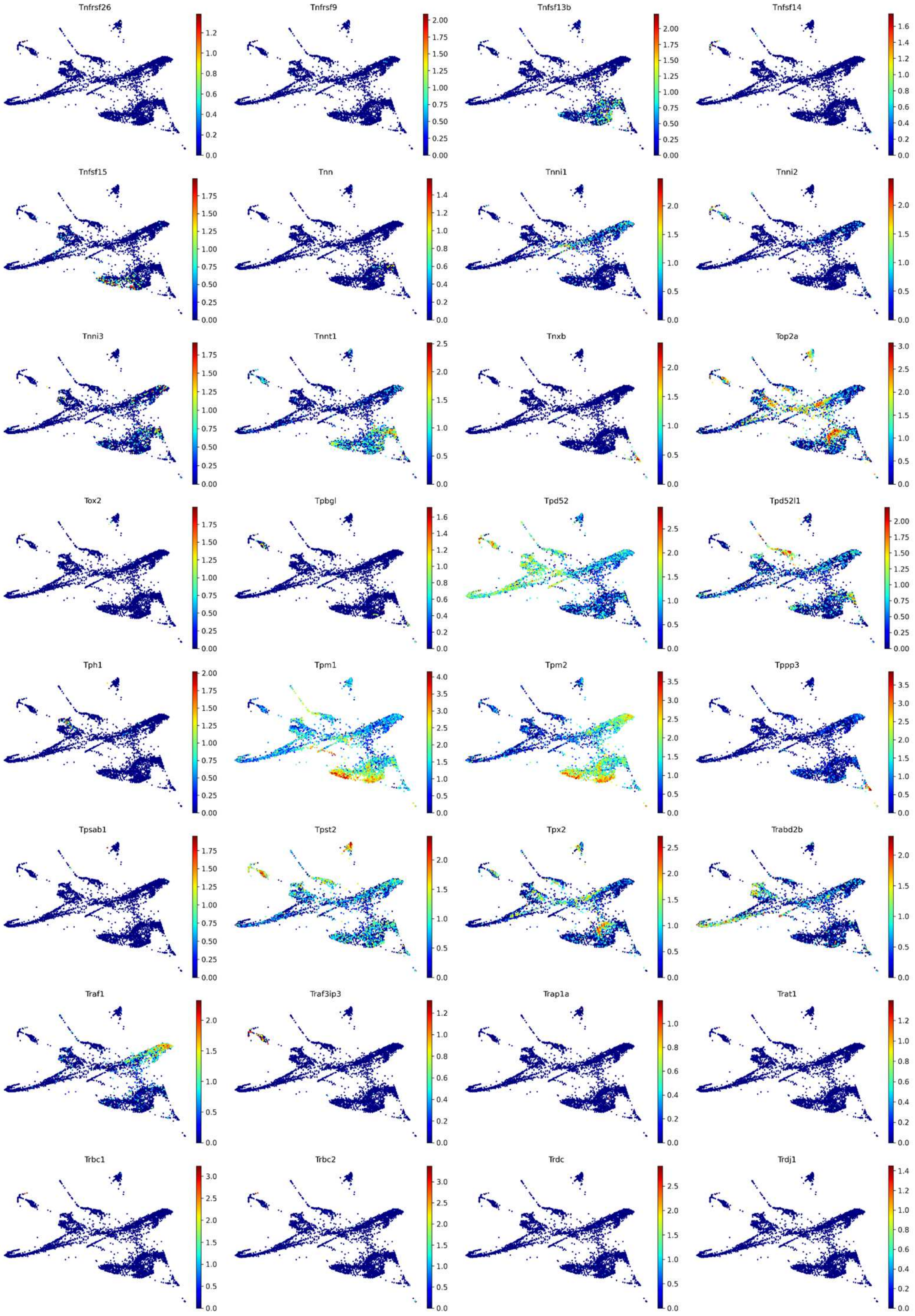

Supplementary Figure S5-90.

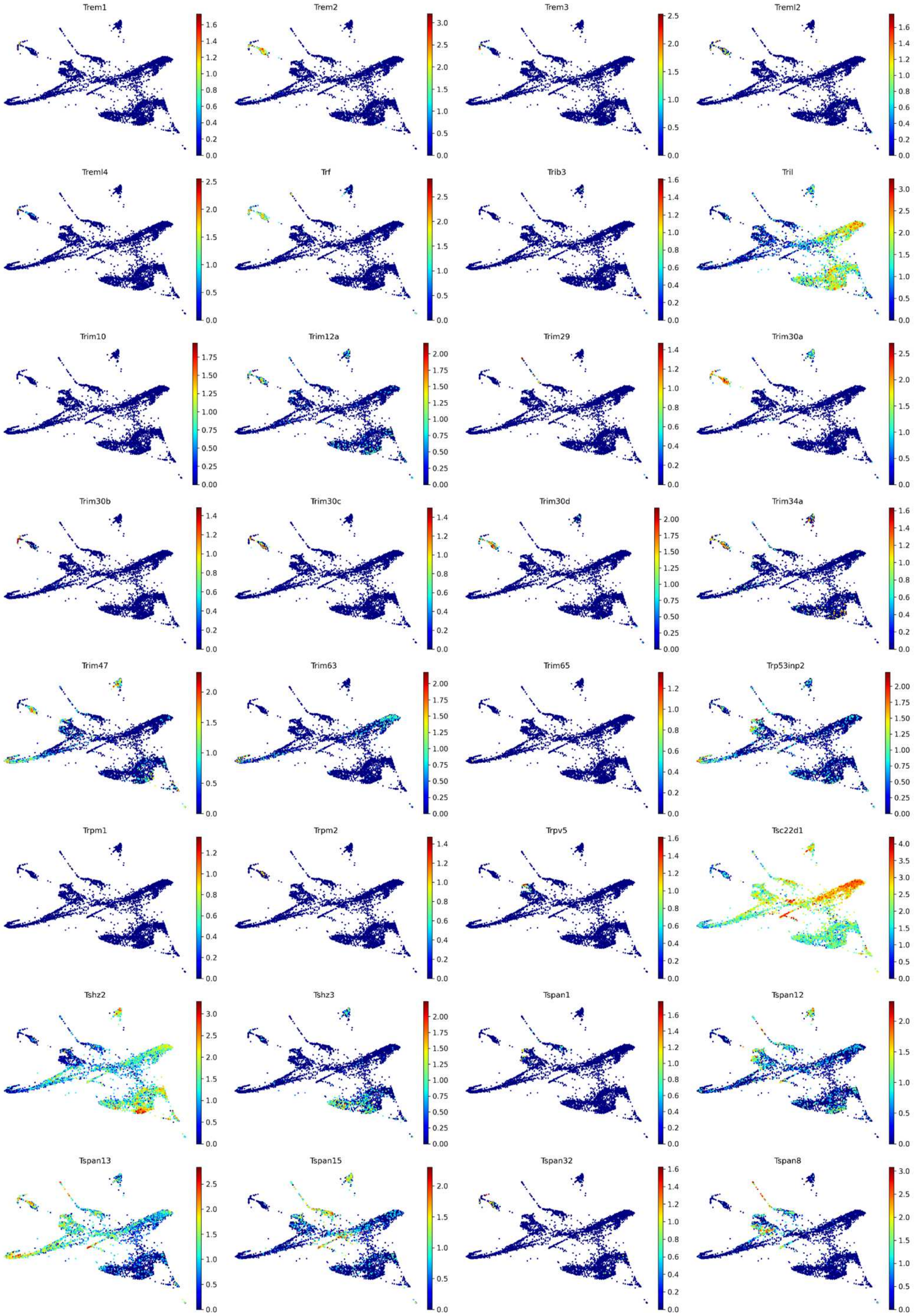

Supplementary Figure S5-91.

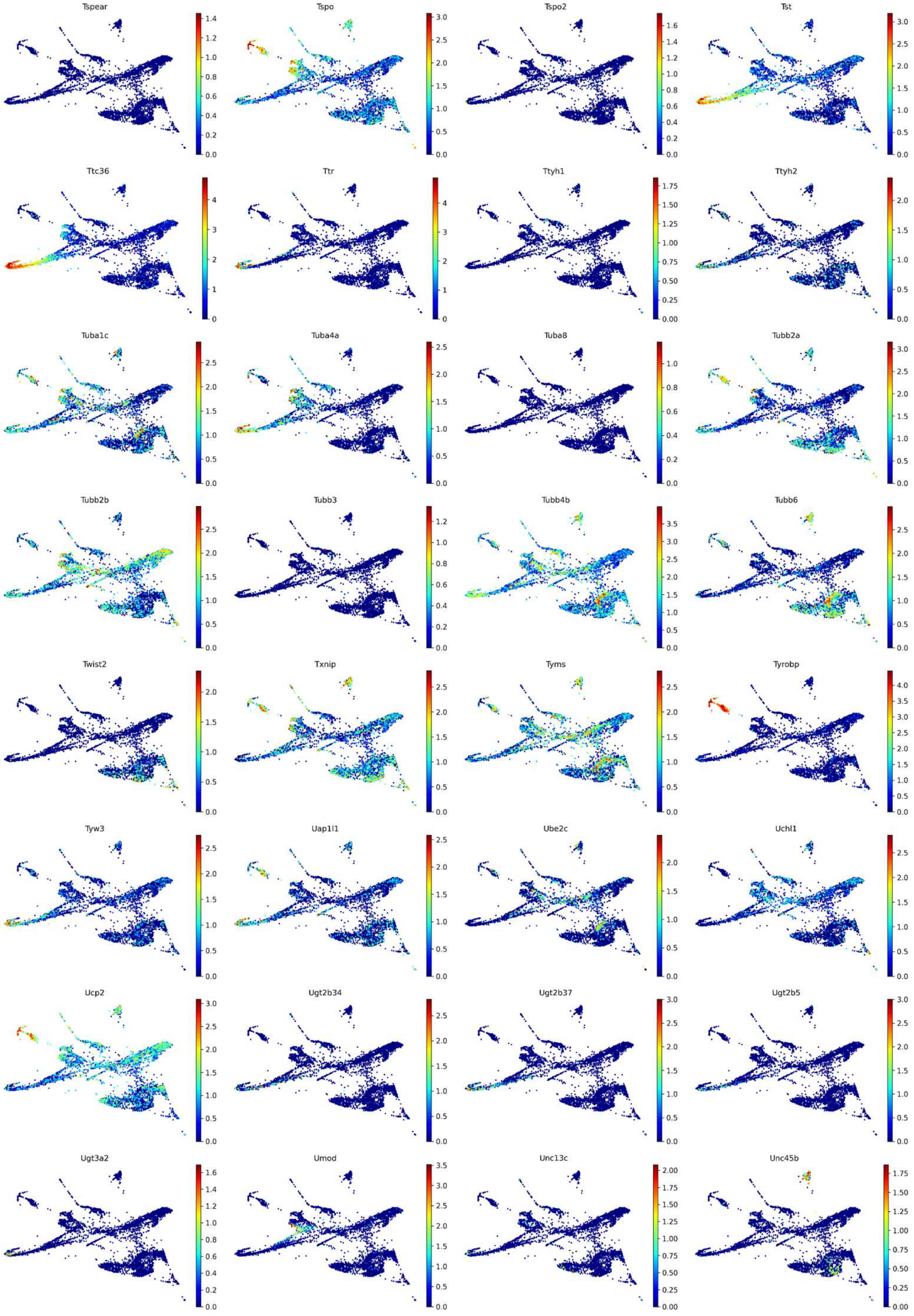

Supplementary Figure S5-92.

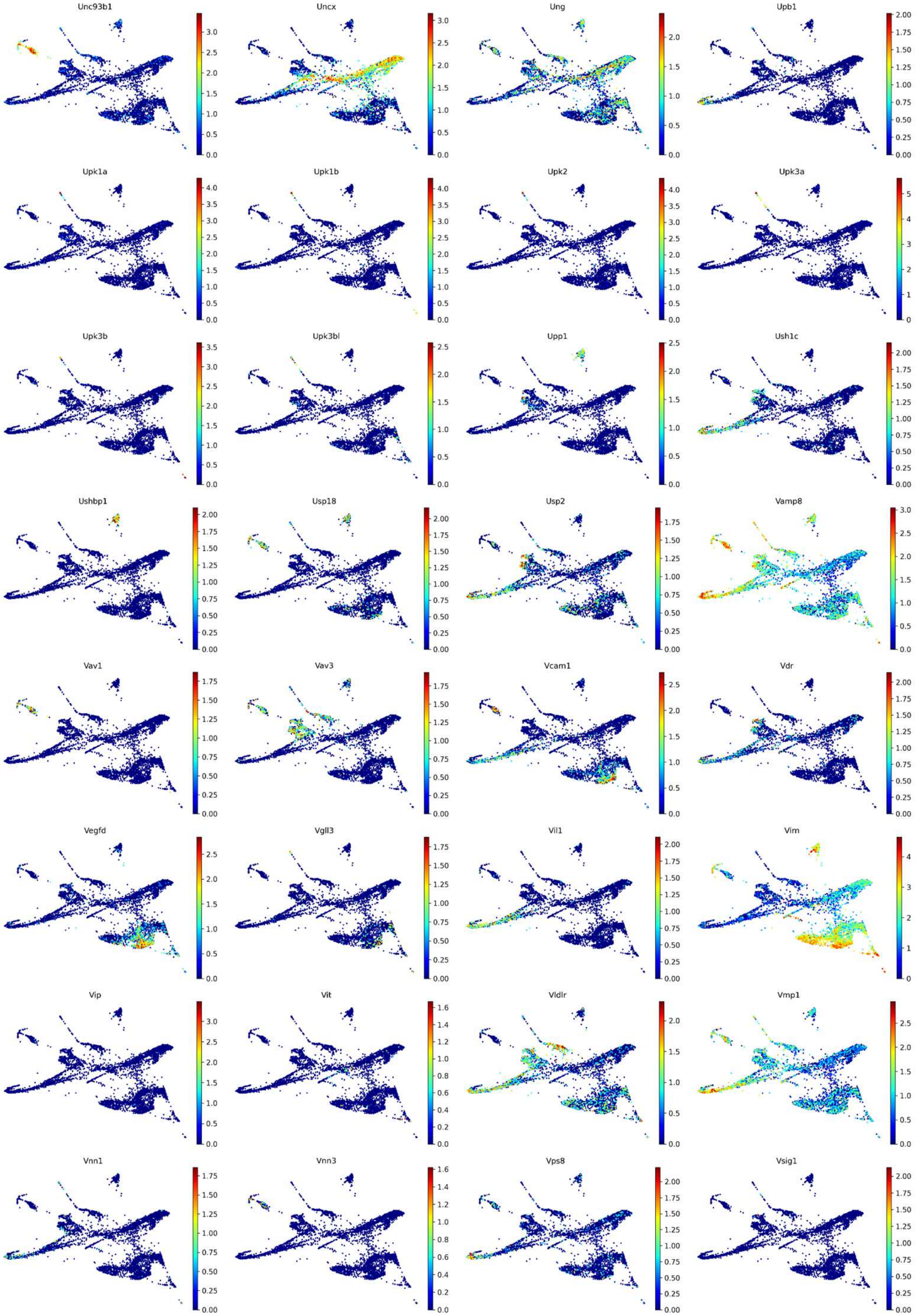

Supplementary Figure S5-93.

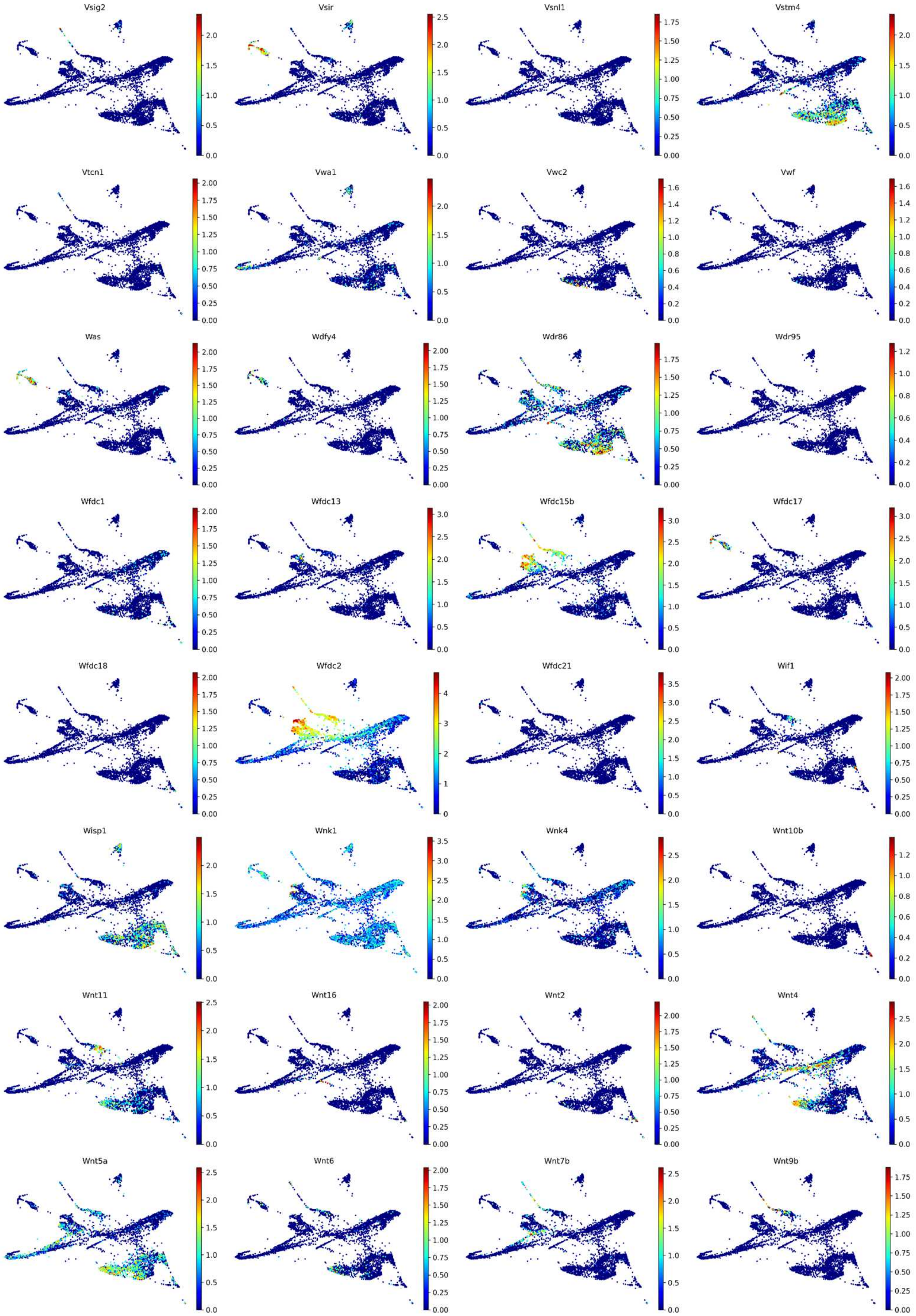

Supplementary Figure S5-94.

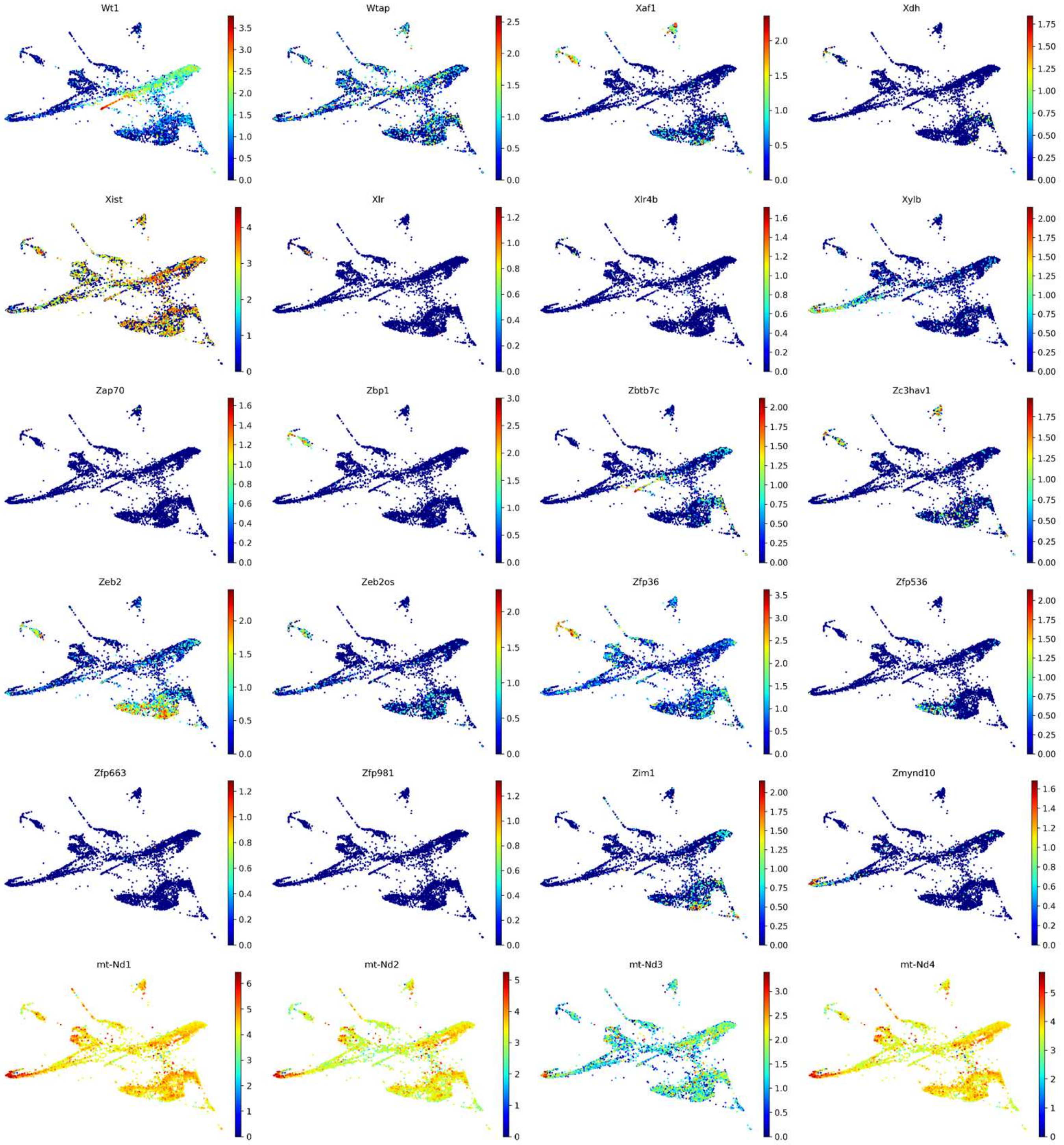

Expression patterns of highly variable genes.
Expression pattern of 3,000 highly variable genes, which were extracted in the pre-process of the scRNA-seq data (Supplementary Fig. S1**b**), are summarized in alphanumeric order. All data were analyzed using Scanpy version 1.4.4.post1 (https://scanpy.readthedocs.io/en/stable/).
