# Supplementary material for: Single cell RNA sequencing uncovers cellular developmental sequences and novel potential intercellular communications in embryonic kidney
Source: Sci Rep. 2021 Jan 8;11:73. doi: 10.1038/s41598-020-80154-y (PMC7794461; doi:10.1038/s41598-020-80154-y)
Supplement: Supplementary file 6 — Supplementary Information 6. [file 41598_2020_80154_MOESM6_ESM.pdf]

Supplementary Figure S6.

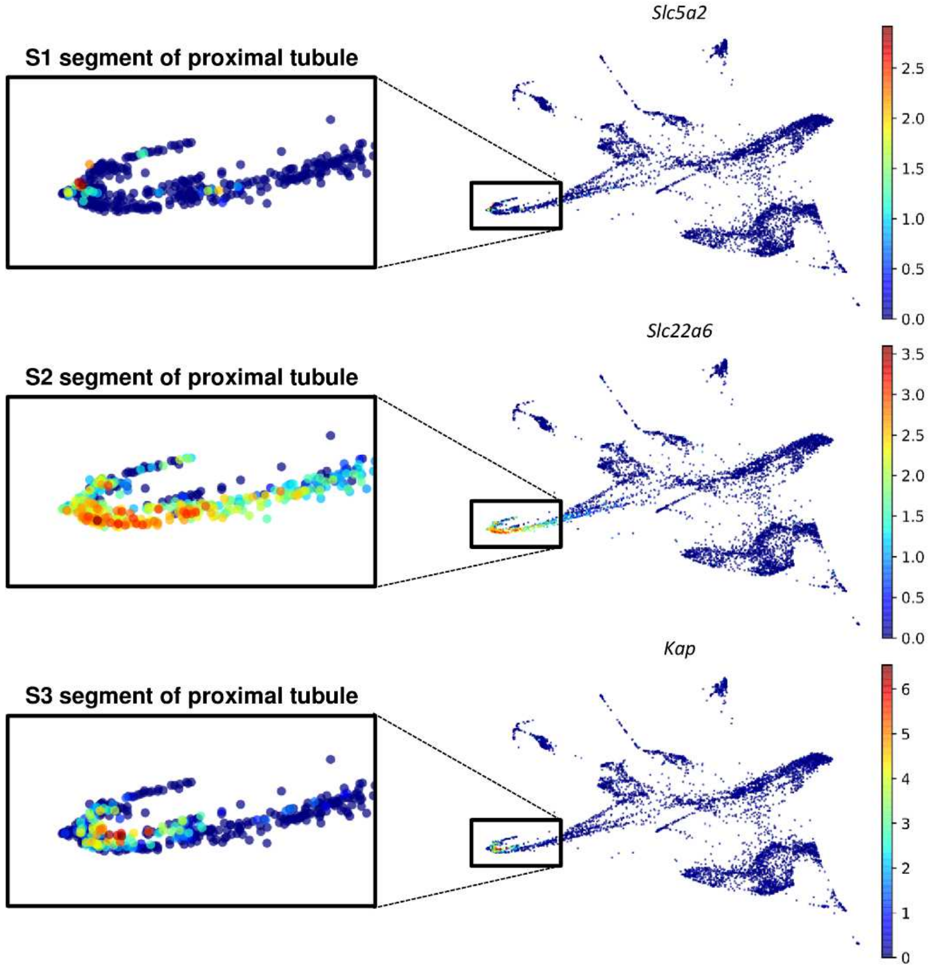

Expression patterns of segment specific marker genes for proximal tubules.

Expression of *Slc5a2*, *Slc22a6*, and *Kap* — makers of S1, S2, and S3 segment, respectively — are shown. All data were analyzed using Scanpy version 1.4.4.post1 (https://scanpy.readthedocs.io/en/stable/).

Supplementary Figure S7.

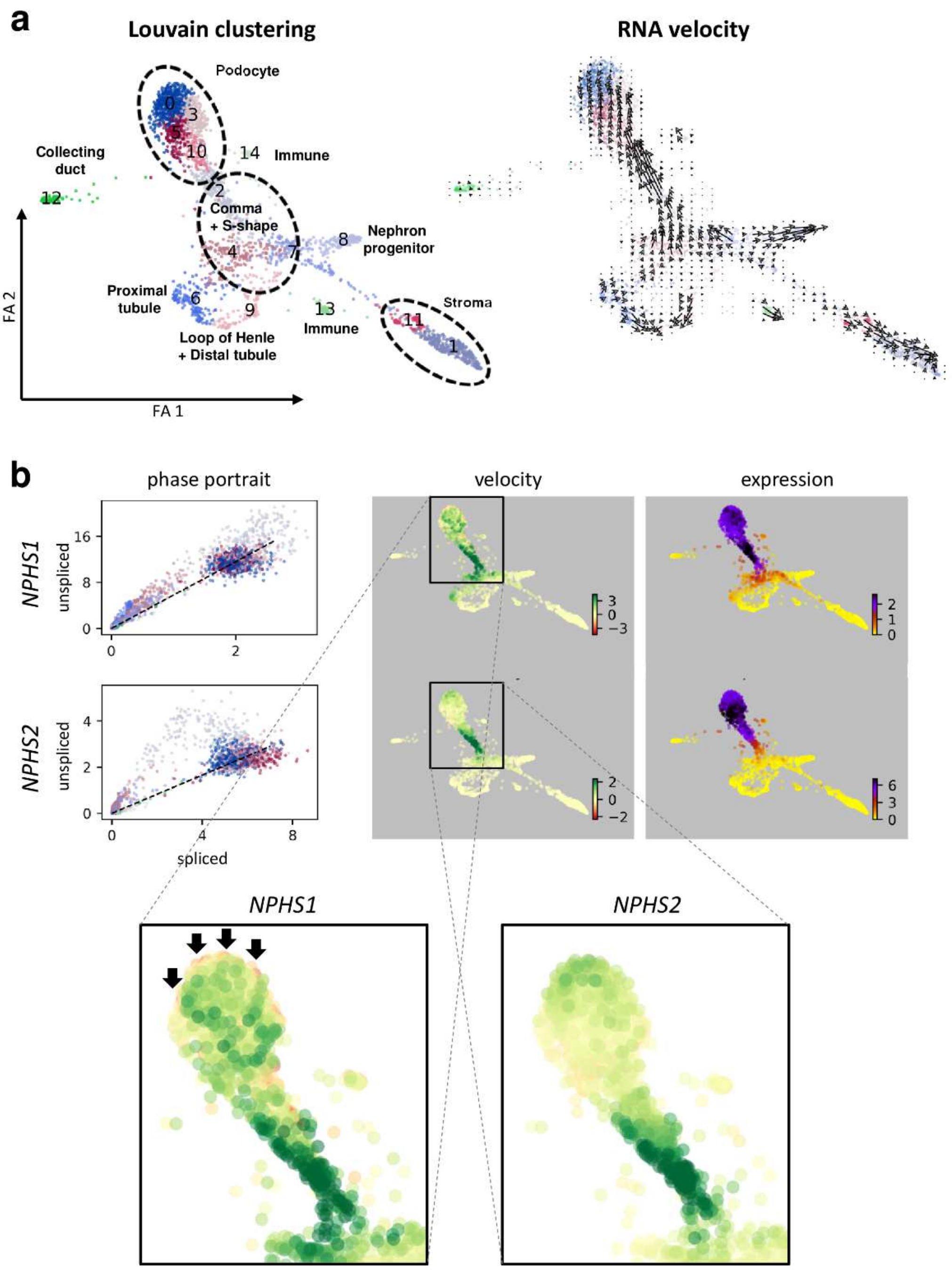

Analyses of scRNA-seq data obtained from human embryonic kidney.
(**a**) Cells were mapped by PAGA-initialized ForceAtlas2. Clusters were annotated based on differentially expressed features shown in Supplementary Table S2. (**b**) Grid-embedded RNA velocities are overlaid onto the map. (**c**) The phase portrait, RNA velocity, and expression levels of *NPHS1* and *NPHS2* are summarized. Dot colors in the phase portraits correspond to cluster colors in Supplementary Fig. S7**a**. Dotted lines in the phase portraits indicate the steady-state

ratio. Colors of the velocity plot indicate where the cells were allocated in the corresponding phase portraits. In the magnified RNA velocity map, arrows indicate negative velocity of *NPHS1* in the podocyte cluster. All data were analyzed using Scanpy version 1.4.4.post1 (https://scanpy.readthedocs.io/en/stable/) and scVelo version 0.1.25 (https://scvelo.readthedocs.io/).

Supplementary Figure S8.

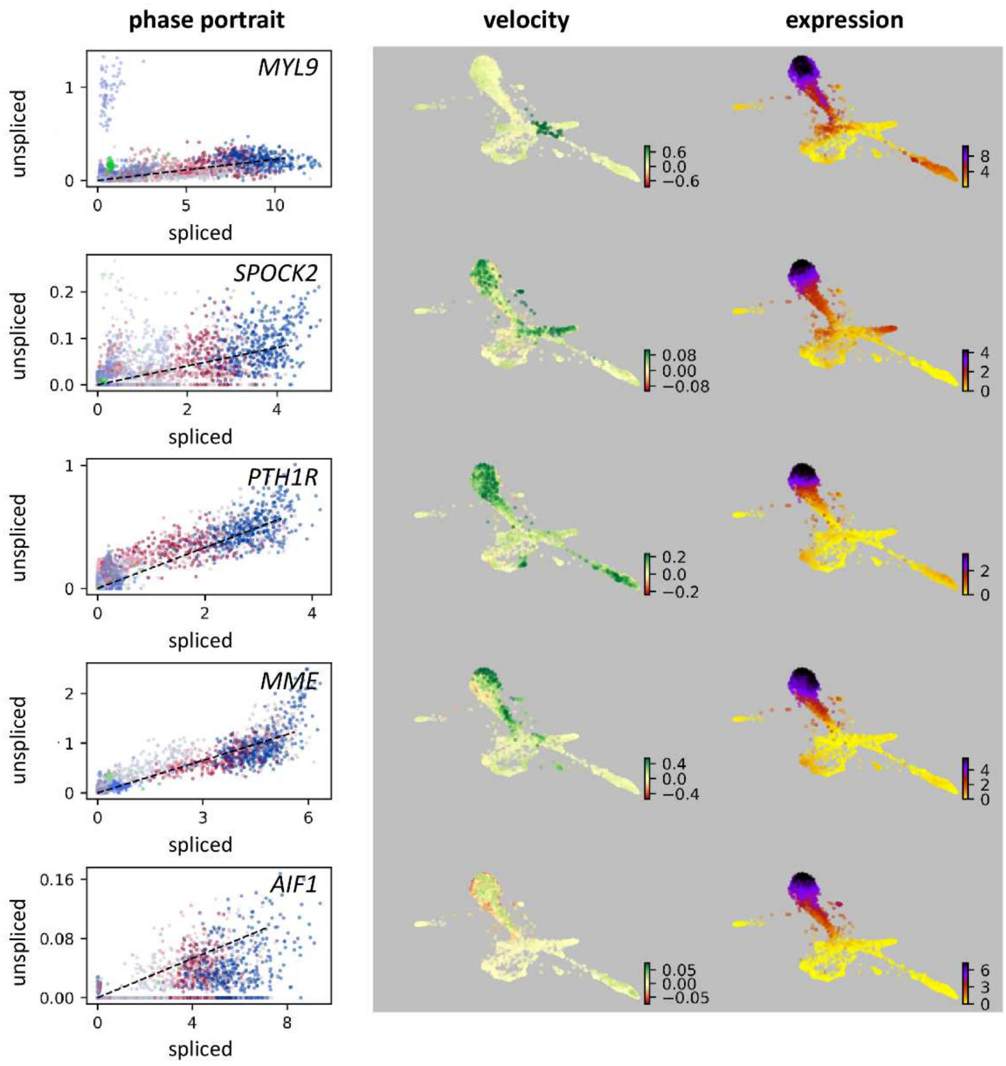

RNA velocity of genes which were highly expressed at the tip of podocyte clusters in human embryonic kidney data.

RNA velocity of genes which were highly expressed at the tip of podocyte cluster in human embryonic kidney scRNA-seq data (cluster 0 in Supplementary Fig. S7**a** and Supplementary Table S2) are shown. All data were analyzed using Scanpy version 1.4.4.post1 (https://scanpy.readthedocs.io/en/stable/) and scVelo version 0.1.25 (https://scvelo.readthedocs.io/).

Supplementary Figure S9.

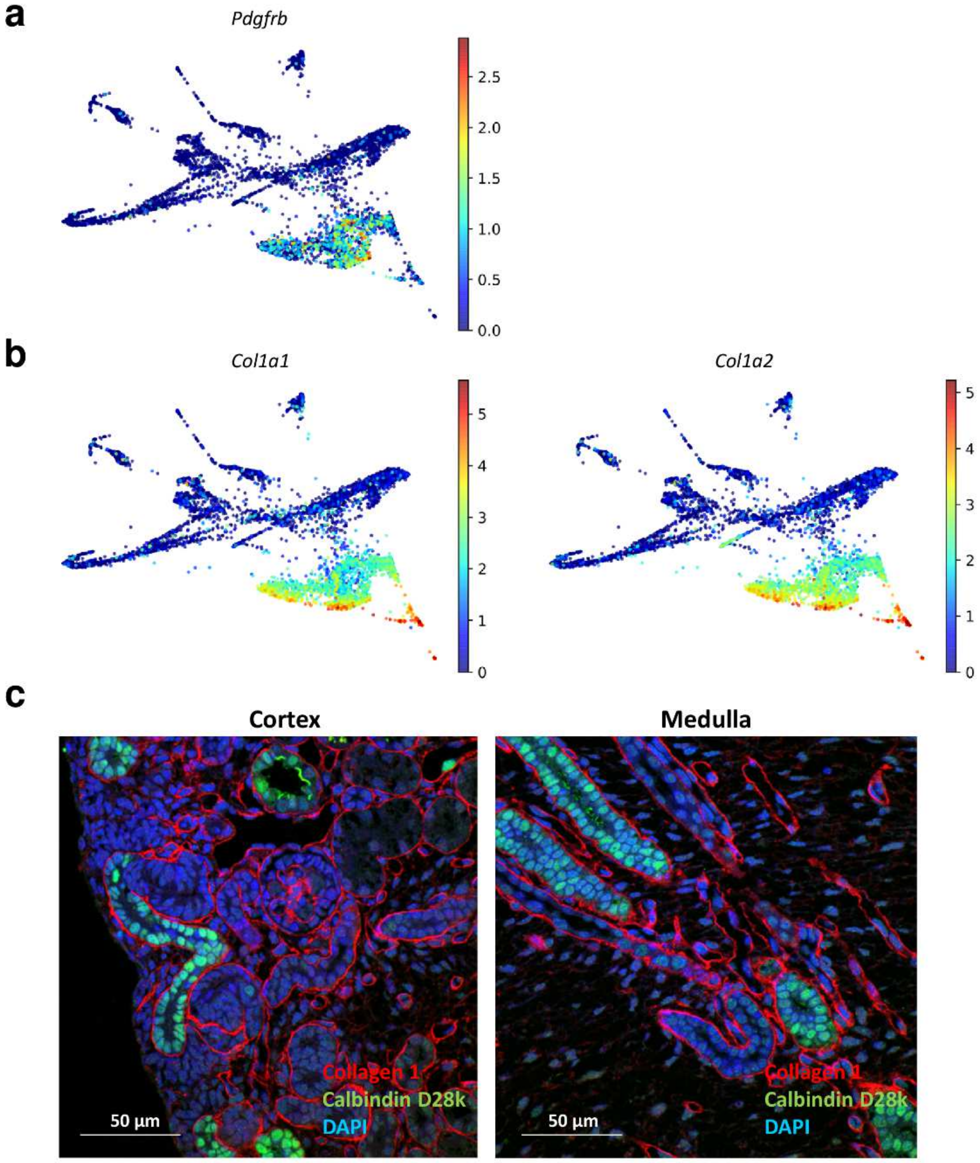

Expression patterns of platelet derived growth factor receptor beta *(Pdgfrb)*, collagen type 1 alpha 1 (*Col1a1),* and collagen type 1 alpha 2 (*Col1a2*).

(**a** and **b**) Expression patterns of *Pdgfrb*, *Col1a1,* and *Col1a2* are shown. (**c**) Embryonic kidney section at day 18.5 was immunohistochemically stained with collagen 1 in red, calbindin D28k in green, and DAPI in blue (scale bar: 50 µm). All data were analyzed using Scanpy version 1.4.4.post1 (https://scanpy.readthedocs.io/en/stable/).

Supplementary References

1. Günzel, D. & Yu, A. S. L. Claudins and the Modulation of Tight Junction Permeability. *Physiol Rev.* **93**, 525–569 (2013).
2. Kiuchi-Saishin, Y. *et al.* Differential Expression Patterns of Claudins, Tight Junction Membrane Proteins, in Mouse Nephron Segments. *J Am Soc Nephrol.* **13**, 875-886 (2002).
3. DiRocco, D. P., Kobayashi, A., Taketo, M. M., McMahon, A. P. & Humphreys, B. D. Wnt4/β-catenin signaling in medullary kidney myofibroblasts. *J Am Soc Nephrol.* **24**, 1399–1412 (2013).
4. Guimarães-Camboa, N. *et al.* Pericytes of Multiple Organs Do Not Behave as Mesenchymal Stem Cells In Vivo. *Cell Stem Cell* **20**, 345-359 (2017).
5. Chang-Panesso, M. & Humphreys, B. D. CD248/Endosialin: A novel pericyte target in renal fibrosis. *Nephron* **131**, 262–264 (2016).
6. Tirosh, I. *et al.* Dissecting the multicellular ecosystem of metastatic melanoma by single-cell RNA-seq. *Science.* **352**, 189–196 (2016).
